# Kinetically-driven reactivity of sulfinylamines enables direct conversion of carboxylic acids to sulfinamides†

**DOI:** 10.1039/d3sc04727j

**Published:** 2023-10-09

**Authors:** Hang T. Dang, Arka Porey, Sachchida Nand, Ramon Trevino, Patrick Manning-Lorino, William B. Hughes, Seth O. Fremin, William T. Thompson, Shree Krishna Dhakal, Hadi D. Arman, Oleg V. Larionov

**Affiliations:** a Department of Chemistry, The University of Texas at San Antonio One UTSA Circle San Antonio TX 78249 USA oleg.larionov@utsa.edu

## Abstract

Sulfinamides are some of the most centrally important four-valent sulfur compounds that serve as critical entry points to an array of emergent medicinal functional groups, molecular tools for bioconjugation, and synthetic intermediates including sulfoximines, sulfonimidamides, and sulfonimidoyl halides, as well as a wide range of other S(iv) and S(vi) functionalities. Yet, the accessible chemical space of sulfinamides remains limited, and the approaches to sulfinamides are largely confined to two-electron nucleophilic substitution reactions. We report herein a direct radical-mediated decarboxylative sulfinamidation that for the first time enables access to sulfinamides from the broad and structurally diverse chemical space of carboxylic acids. Our studies show that the formation of sulfinamides prevails despite the inherent thermodynamic preference for the radical addition to the nitrogen atom, while a machine learning-derived model facilitates prediction of the reaction efficiency based on computationally generated descriptors of the underlying radical reactivity.

## Abstract 2

Sulfinamides can now be readily accessed from carboxylic acids and amines in a direct decarboxylative reaction enabled by the kinetically-driven reactivity of sulfinylamines and acridine photocatalysis.

## Graphical abstract

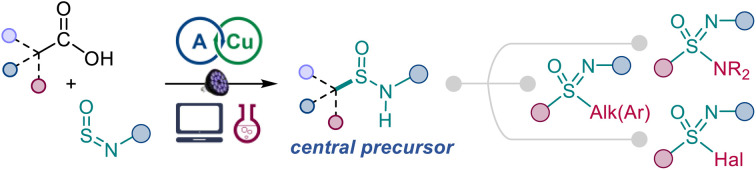

## Introduction

Development of new synthetic transformations is key to enabling access to unexplored chemical space and reducing overreliance on inefficient multistep processes.^1^

**Fig. 1:**
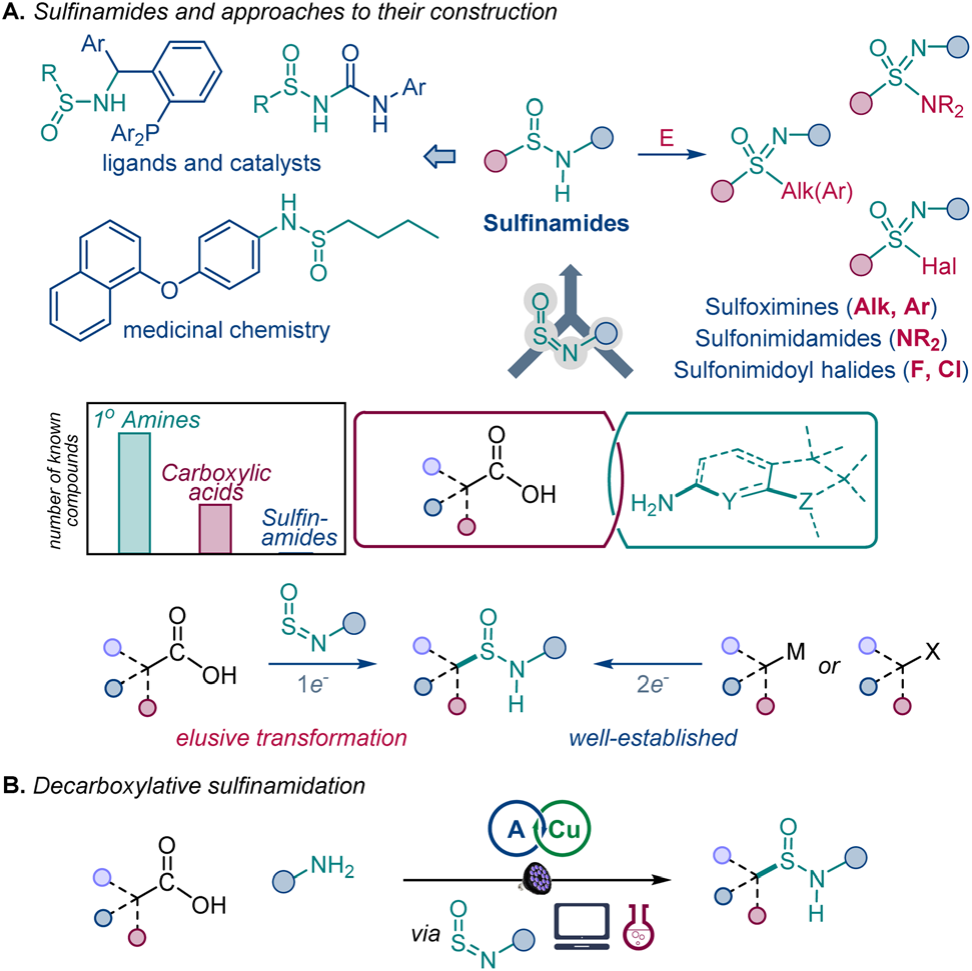
Direct decarboxylative conversion of carboxylic acids to sulfinamides.

The diversity of the reactivities and physicochemical properties of functional groups containing the S=O bond has made it one of the most important structural elements in organic synthesis,^2^ materials research,^3^ and drug discovery,^4^ exemplified by its extensive use as a bioisosteric replacement for the carbonyl group.^4,5^ The sulfinyl group (S^IV^=O) has the potential to become a central synthetic linchpin, because the oxidation state of the sulfur atom can be readily adjusted, unlocking a broad organosulfur chemical space that spans all major sulfur oxidation states and enables rapid functional group diversification of advanced synthetic and medicinal targets. Furthermore, the polarity and Lewis basicity of the sulfinyl group have made it a key structural motif in medicinal chemistry and catalysis.^4,6^ However, installation of the sulfinyl group remains a challenge and is typically achieved by chemical manipulation of preinstalled sulfur functionalities. Sulfinamides have emerged as a potentially important entry point to a broad range of sulfinyl and sulfonyl chemical space (Fig. 1).

In particular, sulfinamides provide facile synthetic access to sulfoximines, sulfonimidamides, and sulfonimidoyl halides that have increasingly important roles in drug discovery and click chemistry and are difficult to generate by other methods, in addition to the widely medicinally used sulfonamides.^7^ Sulfinamides also have numerous applications as directing and auxiliary groups in organic synthesis^8^ and as ligands and catalysts.^9^ Despite their growing importance, synthetic access to sulfinamides is confined to methods that rely on two-electron reactivity typically observed in nucleophilic substitutions and related reactions.^10^ Importantly, recent studies have shown that additions to sulfinylamines represent a promising approach to sulfinamides and related organosulfur compounds.^11^ However, one-electron approaches to sulfinamides remain rare and require precursors that are not readily available, limiting the scope of accessible sulfinamide products.^12^

We envisioned that a reaction that directly converts carboxylic acids to sulfinamides by a one-electron process would significantly improve access to sulfinyl and sulfonyl compounds because of the broad range of structural diversity, molecular complexity, and abundance of carboxylic acids.^13^ Additionally, given the major role of S=O-centered functional groups as bioisosteres of the carboxylic group and other carbonyls,^13^ the reaction would enable rapid generation of their structural analogues in the context of late-stage functionalization and drug discovery. However, direct conversion of carboxylic acids to other functional groups remains challenging because of the high oxidation potentials and reactivity of carboxylic acids that prevent decarboxylative radical generation with commonly used catalytic systems and reagents or requires preactivation and harsh reaction conditions that are incompatible with oxidizable functionalities, *e.g.*, anilines and organosulfur compounds, while the scope of available direct decarboxylative transformations remains limited.^13*b*,*c*,14–16^

Acridine photocatalysis based on the 9-arylacridine structure (A1–A3) has recently emerged as a powerful catalytic platform for the efficient generation of alkyl radicals from carboxylic acids that has enabled direct conversion of carboxylic acids to a variety of functionalities.^13*b*,*c*,15,16^ However, the efficiency of decarboxylative processes remains unpredictable especially in the context of previously unknown synthetic transformations.

We report herein the development of a direct decarboxylative conversion of carboxylic acids to sulfinamides in an acridine-catalyzed reaction with sulfinylamines that are readily accessed from amines, thus for the first time merging the broad chemical space of the two of the most common classes of organic compounds. We show that the observed positional selectivity of the radical addition occurs despite the thermodynamic preference for the addition to the nitrogen atom, and the underlying radical reactivity can be described by a machine learning (ML)-based model, permitting prediction of the reaction efficiency to facilitate synthetic planning and implementation.

## Results and discussion

**Fig. 2:**
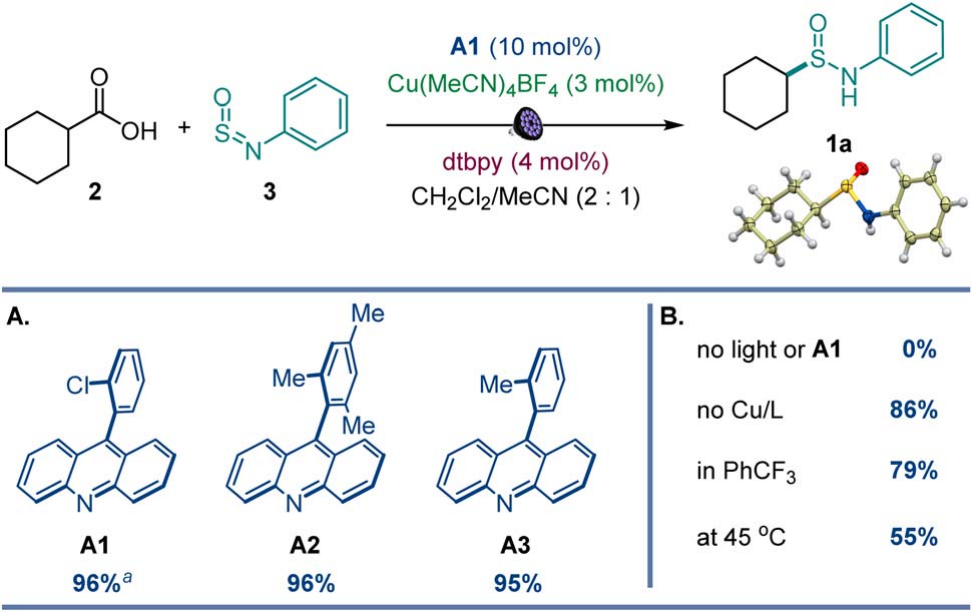
Reaction conditions for the photocatalytic direct decarboxylative sulfinamidation: carboxylic acid 2 (0.2 mmol), sulfinylamine 3 (0.4 mmol, accessed from aniline by activation with thionyl chloride), A1 (10 mol%), Cu(MeCN)_4_BF_4_ (3 mol%), dtbpy (4 mol%), CH_2_Cl_2_/MeCN (2 : 1, 2 mL), LED light (400 nm), 16 h. (A) Acridine photocatalysts. (B) Influence of other reaction parameters. Yield was determined by ^1^H NMR spectroscopy with 1,3,5-trimethoxybenzene as an internal standard. ^*a*^Isolated yield. dtbpy = 4,4′-di-*tert*-butyl-2,2′-bipyridine.

After initial optimization studies, we found that sulfinamide 1a can be efficiently produced from carboxylic acids 2 and sulfinylamine 3 (accessed from aniline prior to the reaction by activation with thionyl chloride^17^) in the presence of acridine catalyst A1 and a copper co-catalyst with 400 nm LED irradiation (Fig. 2). Acridines A1–A3 were all competent catalysts (Fig. 2A), and the reaction required light and the acridine catalyst to proceed. The reaction could also be conducted without the copper co-catalyst and in other solvents (Fig. 2B), albeit with lower yields. Importantly, other photocatalysts, *e.g.*, *N*-phenyl 9-mesitylacridinium catalyst, as well as Ir- and Ru-based photocatalysts, 4CzIPN, and eosin Y did not catalyze the carboxylic acid to sulfinamide conversion (Table S1†).

**Scheme 1:**
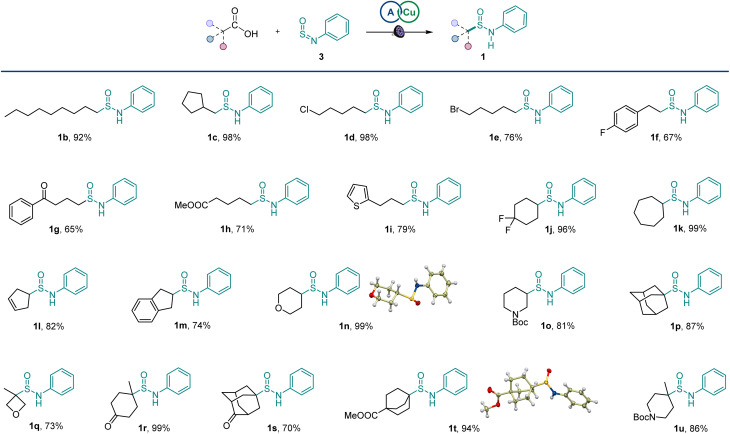
Scope of the direct decarboxylative sulfinamidation. Reaction conditions: carboxylic acid 2 (0.2 mmol), sulfinylamine 3 (0.4 mmol, accessed from aniline by activation with thionyl chloride), A1 (10 mol%), Cu(MeCN)_4_BF_4_ (3 mol%), dtbpy (4 mol%), CH_2_Cl_2_/MeCN (2 : 1, 2 mL), LED light (400 nm), 16 h.

The scope of carboxylic acids was examined next in a reaction with sulfinylamine 3 (Scheme 1). Primary carboxylic acids were suitable substrates, providing an array of sulfinamides bearing chloro, bromo, aryl, ketone, ester, and heteroaryl groups (1b–1i). Secondary carboxylic acids were also competent coupling partners, including unsaturated and heterocyclic acids (1j–1o). Likewise, an assortment of tertiary carboxylic acids featuring medicinally important small ring and cage topologies could be efficiently converted to the corresponding sulfinamides (1p–1u) in good to excellent yields.

**Scheme 2:**
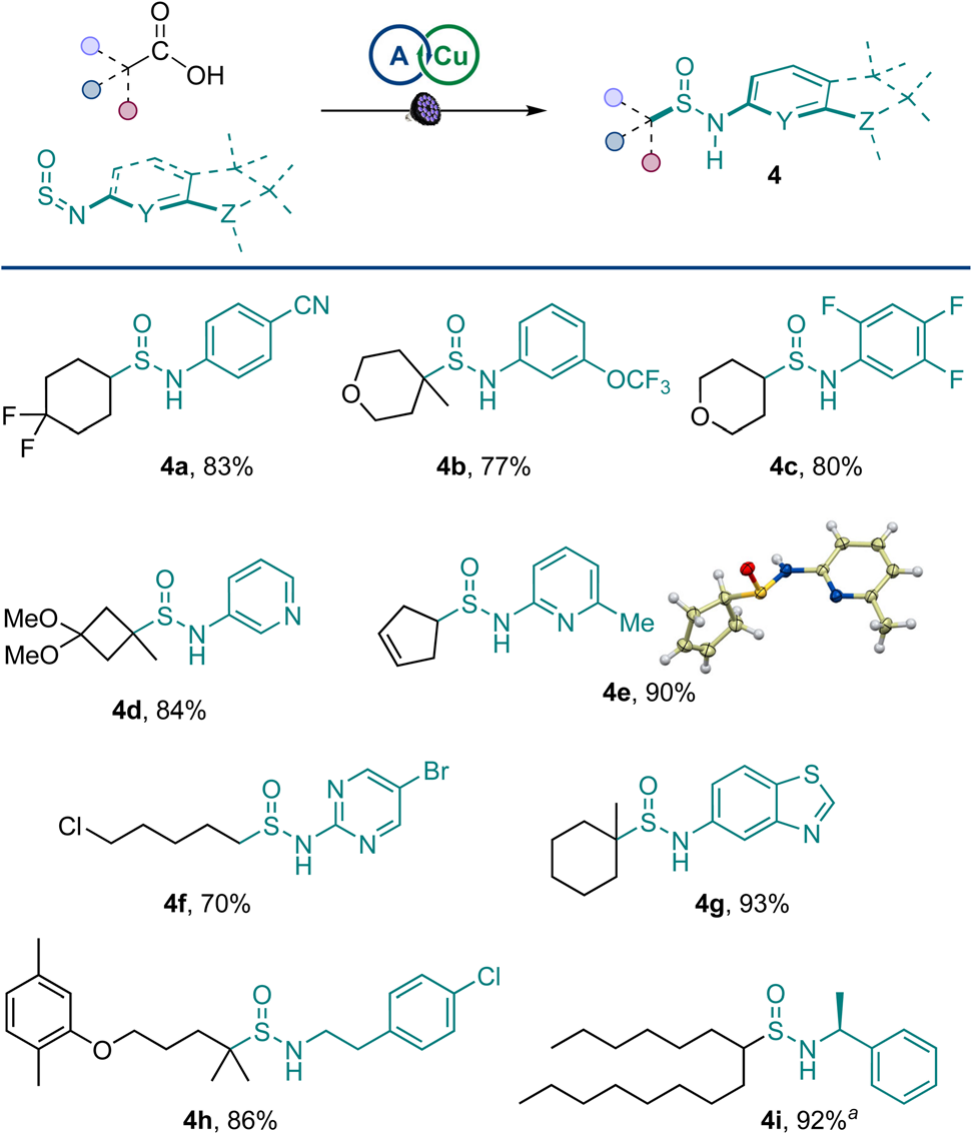
Scope of sulfinylamines. Reaction conditions: carboxylic acid (0.2 mmol), sulfinylamine (from the corresponding amine by activation with thionyl chloride, 0.4 mmol), A1 (10 mol%), Cu(MeCN)_4_BF_4_ (3 mol%), dtbpy (4 mol%), CH_2_Cl_2_/MeCN (2 : 1, 2 mL), LED light (400 nm), 16 h. ^*a*^1 : 1 dr.

A range of amines were pretreated with thionyl chloride, and the resulting sulfinylamines were also equally viable coupling partners, affording an array of structurally diverse sulfinamides 4a–4i in a reaction with primary, secondary, and tertiary carboxylic acids (Scheme 2) and enabling the merger of amines and carboxylic acids in the sulfinamidation reaction. Sulfinamides bearing aryl groups, as well as medically relevant substituted pyridine, pyrimidine, and benzothiazole rings were readily accessed. Similarly, aliphatic sulfinylamines were efficiently converted to the corresponding sulfinamides (4h and 4i).

**Scheme 3:**
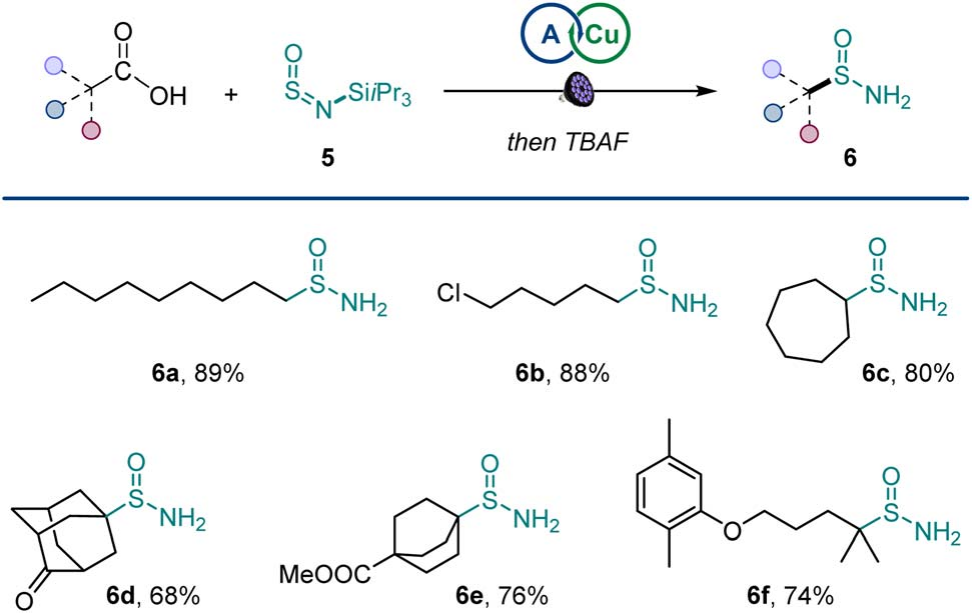
Scope of primary sulfinamides. Reaction conditions: carboxylic acid (0.2 mmol), sulfinylamine 5 (0.33 mmol, from TIPSNH_2_ by activation with thionyl chloride), A1 (10 mol%), Cu(MeCN)_4_BF_4_ (4 mol%), dtbpy (5 mol%), CH_2_Cl_2_/MeCN (2 : 1, 2 mL), LED light (400 nm), 16 h.

Additionally, primary sulfinamides could be produced by using silyl sulfinylamine 5 developed by Willis^11*j*^ with simple post-reaction fluoride treatment (Scheme 3), demonstrating excellent compatibility with primary, secondary, and tertiary carboxylic acids (6a–6f).

**Scheme 4:**
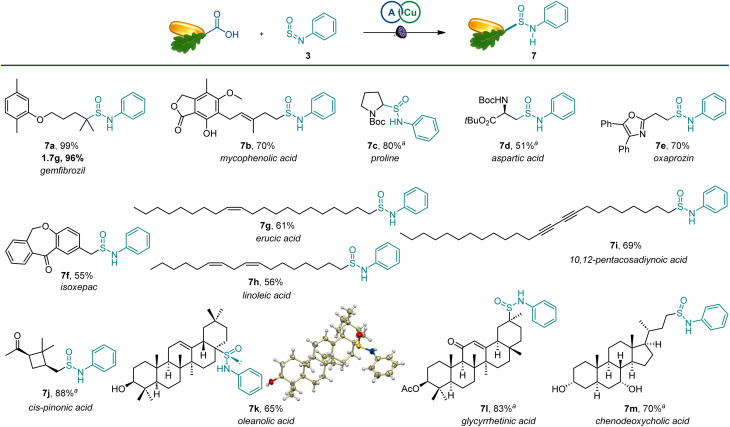
Scope of the direct decarboxylative sulfinamidation. Reaction conditions: see Scheme 1. ^*a*^1 : 1 dr.

The scope of the reaction was also examined in the more structurally complex settings of active pharmaceutical ingredients and natural products (Scheme 4). The carboxylic groups in the lipid regulator gemfibrozil and immunosuppressant mycophenolic acid were readily exchanged with sulfinamides (7a and 7b), and the reaction performed equally well on a gram scale. Similarly, bioisosteric analogues of amino acids could be readily produced from proline and aspartic acid derivatives (7c,d). Nonsteroidal anti-inflammatory drugs oxaprozin and isoxepac also afforded corresponding sulfinamides (7e,f). Furthermore, olefinic and acetylenic moieties in unsaturated fatty acids were well-tolerated (7g–7i). The four-membered ring remained intact in *cis*-pinonic acid (7j), pointing to an efficient alkyl radical trapping by sulfinylamines. Interestingly, oleanolic acid produced sulfinamide 7k as a single diastereomer, indicating that significant stereocontrol can be achieved in sterically constrained molecular settings. Additionally, the reactive enone group in glycyrrhetinic acid (7l) and unprotected hydroxy groups in chenodeoxycholic acid (7m) were well-tolerated.

**Fig. 3:**
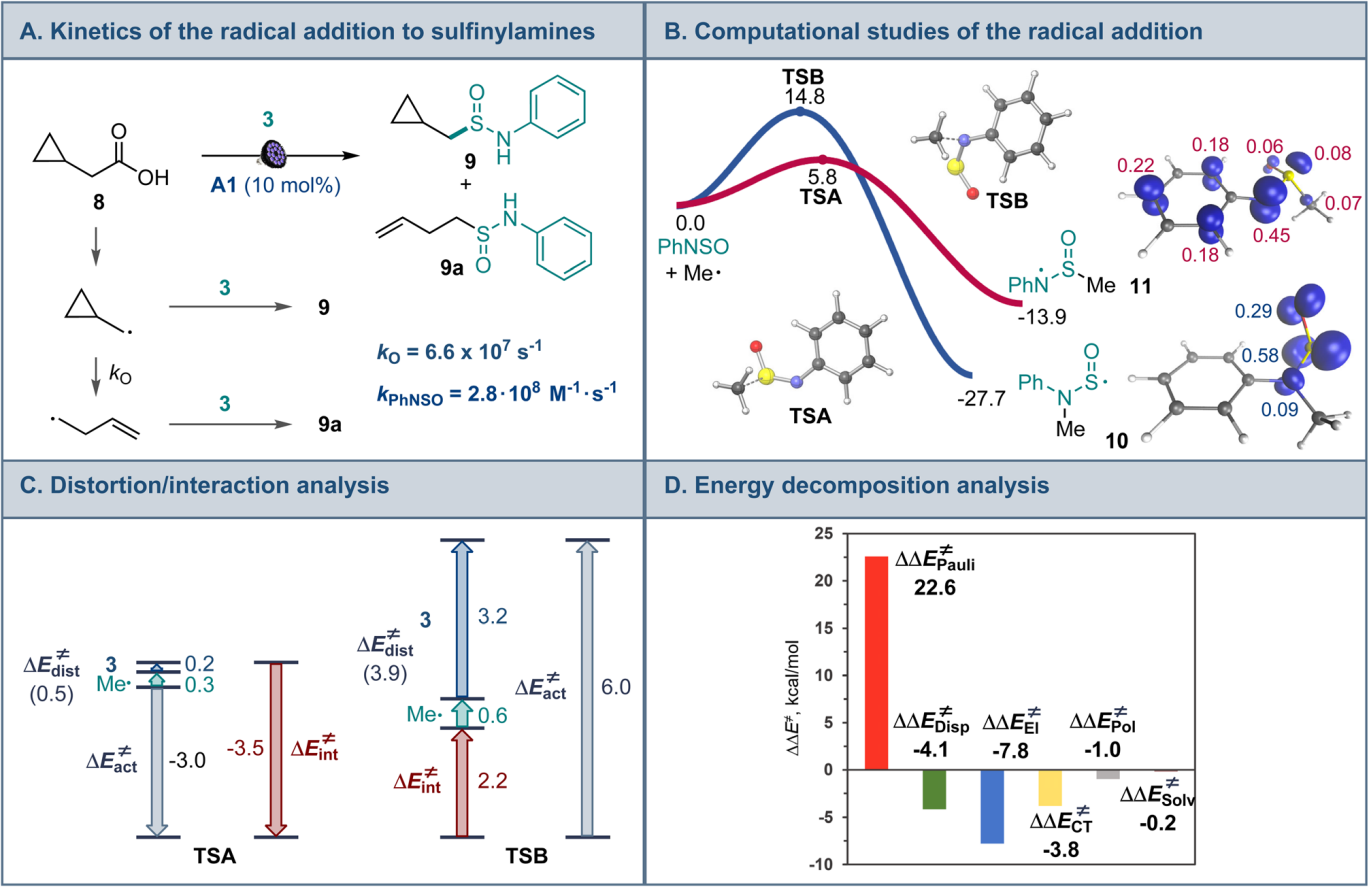
(A) Radical clock studies for the radical addition to sulfinylamines. (B) Computational studies of the radical addition reaction, Δ*G*, kcal mol^−1^, and the spin density isosurface (isovalue = 0.03) for intermediates 10 and 11. (C) Distortion/interaction activation strain model analysis of TSA and TSB. (D) Energy decomposition analysis for TSA and TSB, ΔΔ*E*≠ = ΔE_TSB_≠ − ΔE_TSA_≠, kcal mol^−1^.

Mechanistic and computational studies were carried out to gain insight into the reactivity of sulfinylamines in the decarboxylative reaction with carboxylic acids (Fig. 3A). Radical clock studies with acid 8 producing sulfinamides 9 and 9a revealed that the reaction between the alkyl radical and sulfinylamine 3 proceeds with a rate constant of 2.8 × 10^8^ M^−1^ s^−1^, corroborating the observed high reactivity of sulfinylamines towards alkyl radicals.

Interestingly, computational studies indicated that the alkyl radical addition to the nitrogen atom of sulfinylamine 3 en route to aminosulfinyl intermediate 10 is more thermodynamically favorable than the addition to the sulfur atom that produces sulfinamidyl radical 11 (Fig. 3B), in line with the downward trend in the strengths of C–N and C–S bonds (*cf.*, bond dissociation energies, 85.7 kcal mol^−1^ for C–N and 77.2 kcal mol^−1^ for C–S).^18^ However, the radical addition to the sulfur center was substantially more kinetically favorable, in congruence with the experimental observations. Distortion/interaction activation strain model (ASM) analysis^19^ indicates that the kinetic preference for the S-addition is due to the significantly higher distortion in the sulfinylamine fragment in the N-addition TSB that is required to accommodate the radical attack at the more hindered nitrogen atom, as well as the destabilizing interaction between the fragments in TSB. By contrast, smaller distortion in the sulfinylamine fragment and a stronger stabilizing interfragment interaction is observed in TSA. Furthermore, energy decomposition analysis (EDA)^20^ points to substantially higher Pauli (steric) repulsion in TSB that is not compensated by more favorable electrostatic, charge transfer, polarization, and dispersion interactions (Fig. 3C). Taken together, the studies suggest that the lower steric repulsion at the less encumbered sulfur center minimizes distortion and improves interfragment interaction in transition state TSA, diverting the radical addition to sulfinamidyl radical 11 from the thermodynamically more favorable aminosulfinyl radical 10 and leading to the formation of experimentally observed sulfinamide products.

**Fig. 4:**
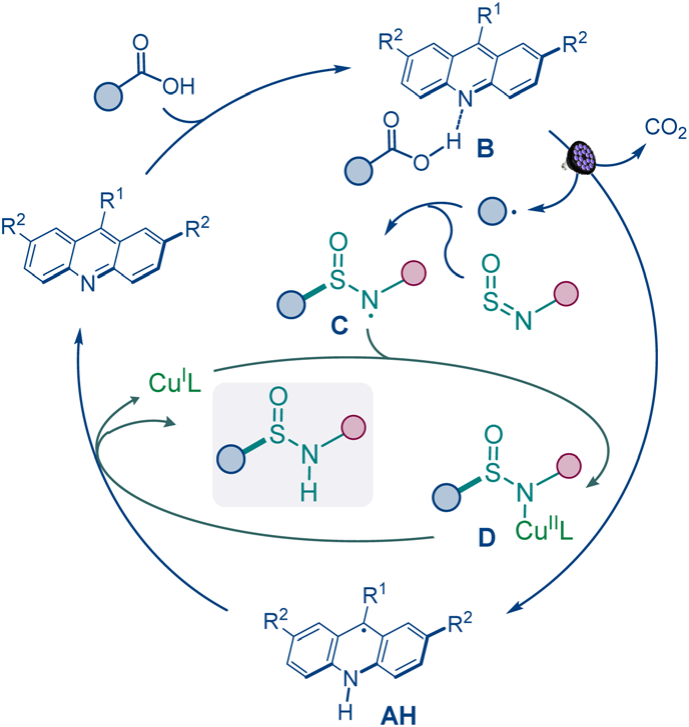
The catalytic manifold for the direct decarboxylative sulfinamidation.

Based on the experimental and computational data and previous studies of the acridine photocatalytic system,^15*a*–*e*^ the mechanistic manifold involves formation of the alkyl radical in the acridine (A) catalytic cycle *via* a photoinduced proton-coupled electron transfer (PCET) in the hydrogen bond complex B (Fig. 4).^15*d*^ The radical is intercepted by the sulfinamide, producing aminosulfinyl radical C that is stabilized in a complex with the copper catalyst (C). Subsequent PCET with acridinyl radical AH releases the sulfinamide product and the copper catalyst.^21^

Development of predictive models for reaction efficiency and selectivity can facilitate implementation of new synthetic methodologies, reduce experimental optimization, and improve synthetic planning, however, the recent work has largely been confined to the development of ML models for two-electron processes.^22^ Given the simplicity of the optimized catalytic system with the acid and sulfinylamine as the only variable components, we questioned if a predictive model for the efficiency of the decarboxylative sulfonamidation can be developed using machine learning tools in conjunction with computationally-derived descriptors of the underlying radical reactivity.

**Fig. 5:**
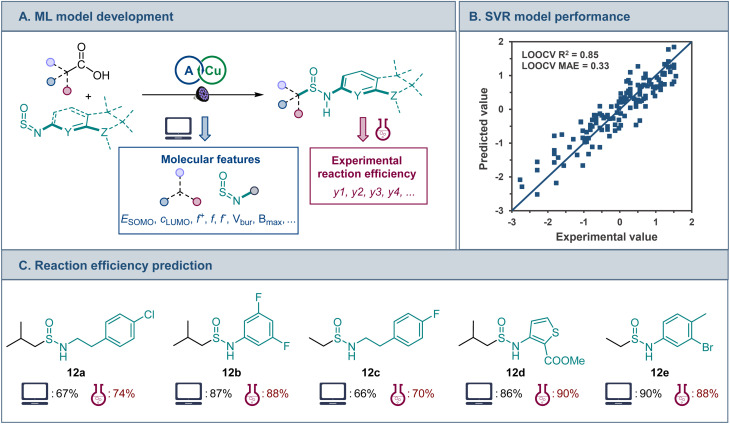
(A) ML model development for the direct decarboxylative sulfinamidation. (B) Predictive performance of the developed SVR model. (C) Comparison of the experimental and predicted efficiencies for an external set of structurally distinct carboxylic acids and sulfinylamines, isolated yields.

A range of features that include frontier MO coefficients and energies, Fukui indices, and steric (buried volume and Sterimol) parameters were generated computationally for the radical center in the alkyl fragments of the acids and the N=S=O group of sulfinylamines (Fig. 5A). Predictive performance of models that are based on various ML algorithms was then evaluated using experimental data for the reaction efficiency. The Support Vector Regression (SVR) model demonstrated the best predictive performance (Fig. 5B) in leave-one-out cross validation (LOOCV, mean absolute error, MAE = 0.33 (6.2%)). Furthermore, feature importance analysis pointed to an interplay of steric and electronic factors in the reactivity of the radical addition partners (Fig. S3†). For sulfinylamine, Sterimol parameters *B*_min_ and *B*_max_ for the O atom and *B*_max_ for the N atom, as well as HOMO-1, LUMO, and LUMO+1 energies were the most important features. On the other hand, HOMO-1 and SOMO energies, as well as the nucleophilic Fukui index and buried volume of the alkyl radical were the most important features. The model was further challenged with structurally distinct carboxylic acids and sulfinylamines that were not present in the training set, and a good predictive performance was observed (12a–e, Fig. 5C), pointing to the potential of the developed ML model to facilitate synthetic planning and implementation of the sulfinamidation reaction, and underscoring the potential of ML approaches based on computationally derived features for modeling the reactivity of one-electron processes.

## Conclusions

In conclusion, we have developed a direct conversion of carboxylic acids to sulfinamides enabled by acridine photocatalysis. The previously unknown transformation merges the broad chemical space of two of the most common functionalities, carboxylic acids and amines, facilitated by a one-electron process involving readily available sulfinylamines. The scope and functional group tolerance of the reaction were demonstrated with an array of functionalized coupling partners and tested in the structurally complex settings of natural products and medicinally relevant compounds. The positional selectivity of the radical addition to sulfinylamines is determined by the kinetic preference for the addition to the sulfur atom, despite the thermodynamic preference for the addition to the nitrogen atom. Furthermore, the radical reactivity can be captured with a set of computationally derived descriptors that enable a successful development of a predictive machine learning model for the sulfinamidation reaction efficiency that may facilitate synthetic planning and implementation of the new transformation.

## Data availability

All experimental procedures, characterization data, NMR spectra for all new compounds, and details of the computational studies can be found in the ESI.†

## Author contributions

HTD, AP, SN, WTT, and SKD carried out the experiments, and RT, PML, WBH, and SOF performed the computational studies. HDA performed the X-ray crystallography studies. OVL conceived the project, wrote the manuscript, and co-wrote the ESI.† HTD, AP, SN, SKD, and RT co-wrote the ESI† and contributed to writing the manuscript.

## Conflicts of interest

There are no conflicts to declare.

## Supplementary Material

SC-014-D3SC04727J-s001

SC-014-D3SC04727J-s002
