# Supplementary material for: Kinetically-driven reactivity of sulfinylamines enables direct conversion of carboxylic acids to sulfinamides
Source: Chem Sci. 2023 Oct 9;14(46):13384–91. doi: 10.1039/d3sc04727j (PMC10685282; doi:10.1039/d3sc04727j)
Supplement: SC-014-D3SC04727J-s001 [file SC-014-D3SC04727J-s001.pdf]

Electronic Supplementary Material (ESI) for Chemical Science.
This journal is © The Royal Society of Chemistry 2023

Supporting Information

Kinetically-Driven Reactivity of Sulfinylamines Enables Direct Conversion of Carboxylic Acids to Sulfinamides

Hang T. Dang, Arka Porey, Sachchida Nand, Ramon Trevino, Patrick Manning-Lorino, William B. Hughes, Seth Fremin, William Thompson, Shree Krishna Dhakal, Hadi D. Arman, and Oleg V. Larionov*

Department of Chemistry, The University of Texas at San Antonio, San Antonio, Texas 78249, United States
oleg.larionov@utsa.edu

Contents

Materials and experimental details S1
General procedures S2
Additional experimental studies S3
Table S1 .... S3
Figure S1 .... S4
Sulfinamide products S4
Computational data S41
X-ray crystallographic data S64
NMR spectroscopic data S70
References S184

Materials and experimental details

Materials: Anhydrous dichloromethane were collected under argon from an LC Technologies solvent purification system, having been passed through two columns packed with molecular sieves.
Experimental equipment: The photoinduced reactions were conducted in borosilicate glass test-tubes (8 mL capacity, Duran) fitted with GL14 screwcaps placed in a test-tube rack on a magnetic stirplate that was flanked by two 36W LED lights (λ_{max} = 400 nm, 2.6 mW/cm^2). The temperature in the test-tube rack was maintained at 25–27 °C with an air flow from a compressed air line. Eight parallel reactions arranged in two rows of four tubes were typically carried out in one test-tube rack.

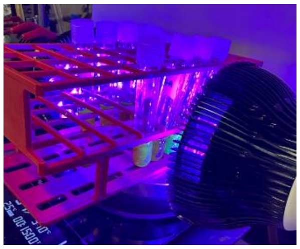

Purification: Column chromatography was performed using CombiFlash Rf-200 (Teledyne-Isco) automated flash chromatography system, as well as manually. Thin layer chromatography was carried out on silica gel-coated glass plates (Merck Kieselgel 60 F254). Plates were visualized under ultraviolet light (254 nm) and using a potassium permanganate stain.

Characterization: ^{1}H, ^{13}C and ^{19}F NMR spectra were recorded at 500 MHz (^{1}H), 125 MHz (^{13}C) and 470.5 MHz (^{19}F) on Bruker AVANCE III 500 instruments in $CDCl_3$ or other specified deuterated solvents with and without tetramethylsilane (TMS) as an internal standard at 25 °C, unless specified otherwise. Chemical shifts (δ) are reported in parts per million (ppm) from tetramethylsilane (^{1}H and ^{13}C) and $CFCl_3$ (^{19}F). Coupling constants (*J*) are in Hz. Proton multiplicity is assigned using the following abbreviations: singlet (s), doublet (d), triplet (t), quartet (q), quintet (quint.), septet (sept.), heptet (hept.), multiplet (m), broad (br).

Infrared measurements were carried out neat on a Bruker Vector 22 FT-IR spectrometer fitted with a Specac diamond attenuated total reflectance (ATR) module.

General procedure for the conversion of amines to sulfinylamines (GP1)

To an oven dried pressure tube equipped with a magnetic stir bar were added amine (1.0 eq) and dry benzene (1M) under positive flow of argon. The reaction mixture was cooled to 0 °C and thionyl chloride (1.3 equiv.) was added dropwise to the reaction mixture. The reaction mixture was allowed to warm to room temperature and then heated to 90 °C for 4 h. The resultant solid was filtered through a pad of anhydrous sodium sulfate and washed with diethyl ether. The filtrate was concentrated under reduced pressure to afford corresponding sulfinylamine that was used for the reactions with carboxylic acids without further purification.

General procedure for photoinduced decarboxylative conversion of carboxylic acids to sulfonamides (GP2)

To a 8 mL test-tube equipped with a stir bar, acid (0.2 mmol), sulfinylamine (56 mg, 0.4 mmol, 2 equiv.), acridine **A1** (5.8 mg, 0.03 mmol, 10 mol%), $Cu(MeCN)_4BF_4$ (1.9–2.5 mg, 0.006–0.008 mmol, 3–4 mol%), dtbpy (2.1–2.7 mg, 0.008–0.01 mmol, 4–5 mol%) and a mixture of DCM and MeCN (2 : 1, v/v, 2 mL). Argon was passed on the surface of the reaction for 10 seconds. The test-tube was capped and the reaction mixture was irradiated with LED light (λ = 400 nm) while stirring at 25–27 °C for 16 h. For work-up, a saturated solution of EDTA (1.5 mL) was added, followed by DCM (5 mL). The reaction mixture was then extracted with DCM (3 × 5 mL). The organic phases were combined, dried over anhydrous Na_2SO_4, concentrated under reduced pressure and the remaining material was purified by flash chromatography on silica gel to give the product.

Additional experimental and computational studies

Table S1. Catalyst Performance in the Acridine-Catalyzed Direct Decarboxylative Sulfinamide Construction.[a]

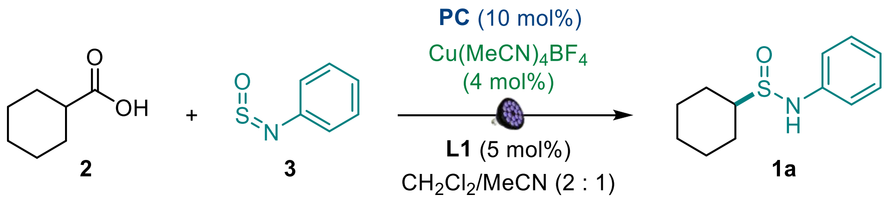

Entry	Photocatalyst	Yield, %
1	Eosin Y at 450 nm	0
2	Eosin Y at 420 nm	0
3	Eosin Y at 400 nm	0
4	Eosin Y disodium salt at 450 nm	0
5	4CzIPN at 450 nm	0
6	4CzIPN at 420 nm	0
7	4CzIPN at 400 nm	0
8	$[Acr\text{-}Mes]^+(BF_4)^-$ at 400 nm	0
9	$[Acr\text{-}Mes]^+(BF_4)^-$ at 450 nm	0[b]
10	$Ir(ppy)_3$ at 450 nm	0[b]
11	$Ir(ppy)_2(pq)$ at 450 nm	0[b]
12	$(Ir[dF(CF_3)ppy]_2(dtbpy))PF_6$ at 450 nm	0[b]
13	$Ru(bpm)_2Cl_2$ at 450 nm	0[b]
14	Ru(*p*-CF_3-bpy)$_3$(BF_4)$_2$ at 450 nm	0[b]
15	TiO_2, anatase	0[c]

[a] Reaction conditions: carboxylic acid **2** (0.2 mmol), sulfinylamine 3 prepared from aniline according the GP1 (0.33 mmol), **A1** (10 mol%), $Cu(MeCN)_4BF_4$ (4 mol%), **L1** (5 mol%), CH_2Cl_2/MeCN (2 : 1, 2 mL), LED light (400 nm), 16 h. Yield was determined by ^1H NMR spectroscopy with 1,4-dimethoxybenzene as an internal standard. [b] 2 mol% photocatalyst was used. [c] nanopowder, <25 nm particle size, 30 mg. 4CzIPN: 1,2,3,5-Tetrakis-(carbazol-9-yl)-4,6-dicyanobenzene, $[Acr\text{-}Mes]^+(BF_4)^-$: 10-Phenyl-9-(2,4,6-trimethylphenyl)acridinium tetrafluoro-borate, $Ir(ppy)_3$: Tris(2-phenylpyridine)iridium(III), $Ir(ppy)_2(pq)$: bis(2-phenylpyridine)(2-phenyl-qui-noline)iridium(III), $(Ir[dF(CF_3)ppy]_2(dtbpy))PF_6$: [4,4′-Bis(1,1-dimethylethyl)-2,2′-bipyridine-*N1*,*N1′*]-bis[3,5-difluoro-2-[5-(trifluoromethyl)-2-pyridinyl-*N*]phenyl-*C*]Iridium(III) hexafluorophosphate, $Ru(bpm)_2Cl_2$: Tris(2,2′-bipyrimide)-ruthenium(II) dichloride, Ru(*p*-CF_3-bpy)$_3$(BF_4)$_2$: Tris(2,2′-(*p*CF_3)bi-pyridine)ruthenium(II) tetrafluoroborate.

Kinetic studies of the reaction of acid 8 with sulfinylamine 3

The reaction with acid **8** (c = 0.1M) and sulfinylamine 3 prepared from aniline according to GP1 (2, 2.5, 2.75, 3, 3.25, and 3.75 equiv.) was conducted for 8 h as described in GP2. The ratio of products **9** and **9a** was obtained by ^{1}H NMR with 1,3,5-trimethoxybenzene as an internal standard. The kinetic relationships described by the following equations:

$$\frac{[\mathbf{9}]}{[\mathbf{9a}]} = \frac{k_{\mathbf{PhNSO}} \times [R\cdot] \times [\mathbf{3}]}{k_o \times [R\cdot]}$$

$$\frac{[\mathbf{9}]}{[\mathbf{9a}]} = \frac{[\mathbf{3}]}{k_{\mathbf{8a}}} \times k_{\mathbf{PhNSO}}$$

and ko = 6.6 x 10^7 s^{-1} [1] allow the calculation of the rate constant for the alkyl radical addition to sulfinylamine **3**: $k_{PhNSO} = 2.8 \cdot 10^8\ M^{-1}\cdot s^{-1}$.

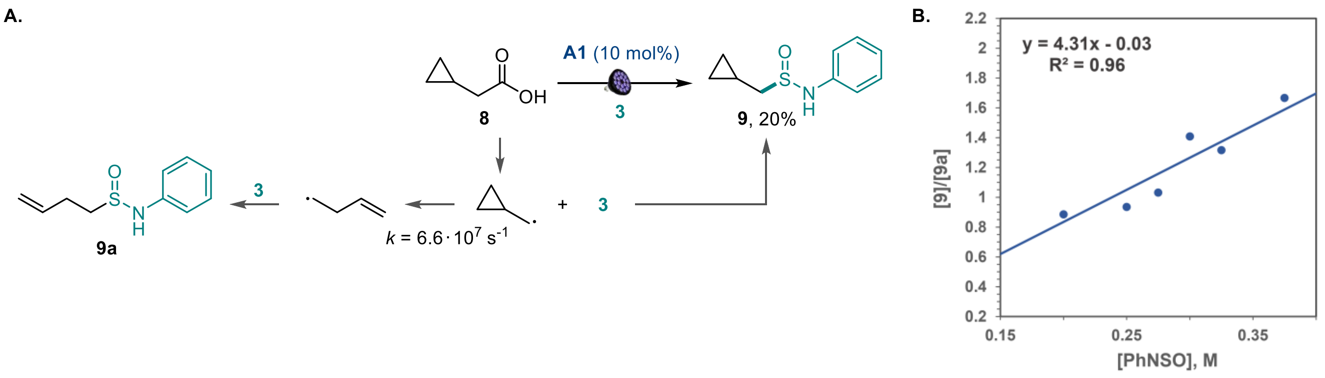

Figure S1. Kinetic studies of the reaction of acid **8** with sulfinylamine **3**. **A.** Reaction pathway for the formation of sulfinamides **9** and **9a**. **B.** Kinetic dependence of the ratio of products **9** and **9a** on the concentration of sulfinylamine **3**.

Sulfinamide products

N-Phenylcyclohexanesulfinamide (1a)[2]

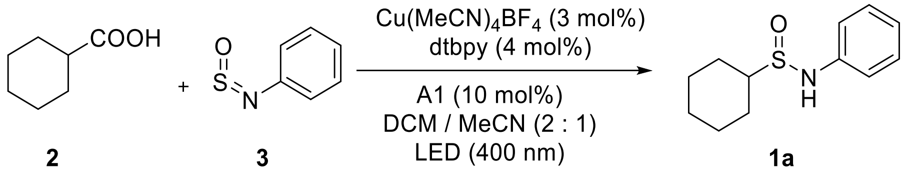

According to GP2, the reaction was carried out with cyclohexanecarboxylic acid (26 mg, 0.2 mmol), sulfinylamine **3** prepared from aniline according the GP1 (70 mg, 0.5 mmol, 2.5 equiv.), acridine **A1** (5.8 mg, 0.03 mmol, 10 mol%), $Cu(MeCN)_4BF_4$ (1.9 mg, 0.006 mmol, 3 mol%), dtbpy (2.2 mg, 0.008 mmol, 4 mol%) and DCM : MeCN (2 : 1, v/v, 2 mL). Argon was passed on the surface of the reaction for 10 seconds. The test-tube was capped and the reaction mixture was irradiated with LED light (λ = 400 nm) while stirring at 25–27 °C for 16 h. For work-up, a saturated solution of EDTA (1.5 mL) was added, followed by DCM (5 mL). The reaction mixture was then extracted with DCM (3 × 5 mL). The organic phases were combined, dried over anhydrous Na_2SO_4, concentrated under reduced pressure and the

remaining material was purified by flash chromatography on silica gel (EtOAc/hexane, 7 : 3 v/v) to give product **1a** (42 mg, 95%) as a colorless liquid.

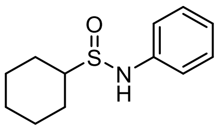

^{1}H NMR (500 MHz, CDCl3) δ 7.25 (2 H, t, J = 7.3 Hz), 7.04 (2 H, d, J = 7.9 Hz), 7.01 (1 H, t, J = 7.3 Hz), 2.88 (1 H, tt, J = 11.3, 3.8 Hz), 2.21–2.02 (2 H, m), 1.97–1.81 (2 H, m), 1.75–1.65 (1 H, m), 1.61–1.20 (6 H, m) ppm. – ^{13}C NMR (125 MHz, $CDCl_3$): 141.9, 129.43, 129.38, 122.8, 118.2, 118.1, 62.9, 26.4, 26.2, 25.5, 25.2, 25.0 ppm. – IR: 1602, 1498, 1446, 1032, 1028, 880. 742, 692 cm^{-1}.

N-Phenylnonane-1-sulfinamide (1b)

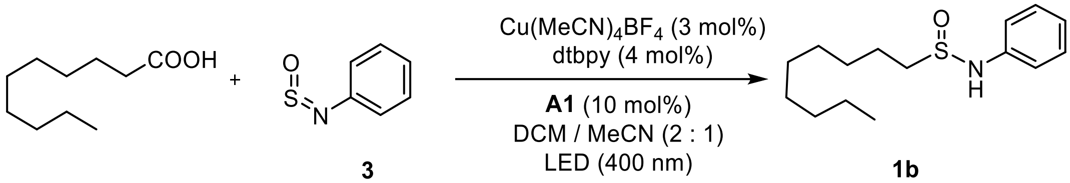

According to GP2, the reaction was carried out with decanoic acid (34 mg, 0.2 mmol), sulfinylamine **3** prepared from aniline according the GP1 (56 mg, 0.4 mmol, 2 equiv.), acridine **A1** (5.8 mg, 0.03 mmol, 10 mol%), $Cu(MeCN)_4BF_4$ (1.9 mg, 0.006 mmol, 3 mol%), dtbpy (2.2 mg, 0.008 mmol, 4 mol%) and DCM : MeCN (2 : 1, v/v, 2 mL). Argon was passed on the surface of the reaction for 10 seconds. The test-tube was capped and the reaction mixture was irradiated with LED light (λ = 400 nm) while stirring at 25–27 °C for 16 h. For work-up, a saturated solution of EDTA (1.5 mL) was added, followed by DCM (5 mL). The reaction mixture was then extracted with DCM (3 × 5 mL). The organic phases were combined, dried over anhydrous Na_2SO_4, concentrated under reduced pressure and the remaining material was purified by flash chromatography on silica gel (EtOAc/hexane, 3 : 7 v/v) to give product **1b** (49 mg, 92%) as a colorless solid.

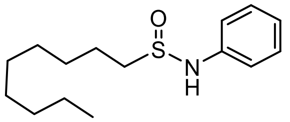

M.p.: 68–70 °C. – ^{1}H NMR (500 MHz, $CDCl_3$): 7.30–7.18 (3 H, m), 7.05 (2 H, d, *J* = 8.0 Hz), 7.01 (1 H, t, *J* = 7.4 Hz), 3.07–2.97 (2 H, m), 1.73 (2 H, p, *J* = 7.6 Hz), 1.51–1.20 (12 H, m), 0.91 (3 H, t, *J* = 6.8 Hz) ppm. – ^{13}C NMR (125 MHz, $CDCl_3$): 141.5, 129.4, 122.8, 118.1, 55.9, 31.8, 29.3, 29.24, 29.20, 28.6, 23.4, 22.7, 14.1 ppm. – IR: 3168, 2923, 2853, 1600, 1497, 1465, 1283, 1145, 1039, 889 cm^{-1}. – HRMS: calcd for $C_{15}H_{25}NOS$: 268.1730, found 268.1727 [M+H^{+}].

1-Cyclopentyl-*N*-phenylmethanesulfinamide (1c)

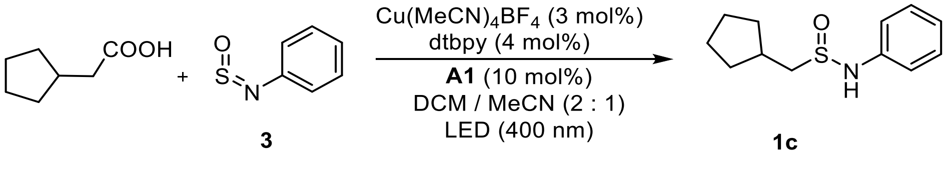

According to GP2, the reaction was carried out with 2-cyclopentylacetic acid (26 mg, 0.2 mmol), sulfinylamine 3 prepared from aniline according the GP1 (56 mg, 0.4 mmol, 2 equiv.), acridine **A1** (5.8 mg, 0.03 mmol, 10 mol%), $Cu(MeCN)_4BF_4$ (1.9 mg, 0.006 mmol, 3 mol%), dtbpy (2.2 mg, 0.008 mmol, 4 mol%) and DCM : MeCN (2 : 1, v/v, 2 mL). Argon was passed on the surface of the reaction for 10 seconds. The test-tube was capped and the reaction mixture was irradiated with LED light (λ = 400 nm) while stirring at 25–27 °C for 16 h. For work-up, a saturated solution of EDTA (1.5 mL) was added, followed by DCM (5 mL). The reaction mixture was then extracted with DCM (3 × 5 mL). The organic phases were combined, dried over anhydrous Na_2SO_4, concentrated under reduced pressure and the remaining material was purified by flash chromatography on silica gel (EtOAc/hexane, 7 : 3 v/v) to give product **1c** (44 mg, 98%) as a colorless solid.

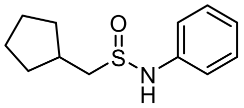

M.p.: 48–50 °C. – ^1H NMR (500 MHz, $CDCl_3$): 7.44 (1 H, s), 7.24 (2 H, t, J = 7.7 Hz), 7.05 (2 H, d, J = 8.0 Hz), 7.00 (1 H, t, J = 7.4 Hz), 3.10 (1 H, dd, J = 12.8, 6.8 Hz), 3.02 (1 H, dd, J = 12.8, 8.2 Hz), 2.25 (1 H, hept, J = 7.9 Hz), 2.06–1.91 (1 H, m), 1.89–1.78 (1 H, m), 1.75–1.52 (4 H, m), 1.38–1.20 (3 H, m) ppm. – ^{13}C NMR (125 MHz, $CDCl_3$): 141.6, 129.4, 122.7, 118.0, 62.0, 35.2, 32.5, 32.1, 24.94, 24.87 ppm. – IR: 3156, 2953, 2868, 1600, 1496, 1451, 1404, 1283, 1225, 1149, 1035, 888 cm^{-1}. – HRMS: calcd for $C_{12}H_{17}NOS$: 224.1104, found 224.1102 $[M+H^+]$.

5-Chloro-*N*-phenylpentane-1-sulfinamide (1d)

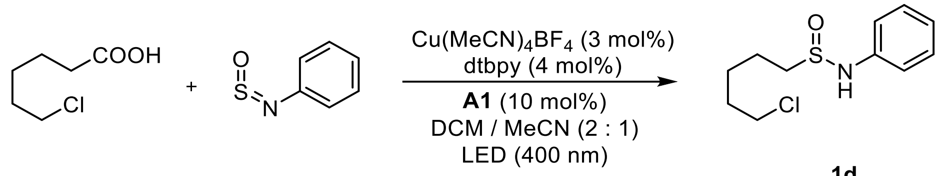

According to GP2, the reaction was carried out with 6-chlorohexanoic acid (30 mg, 0.2 mmol), sulfinylamine 3 prepared from aniline according the GP1 (56 mg, 0.4 mmol, 2 equiv.), acridine **A1** (5.8 mg, 0.03 mmol, 10 mol%), $Cu(MeCN)_4BF_4$ (1.9 mg, 0.006 mmol, 3 mol%), dtbpy (2.2 mg, 0.008 mmol, 4 mol%) and DCM : MeCN (2 : 1, v/v, 2 mL). Argon was passed on the surface of the reaction for 10 seconds. The test-tube was capped and the reaction mixture was irradiated with LED light (λ = 400 nm) while stirring at 25–27 °C for 16 h. For work-up, a saturated solution of EDTA (1.5 mL) was added, followed by DCM (5 mL). The reaction mixture was then extracted with DCM (3 × 5 mL). The organic phases were combined, dried over anhydrous Na_2SO_4, concentrated under reduced pressure and the remaining material was purified by flash chromatography on silica gel (EtOAc/hexane, 7 : 3 v/v) to give product **1d** (48 mg, 98%) as a colorless solid.

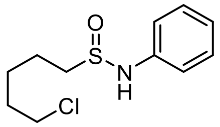

M.p.: 46–48 °C. – ^1H NMR (500 MHz, $CDCl_3$): 7.30–7.23 (3 H, m), 7.09–7.00 (3 H, m), 3.53 (2 H, t, J = 6.5 Hz), 3.02 (2 H, td, J = 7.5, 3.7 Hz), 1.85–1.71 (4 H, m), 1.65–1.48 (2 H, m) ppm. – ^{13}C NMR (125 MHz, $CDCl_3$): 141.3, 129.5, 123.0, 118.2, 55.5, 44.5, 32.1,

25.9, 22.8 ppm. – IR: 3152, 2920, 2851, 2634, 1599, 1496, 1461, 1405, 1342, 1283, 1148, 1029, 887 cm^{-1}. – HRMS: calcd for $C_{11}H_{16}ClNOS$: 246.0714, found 246.0712 [M+H$^+$].

5-Bromo-*N*-phenylpentane-1-sulfinamide (1e)

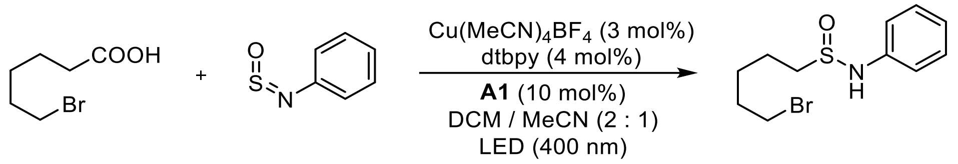

3 1e

According to GP2, the reaction was carried out with 6-bromohexanoic acid (29 mg, 0.2 mmol), sulfinylamine 3 prepared from aniline according the GP1 (56 mg, 0.4 mmol, 2 equiv.), acridine **A1** (5.8 mg, 0.03 mmol, 10 mol%), $Cu(MeCN)_4BF_4$ (1.9 mg, 0.006 mmol, 3 mol%), dtbpy (2.2 mg, 0.008 mmol, 4 mol%) and DCM : MeCN (2 : 1, v/v, 2 mL). Argon was passed on the surface of the reaction for 10 seconds. The test-tube was capped and the reaction mixture was irradiated with LED light (λ = 400 nm) while stirring at 25–27 °C for 16 h. For work-up, a saturated solution of EDTA (1.5 mL) was added, followed by DCM (5 mL). The reaction mixture was then extracted with DCM (3 × 5 mL). The organic phases were combined, dried over anhydrous Na_2SO_4, concentrated under reduced pressure and the remaining material was purified by flash chromatography on silica gel (EtOAc/hexane, 7 : 3 v/v) to give product **1e** (44 mg, 76%) as a colorless liquid.

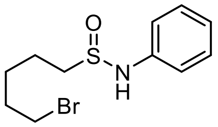

^{1}H NMR (500 MHz, $CDCl_3$): 7.31–7.22 (3 H, m), 7.05 (2 H, d, *J* = 7.7 Hz), 7.02 (1 H, t, *J* = 7.4 Hz), 3.40 (2 H, t, *J* = 6.6 Hz), 3.10–2.95 (2 H, m), 1.87 (2 H, p, *J* = 7.1 Hz), 1.76 (2 H, p, *J* = 7.7 Hz), 1.68–1.45 (2 H, m) ppm. – ^{13}C NMR (125 MHz, $CDCl_3$): 141.3, 129.5, 123.1, 118.2, 55.5, 33.2, 32.2, 27.1, 22.6 ppm. – IR: 2925, 2858, 2587, 1714, 1598, 1494, 1462, 1300, 1261, 1144, 1031 cm^{-1}. – HRMS: calcd for $C_{11}H_{16}BrNOS$: 290.0209, found 290.0206[M+H$^+$].

2-(4-Fluorophenyl)-*N*-phenylethane-1-sulfinamide (1f)

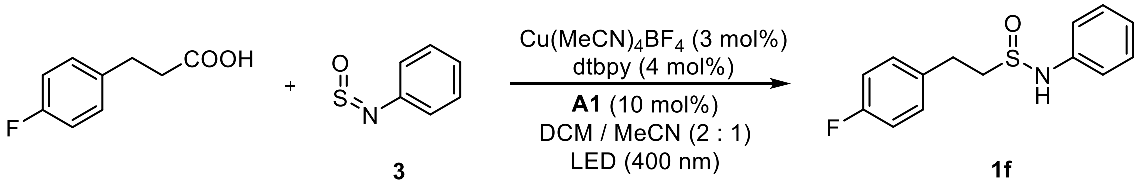

3 **1f**

According to GP2, the reaction was carried out with 3-(4-fluorophenyl)propanoic acid (33 mg, 0.2 mmol), sulfinylamine 3 prepared from aniline according the GP1 (56 mg, 0.4 mmol, 2 equiv.), acridine **A1** (5.8 mg, 0.03 mmol, 10 mol%), $Cu(MeCN)_4BF_4$ (1.9 mg, 0.006 mmol, 3 mol%), dtbpy (2.2 mg, 0.008 mmol, 4 mol%) and DCM : MeCN (2 : 1, v/v, 2 mL). Argon was passed on the surface of the reaction for 10 seconds. The test-tube was capped and the reaction mixture was irradiated with LED light (λ = 400 nm) while stirring at 25–27 °C for 16 h. For work-up, a saturated solution of EDTA (1.5 mL) was added,

followed by DCM (5 mL). The reaction mixture was then extracted with DCM (3 × 5 mL). The organic phases were combined, dried over anhydrous Na_2SO_4, concentrated under reduced pressure and the remaining material was purified by flash chromatography on silica gel (EtOAc/hexane, 7 : 3 v/v) to give product **1f** (35 mg, 67%) as a colorless solid.

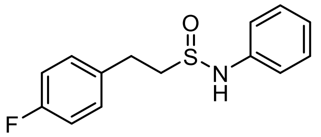

M.p.: 168–170 °C. – ^{1}H NMR (500 MHz, $CDCl_3$): 7.32–7.23 (2 H, m), 7.22–7.13 (2 H, m), 7.08–6.92 (6 H, m), 3.34–3.22 (2 H, m), 3.05 (2 H, t, *J* = 7.4 Hz) ppm. – ^{13}C NMR (125 MHz, $CDCl_3$): 161.8 (d, *J* = 245.2 Hz), 141.0, 134.1 (d, *J* = 3.4 Hz), 130.0 (d, *J* = 8.0 Hz), 129.5, 123.4, 118.5, 115.7 (d, *J* = 21.5 Hz), 57.0, 28.8 ppm. – ^{19}F NMR (471 MHz, $CDCl_3$) δ -115.9 (t, *J* = 7.7 Hz). – IR: 2918, 2651, 1599, 1508, 1495, 1416, 1315, 1220, 1153, 1126, 1090, 1037, 940 cm^{-1}. – HRMS: calcd for $C_{14}H_{14}FNOS$: 264.0853, found 264.0851[M+H$^+$].

4-Oxo-*N*,4-diphenylbutane-1-sulfinamide (1g)

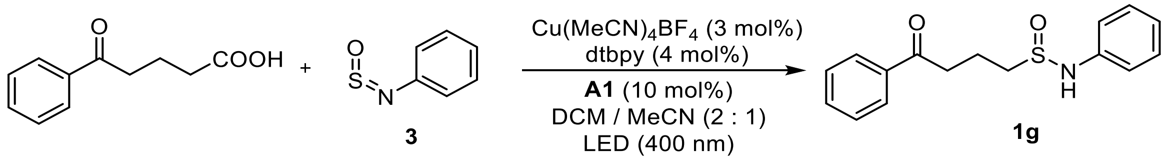

According to GP2, the reaction was carried out with 3-(((4-oxo-4-phenylbutyl)sulfinyl)amino)benzene-1-ylium (57 mg, 0.2 mmol), sulfinylamine 3 prepared from aniline according the GP1 (56 mg, 0.4 mmol, 2 equiv.), acridine **A1** (5.8 mg, 0.03 mmol, 10 mol%), $Cu(MeCN)_4BF_4$ (1.9 mg, 0.006 mmol, 3 mol%), dtbpy (2.2 mg, 0.008 mmol, 4 mol%) and DCM : MeCN (2 : 1, v/v, 2 mL). Argon was passed on the surface of the reaction for 10 seconds. The test-tube was capped and the reaction mixture was irradiated with LED light (λ = 400 nm) while stirring at 25–27 °C for 16 h. For work-up, a saturated solution of EDTA (1.5 mL) was added, followed by DCM (5 mL). The reaction mixture was then extracted with DCM (3 × 5 mL). The organic phases were combined, dried over anhydrous Na_2SO_4, concentrated under reduced pressure and the remaining material was purified by flash chromatography on silica gel (EtOAc/hexane, 8 : 2 v/v) to give product **1g** (37 mg, 65%) as a colorless solid.

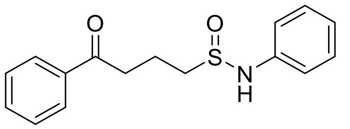

M.p.: 110–112 °C. – ^{1}H NMR (500 MHz, $CDCl_3$): 7.95 (2 H, d, *J* = 1.5 Hz), 7.66–7.55 (1 H, m), 7.48 (2 H, t, *J* = 7.7 Hz), 7.26 (2 H, t, *J* = 7.9 Hz), 7.16–7.06 (3 H, m), 7.02 (1 H, t, *J* = 7.5 Hz), 3.31–3.02 (4 H, m), 2.23 (2 H, p, *J* = 7.2 Hz) ppm. – ^{13}C NMR (125 MHz, $CDCl_3$): 198.7, 141.2, 136.5, 133.4, 129.5, 128.7, 128.0, 123.2, 118.4, 54.8, 36.7, 17.8 ppm. – IR: 3361, 3057, 2923, 2619, 1678, 1622, 1596, 1580, 1498, 1448, 1360, 1318, 1224 cm^{-1}. – HRMS: calcd for $C_{16}H_{17}NO_2S$: 288.1053 , found 288.1051[M+H$^+$].

Methyl 5-((phenylamino)sulfinyl)pentanoate (1h)

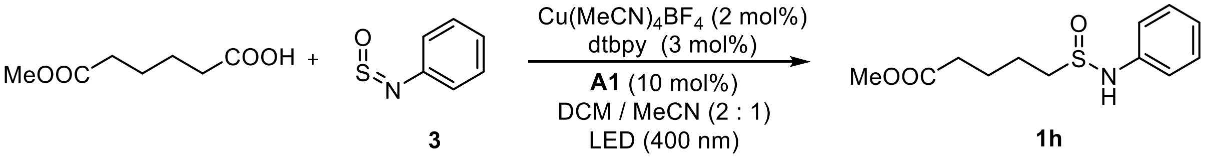

According to GP2, the reaction was carried out with 5-acetoxypentanoic acid (32 mg, 0.2 mmol), sulfinylamine 3 prepared from aniline according the GP1 (56 mg, 0.4 mmol, 2 equiv.), acridine **A1** (5.8 mg, 0.03 mmol, 10 mol%), $Cu(MeCN)_4BF_4$ (1.3 mg, 0.004 mmol, 2 mol%), dtbpy (1.6 mg, 0.006 mmol, 3 mol%) and DCM : MeCN (2 : 1, v/v, 2 mL). Argon was passed on the surface of the reaction for 10 seconds. The test-tube was capped and the reaction mixture was irradiated with LED light (λ = 400 nm) while stirring at 25–27 °C for 16 h. For work-up, a saturated solution of EDTA (1.5 mL) was added, followed by DCM (5 mL). The reaction mixture was then extracted with DCM (3 × 5 mL). The organic phases were combined, dried over anhydrous Na_2SO_4, concentrated under reduced pressure and the remaining material was purified by flash chromatography on silica gel (EtOAc/hexane, 7 : 3 v/v) to give product **1h** (36 mg, 71%) as a colorless solid.

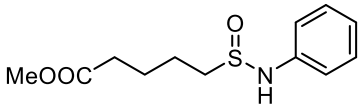

M.p.: 40–42 °C. – ^1H NMR (500 MHz, $CDCl_3$): 7.27 (2 H, t, J = 7.9 Hz), 7.08–7.00 (3 H, m), 6.97 (1 H, s), 3.69 (3 H, s), 3.01 (2 H, tt, J = 13.4, 6.9 Hz), 2.37 (2 H, d, J = 6.7 Hz), 1.84–1.68 (4 H, m, 4H) ppm. – ^{13}C NMR (125 MHz, $CDCl_3$): 173.4, 141.2, 129.5, 123.2, 118.4, 55.4, 51.7, 33.5, 23.8, 22.8 ppm. – IR: 3565, 2949, 1732, 1600, 1497, 1437, 1173, 1036, 890 cm^{-1}. – HRMS: calcd for $C_{11}H_{16}ClNOS$: 256.1002, found 256.1000 [$M+H^+$].

N-Phenyl-3-(thiophen-2-yl)propane-1-sulfinamide (1i)

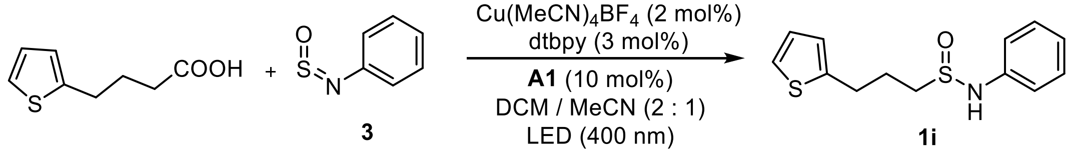

According to GP2, the reaction was carried out with 4-(thiophen-2-yl)butanoic acid (34 mg, 0.2 mmol), sulfinylamine 3 prepared from aniline according the GP1 (56 mg, 0.4 mmol, 2 equiv.), acridine **A1** (5.8 mg, 0.03 mmol, 10 mol%), $Cu(MeCN)_4BF_4$ (1.3 mg, 0.004 mmol, 2 mol%), dtbpy (1.6 mg, 0.006 mmol, 3 mol%) and DCM : MeCN (2 : 1, v/v, 2 mL). Argon was passed on the surface of the reaction for 10 seconds. The test-tube was capped and the reaction mixture was irradiated with LED light (λ = 400 nm) while stirring at 25–27 °C for 16 h. For work-up, a saturated solution of EDTA (1.5 mL) was added, followed by DCM (5 mL). The reaction mixture was then extracted with DCM (3 × 5 mL). The organic phases were combined, dried over anhydrous Na_2SO_4, concentrated under reduced pressure and the remaining material was purified by flash chromatography on silica gel (EtOAc/hexane, 7 : 3 v/v) to give product **1i** (43 mg, 79%) as a colorless solid.

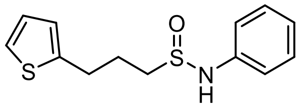

M.p.: 66–68 °C. – ^{1}H NMR (500 MHz, $CDCl_3$): 7.31–7.13 (4 H, m), 7.11–7.00 (3 H, m), 6.95 (1 H, t, J = 4.3 Hz), 6.81 (1 H, d, J = 3.4 Hz), 3.13–2.88 (4 H, m), 2.12 (2 H, p, J = 7.6 Hz) ppm. – ^{13}C NMR (125 MHz, $CDCl_3$): 143.0, 141.3, 129.5, 127.0, 125.0, 123.7, 123.1, 118.2, 54.7, 28.6, 25.4 ppm. – IR: 3152, 2922, 1599, 1496, 1439, 1405, 1319, 1283, 1227, 1149, 1038 cm^{-1}. – HRMS: calcd for $C_{13}H_{15}NOS_2$: 266.0668, found 266.0667 [M+H^{+}].

4,4-Difluoro-*N*-phenylcyclohexane-1-sulfinamide (1j)

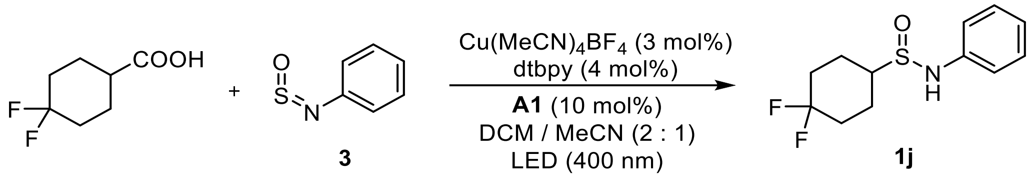

According to GP2, the reaction was carried out with 4,4-difluorocyclohexane-1-carboxylic acid (33 mg, 0.2 mmol), sulfinylamine 3 prepared from aniline according the GP1 (56 mg, 0.4 mmol, 2 equiv.), acridine **A1** (5.8 mg, 0.03 mmol, 10 mol%), $Cu(MeCN)_4BF_4$ (1.9 mg, 0.006 mmol, 3 mol%), dtbpy (2.2 mg, 0.008 mmol, 4 mol%) and DCM : MeCN (2 : 1, v/v, 2 mL). Argon was passed on the surface of the reaction for 10 seconds. The test-tube was capped and the reaction mixture was irradiated with LED light (λ = 400 nm) while stirring at 25–27 °C for 16 h. For work-up, a saturated solution of EDTA (1.5 mL) was added, followed by DCM (5 mL). The reaction mixture was then extracted with DCM (3 × 5 mL). The organic phases were combined, dried over anhydrous Na_2SO_4, concentrated under reduced pressure and the remaining material was purified by flash chromatography on silica gel (EtOAc/hexane, 4 : 6 v/v) to give product **1j** (50 mg, 96%) as a colorless solid.

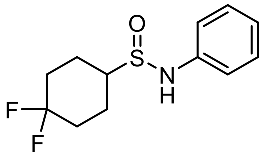

M.p.: 112–114 °C. – ^{1}H NMR (500 MHz, $CDCl_3$): 7.28 (2 H, t, J = 7.9 Hz), 7.14–7.03 (4 H, m), 3.04–2.89 (1 H, m), 2.33–2.06 (4 H, m), 2.01–1.57 (4 H, m) ppm. – ^{13}C NMR (125 MHz, $CDCl_3$): 141.3, 129.6, 123.3, 122.2 (t, J = 241.4 Hz), 118.2, 60.3, 32.2 (td, J = 24.9, 20.3 Hz), 23.0 (dd, J = 36.6, 8.5 Hz) ppm. – ^{19}F NMR (471 MHz, $CDCl_3$) δ -94.94 (d, J = 238.7 Hz), -100.34 (d, J = 238.5 Hz). – IR: 2918, 2851, 2620, 1593, 1540, 1497, 1448, 1372, 1292, 1270, 1202, 1181, 1147, 1121, 1101 cm^{-1}. – HRMS: calcd for $C_{12}H_{15}F_2NOS$: 260.0915, found 260.0912 [M+H^{+}].

N-Phenylcycloheptanesulfinamide (1k)

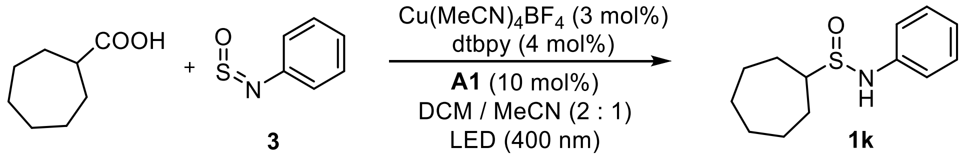

According to GP2, the reaction was carried out with cycloheptanecarboxylic acid (28 mg, 0.2 mmol), sulfinylamine 3 prepared from aniline according the GP1 (56 mg, 0.4 mmol, 2 equiv.), acridine **A1** (5.8 mg, 0.03 mmol, 10 mol%), $Cu(MeCN)_4BF_4$ (1.9 mg, 0.006 mmol, 3 mol%), dtbpy (2.2 mg, 0.008 mmol, 4

mol%) and DCM : MeCN (2 : 1, v/v, 2 mL). Argon was passed on the surface of the reaction for 10 seconds. The test-tube was capped and the reaction mixture was irradiated with LED light (λ = 400 nm) while stirring at 25–27 °C for 16 h. For work-up, a saturated solution of EDTA (1.5 mL) was added, followed by DCM (5 mL). The reaction mixture was then extracted with DCM (3 × 5 mL). The organic phases were combined, dried over anhydrous Na_2SO_4, concentrated under reduced pressure and the remaining material was purified by flash chromatography on silica gel (EtOAc/hexane, 2 : 8 v/v) to give product **1k** (48 mg, 99%) as a colorless solid.

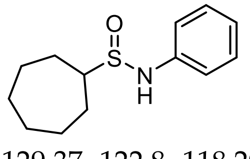

M.p.: 68–70 °C. – ^{1}H NMR (500 MHz, $CDCl_3$): 7.25 (2 H, t, *J* = 7.9 Hz), 7.09–6.99 (3 H, m), 6.44 (1 H, s), 2.96 (1 H, tt, *J* = 9.3, 4.4 Hz), 2.33–2.06 (2 H, m), 1.92–1.72 (3 H, m), 1.68–1.43 (7 H, m) ppm. – ^{13}C NMR (125 MHz, $CDCl_3$): 142.0, 129.4, 129.42, 129.37, 122.8, 118.20, 118.18, 118.1, 64.5, 28.7, 28.3, 27.6, 26.7, 26.12, 26.08 ppm. – IR: 2922, 2852, 1599, 1496, 1459, 1412, 1281, 1225, 1174, 1143, 1077, 1038, 885 cm^{-1}. – HRMS: calcd for $C_{13}H_{19}NOS$: 260.1080, found 260.1082 [M+Na$^+$].

N-Phenylcyclopent-3-ene-1-sulfinamide (1l)

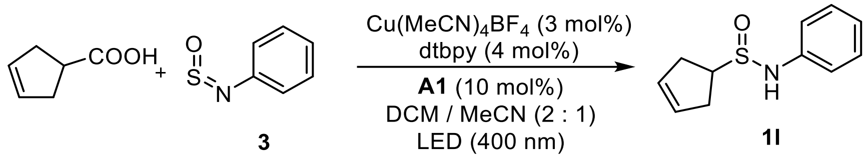

According to GP2, the reaction was carried out with cyclopent-3-ene-1-carboxylic acid (22 mg, 0.2 mmol), sulfinylamine 3 prepared from aniline according the GP1 (56 mg, 0.4 mmol, 2 equiv.), acridine **A1** (5.8 mg, 0.03 mmol, 10 mol%), $Cu(MeCN)_4BF_4$ (1.9 mg, 0.006 mmol, 3 mol%), dtbpy (2.2 mg, 0.008 mmol, 4 mol%) and DCM : MeCN (2 : 1, v/v, 2 mL). Argon was passed on the surface of the reaction for 10 seconds. The test-tube was capped and the reaction mixture was irradiated with LED light (λ = 400 nm) while stirring at 25–27 °C for 16 h. For work-up, a saturated solution of EDTA (1.5 mL) was added, followed by DCM (5 mL). The reaction mixture was then extracted with DCM (3 × 5 mL). The organic phases were combined, dried over anhydrous Na_2SO_4, concentrated under reduced pressure and the remaining material was purified by flash chromatography on silica gel (EtOAc/hexane, 6 : 4 v/v) to give product **1l** (34 mg, 82%) as a colorless liquid.

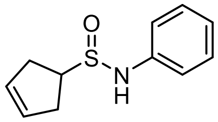

^{1}H NMR (500 MHz, $CDCl_3$): 7.30 – 7.21 (2 H, m), 7.14–6.87 (4 H, m), 5.92–5.68 (2 H, m), 3.80 (1 H, tt, *J* = 8.6, 4.1 Hz), 3.00–2.86 (1 H, m), 2.78–2.53 (3 H, m) ppm. – ^{13}C NMR (125 MHz, $CDCl_3$): 141.8, 129.4, 129.1, 122.8, 118.0, 61.9, 33.8, 33.7 ppm. – IR: 1715, 1600, 1497, 1493, 1242, 1302, 1147, 1040, 890 cm^{-1}. – HRMS: calcd for $C_{11}H_{13}NOS$: 208.0791, found 208.0789 [M+H$^+$].

N-Phenyl-2,3-dihydro-1H-indene-2-sulfinamide (1m)

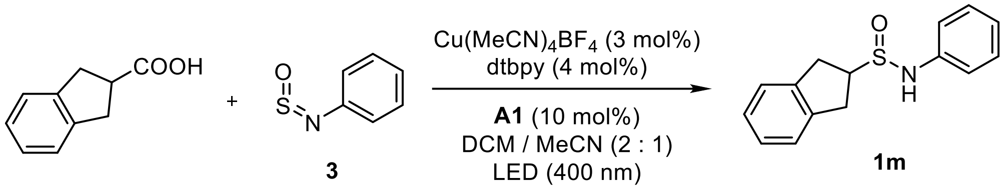

According to GP2, the reaction was carried out with 2,3-dihydro-1H-indene-2-carboxylic acid (32 mg, 0.2 mmol), sulfinylamine 3 prepared from aniline according the GP1 (56 mg, 0.4 mmol, 2 equiv.), acridine **A1** (5.8 mg, 0.03 mmol, 10 mol%), $Cu(MeCN)_4BF_4$ (1.9 mg, 0.006 mmol, 3 mol%), dtbpy (2.2 mg, 0.008 mmol, 4 mol%) and DCM : MeCN (2 : 1, v/v, 2 mL). Argon was passed on the surface of the reaction for 10 seconds. The test-tube was capped and the reaction mixture was irradiated with LED light (λ = 400 nm) while stirring at 25–27 °C for 16 h. For work-up, a saturated solution of EDTA (1.5 mL) was added, followed by DCM (5 mL). The reaction mixture was then extracted with DCM (3 × 5 mL). The organic phases were combined, dried over anhydrous Na_2SO_4, concentrated under reduced pressure and the remaining material was purified by flash chromatography on silica gel (EtOAc/hexane, 4 : 6 v/v) to give product **1m** (38 mg, 74%) as a colorless solid.

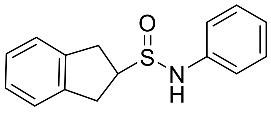

M.p.: 109–110 °C. – ^{1}H NMR (500 MHz, $CDCl_3$): 7.37–7.21 (6 H, m), 7.10–6.97 (4 H, m), 3.98 (1 H, tt, J = 8.4, 4.9 Hz), 3.53 (1 H, dd, J = 17.2, 4.7 Hz), 3.40–3.21 (3 H, m) ppm. – ^{13}C NMR (125 MHz, $CDCl_3$): 141.6, 140.4, 140.3, 129.5, 127.2, 127.1, 124.8, 124.5, 123.0, 118.2, 63.4, 33.8 ppm. – IR: 3151, 3044, 2898, 1705, 1641, 1598, 1496, 1485, 1459, 1319, 1282, 1224, 1147, 1055, 866 cm^{-1}. – HRMS: calcd for $C_{15}H_{15}NOS$: 258.0947, found 258.0946 [M+H$^+$].

N-Phenyltetrahydro-2H-pyran-4-sulfinamide (1n)[3]

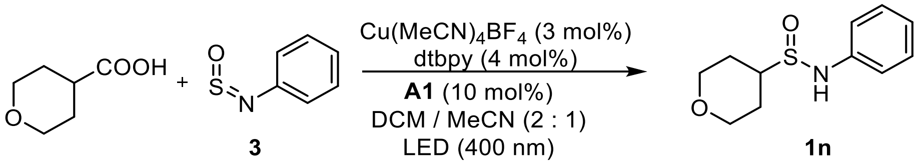

According to GP2, the reaction was carried out with tetrahydro-2H-pyran-4-carboxylic acid (26 mg, 0.2 mmol), sulfinylamine 3 prepared from aniline according the GP1 (56 mg, 0.4 mmol, 2 equiv.), acridine **A1** (5.8 mg, 0.03 mmol, 10 mol%), $Cu(MeCN)_4BF_4$ (1.9 mg, 0.006 mmol, 3 mol%), dtbpy (2.2 mg, 0.008 mmol, 4 mol%) and DCM : MeCN (2 : 1, v/v, 2 mL). Argon was passed on the surface of the reaction for 10 seconds. The test-tube was capped and the reaction mixture was irradiated with LED light (λ = 400 nm) while stirring at 25–27 °C for 16 h. For work-up, a saturated solution of EDTA (1.5 mL) was added, followed by DCM (5 mL). The reaction mixture was then extracted with DCM (3 × 5 mL). The organic phases were combined, dried over anhydrous Na_2SO_4, concentrated under reduced pressure and the remaining material was purified by flash chromatography on silica gel (EtOAc/hexane, 0 : 10 v/v) to give product **1n** (45 mg, 99%) as a colorless solid.

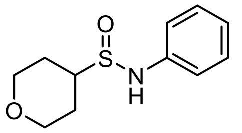

M.p.: 90–92 °C. – ^{1}H NMR (500 MHz, $CDCl_3$): 7.32 (1 H, s), 7.26 (2 H, t, *J* = 7.8 Hz), 7.06 (2 H, d, *J* = 8.0 Hz), 7.03 (1 H, t, *J* = 7.0 Hz), 4.04 (1 H, td, *J* = 12.2, 7.2 Hz), 3.37 (1 H, td, *J* = 11.5, 2.4 Hz), 3.26 (1 H, td, *J* = 11.6, 2.4 Hz), 3.13 (1 H, tt, *J* = 11.7, 4.2 Hz), 2.02 (1 H, ddd, *J* = 13.2, 4.4, 2.2 Hz), 1.92 (1 H, ddd, *J* = 13.2, 4.4, 2.3 Hz), 1.88–1.78 (1 H, m), 1.77–1.66 (1 H, m) ppm. – ^{13}C NMR (125 MHz, $CDCl_3$): 141.6, 129.5, 123.1, 118.1, 66.7, 66.6, 59.7, 27.0, 26.4 ppm. – IR: 3397, 3144, 2957, 2848, 1599, 1496, 1445, 1415, 1282, 1235, 1102, 1049, 881, 751 cm^{-1}.

Tert-butyl 3-((phenylamino)sulfinyl)piperidine-1-carboxylate (1o)

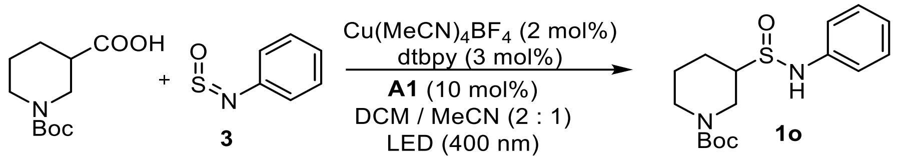

According to GP2, the reaction was carried out with 1-(tert-butoxycarbonyl)piperidine-3-carboxylic acid (49 mg, 0.2 mmol), sulfinylamine 3 prepared from aniline according the GP1 (70 mg, 0.5 mmol, 2.5 equiv.), acridine **A1** (5.8 mg, 0.03 mmol, 10 mol%), $Cu(MeCN)_4BF_4$ (1.3 mg, 0.004 mmol, 2 mol%), dtbpy (1.6 mg, 0.006 mmol, 3 mol%) and DCM : MeCN (2 : 1, v/v, 2 mL). Argon was passed on the surface of the reaction for 10 seconds. The test-tube was capped and the reaction mixture was irradiated with LED light (λ = 400 nm) while stirring at 25–27 °C for 16 h. For work-up, a saturated solution of EDTA (1.5 mL) was added, followed by DCM (5 mL). The reaction mixture was then extracted with DCM (3 × 5 mL). The organic phases were combined, dried over anhydrous Na_2SO_4, concentrated under reduced pressure and the remaining material was purified by flash chromatography on silica gel (EtOAc/hexane, 6 : 4 v/v) to give product **1o** (53 mg, 82%) as a colorless liquid.

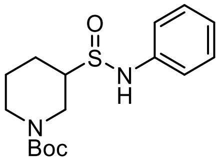

M.p.: 92–94 °C. – ^{1}H NMR (500 MHz, $CDCl_3$): 7.32 (2 H, t, J = 7.7 Hz), 7.09 (2 H, d, J = 7.8 Hz), 7.05 (1 H, t, J = 7.4 Hz), 3.94 (1 H, s), 3.59 (1 H, s), 3.24 (1 H, s), 2.12–1.93 (2 H, m), 1.86–1.67 (2 H, m), 1.56–1.43 (12 H, m) ppm. – ^{13}C NMR (125 MHz, $CDCl_3$): 154.5, 142.4, 129.5, 122.7, 118.0, 117.3, 79.4, 60.2, 43.6, 27.6, 24.53, 23.51 ppm. – IR: 3444, 3155, 2973, 2857, 1682, 1600, 1496, 1269, 1242, 1053, 887, 750 cm^{-1}. – HRMS: calcd for $C_{16}H_{24}N_2O_3S$: 325.1580, found 325.1579 [M+H$^+$].

***N*-Phenyladamantane-1-sulfinamide (1p)**

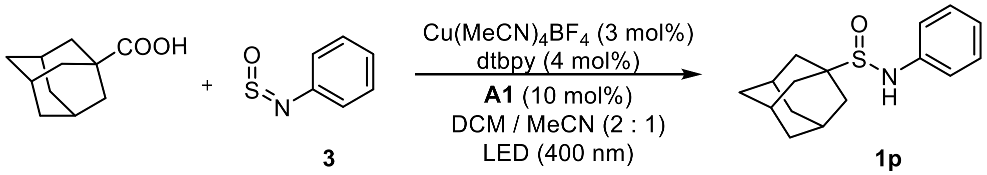

According to GP2, the reaction was carried out with adamantane-1-carboxylic acid (36 mg, 0.2 mmol), sulfinylamine 3 prepared from aniline according the GP1 (56 mg, 0.4 mmol, 2 equiv.), acridine **A1** (5.8

mg, 0.03 mmol, 10 mol%), $Cu(MeCN)_4BF_4$ (1.9 mg, 0.006 mmol, 3 mol%), dtbpy (2.2 mg, 0.008 mmol, 4 mol%) and DCM : MeCN (2 : 1, v/v, 2 mL). Argon was passed on the surface of the reaction for 10 seconds. The test-tube was capped and the reaction mixture was irradiated with LED light (λ = 400 nm) while stirring at 25–27 °C for 16 h. For work-up, a saturated solution of EDTA (1.5 mL) was added, followed by DCM (5 mL). The reaction mixture was then extracted with DCM (3 × 5 mL). The organic phases were combined, dried over anhydrous Na_2SO_4, concentrated under reduced pressure and the remaining material was purified by flash chromatography on silica gel (EtOAc/hexane, 3.5 : 6.5 v/v) to give product **1p** (48 mg, 87%) as a colorless solid.

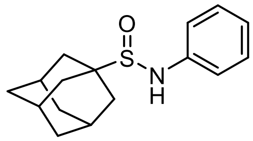

M.p.: 122–124 °C. – ^1H NMR (500 MHz, $CDCl_3$): 7.26 (2 H, t, *J* = 7.9 Hz), 7.04 (2 H d, *J* = 7.9 Hz), 7.00 (1 H, t, *J* = 7.4 Hz), 6.00 (1 H, s), 2.19 (3 H, t, *J* = 3.2 Hz), 2.03–1.89 (6 H, m), 1.84–1.67 (6 H, m) ppm. – ^{13}C NMR (125 MHz, $CDCl_3$): 142.4, 129.3, 122.6, 118.1, 58.3, 36.4, 34.7, 28.6 ppm. – IR: 3165, 3043, 2898, 2848, 1955, 1495, 1475, 1450, 1315, 1075, 1061 cm^{-1}. – HRMS: calcd for $C_{16}H_{21}NOS$: 276.1417, found 276.1416 [$M+H^+$].

3-Methyl-*N*-phenyloxetane-3-sulfinamide (1q)

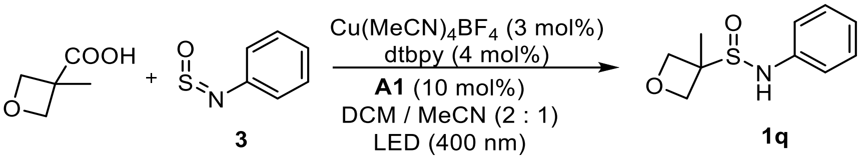

According to GP2, the reaction was carried out with 3-methyloxetane-3-carboxylic acid (23 mg, 0.2 mmol), sulfinylamine 3 prepared from aniline according the GP1 (56 mg, 0.4 mmol, 2 equiv.), acridine **A1** 5.8 mg, 0.03 mmol, 10 mol%), $Cu(MeCN)_4BF_4$ (1.9 mg, 0.006 mmol, 3 mol%), dtbpy (2.2 mg, 0.008 mmol, 4 mol%) and DCM : MeCN (2 : 1, v/v, 2 mL). Argon was passed on the surface of the reaction for 10 seconds. The test-tube was capped and the reaction mixture was irradiated with LED light (λ = 400 nm) while stirring at 25–27 °C for 16 h. For work-up, a saturated solution of EDTA (1.5 mL) was added, followed by DCM (5 mL). The reaction mixture was then extracted with DCM (3 × 5 mL). The organic phases were combined, dried over anhydrous Na_2SO_4, concentrated under reduced pressure and the remaining material was purified by flash chromatography on silica gel (EtOAc/hexane, 6 : 4 v/v) to give product **1q** (31 mg, 73%) as a colorless solid.

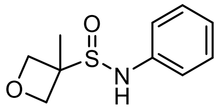

M.p.: 110–112 °C. – ^1H NMR (500 MHz, $CDCl_3$): 7.35–7.28 (2 H, m), 7.15–7.05 (3 H, m), 5.81 (1 H, s), 5.06 (1 H, d, *J* = 7.2 Hz), 4.95 (1 H, d, *J* = 7.5 Hz), 4.65–4.60 (2 H, m), 1.76 (3 H, s) ppm. – ^{13}C NMR (125 MHz, $CDCl_3$): 141.0, 129.6, 123.8, 118.9, 60.8, 15.9 ppm. – IR: 3419, 2874, 2621, 1705, 1634, 1601, 1539, 1498, 1454, 1362, 1218, 1158, 1029, 982 cm^{-1}. – HRMS: calcd for $C_{10}H_{13}NO_2S$: 212.0740, found 212.0737 [$M+H^+$].

1-Methyl-4-oxo-*N*-phenylcyclohexane-1-sulfinamide (1r)

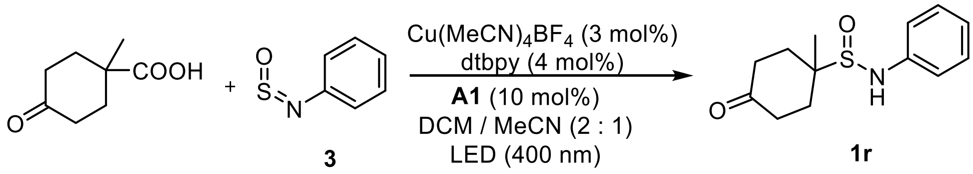

According to GP2, the reaction was carried out with 1-methyl-4-oxocyclohexane-1-carboxylic acid (31 mg, 0.2 mmol), sulfinylamine 3 prepared from aniline according the GP1 (56 mg, 0.4 mmol, 2 equiv.), acridine **A1** (5.8 mg, 0.03 mmol, 10 mol%), $Cu(MeCN)_4BF_4$ (1.9 mg, 0.006 mmol, 3 mol%), dtbpy (2.2 mg, 0.008 mmol, 4 mol%) and DCM : MeCN (2 : 1, v/v, 2 mL). Argon was passed on the surface of the reaction for 10 seconds. The test-tube was capped and the reaction mixture was irradiated with LED light (λ = 400 nm) while stirring at 25−27 °C for 16 h. For work-up, a saturated solution of EDTA (1.5 mL) was added, followed by DCM (5 mL). The reaction mixture was then extracted with DCM (3 × 5 mL). The organic phases were combined, dried over anhydrous Na_2SO_4, concentrated under reduced pressure and the remaining material was purified by flash chromatography on silica gel (EtOAc/hexane, 6 : 4 v/v) to give product **1r** (50 mg, 99%) as a colorless solid.

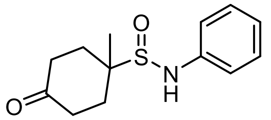

M.p.: 80–82 °C. – ^{1}H NMR (500 MHz, $CDCl_3$): 7.31–7.22 (3 H, m), 7.05 (2 H, d, *J* = 7.7 Hz), 7.02 (1 H, t, *J* = 7.4 Hz), 3.40 (2 H, t, *J* = 6.6 Hz), 3.10–2.95 (2 H, m), 1.87 (2 H, p, *J* = 7.1 Hz), 1.76 (2 H, p, *J* = 7.7 Hz), 1.68–1.45 (2 H, m) ppm. – ^{13}C NMR (125 MHz, $CDCl_3$): 141.3, 129.5, 123.1, 118.2, 55.5, 33.2, 32.2, 27.1, 22.6 ppm. – IR: 3419, 3185, 2955, 1712, 1599, 1497, 1416, 1339, 1281, 1219, 1140, 1057, 1029, 881 cm^{-1}. – HRMS: calcd for $C_{13}H_{17}NO_2S$: 274.0872, found 274.0878 [M+Na$^+$].

(1s,3*R*,5*S*,7s)-4-Oxo-*N*-phenyladamantane-1-sulfinamide (1s)

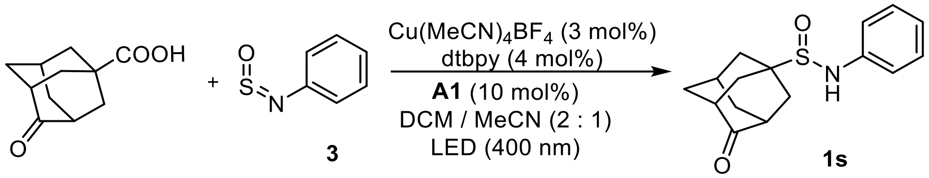

According to GP2, the reaction was carried out with (1s,3*R*,5*S*,7s)-4-oxoadamantane-1-carboxylic acid (39 mg, 0.2 mmol), sulfinylamine 3 prepared from aniline according the GP1 (56 mg, 0.4 mmol, 2 equiv.), acridine **A1** (5.8 mg, 0.03 mmol, 10 mol%), $Cu(MeCN)_4BF_4$ (1.9 mg, 0.006 mmol, 3 mol%), dtbpy (2.2 mg, 0.008 mmol, 4 mol%) and DCM : MeCN (2 : 1, v/v, 2 mL). Argon was passed on the surface of the reaction for 10 seconds. The test-tube was capped and the reaction mixture was irradiated with LED light (λ = 400 nm) while stirring at 25−27 °C for 16 h. For work-up, a saturated solution of EDTA (1.5 mL) was added, followed by DCM (5 mL). The reaction mixture was then extracted with DCM (3 × 5 mL). The organic phases were combined, dried over anhydrous Na_2SO_4, concentrated under reduced

pressure and the remaining material was purified by flash chromatography on silica gel (EtOAc/hexane, 6 : 4 v/v) to give product **1s** (41 mg, 70%) as a colorless solid.

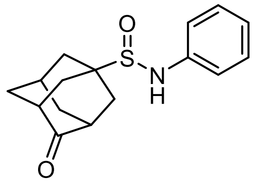

M.p.: 106–108 °C. – ^{1}H NMR (500 MHz, $CDCl_3$): 7.30–7.23 (2 H, m), 7.11–6.99 (3 H, m), 6.17 (1 H, s), 2.72 (2 H, t, *J* = 3.1 Hz), 2.35 (1 H, p, *J* = 3.1 Hz), 2.28–2.11 (7 H, m), 2.08 (1 H, s) ppm. – ^{13}C NMR (125 MHz, $CDCl_3$): 215.1, 141.8, 129.5, 123.1, 118.2, 57.2, 45.9, 45.8, 38.3, 38.2, 36.3, 35.8, 33.7, 27.9 ppm. –IR: 3184, 2926, 2857, 1719, 1599, 1496, 1454, 1403, 1281, 1224, 1061, 1031, 882 cm^{-1}. – HRMS: calcd for $C_{16}H_{19}NO_2S$: 290.1209, found 290.1204 [M+H$^+$].

Methyl 4-((phenylamino)sulfinyl)bicyclo[2.2.2]octane-1-carboxylate (1t)

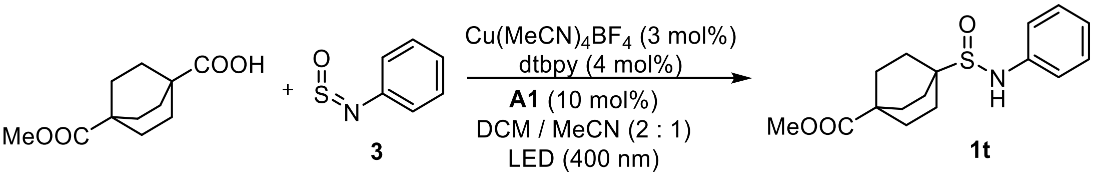

According to GP2, the reaction was carried out with 4-(methoxycarbonyl)bicyclo[2.2.2]octane-1-carboxylic acid (42 mg, 0.2 mmol), sulfinylamine 3 prepared from aniline according the GP1 (56 mg, 0.4 mmol, 2 equiv.), acridine **A1** (5.8 mg, 0.03 mmol, 10 mol%), $Cu(MeCN)_4BF_4$ (1.9 mg, 0.006 mmol, 3 mol%), dtbpy (2.2 mg, 0.008 mmol, 4 mol%) and DCM : MeCN (2 : 1, v/v, 2 mL). Argon was passed on the surface of the reaction for 10 seconds. The test-tube was capped and the reaction mixture was irradiated with LED light (λ = 400 nm) while stirring at 25–27 °C for 16 h. For work-up, a saturated solution of EDTA (1.5 mL) was added, followed by DCM (5 mL). The reaction mixture was then extracted with DCM (3 × 5 mL). The organic phases were combined, dried over anhydrous Na_2SO_4, concentrated under reduced pressure and the remaining material was purified by flash chromatography on silica gel (EtOAc/hexane, 8 : 2 v/v) to give product **1t** (58 mg, 94%) as a colorless solid.

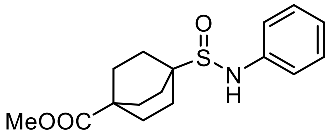

M.p.: 138–140 °C. – ^{1}H NMR (500 MHz, $CDCl_3$): 7.30–7.22 (2 H, m), 7.04–6.98 (3 H, m), 5.89 (1 H, s), 3.68 (3 H, s), 2.05–1.79 (12 H, m) ppm. – ^{13}C NMR (125 MHz, $CDCl_3$): 177.0, 141.9, 129.4, 122.9, 118.2, 57.3, 51.9, 39.0, 28.0, 23.9 ppm. – IR: 3041, 1726, 1597, 1453, 1238, 1053, 897, 785 cm^{-1}. – HRMS: calcd for $C_{16}H_{21}NO_3S$: 308.1315, found 308.1313 [M+H$^+$].

tert-Butyl 4-methyl-4-((phenylamino)sulfinyl)piperidine-1-carboxylate (1u)

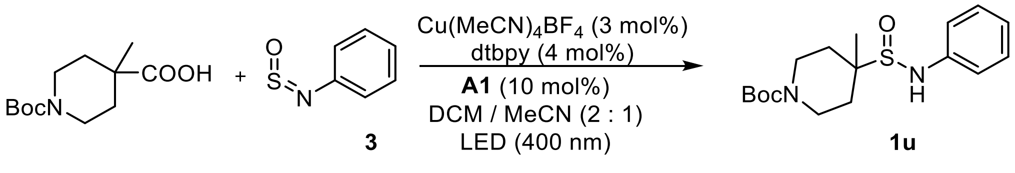

According to GP2, the reaction was carried out with 1-(*tert*-butoxycarbonyl)-4-methylpiperidine-4-carboxylic acid (49 mg, 0.2 mmol), sulfinylamine 3 prepared from aniline according the GP1 (56 mg, 0.4 mmol, 2 equiv.), acridine **A1** (5.8 mg, 0.03 mmol, 10 mol%), Cu(MeCN)$_4$BF$_4$ (1.9 mg, 0.006 mmol, 3 mol%), dtbpy (2.2 mg, 0.008 mmol, 4 mol%) and DCM : MeCN (2 : 1, v/v, 2 mL). Argon was passed on the surface of the reaction for 10 seconds. The test-tube was capped and the reaction mixture was irradiated with LED light (λ = 400 nm) while stirring at 25–27 °C for 16 h. For work-up, a saturated solution of EDTA (1.5 mL) was added, followed by DCM (5 mL). The reaction mixture was then extracted with DCM (3 × 5 mL). The organic phases were combined, dried over anhydrous Na_2SO_4, concentrated under reduced pressure and the remaining material was purified by flash chromatography on silica gel (EtOAc/hexane, 7 : 3 v/v) to give product **1u** (58 mg, 86%) as a colorless solid.

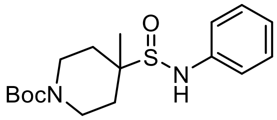

M.p.: 120–122 °C. – ^{1}H NMR (500 MHz, CDCl$_3$): 7.29 (2 H, t, *J* = 7.9 Hz), 7.05 (1 H, t, *J* = 7.5 Hz), 7.02 (2 H, d, *J* = 8.7 Hz), 5.58 (1 H, s), 4.08–3.83 (2 H, m), 3.30–3.04 (2 H, m), 2.07–1.96 (1 H, m), 1.89 (1 H, ddd, *J* = 14.4, 10.5, 4.5 Hz), 1.70–1.58 (2 H, m), 1.48 (9 H, s), 1.40 (3 H, s) ppm. – ^{13}C NMR (125 MHz, CDCl$_3$): 154.6, 141.7, 129.5, 123.3, 118.6, 78.00, 58.4, 39.1, 31.4, 28.4, 14.4 ppm. – IR: 3045, 2929, 2593, 1667, 1606, 1496, 1423, 1365, 1132, 1083, 1024, 906 cm^{-1}. – HRMS: calcd for $C_{17}H_{26}N_2O_3S$: 361.1556, found 361.1553 [M+H$^+$].

N-(4-Cyanophenyl)-4,4-difluorocyclohexane-1-sulfinamide (4a)

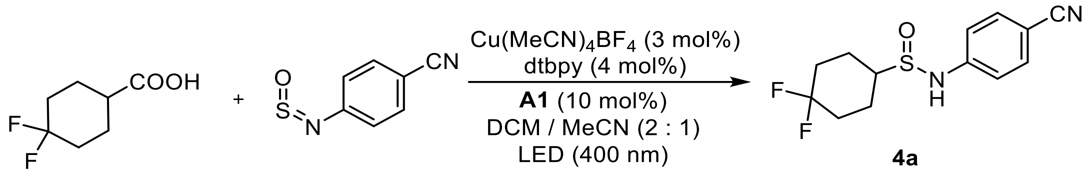

According to GP2, the reaction was carried out with 4,4-difluorocyclohexane-1-carboxylic acid (33 mg, 0.2 mmol), 4-((`-l4-sulfaneylidene)amino)benzonitrile (82 mg, 0.5 mmol, 2.5 equiv., prepared from 4-aminobenzonitrile, according to GP1), acridine **A1** (5.8 mg, 0.03 mmol, 10 mol%), Cu(MeCN)$_4$BF$_4$ (1.9 mg, 0.006 mmol, 3 mol%), dtbpy (2.2 mg, 0.008 mmol, 4 mol%) and DCM : MeCN (2 : 1, v/v, 2 mL). Argon was passed on the surface of the reaction for 10 seconds. The test-tube was capped and the reaction mixture was irradiated with LED light (λ = 400 nm) while stirring at 25–27 °C for 16 h. For work-up, a saturated solution of EDTA (1.5 mL) was added, followed by DCM (5 mL). The reaction mixture was then extracted with DCM (3 × 5 mL). The organic phases were combined, dried over anhydrous Na_2SO_4, concentrated under reduced pressure and the remaining material was purified by flash chromatography on silica gel (EtOAc/hexane, 9 : 1v/v) to give product **4a** (47 mg, 83%) as a colorless solid.

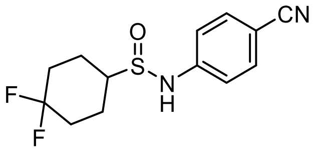

m.p.: 108–110 °C. ^{1}H NMR (500 MHz, CDCl$_3$): 7.59 (1 H, s), 7.53 (2 H, d, J = 8.3 Hz), 7.06 (2 H, d, J = 8.3 Hz), 3.00 (1 H, tt, J = 8.8, 4.0 Hz), 2.35–2.05 (4 H, m), 2.01–1.69 (4 H, m) ppm. – ^{13}C NMR (125 MHz, CDCl$_3$): 145.8, 133.8, 121.9

Go to table of contents

(t, J = 241.6 Hz), 118.7, 117.0, 105.8, 60.7, 32.2 (td, J = 25.2, 13.7 Hz), 22.7 (dd, J = 29.3, 8.4 Hz) ppm. – ^{19}F NMR (471 MHz, $CDCl_3$) δ -95.00 (d, *J* = 240.0 Hz), -100.70 (d, *J* = 241.5 Hz). – IR: 3352, 3228, 2926, 2221, 1630, 1604, 1508, 1376, 1150, 1106, 960 cm^{-1}. – HRMS: calcd for $C_{13}H_{14}F_2N_2OS$: 285.0868, found 285.0863 [$M+H^+$].

4-Methyl-*N*-(3-(trifluoromethoxy)phenyl)tetrahydro-2*H*-pyran-4-sulfinamide (4b)

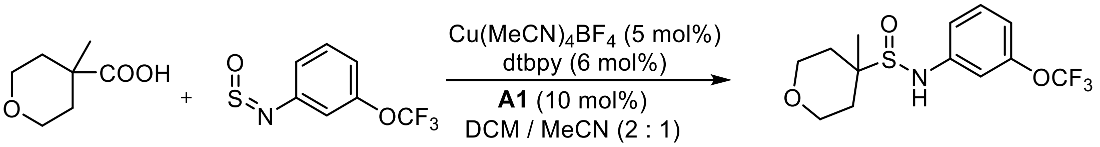

4b

According to GP2, the reaction was carried out with 4-methyltetrahydro-2*H*-pyran-4-carboxylic acid (29 mg, 0.2 mmol), ((3-(trifluoromethoxy)phenyl)imino)-l4-sulfanone (112 mg, 0.5 mmol, 2.5 equiv. prepared from 3-(trifluoromethoxy)aniline, according to GP1), acridine **A1** (5.8 mg, 0.03 mmol, 10 mol%), $Cu(MeCN)_4BF_4$ (3.1 mg, 0.01 mmol, 5 mol%), dtbpy (3.2 mg, 0.012 mmol, 6 mol%) and DCM : MeCN (2 : 1, v/v, 2 mL). Argon was passed on the surface of the reaction for 10 seconds. The test-tube was capped and the reaction mixture was irradiated with LED light (λ = 400 nm) while stirring at 25–27 °C for 16 h. For work-up, a saturated solution of EDTA (1.5 mL) was added, followed by DCM (5 mL). The reaction mixture was then extracted with DCM (3 × 5 mL). The organic phases were combined, dried over anhydrous Na_2SO_4, concentrated under reduced pressure and the remaining material was purified by flash chromatography on silica gel (EtOAc/hexane, 5 : 5 v/v) to give product **4b** (50 mg, 77%) as a colorless liquid.

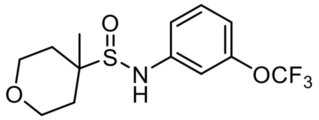

^1H NMR (500 MHz, $CDCl_3$): 7.23 (1 H, t, J = 8.1 Hz), 6.94 (1 H, d, J = 8.3 Hz), 6.88 (1 H, s), 6.84 (1 H, d, J = 8.0 Hz), 6.42 (1 H, s), 3.99 (1 H, dt, J = 12.0, 4.2 Hz), 3.93 (1 H, dt, J = 11.9, 4.2 Hz), 3.69–3.57 (2 H, m), 2.13 (2 H, ddd, J = 13.5, 10.6, 4.7 Hz), 2.02–1.93 (1 H, m), 1.65–1.51 (1 H, m), 1.44 (3 H, s) ppm. – ^{13}C NMR (125 MHz, $CDCl_3$): 145.0, 143.8, 130.5, 120.4 (q, J = 257.4 Hz), 115.7, 114.7, 110.4, 63.38, 63.35, 57.8, 31.6, 31.0, 14.5 ppm. – ^{19}F NMR (471 MHz, $CDCl_3$) δ -57.7 (s).– IR: 3170, 2963, 2859, 1612, 1494, 1392, 1259, 1218, 1160, 1105, 1059, 1001, 881, 765, 749 cm^{-1}. – HRMS: calcd for $C_{13}H_{16}F_3NO_3S$: 346.0695, found 346.0700 [$M+Na^+$].

N-(2,4,5-Trifluorophenyl)tetrahydro-2*H*-pyran-4-sulfinamide (4c)

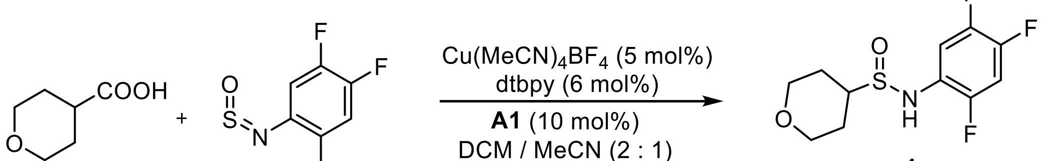

4c

According to GP2, the reaction was carried out with tetrahydro-2*H*-pyran-4-carboxylic acid (26 mg, 0.2 mmol), ((2,4,5-trifluorophenyl)imino)-l4-sulfanone (97 mg, 0.5 mmol, 2.5 equiv. prepared from 2,4,5-

trifluoroaniline according to GP1), acridine **A1** (5.8 mg, 0.03 mmol, 10 mol%), $Cu(MeCN)_4BF_4$ (3.1 mg, 0.01 mmol, 5 mol%), dtbpy (3.2 mg, 0.012 mmol, 6 mol%) and DCM : MeCN (2 : 1, v/v, 2 mL). Argon was passed on the surface of the reaction for 10 seconds. The test-tube was capped and the reaction mixture was irradiated with LED light (λ = 400 nm) while stirring at 25−27 °C for 16 h. For work-up, a saturated solution of EDTA (1.5 mL) was added, followed by DCM (5 mL). The reaction mixture was then extracted with DCM (3 × 5 mL). The organic phases were combined, dried over anhydrous Na_2SO_4, concentrated under reduced pressure and the remaining material was purified by flash chromatography on silica gel (EtOAc/hexane, 8 : 2 v/v) to give product **4c** (45 mg, 80%) as a colorless liquid.

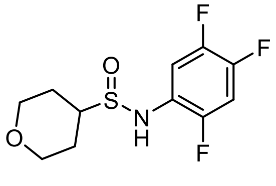

^{1}H NMR (500 MHz, $CDCl_3$): 7.17 (1 H, dt, J = 10.7, 7.9 Hz), 7.01 (1 H, td, J = 9.6, 7.6 Hz), 6.00 (1 H, s), 4.28–4.04 (2 H, m), 3.48 (2 H, tt, J = 11.6, 2.6 Hz), 3.08 (1 H, tt, J = 11.7, 4.2 Hz), 2.16–2.04 (1 H, m), 2.01–1.72 (3 H, m) ppm. – ^{13}C NMR (125 MHz, $CDCl_3$): 149.6–147.1, 147.9–145.6 (m), 146.9–144.3 (m), 125.7 (dd, J = 14.4, 6.7 Hz), 108.8–108.3 (m), 105.9 (ddd, J = 24.2, 17.6, 5.4 Hz), 66.8, 66.6, 60.3, 26.4, 26.0 ppm. – ^{19}F NMR (471 MHz, $CDCl_3$) δ -129.31 – -133.71 (m), -137.66 – -141.50 (m). – IR: 3063, 2964, 2917, 2848, 1645, 1520, 1446, 1417, 1267, 1222, 1129, 1050, 878, 788, 751 cm^{-1}. – HRMS: calcd for $C_{11}H_{12}F_3NO_2S$: 280.0614, found 280.0609 [M+H$^+$].

3,3-Dimethoxy-1-methyl-*N*-(pyridin-3-yl)cyclobutane-1-sulfinamide (4d)

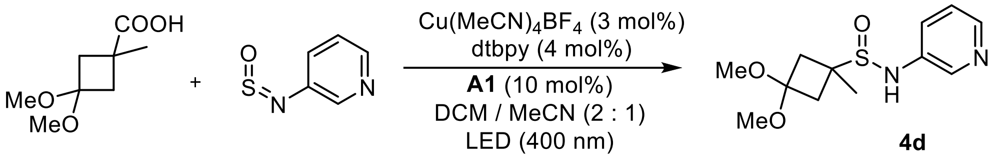

According to GP2, the reaction was carried out with 3,3-dimethoxy-1-methylcyclobutane-1-carboxylic acid (35 mg, 0.2 mmol), (pyridin-3-ylimino)-l4-sulfanone (70 mg, 0.5 mmol, 2.5 equiv. prepared from 3-aminopyridine according to GP1), acridine **A1** (5.8 mg, 0.03 mmol, 10 mol%), $Cu(MeCN)_4BF_4$ (1.9 mg, 0.006 mmol, 3 mol%), dtbpy (2.2 mg, 0.008 mmol, 4 mol%) and DCM : MeCN (2 : 1, v/v, 2 mL). Argon was passed on the surface of the reaction for 10 seconds. The test-tube was capped and the reaction mixture was irradiated with LED light (λ = 400 nm) while stirring at 25−27 °C for 16 h. For work-up, a saturated solution of EDTA (1.5 mL) was added, followed by DCM (5 mL). The reaction mixture was then extracted with DCM (3 × 5 mL). The organic phases were combined, dried over anhydrous Na_2SO_4, concentrated under reduced pressure and the remaining material was purified by flash chromatography on silica gel (MeOH/DCM, 1.5 : 8.5 v/v) to give product **4d** (46 mg, 84%) as a colorless liquid.

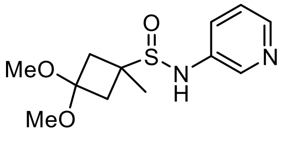

^{1}H NMR (500 MHz, $CDCl_3$): 8.37 (1 H, s), 8.28 (1 H, s), 7.43 (1 H, d, J = 7.0 Hz), 7.21 (1 H, dd, J = 8.6, 4.6 Hz), 6.48 (1 H, s), 3.21 (3 H, s), 3.18 (3 H, s), 2.84–2.62 (2 H, m), 2.21–2.12 (2 H, m), 1.63 (3 H, s) ppm. – ^{13}C NMR (125 MHz, $CDCl_3$): 144.1,

140.2, 138.7, 124.8, 123.9, 97.8, 52.5, 48.71, 48.65, 39.1, 38.1, 19.7 ppm. – IR: 3053, 2970, 1789, 1722, 1463, 1412, 1265, 1094, 735 cm^{-1}. – HRMS: calcd for $C_{12}H_{18}N_2O_3S$: 293.0930, found 293.0932 [M+Na$^+$].

N-(6-Methylpyridin-2-yl)cyclopent-3-ene-1-sulfinamide (4e)

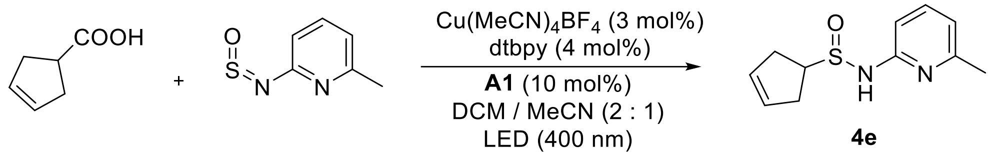

According to GP2, the reaction was carried out with cyclopent-3-ene-1-carboxylic acid (22 mg, 0.2 mmol), ((6-methylpyridin-2-yl)imino)-l4-sulfanone (77 mg, 0.5 mmol, 2.5 equiv. prepared from 1.0 equiv. 2-amino-6-methylpyridine according to modified GP1 with 2.0 equiv. triethylamine and 1.0 equiv. thionyl chloride in 0.6 (M) benzene under reflux for 2 h), acridine **A1** (5.8 mg, 0.03 mmol, 10 mol%), $Cu(MeCN)_4BF_4$ (1.9 mg, 0.006 mmol, 3 mol%), dtbpy (2.2 mg, 0.008 mmol, 4 mol%) and DCM : MeCN (2 : 1, v/v, 2 mL). Argon was passed on the surface of the reaction for 10 seconds. The test-tube was capped and the reaction mixture was irradiated with LED light (λ = 400 nm) while stirring at 25–27 °C for 16 h. For work-up, a saturated solution of EDTA (1.5 mL) was added, followed by DCM (5 mL). The reaction mixture was then extracted with DCM (3 × 5 mL). The organic phases were combined, dried over anhydrous Na_2SO_4, concentrated under reduced pressure and the remaining material was purified by flash chromatography on silica gel (EtOAc/hexane, 9 : 1 v/v) to give product **4e** (40 mg, 90%) as a colorless solid.

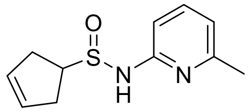

m.p.: 138–140 °C. – ^{1}H NMR (500 MHz, $CDCl_3$): 7.45 (2 H, t, J = 7.8 Hz), 6.75 (2 H, t, J = 8.5 Hz), 5.80–5.70 (2 H, m), 3.79 (1 H, tt, J = 8.9, 4.5 Hz), 2.94 (1 H, d, J = 17.8 Hz), 2.82–2.63 (3 H, m), 2.46 (3 H, s) ppm. – ^{13}C NMR (125 MHz, $CDCl_3$): 157.6, 154.1, 138.6, 129.2, 129.1, 117.2, 106.8, 61.3, 33.6, 33.4, 24.1 ppm. – IR: 3142, 2921, 2849, 1595, 1577, 1454, 1398, 1220, 1042, 948, 786 cm^{-1}. – HRMS: calcd for $C_{11}H_{14}N_2OS$: 245.0719, found 245.0723 [M+Na$^+$].

N-(5-Bromopyrimidin-2-yl)-5-chloropentane-1-sulfinamide (4f)

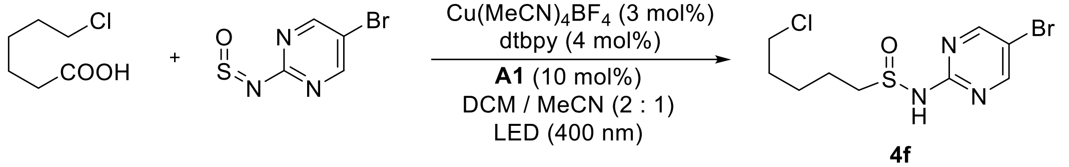

4f

According to GP2, the reaction was carried out with 6-chlorohexanoic acid (30 mg, 0.2 mmol), ((5-bromopyrimidin-2-yl)imino)-l4-sulfanone (110 mg, 0.5 mmol, 2.5 equiv. prepared from 1.0 equiv. 2-amino-5-bromopyrimidine, according to modified GP1 with 2.0 equiv. triethylamine and 1.0 equiv. thionyl chloride in 0.6M solution in benzene under reflux for 2 h,), acridine **A1** (5.8 mg, 0.03 mmol, 10

mol%), $Cu(MeCN)_4BF_4$ (1.9 mg, 0.006 mmol, 3 mol%), dtbpy (2.2 mg, 0.008 mmol, 4 mol%) and DCM : MeCN (2 : 1, v/v, 2 mL). Argon was passed on the surface of the reaction for 10 seconds. The test-tube was capped and the reaction mixture was irradiated with LED light (λ = 400 nm) while stirring at 25−27 °C for 16 h. For work-up, a saturated solution of EDTA (1.5 mL) was added, followed by DCM (5 mL). The reaction mixture was then extracted with DCM (3 × 5 mL). The organic phases were combined, dried over anhydrous Na_2SO_4, concentrated under reduced pressure and the remaining material was purified by flash chromatography on silica gel (EtOAc/hexane, 8 : 2 v/v) to give product **4f** (46 mg, 70%) as a colorless liquid.

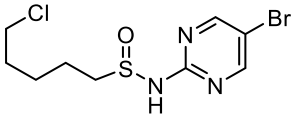

^{1}H NMR (500 MHz, $CDCl_3$): 8.69 (1 H, s), 8.52 (2 H, d, J = 1.1 Hz), 3.55 (2 H, t, J = 6.5 Hz), 3.10 (2 H, td, J = 7.4, 3.0 Hz), 1.88–1.78 (4 H, m), 1.73–1.58 (2 H, m) ppm. – ^{13}C NMR (125 MHz, $CDCl_3$): 159.0, 158.4, 111.9, 54.9, 44.5, 32.1, 25.9, 22.3 ppm. – IR: 3178, 2917, 2849, 1601, 1497, 1468, 1285, 1230, 1081, 1029, 888, 748 cm^{-1}.

_N_-(Benzo[d]thiazol-5-yl)-1-methylcyclohexane-1-sulfinamide (4g)

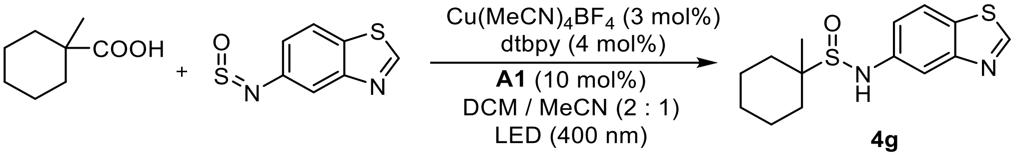

According to GP2, the reaction was carried out with 1-methylcyclohexane-1-carboxylic acid (28 mg, 0.2 mmol), ((1-phenylethyl)imino)-l4-sulfanone (98 mg, 0.5 mmol, 2.5 equiv. prepared from 1,3-benzothiazol-5-amine according to GP1), acridine A1 (5.8 mg, 0.03 mmol, 10 mol%), $Cu(MeCN)_4BF_4$ (1.9 mg, 0.006 mmol, 3 mol%), dtbpy (2.2 mg, 0.008 mmol, 4 mol%) and DCM : MeCN (2 : 1, v/v, 2 mL). Argon was passed on the surface of the reaction for 10 seconds. The test-tube was capped and the reaction mixture was irradiated with LED light (λ = 400 nm) while stirring at 25−27 °C for 16 h. For work-up, a saturated solution of EDTA (1.5 mL) was added, followed by DCM (5 mL). The reaction mixture was then extracted with DCM (3 × 5 mL). The organic phases were combined, dried over anhydrous Na_2SO_4, concentrated under reduced pressure and the remaining material was purified by flash chromatography on silica gel (EtOAc/hexane, 7 : 3 v/v) to give product 4g (55 mg, 93%) as a colorless liquid.

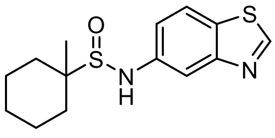

^{1}H NMR (500 MHz, $CDCl_3$): 8.95 (1 H, s), 7.76 (1 H, s), 7.70 (1 H, d, J = 8.6 Hz), 7.15 (1 H, d, J = 8.2 Hz), 6.26 (1 H, s), 1.93–1.84 (1 H, m), 1.84–1.44 (9 H, m), 1.35 (3 H, s) ppm. – ^{13}C NMR (125 MHz, $CDCl_3$): 155.2, 154.2, 141.7, 127.6, 122.2, 117.6, 111.9, 60.4, 32.1, 30.8, 25.5, 21.8, 21.6, 15.8 ppm. – IR: 2927, 2854, 1596, 1544, 1448, 1403, 1269, 1104, 1069, 998, 869, 799, 734 cm^{-1}. – HRMS: calcd for $C_{14}H_{18}N_2OS_2$: 317.0753 found 317.0762 [M+Na$^+$].

N-(4-Chlorophenethyl)-5-(2,5-dimethylphenoxy)-2-methylpentane-2-sulfinamide (4h)

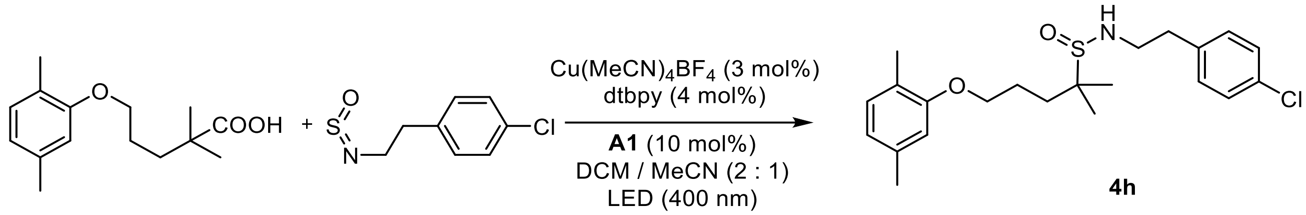

According to GP2, the reaction was carried out with 5-(2,5-dimethylphenoxy)-2,2-dimethylpentanoic acid (50 mg, 0.2 mmol), ((4-chlorobenzyl)imino)-l4-sulfanone (94 mg, 0.5 mmol, 2.5 equiv. prepared from 2-(4-chlorophenyl)ethylamine according to GP1), acridine A1 (5.8 mg, 0.03 mmol, 10 mol%), $Cu(MeCN)_4BF_4$ (1.9 mg, 0.006 mmol, 3 mol%), dtbpy (2.2 mg, 0.008 mmol, 4 mol%) and DCM : MeCN (2 : 1, v/v, 2 mL). Argon was passed on the surface of the reaction for 10 seconds. The test-tube was capped and the reaction mixture was irradiated with LED light (λ = 400 nm) while stirring at 25–27 °C for 16 h. For work-up, a saturated solution of EDTA (1.5 mL) was added, followed by DCM (5 mL). The reaction mixture was then extracted with DCM (3 × 5 mL). The organic phases were combined, dried over anhydrous Na_2SO_4, concentrated under reduced pressure and the remaining material was purified by flash chromatography on silica gel (EtOAc/hexane, 7 : 3 v/v) to give product 4h (70 mg, 86%) as a colorless liquid.

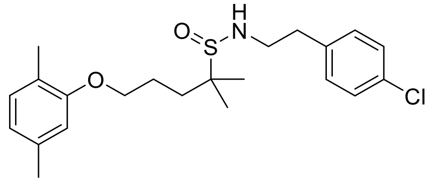

^{1}H NMR (500 MHz, $CDCl_3$): 7.28 (2 H, d, J = 8.3 Hz), 7.16 (2 H, d, J = 8.4 Hz), 7.04 (1 H, d, J = 7.4 Hz), 6.70 (1 H, d, J = 7.5 Hz), 6.64 (1 H, s), 3.94 (2 H, td, J = 6.0, 2.2 Hz), 3.55–3.44 (1 H, m), 3.40–3.26 (2 H, m), 2.95–2.83 (2 H, m), 2.34 (3 H, s), 2.20 (3 H, s), 1.96–1.76 (3 H, m), 1.65 (1 H, td, J = 12.3, 4.1 Hz), 1.22 (3 H, s), 1.19 (3 H, s)ppm. – ^{13}C NMR (125 MHz, $CDCl_3$): 156.8, 137.1, 136.6, 132.4, 130.38, 130.35, 128.7, 123.5, 120.9, 112.0, 67.7, 58.7, 47.0, 36.8, 33.0, 23.9, 21.5, 19.9, 19.3, 15.8 ppm. – IR: 3818, 3647, 3360, 3051, 2950, 1722, 1656, 1620, 1497, 1264, 1028, 736 cm^{-1}. – HRMS: calcd for $C_{22}H_{30}ClNO_2S$: 408.1759, found 408.1752[M+H$^+$].

N-(1-Phenylethyl)pentadecane-7-sulfinamide (4i)

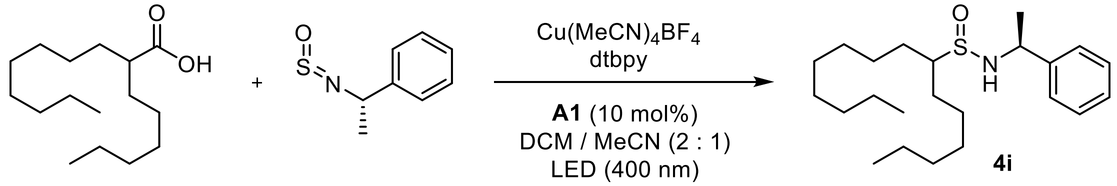

Go to table of contents

According to GP2, the reaction was carried out with 2-hexyldecanoic acid (51 mg, 0.2 mmol), ((1-phenylethyl)imino)-l4-sulfanone (84 mg, 0.5 mmol, 2.5 equiv. prepared from (*S*)-α-methylbenzylamine, according to GP1), acridine A1 (5.8 mg, 0.03 mmol, 10 mol%), $Cu(MeCN)_4BF_4$ (1.9 mg, 0.006 mmol, 3 mol%), dtbpy (2.2 mg, 0.008 mmol, 4 mol%) and DCM : MeCN (2 : 1, v/v, 2 mL). Argon was passed on the surface of the reaction for 10 seconds. The test-tube was capped and the reaction mixture was irradiated with LED light (λ = 400 nm) while stirring at 25–27 °C for 16 h. For work-up, a saturated solution of EDTA (1.5 mL) was added, followed by DCM (5 mL). The reaction mixture was then extracted with DCM (3 × 5 mL). The organic phases were combined, dried over anhydrous Na_2SO_4, concentrated under reduced pressure and the remaining material was purified by flash chromatography on silica gel (EtOAc/hexane, 2.5 : 7.5 v/v) to give product **4i** as a 1 : 1 mixture of diastereomers (70 mg, 92%, 1 : 1 dr) as a colorless liquid.

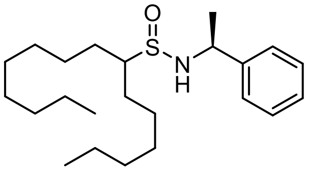

4i-1: ^{1}H NMR (500 MHz, $CDCl_3$): 7.40–7.23 (5 H, m), 4.61 (1 H, qd, J = 6.6, 2.8 Hz), 3.71 (1 H, d, J = 3.0 Hz), 2.54–2.46 (1 H, m), 1.92–1.21 (27 H, m), 0.90 (6 H, t, J = 6.8 Hz) ppm. – ^{13}C NMR (125 MHz, $CDCl_3$): 144.2, 128.8, 127.8, 126.6, 63.8, 52.9, 31.9, 31.6, 29.7, 29.4, 29.2, 27.9, 26.88, 26.85, 26.4, 26.3, 23.1, 22.7, 22.6, 14.12, 14.07 ppm. – IR: 2927, 2654, 1673, 1456, 1275, 1261, 763, 750 cm^{-1}.

4i-2: ^{1}H NMR (500 MHz, $CDCl_3$): 7.40–7.26 (5 H, m), 4.61 (1 H, qd, J = 6.7, 3.5 Hz), 3.56 (1 H, d, J = 3.8 Hz), 2.52–2.43 (1 H, m), 1.95–1.81 (1 H, m), 1.75–1.20 (26 H, m), 0.97–0.86 (6 H, m) ppm. – ^{13}C NMR (125 MHz, $CDCl_3$):143.5, 128.5, 127.5, 126.9, 64.5, 54.5, 31.9, 31.63, 31.60, 29.74, 29.71, 29.40, 29.36, 29.3, 28.0, 26.91, 26.87, 26.53, 26.48, 26.1, 25.1, 22.7, 22.6, 14.12, 14.07 ppm. – IR: 2924, 2853, 1672, 1490, 1448, 1376, 1264, 1059, 825, 738 cm^{-1}. – HRMS: calcd for $C_{23}H_{41}NOS$: 402.2801, found 402.2808 [M+Na$^+$].

Nonane-1-sulfinamide (6a)

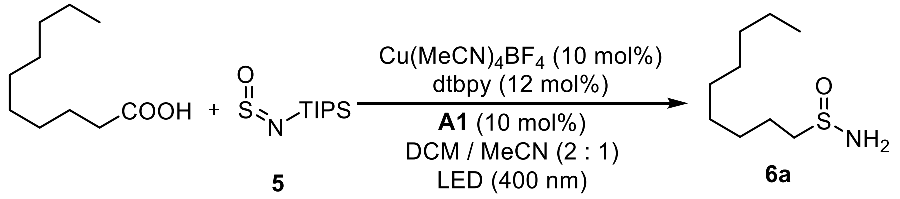

According to GP2, the reaction was carried out with decanoic acid (34 mg, 0.2 mmol), ((triisopropylsilyl)imino)-l4-sulfanone (110 mg, 0.5 mmol, 2.5 equiv. prepared from triisopropylsilanamine according to the literature procedure[4]), acridine **A1** (5.8 mg, 0.03 mmol, 10 mol%), $Cu(MeCN)_4BF_4$ (6.28 mg, 0.020 mmol, 10 mol%), dtbpy (6.43 mg, 0.024 mmol, 12 mol%) and DCM : MeCN (2 : 1, v/v, 2 mL). Argon was passed on the surface of the reaction for 10 seconds. The test-tube was capped and the reaction mixture was irradiated with LED light (λ = 400 nm) while stirring at 25–27 °C for 16 h. After completion of the first step mixture was treated with tetrabutylammonium fluoride (1 mmol, 5 equiv.) and stirred for additional 4 h at room temperature. For work-up, a saturated solution of EDTA (1.5 mL) was added, followed by DCM (5 mL). The reaction mixture was then

extracted with DCM (3 × 5 mL). The organic phases were combined, dried over anhydrous Na_2SO_4, concentrated under reduced pressure and the remaining material was purified by flash chromatography on silica gel (EtOAc/hexane, 9 : 1 v/v) to give product **6a** (34 mg, 89%) as a colorless liquid.

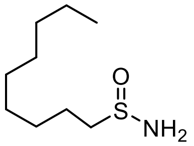

^{1}H NMR (500 MHz, $CDCl_3$): 4.08 (2 H, s), 2.83–2.68 (2 H, m), 1.80–1.66 (2 H, m), 1.54–1.18 (12 H, m), 0.90 (3 H, t, J = 6.8 Hz) ppm. – ^{13}C NMR (125 MHz, $CDCl_3$): 57.7, 31.8, 29.3, 29.24, 29.20, 28.6, 22.8, 22.7, 14.1 ppm. – IR: 3352, 2924, 2856, 1603, 1462, 1206, 1194, 1152, 1067, 1014, 881 cm^{-1}. – HRMS: calcd for $C_9H_{21}NOS$: 214.1236, found 214.1239 [M+Na$^+$].

5-Chloropentane-1-sulfinamide (6b)

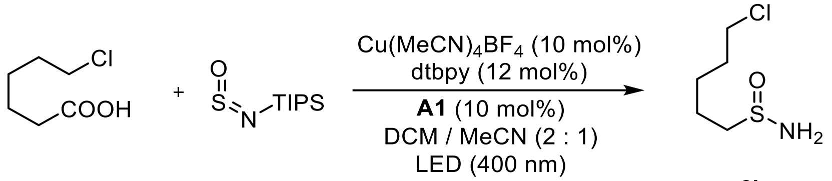

5 **6b**

According to GP2, the reaction was carried out with 6-chlorohexanoic acid (30 mg, 0.2 mmol), ((triisopropylsilyl)imino)-l4-sulfanone (110 mg, 0.5 mmol, 2.5 equiv. according to the literature procedure[4]), acridine **A1** (5.8 mg, 0.03 mmol, 10 mol%), $Cu(MeCN)_4BF_4$ (6.28 mg, 0.020 mmol, 10 mol%), dtbpy (6.43 mg, 0.024 mmol, 12 mol%) and DCM : MeCN (2 : 1, v/v, 2 mL). Argon was passed on the surface of the reaction for 10 seconds. The test-tube was capped and the reaction mixture was irradiated with LED light (λ = 400 nm) while stirring at 25–27 °C for 16 h. After completion of the first step mixture was treated with tetrabutylammonium fluoride (1 mmol, 5 equiv.) and stirred for additional 4 h at room temperature. For work-up, a saturated solution of EDTA (1.5 mL) was added, followed by DCM (5 mL). The reaction mixture was then extracted with DCM (3 × 5 mL). The organic phases were combined, dried over anhydrous Na_2SO_4, concentrated under reduced pressure and the remaining material was purified by flash chromatography on silica gel (EtOAc/hexane, 9 : 1 v/v) to give product **6b** (30 mg, 88%) as a colorless liquid.

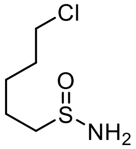

^{1}H NMR (500 MHz, $CDCl_3$): 4.24 (2 H, s), 3.57 (2 H, t, J = 6.5 Hz), 2.78 (2 H, tt, J = 13.2, 5.6 Hz), 1.91–1.70 (4 H, m), 1.67–1.49 (2 H, m) ppm. – ^{13}C NMR (125 MHz, $CDCl_3$): 57.3, 44.6, 32.1, 25.9, 22.3 ppm. – IR: 3745, 3396, 2943, 2866, 2692, 2173, 1603, 1460, 1246, 1204, 1151, 1038, 941 cm^{-1}. – HRMS: calcd for $C_5H_{12}ClNOS$: 192.0221, found 192.0220 [M+Na$^+$].

Cycloheptanesulfinamide (6c)

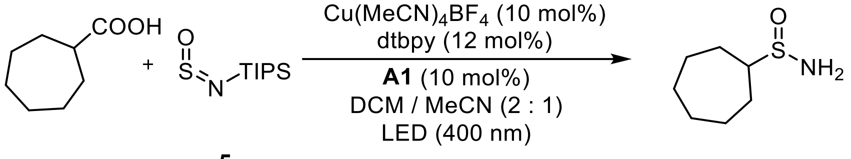

According to GP2, the reaction was carried out with cycloheptanecarboxylic acid (28 mg, 0.2 mmol), ((triisopropylsilyl)imino)-l4-sulfanone (110 mg, 0.5 mmol, 2.5 equiv. prepared from triisopropylsilanamine according to the literature procedure[4]), acridine **A1** (5.8 mg, 0.03 mmol, 10 mol%), $Cu(MeCN)_4BF_4$ (6.28 mg, 0.020 mmol, 10 mol%), dtbpy (6.43 mg, 0.024 mmol, 12 mol%) and DCM : MeCN (2 : 1, v/v, 2 mL). Argon was passed on the surface of the reaction for 10 seconds. The test-tube was capped and the reaction mixture was irradiated with LED light (λ = 400 nm) while stirring at 25–27 °C for 16 h. After completion of the first step mixture was treated with tetrabutylammonium fluoride (1 mmol, 5 equiv.) and stirred for additional 4 h at room temperature. For work-up, a saturated solution of EDTA (1.5 mL) was added, followed by DCM (5 mL). The reaction mixture was then extracted with DCM (3 × 5 mL). The organic phases were combined, dried over anhydrous Na_2SO_4, concentrated under reduced pressure and the remaining material was purified by flash chromatography on silica gel (EtOAc/hexane, 10 : 0 v/v) to give product **6c** (26 mg, 80%) as a colorless liquid.

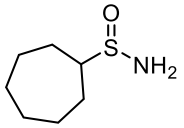

^{1}H NMR (500 MHz, $CDCl_3$): 3.94 (2 H, s), 2.62 (1 H, tt, J = 9.5, 4.3 Hz), 2.19 (1 H, ddd, J = 10.9, 7.0, 3.8 Hz), 2.05 (1 H, ddd, J = 11.8, 7.6, 3.8 Hz), 1.90–1.76 (2 H, m), 1.73–1.46 (8 H, m) ppm. – ^{13}C NMR (125 MHz, $CDCl_3$): 64.7, 28.6, 28.2, 27.0, 26.3, 26.1, 26.0 ppm. – IR: 3236, 2929, 2858, 1712, 1687, 1455, 1293, 1050, 1017, 902 cm^{-1}. – HRMS: calcd for $C_7H_{15}NOS$: 162.0947, found 162.0944 [M+Na$^+$].

(1S,3*R*,5*S*,7s)-4-Oxoadamantane-1-sulfinamide (6d)

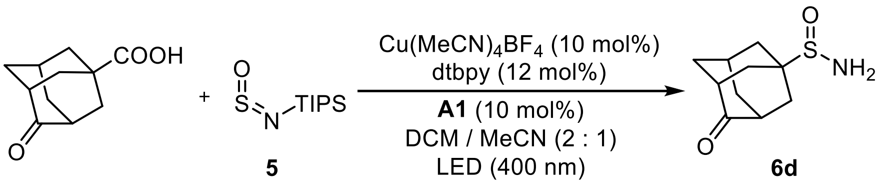

According to GP2, the reaction was carried out with (1s,3*R*,5*S*,7s)-4-oxoadamantane-1-carboxylic acid (39 mg, 0.2 mmol), ((triisopropylsilyl)imino)-l4-sulfanone (110 mg, 0.5 mmol, 2.5 equiv. prepared from triisopropylsilanamine according to the literature procedure[4]), acridine **A1** (5.8 mg, 0.03 mmol, 10 mol%), $Cu(MeCN)_4BF_4$ (6.28 mg, 0.020 mmol, 10 mol%), dtbpy (6.43 mg, 0.024 mmol, 12 mol%) and DCM : MeCN (2 : 1, v/v, 2 mL). Argon was passed on the surface of the reaction for 10 seconds. The test-tube was capped and the reaction mixture was irradiated with LED light (λ = 400 nm) while stirring at 25–27 °C for 16 h. After completion of the first step mixture was treated with tetrabutylammonium

fluoride (1 mmol, 5 equiv.) and stirred for additional 4 h at room temperature. For work-up, a saturated solution of EDTA (1.5 mL) was added, followed by DCM (5 mL). The reaction mixture was then extracted with DCM (3 × 5 mL). The organic phases were combined, dried over anhydrous Na_2SO_4, concentrated under reduced pressure and the remaining material was purified by flash chromatography on silica gel (MeOH/DCM, 1 : 12 v/v) to give product **6d** (29 mg, 68%) as a colorless liquid.

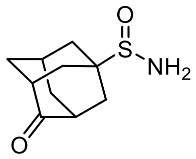

^{1}H NMR (500 MHz, $CDCl_3$): 3.99 (2 H, s), 2.75–2.68 (2 H, m), 2.39–2.34 (1 H, m), 2.24–2.01 (10 H, m) ppm. – ^{13}C NMR (125 MHz, $CDCl_3$): 215.5, 56.0, 45.83, 45.78, 38.4, 38.3, 36.2, 35.4, 33.2, 27.8 ppm. – IR: 3407, 3205, 3089, 2961, 2870, 1723, 1593, 1248, 1030, 897 cm^{-1}. – HRMS: calcd for $C_{10}H_{15}NO_2S$: 236.0716, found 236.0714 [M+Na^+].

Methyl 4-(aminosulfinyl)bicyclo[2.2.2]octane-1-carboxylate (6e)

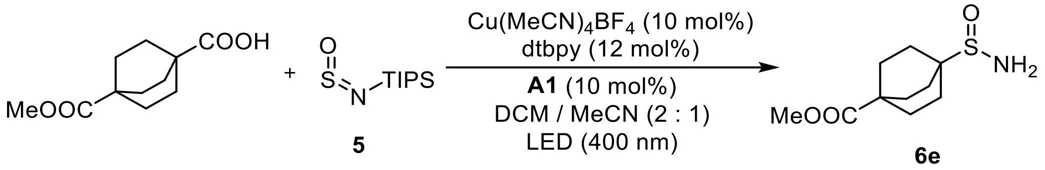

According to GP2, the reaction was carried out with 4-(methoxycarbonyl)bicyclo[2.2.2]octane-1-carboxylic acid (42 mg, 0.2 mmol), ((triisopropylsilyl)imino)-l4-sulfanone (110 mg, 0.5 mmol, 2.5 equiv. prepared from triisopropylsilanamine according to the literature procedure[4]), acridine **A1** (5.8 mg, 0.03 mmol, 10 mol%), $Cu(MeCN)_4BF_4$ (6.28 mg, 0.020 mmol, 10 mol%), dtbpy (6.43 mg, 0.024 mmol, 12 mol%) and DCM : MeCN (2 : 1, v/v, 2 mL). Argon was passed on the surface of the reaction for 10 seconds. The test-tube was capped and the reaction mixture was irradiated with LED light (λ = 400 nm) while stirring at 25–27 °C for 16 h. After completion of the first step mixture was treated with tetrabutylammonium fluoride (1 mmol, 5 equiv.) and stirred for additional 4 h at room temperature. For work-up, a saturated solution of EDTA (1.5 mL) was added, followed by DCM (5 mL). The reaction mixture was then extracted with DCM (3 × 5 mL). The organic phases were combined, dried over anhydrous Na_2SO_4, concentrated under reduced pressure and the remaining material was purified by flash chromatography on silica gel (EtOAc/hexane, 9 : 1 v/v) to give product **6e** (35 mg, 76%) as a colorless liquid.

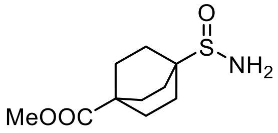

^{1}H NMR (500 MHz, $CDCl_3$): 3.78 (2 H, s), 3.68 (3 H, s), 2.01–1.71 (12 H, m) ppm. – ^{13}C NMR (125 MHz, $CDCl_3$): 177.2, 56.2, 51.9, 39.0, 28.0, 23.5 ppm. – IR: 3222, 2921, 2866, 1614, 1508, 1468, 1284, 1264, 1129,1034, 803 cm^{-1}. – HRMS: calcd for $C_{10}H_{17}NO_3S$: 232.1002, found 232.0996 [M+Na^+].

5-(2,5-Dimethylphenoxy)-2-methylpentane-2-sulfinamide (6f)

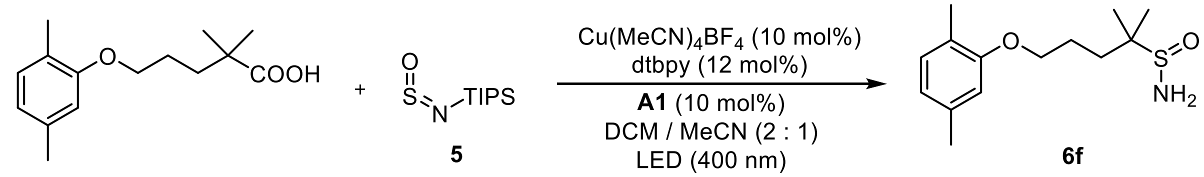

According to GP2, the reaction was carried out with 5-(2,5-dimethylphenoxy)-2,2-dimethylpentanoic acid (50 mg, 0.2 mmol), ((triisopropylsilyl)imino)-l4-sulfanone (110 mg, 0.5 mmol, 2.5 equiv. prepared from triisopropylsilanamine according to the literature procedure[4]), acridine **A1** (5.8 mg, 0.03 mmol, 10 mol%), $Cu(MeCN)_4BF_4$ (6.28 mg, 0.020 mmol, 10 mol%), dtbpy (6.43 mg, 0.024 mmol, 12 mol%) and DCM : MeCN (2 : 1, v/v, 2 mL). Argon was passed on the surface of the reaction for 10 seconds. The test-tube was capped and the reaction mixture was irradiated with LED light ($\lambda = 400$ nm) while stirring at 25–27 °C for 16 h. After completion of the first step mixture was treated with tetrabutylammonium fluoride (1 mmol, 5 equiv.) and stirred for additional 4 h at room temperature. For work-up, a saturated solution of EDTA (1.5 mL) was added, followed by DCM (5 mL). The reaction mixture was then extracted with DCM (3 × 5 mL). The organic phases were combined, dried over anhydrous Na_2SO_4, concentrated under reduced pressure and the remaining material was purified by flash chromatography on silica gel (EtOAc/hexane, 7 : 3 v/v) to give product **6f** (40 mg, 74%) as a colorless liquid.

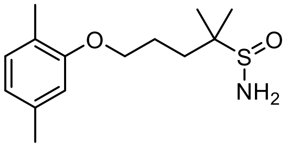

^{1}H NMR (500 MHz, $CDCl_3$): 7.03 (1 H, d, J = 7.4 Hz), 6.69 (1 H, d, J = 7.4 Hz), 6.63 (1 H, s), 4.05–3.92 (2 H, m), 3.88 (2 H, s), 2.33 (3 H, s), 2.20 (3 H, s), 2.04–1.64 (4 H, m), 1.27 (3 H, s), 1.25 (3 H, s) ppm. – ^{13}C NMR (125 MHz, $CDCl_3$): 156.8, 136.5, 130.4, 123.6, 120.9, 112.0, 67.8, 58.2, 32.5, 23.9, 21.4, 19.2, 19.1, 15.8 ppm. – IR: 3235, 2921, 2853, 1645, 1458, 1172, 1015, 885 cm^{-1}. – HRMS: calcd for $C_{14}H_{23}NO_2S$: 292.1342, found 292.1344 [M+Na$^+$].

5-(2,5-Dimethylphenoxy)-2-methyl-*N*-phenylpentane-2-sulfinamide (7a)

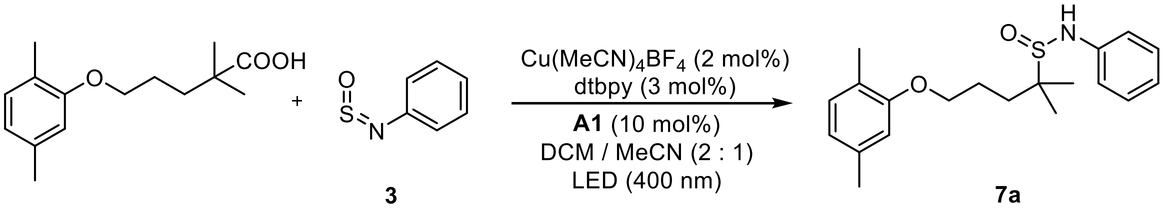

According to GP2, the reaction was carried out with 5-(2,5-dimethylphenoxy)-2,2-dimethylpentanoic acid (50 mg, 0.2 mmol), sulfinylamine 3 prepared from aniline according the GP1 (56 mg, 0.4 mmol, 2 equiv. prepared from triisopropylsilanamine according to the literature procedure[4]), acridine **A1** (5.8 mg, 0.03 mmol, 10 mol%), $Cu(MeCN)_4BF_4$ (1.3 mg, 0.004 mmol, 2 mol%), dtbpy (1.6 mg, 0.006 mmol, 3 mol%) and DCM : MeCN (2 : 1, v/v, 2 mL). Argon was passed on the surface of the reaction for 10

seconds. The test-tube was capped and the reaction mixture was irradiated with LED light (λ = 400 nm) while stirring at 25–27 °C for 16 h. After completion of the first step mixture was treated with tetrabutylammonium fluoride (1 mmol, 5 equiv.) and stirred for additional 4 h at room temperature. For work-up, a saturated solution of EDTA (1.5 mL) was added, followed by DCM (5 mL). The reaction mixture was then extracted with DCM (3 × 5 mL). The organic phases were combined, dried over anhydrous Na_2SO_4, concentrated under reduced pressure and the remaining material was purified by flash chromatography on silica gel (EtOAc/hexane, 3.5 : 6.5 v/v) to give product **7a** (69 mg, 99%) as a colorless liquid.

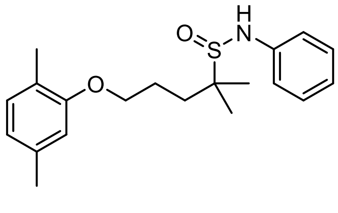

^{1}H NMR (500 MHz, $CDCl_3$): 7.33–7.25 (2 H, m), 7.09–6.98 (4 H, m), 6.71 (1 H, d, J = 7.4 Hz), 6.65 (1 H, s), 5.73 (1 H, s), 3.99 (2 H, dt, J = 4.0, 2.0 Hz), 2.35 (3 H, s), 2.20 (3 H, s), 2.07–1.78 (4 H, m), 1.39 (3 H, s), 1.37 (3 H, s) ppm. – ^{13}C NMR (125 MHz, $CDCl_3$): 156.8, 142.1, 136.5, 130.4, 129.4, 123.6, 123.0, 120.9, 118.4, 112.0, 67.7, 59.5, 32.7, 23.9, 21.4, 19.7, 19.4, 15.8 ppm. – IR: 3167, 3044, 2920, 2865, 1598, 1507, 1495, 1444, 1384, 1338, 1128 cm^{-1}. – HRMS: calcd for $C_{20}H_{27}NO_2S$: 346.1835, found 346.1834 [M+H$^+$].

(*E*)-5-(4-Hydroxy-6-methoxy-7-methyl-3-oxo-1,3-dihydroisobenzofuran-5-yl)-3-methyl-*N*-phenylpent-3-ene-1-sulfinamide (7b)

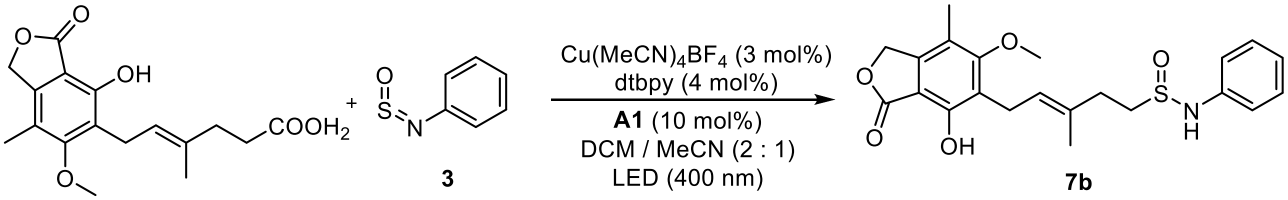

According to GP2, the reaction was carried out with (*E*)-6-(4-hydroxy-6-methoxy-7-methyl-3-oxo-1,3-dihydroisobenzofuran-5-yl)-4-methylhex-4-enoic acid (64 mg, 0.2 mmol), sulfinylamine 3 prepared from aniline according the GP1 (56 mg, 0.4 mmol, 2 equiv. prepared from aniline according to the literature procedure[4]), acridine **A1** (5.8 mg, 0.03 mmol, 10 mol%), $Cu(MeCN)_4BF_4$ (1.9 mg, 0.006 mmol, 3 mol%), dtbpy (2.2 mg, 0.008 mmol, 4 mol%) and DCM : MeCN (2 : 1, v/v, 2 mL). Argon was passed on the surface of the reaction for 10 seconds. The test-tube was capped and the reaction mixture was irradiated with LED light (λ = 400 nm) while stirring at 25–27 °C for 16 h. For work-up, a saturated solution of EDTA (1.5 mL) was added, followed by DCM (5 mL). The reaction mixture was then extracted with DCM (3 × 5 mL). The organic phases were combined, dried over anhydrous Na_2SO_4, concentrated under reduced pressure and the remaining material was purified by flash chromatography on silica gel (EtOAc/hexane, 8 : 2 v/v) to give product **7b** (58 mg, 70%) as a colorless liquid.

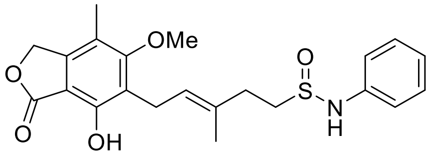

^{1}H NMR (500 MHz, $CDCl_3$): 7.72 (1 H, s), 7.21 (2 H, t, *J* = 7.9 Hz), 7.02–6.94 (3 H, m), 6.82 (1 H, s), 5.40–5.32 (1 H, m), 5.21 (2 H, s), 3.77 (3 H, s), 3.42 (2 H, d, *J* = 7.1 Hz), 3.21–2.99 (2 H, m), 2.54–2.36 (2 H, m), 2.16 (3 H, s), 1.85 (3 H, s) ppm. – ^{13}C NMR (125 MHz, $CDCl_3$): 172.9, 163.6, 153.6, 144.2, 141.2, 132.4, 129.4, 124.5, 123.1, 121.7, 118.5, 116.8, 106.4, 70.1, 61.0, 54.1, 32.8, 22.7, 16.2, 11.6 ppm. – IR: 3418, 2924, 1731, 1621, 1601, 1496, 1454, 1410, 1367 1328, 1193, 1135 cm^{-1}. – HRMS: calcd for $C_{22}H_{25}NO_5S$: 416.1526, found 416.1527 [M+H$^+$].

Tert-butyl 2-((phenylamino)sulfinyl)pyrrolidine-1-carboxylate (7c)

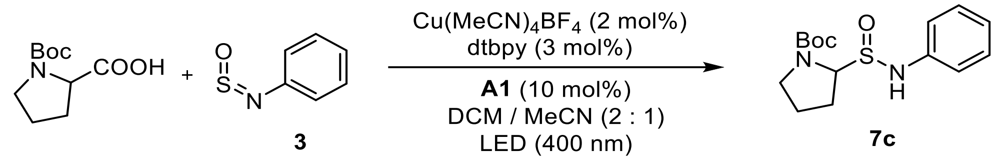

According to GP2, the reaction was carried out with (tert-butoxycarbonyl)proline (43 mg, 0.2 mmol), sulfinylamine 3 prepared from aniline according the GP1 (70 mg, 0.5 mmol, 2.5 equiv.), acridine **A1** (5.8 mg, 0.03 mmol, 10 mol%), $Cu(MeCN)_4BF_4$ (1.3 mg, 0.004 mmol, 2 mol%), dtbpy (1.6 mg, 0.006 mmol, 3 mol%) and DCM : MeCN (2 : 1, v/v, 2 mL). Argon was passed on the surface of the reaction for 10 seconds. The test-tube was capped and the reaction mixture was irradiated with LED light (λ = 400 nm) while stirring at 25–27 °C for 16 h. For work-up, a saturated solution of EDTA (1.5 mL) was added, followed by DCM (5 mL). The reaction mixture was then extracted with DCM (3 × 5 mL). The organic phases were combined, dried over anhydrous Na_2SO_4, concentrated under reduced pressure and the remaining material was purified by flash chromatography on silica gel (EtOAc/hexane, 6 : 4 v/v) to give product **7c** (50 mg, 80%, 1 : 1 dr, **7c-1**/**7c-2**) as a colorless liquid.

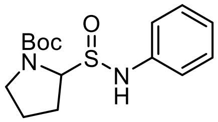

7c-1: ^{1}H NMR (500 MHz, $CDCl_3$): 7.33 (2 H, t, J = 7.7 Hz), 7.13–7.04 (3 H, m), 3.67–3.28 (4 H, m), 2.41–1.92 (4 H, m), 1.45 (9 H, s) ppm. – ^{13}C NMR (125 MHz, $CDCl_3$): 142.0, 129.5, 123.0, 118.23, 118.18, 117.3, 79.0, 46.0, 44.9, 27.7, 27.6 ppm. – IR: 3355, 2973, 2928, 1689, 1618, 1511, 1455, 1394, 1365, 1255, 1166, 1116, 749 cm^{-1}.

7c-2: ^{1}H NMR (500 MHz, $CDCl_3$): 7.38–7.28 (2 H, m), 7.16–7.02 (3 H, m, 3H), 3.85–3.77 (1 H, m), 3.63 (2 H, d, J = 8.0 Hz), 3.52–3.34 (2 H, m), 2.32–2.08 (2 H, m), 2.02–1.93 (1 H, m), 1.47 (9 H, s) ppm. – ^{13}C NMR (125 MHz, $CDCl_3$): 142.1, 129.5, 122.9, 118.2, 117.3, 79.0, 45.6, 45.3, 44.8, 27.6, 25.7 ppm. – IR: 3362, 3225, 2956, 2210, 1722, 1628, 1604, 1515, 1317, 1172, 831 cm^{-1}. – IR: 3355, 2974, 2930, 1676, 1604, 1499, 1456, 1401, 1365, 1256, 1167, 1119, 874, 750 cm^{-1}.

tert-Butyl (tert-butoxycarbonyl)((phenylamino)sulfinyl)alaninate (7d)

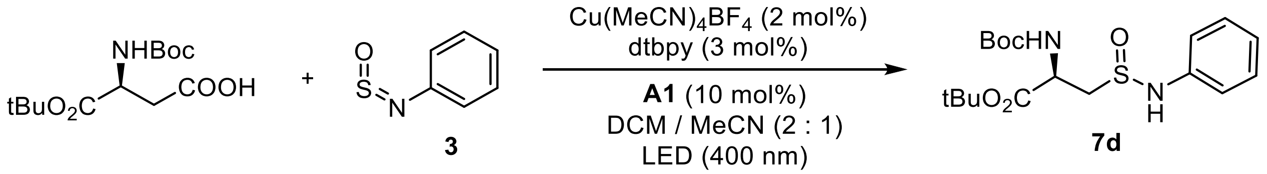

According to GP2, the reaction was carried out with 4-(*tert*-butoxy)-3-((*tert*-butoxycarbonyl)amino)-4-oxobutanoic acid (58 mg, 0.2 mmol), sulfinylamine 3 prepared from aniline according the GP1 (56 mg, 0.4 mmol, 2 equiv.), acridine **A1** (5.8 mg, 0.03 mmol, 10 mol%), $Cu(MeCN)_4BF_4$ (1.3 mg, 0.004 mmol, 2 mol%), dtbpy (1.6 mg, 0.006 mmol, 3 mol%) and DCM : MeCN (2 : 1, v/v, 2 mL). Argon was passed on the surface of the reaction for 10 seconds. The test-tube was capped and the reaction mixture was irradiated with LED light (λ = 400 nm) while stirring at 25–27 °C for 16 h. For work-up, a saturated solution of EDTA (1.5 mL) was added, followed by DCM (5 mL). The reaction mixture was then extracted with DCM (3 × 5 mL). The organic phases were combined, dried over anhydrous Na_2SO_4, concentrated under reduced pressure and the remaining material was purified by flash chromatography on silica gel (EtOAc/hexane, 3 : 7 v/v) to give product **7d** (39 mg, 51%, 1 : 1 dr) as a colorless solid.

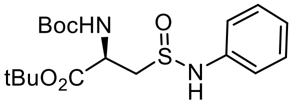

M.p.: 73–75 °C. – ^{1}H NMR (500 MHz, $CDCl_3$): 7.29 – 7.22 (2 H, m), 7.20 – 6.95 (4 H, m), 5.62 (1 H, dd, *J* = 15.6, 7.7 Hz), 4.74 – 4.51 (1 H, m), 3.71 – 3.28 (2 H, m), 1.48 (18 H, m) ppm. – ^{13}C NMR (125 MHz, $CDCl_3$): ^{13}C NMR (126 MHz, $CDCl_3$) δ 169.5, 169.4, 155.4, 129.5, 129.5, 123.7, 123.4, 119.4, 118.7, 83.4, 80.6, 57.1, 50.3, 49.9, 28.3, 27.9 ppm. – IR: 3346, 2977, 2931, 1731, 1697, 1600, 1522, 1496, 1392, 1367, 1354, 1246, 1150, 1031 cm^{-1}. – HRMS: calcd for $C_{18}H_{28}N_2O_5S$: 385.1792, found 385.1793 [M+H$^+$].

2-(4,5-Diphenyloxazol-2-yl)-*N*-phenylethane-1-sulfinamide (7e)

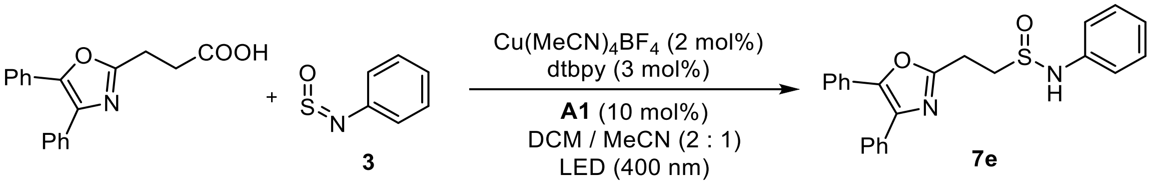

According to GP2, the reaction was carried out with 3-(4,5-diphenyloxazol-2-yl)propanoic acid (59 mg, 0.2 mmol), sulfinylamine 3 prepared from aniline according the GP1 (56 mg, 0.4 mmol, 2 equiv.), acridine **A1** (5.8 mg, 0.03 mmol, 10 mol%), $Cu(MeCN)_4BF_4$ (1.3 mg, 0.004 mmol, 2 mol%), dtbpy (1.6 mg, 0.006 mmol, 3 mol%) and DCM : MeCN (2 : 1, v/v, 2 mL). Argon was passed on the surface of the reaction for 10 seconds. The test-tube was capped and the reaction mixture was irradiated with LED light (λ = 400 nm) while stirring at 25–27 °C for 16 h. For work-up, a saturated solution of EDTA (1.5 mL) was added, followed by DCM (5 mL). The reaction mixture was then extracted with DCM (3 × 5 mL). The organic phases were combined, dried over anhydrous Na_2SO_4, concentrated under reduced

pressure and the remaining material was purified by flash chromatography on silica gel (EtOAc/hexane, 7 : 3 v/v) to give product **7e** (54 mg, 70%) as a colorless liquid.

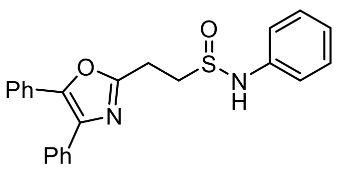

M.p.: 95–97°C. – ^{1}H NMR (500 MHz, $CDCl_3$): 8.43 (1 H, s), 7.78–7.64 (2 H, m), 7.64–7.57 (2 H, m), 7.39 (6 H, dt, *J* = 14.4, 7.2 Hz), 7.24 (2 H, t, *J* = 7.8 Hz), 7.13–6.95 (3 H, m), 3.71–3.47 (3 H, m), 3.28 (1 H, dt, *J* = 16.8, 5.7 Hz) ppm. – ^{13}C NMR (125 MHz, $CDCl_3$): 161.3, 146.1, 141.3, 134.9, 132.0, 129.5, 128.8, 128.74, 128.67, 128.5, 128.4, 127.9, 126.6, 123.0, 118.1, 45.0, 20.5 ppm. – IR: 3420, 1669, 1595, 1580, 1497, 1448, 1314, 1211, 1174, 1072, 1041, 964 cm^{-1}. – HRMS: calcd for $C_{23}H_{20}N_2O_2S$: 389.1318, found 389.1318 [M+H$^+$].

1-(11-Oxo-6,11-dihydrodibenzo[*b*,*e*]oxepin-2-yl)-*N*-phenylmethanesulfinamide (7f)

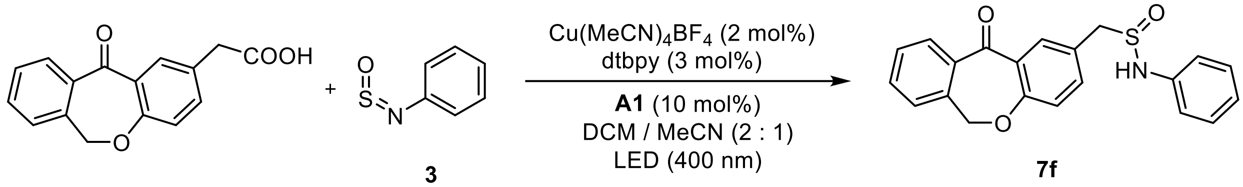

According to GP2, the reaction was carried out with 2-(11-oxo-6,11-dihydrodibenzo[*b*,*e*]oxepin-2-yl)acetic acid (54 mg, 0.2 mmol), sulfinylamine 3 prepared from aniline according the GP1 (56 mg, 0.4 mmol, 2 equiv.), acridine **A1** (5.8 mg, 0.03 mmol, 10 mol%), $Cu(MeCN)_4BF_4$ (1.3 mg, 0.004 mmol, 2 mol%), dtbpy (1.6 mg, 0.006 mmol, 3 mol%) and DCM : MeCN (2 : 1, v/v, 2 mL). Argon was passed on the surface of the reaction for 10 seconds. The test-tube was capped and the reaction mixture was irradiated with LED light (λ = 400 nm) while stirring at 25–27 °C for 16 h. For work-up, a saturated solution of EDTA (1.5 mL) was added, followed by DCM (5 mL). The reaction mixture was then extracted with DCM (3 × 5 mL). The organic phases were combined, dried over anhydrous Na_2SO_4, concentrated under reduced pressure and the remaining material was purified by flash chromatography on silica gel (EtOAc/hexane, 8 : 2 v/v) to give product **7f** (40 mg, 55%) as a colorless solid.

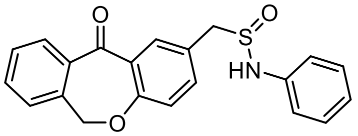

M.p.: 98–100 °C. – ^{1}H NMR (500 MHz, $CDCl_3$): 8.23 (1 H, s), 7.92 (1 H, d, *J* = 7.7 Hz), 7.60 (1 H, t, *J* = 7.5 Hz), 7.55–7.48 (2 H, m), 7.40 (1 H, d, *J* = 7.5 Hz), 7.30–7.22 (2 H, m), 7.10 (1 H, dd, *J* = 8.4, 1.5 Hz), 7.04 (1 H, t, *J* = 7.4 Hz), 7.00 (1 H, d, *J* = 7.9 Hz), 6.13 (1 H, s), 5.23 (2 H, s), 4.31 (1 H, d, *J* = 13.1 Hz), 4.16 (1 H, d, *J* = 13.1 Hz) ppm. – ^{13}C NMR (125 MHz, $CDCl_3$): 190.6, 161.5, 140.7, 140.3, 137.4, 135.4, 133.9, 133.0, 129.6, 129.5, 129.4, 127.9, 125.4, 123.6, 122.7, 121.7, 119.2, 73.7, 60.3 ppm. – IR: 2921, 2851, 1731, 1694, 1647, 1600, 1490, 1456, 1413, 1301, 1240, 1139 cm^{-1}. – HRMS: calcd for $C_{21}H_{17}NO_3S$: 364.1002, found 364.1000 [M+H$^+$].

(*Z*)-*N*-Phenylhenicos-12-ene-1-sulfinamide (7g)

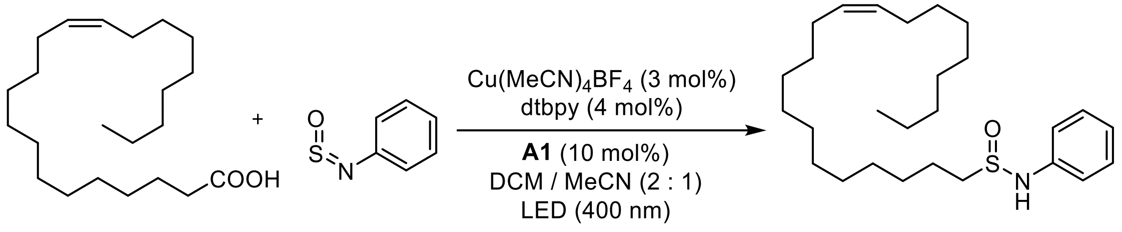

3 **7g**

According to GP2, the reaction was carried out with (*Z*)-docos-13-enoic acid (68 mg, 0.2 mmol), sulfinylamine 3 prepared from aniline according the GP1 (56 mg, 0.4 mmol, 2 equiv.), acridine **A1** (5.8 mg, 0.03 mmol, 10 mol%), $Cu(MeCN)_4BF_4$ (1.9 mg, 0.006 mmol, 3 mol%), dtbpy (2.2 mg, 0.008 mmol, 4 mol%) and DCM : MeCN (2 : 1, v/v, 2 mL). Argon was passed on the surface of the reaction for 10 seconds. The test-tube was capped and the reaction mixture was irradiated with LED light (λ = 400 nm) while stirring at 25–27 °C for 16 h. For work-up, a saturated solution of EDTA (1.5 mL) was added, followed by DCM (5 mL). The reaction mixture was then extracted with DCM (3 × 5 mL). The organic phases were combined, dried over anhydrous Na_2SO_4, concentrated under reduced pressure and the remaining material was purified by flash chromatography on silica gel (EtOAc/hexane, 4 : 6 v/v) to give product **7g** (52 mg, 61%) as a colorless solid.

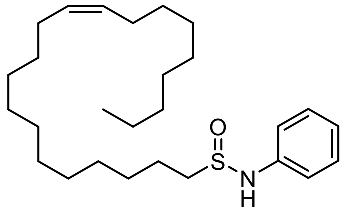

M.p.: 48–50 °C. – ^{1}H NMR (500 MHz, $CDCl_3$): 7.27 (2 H, t, *J* = 8.0 Hz), 7.07 (2 H, d, *J* = 8.0 Hz), 7.03 (1 H, t, *J* = 7.4 Hz), 5.42–5.33 (2 H, m), 3.07–2.97 (2 H, m), 2.04 (4 H, q, *J* = 6.5 Hz), 1.74 (2 H, p, *J* = 7.7 Hz), 1.53–1.18 (28 H, m), 0.90 (3 H, t, *J* = 6.8 Hz) ppm. – ^{13}C NMR (125 MHz, $CDCl_3$): 141.3, 129.93, 129.86, 129.5, 129.4, 123.1, 118.4, 55.9, 31.9, 29.8, 29.72, 29.67, 29.60, 29.55, 29.4, 29.33, 29.25, 28.6, 27.2, 23.3, 22.7, 14.1 ppm. – IR: 2918, 2849, 1601, 1498, 1466, 1036, 889 cm^{-1}. – HRMS: calcd for $C_{27}H_{47}NOS$: 434.3451, found 434.3450 [M+H$^+$].

(8*Z*,11*Z*)-*N*-Phenylheptadeca-8,11-diene-1-sulfinamide (7h)

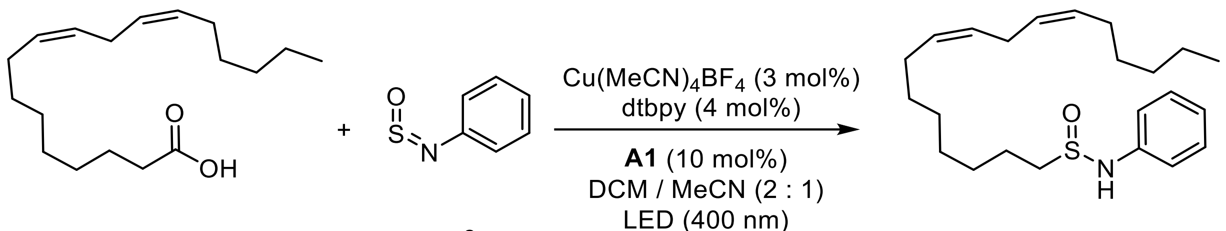

3 **7h**

According to GP2, the reaction was carried out with (9Z,12Z)-octadeca-9,12-dienoic acid (56 mg, 0.2 mmol), sulfinylamine 3 prepared from aniline according the GP1 (56 mg, 0.4 mmol, 2 equiv.), acridine **A1** (5.8 mg, 0.03 mmol, 10 mol%), $Cu(MeCN)_4BF_4$ (1.9 mg, 0.006 mmol, 3 mol%), dtbpy (2.2 mg, 0.008 mmol, 4 mol%) and DCM : MeCN (2 : 1, v/v, 2 mL). Argon was passed on the surface of the reaction for 10 seconds. The test-tube was capped and the reaction mixture was irradiated with LED light (λ = 400

nm) while stirring at 25−27 °C for 16 h. For work-up, a saturated solution of EDTA (1.5 mL) was added, followed by DCM (5 mL). The reaction mixture was then extracted with DCM (3 × 5 mL). The organic phases were combined, dried over anhydrous Na_2SO_4, concentrated under reduced pressure and the remaining material was purified by flash chromatography on silica gel (EtOAc/hexane, 4 : 6 v/v) to give product **7h** (42 mg, 56%) as a colorless liquid.

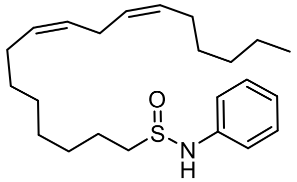

^{1}H NMR (500 MHz, $CDCl_3$): 7.26 (2 H, t, *J* = 7.9 Hz), 7.12–6.98 (4 H, m), 5.38 (4 H, qd, *J* = 10.7, 5.3 Hz), 3.00 (2 H, dq, *J* = 9.5, 4.6 Hz), 2.80 (2 H, t, *J* = 6.6 Hz), 2.07 (4 H, q, *J* = 7.0 Hz), 1.74 (2 H, p, *J* = 7.6 Hz), 1.52–1.20 (14 H, m), 0.91 (3 H, t, *J* = 6.8 Hz) ppm. – ^{13}C NMR (125 MHz, $CDCl_3$): 141.4, 130.3, 129.9, 129.4, 128.2, 127.9, 123.0, 118.3, 55.9, 31.5, 29.5, 29.4, 29.2, 29.0, 28.6, 27.22, 27.17, 25.7, 23.3, 22.6, 14.1 ppm. – IR: 2924, 2854, 1600, 1497, 1464, 1040, 889 cm^{-1}. – HRMS: calcd for $C_{23}H_{37}NOS$: 398.2488, found 398.2490 [M+Na$^+$].

_N_-Phenyltetracosa-9,11-diyne-1-sulfinamide (7i)

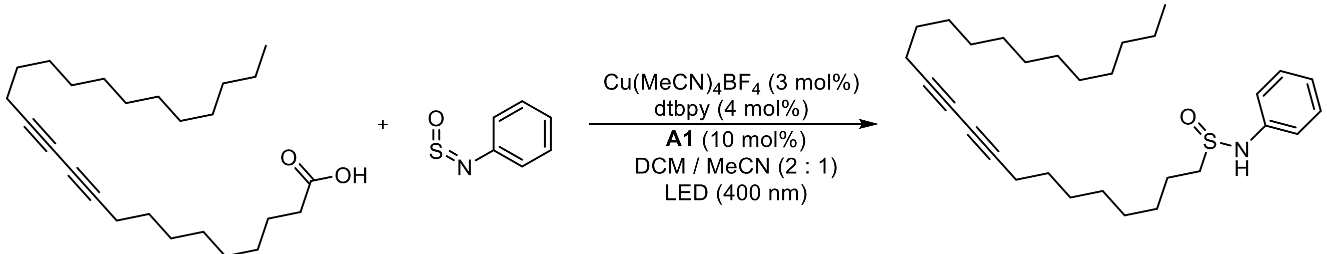

3 7i

According to GP2, the reaction was carried out with pentacosa-10,12-diynoic acid (75 mg, 0.2 mmol), sulfinylamine 3 prepared from aniline according the GP1 (56 mg, 0.4 mmol, 2 equiv.), acridine **A1** (5.8 mg, 0.03 mmol, 10 mol%), $Cu(MeCN)_4BF_4$ (1.9 mg, 0.006 mmol, 3 mol%), dtbpy (2.2 mg, 0.008 mmol, 4 mol%) and DCM : MeCN (2 : 1, v/v, 2 mL). Argon was passed on the surface of the reaction for 10 seconds. The test-tube was capped and the reaction mixture was irradiated with LED light (λ = 400 nm) while stirring at 25−27 °C for 16 h. For work-up, a saturated solution of EDTA (1.5 mL) was added, followed by DCM (5 mL). The reaction mixture was then extracted with DCM (3 × 5 mL). The organic phases were combined, dried over anhydrous Na_2SO_4, concentrated under reduced pressure and the remaining material was purified by flash chromatography on silica gel (EtOAc/hexane, 4 : 6 v/v) to give product **7i** (65 mg, 69%) as a colorless solid.

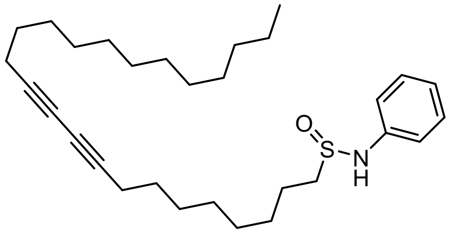

M.p.: 45–47 °C. − ^{1}H NMR (500 MHz, $CDCl_3$): 7.37–7.27 (3 H, m), 7.08 (2 H, d, *J* = 7.9 Hz), 6.25 (1 H, s), 2.97 (2 H, hept, *J* = 6.5 Hz), 2.26 (4 H, t, *J* = 7.5 Hz), 1.77 (2 H, p, *J* = 7.6 Hz), 1.62–1.17 (30 H, m), 0.90 (3 H, t, *J* = 6.8 Hz) ppm. – ^{13}C NMR (125 MHz, $CDCl_3$): 141.0, 129.5, 123.4, 118.8, 77.7, 77.4, 65.4, 65.2, 56.2, 31.9, 29.7, 29.6, 29.5, 29.4, 29.11, 29.05, 28.9, 28.8, 28.7, 28.6, 28.4, 28.2, 23.1, 22.7, 19.22, 19.18, 14.1 ppm. – IR: 2917, 2848, 1738,

Go to table of contents

1601, 1498, 1466, 1294, 1224, 1138, 1080, 1037 cm^{-1}. – HRMS: calcd for $C_{30}H_{47}NOS$: 470.3451, found 470.3450 [M+H$^+$].

1-((1*S*,3*R*)-3-Acetyl-2,2-dimethylcyclobutyl)-*N*-phenylmethanesulfinamide (7j)

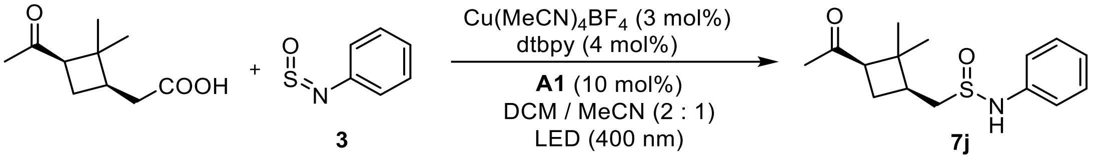

According to GP2, the reaction was carried out with *cis*-pinonic acid (37 mg, 0.2 mmol), sulfinylamine 3 prepared from aniline according the GP1 (70 mg, 0.5 mmol, 2.5 equiv.), acridine **A1** (5.8 mg, 0.03 mmol, 10 mol%), $Cu(MeCN)_4BF_4$ (1.9 mg, 0.006 mmol, 3 mol%), dtbpy (2.2 mg, 0.008 mmol, 4 mol%) and DCM : MeCN (2 : 1, v/v, 2 mL). Argon was passed on the surface of the reaction for 10 seconds. The test-tube was capped and the reaction mixture was irradiated with LED light (λ = 400 nm) while stirring at 25–27 °C for 16 h. For work-up, a saturated solution of EDTA (1.5 mL) was added, followed by DCM (5 mL). The reaction mixture was then extracted with DCM (3 × 5 mL). The organic phases were combined, dried over anhydrous Na_2SO_4, concentrated under reduced pressure and the remaining material was purified by flash chromatography on silica gel (EtOAc/hexane, 7 : 3 v/v) to give product **7j** (49 mg, 88%, 1 : 1 dr) as a colorless liquid.

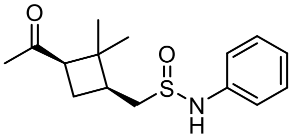

^{1}H NMR (500 MHz, $CDCl_3$): 7.25 (4 H, t, J = 7.7 Hz), 7.13–6.98 (6 H, m), 3.10–2.84 (8 H, m), 2.49–2.34 (2 H, m), 2.23–2.10 (2 H, m), 2.06 (3 H, s), 2.06 (3 H, s), 2.04–1.90 (2 H, m), 1.37 (3 H, s), 1.30 (3 H, s), 0.94 (3 H, s), 0.92 (3 H, s) ppm. – ^{13}C NMR (125 MHz, $CDCl_3$): 207.1, 207.0, 141.2, 129.5, 123.1, 118.3, 118.2, 57.2, 56.7, 54.1, 54.0, 43.6, 43.5, 36.4, 36.0, 30.3, 30.2, 30.1, 29.7, 23.0, 22.8, 17.8, 17.7 ppm. – IR: 3378, 3182, 2955, 1698, 1600, 1497, 1369, 1225, 1182, 1024, 750 cm^{-1}. – HRMS: calcd for $C_{15}H_{21}NO_2S$: 280.1366, found 280.1362 [M+H$^+$].

(6aS,6bR,8a*R*,10*S*,12a*R*,12b*R*,14b*S*)-10-hydroxy-2,2,6a,6b,9,9,12a-heptamethyl-*N*-phenyl-1,3,4,5,6,6a,6b,7,8,8a,9,10,11,12,12a,12b,13,14b-octadecahydropicene-4a(2*H*)-sulfinamide (7k)

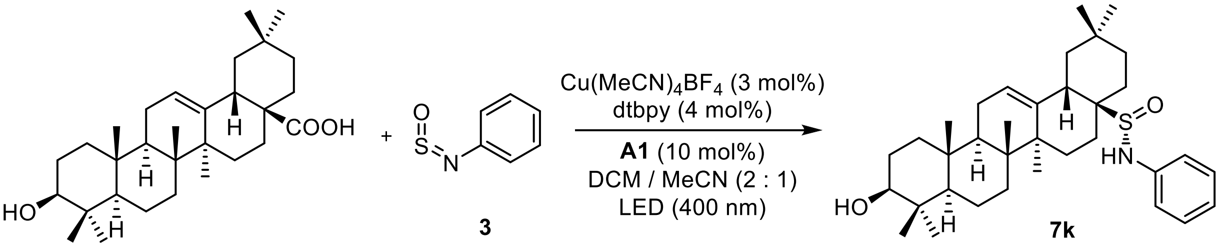

According to GP2, the reaction was carried out with oleanolic acid (91 mg, 0.2 mmol), sulfinylamine 3 prepared from aniline according the GP1 (70 mg, 0.5 mmol, 2.5 equiv.), acridine **A1** (5.8 mg, 0.03 mmol,

10 mol%), $Cu(MeCN)_4BF_4$ (1.9 mg, 0.006 mmol, 3 mol%), dtbpy (2.2 mg, 0.008 mmol, 4 mol%) and DCM : MeCN (2 : 1, v/v, 2 mL). Argon was passed on the surface of the reaction for 10 seconds. The test-tube was capped and the reaction mixture was irradiated with LED light (λ = 400 nm) while stirring at 25−27 °C for 16 h. For work-up, a saturated solution of EDTA (1.5 mL) was added, followed by DCM (5 mL). The reaction mixture was then extracted with DCM (3 × 5 mL). The organic phases were combined, dried over anhydrous Na_2SO_4, concentrated under reduced pressure and the remaining material was purified by flash chromatography on silica gel (EtOAc/hexane, 3 : 7 v/v) to give product **7k** (71 mg, 65%) as a colorless solid.

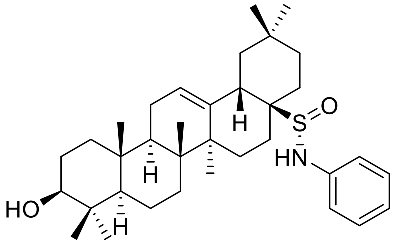

M.p.: 182–184°C. – ^1H NMR (500 MHz, $CDCl_3$): 7.33 – 7.25 (2 H, m), 7.05–6.96 (3 H, m), 5.48 (1 H, t, J = 3.7 Hz), 5.42 (1 H, s), 3.24 (1 H, dd, J = 10.9, 5.0 Hz), 2.85 (1 H, dd, J = 13.6, 4.7 Hz), 2.45 (1 H, td, J = 12.5, 4.9 Hz), 2.29 (1 H, td, J = 14.4, 4.6 Hz), 2.10–1.88 (3 H, m), 1.81 (1 H, t, J = 13.5 Hz), 1.73–1.21 (18 H, m), 1.11 (4 H, m), 1.07–0.92 (13 H, m), 0.82 (3 H, s) ppm. – ^{13}C NMR (125 MHz, $CDCl_3$): 13C NMR (126 MHz, CDCl3) δ 142.3, 140.8, 129.6, 125.4, 122.8, 118.0, 79.0, 64.7, 55.3, 47.6, 47.0, 41.7, 40.5, 40.2, 38.8, 38.6, 37.01, 36.98, 33.9, 33.0, 32.8, 30.9, 29.7, 28.1, 27.2, 25.7, 25.5, 23.7, 23.0, 20.1, 18.3, 17.3, 15.64, 15.56 ppm. – IR: 3361, 2943, 1599, 1495, 1366, 1276, 1029, 906, 875, 729 cm^{-1}. – HRMS: calcd for $C_{35}H_{53}NO_2S$: 574.3689, found 574.3695 [$M+Na^+$].

(3*S*,6a*R*,6b*S*,8a*S*,11*S*,12a*S*,14a*R*,14b*S*)-4,4,6a,6b,8a,11,14b-Heptamethyl-14-oxo-11-((phenylamino)sulfinyl)-1,2,3,4,4a,5,6,6a,6b,7,8,8a,9,10,11,12,12a,14,14a,14b-icosahydropicen-3-yl acetate (7l)

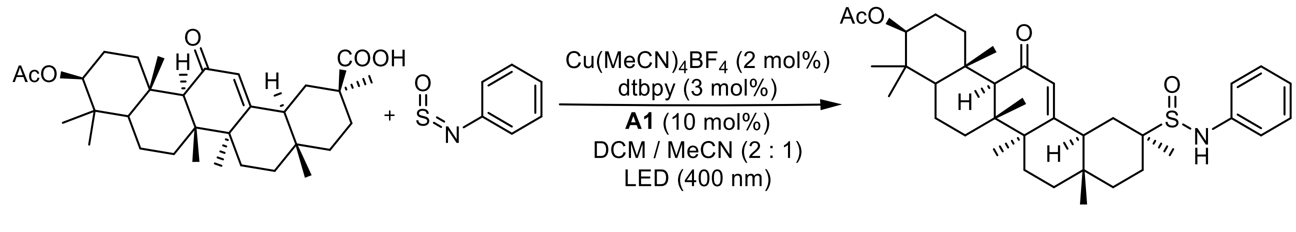

3 **7l**

According to GP2, the reaction was carried out with glycyrrhetinic acid (124 mg, 0.2 mmol), sulfinylamine 3 prepared from aniline according the GP1 (56 mg, 0.4 mmol, 2 equiv.), acridine **A1** (5.8 mg, 0.03 mmol, 10 mol%), $Cu(MeCN)_4BF_4$ (1.3 mg, 0.004 mmol, 2 mol%), dtbpy (1.6 mg, 0.006 mmol, 3 mol%) and DCM : MeCN (2 : 1, v/v, 2 mL). Argon was passed on the surface of the reaction for 10 seconds. The test-tube was capped and the reaction mixture was irradiated with LED light (λ = 400 nm) while stirring at 25–27 °C for 16 h. For work-up, a saturated solution of EDTA (1.5 mL) was added, followed by DCM (5 mL). The reaction mixture was then extracted with DCM (3 × 5 mL). The organic phases were combined, dried over anhydrous Na_2SO_4, concentrated under reduced pressure and the

remaining material was purified by flash chromatography on silica gel (EtOAc/hexane, 8 : 2 v/v) to give product **7l** (101 mg, 83%, 1 : 1 dr) as a colorless solid.

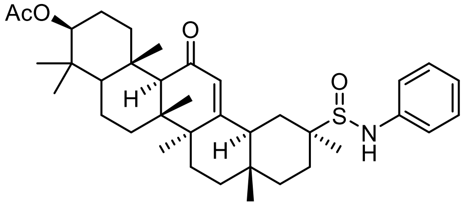

M.p.: 168–170 °C. – ^{1}H NMR (500 MHz, $CDCl_3$): 7.35–7.23 (2 H, m), 7.11–6.97 (3 H, m), 5.77–5.36 (2 H, m), 4.57–4.47 (1 H, m), 2.84–2.72 (1 H, m), 2.61–0.74 (43 H, m) ppm. – ^{13}C NMR (125 MHz, $CDCl_3$): 199.9, 199.83, 199.77, 199.6, 170.99, 170.96, 168.0, 167.7, 167.1, 142.0, 141.9, 129.7, 129.49, 129.46, 129.2, 128.9, 128.7, 123.5, 123.2, 123.1, 118.8, 118.7, 118.4, 118.2, 80.58, 80.55, 61.8, 61.73, 61.70, 60.83, 60.78, 60.2, 55.0, 54.9, 46.9, 46.6, 46.1, 45.5, 45.43, 45.39, 43.3, 43.2, 39.6, 39.1, 38.8, 38.74, 38.71, 38.0, 37.0, 36.9, 36.6, 35.7, 35.23, 35.18, 32.7, 32.6, 32.5, 32.3, 29.7, 29.5, 29.4, 28.2, 28.13, 28.06, 26.9, 26.7, 26.28, 26.25, 26.0, 24.3, 23.54, 23.50, 23.4, 21.3, 18.72, 18.67, 18.4, 18.3, 17.40, 17.36, 16.7, 16.41, 16.36, 14.3 ppm. – ^{13}C NMR (125 MHz, $CDCl_3$): ppm. – IR: 2924, 2873, 1729, 1656, 1599, 1497, 1464, 1371, 1240, 1142, 1045, 1028, 1001, 985 cm^{-1}. – HRMS: calcd for $C_{37}H_{53}NO_4S$: 608.3768, found 608.3764 [M+H$^+$].

(3*R*)-3-((3*R*,7*R*,8*R*,9*S*,10*S*,13*R*,14*S*,17*R*)-3,7-Dihydroxy-10,13-dimethylhexadecahydro-1*H*-cyclopenta[a]phenanthren-17-yl)-*N*-phenylbutane-1-sulfinamide (7m)

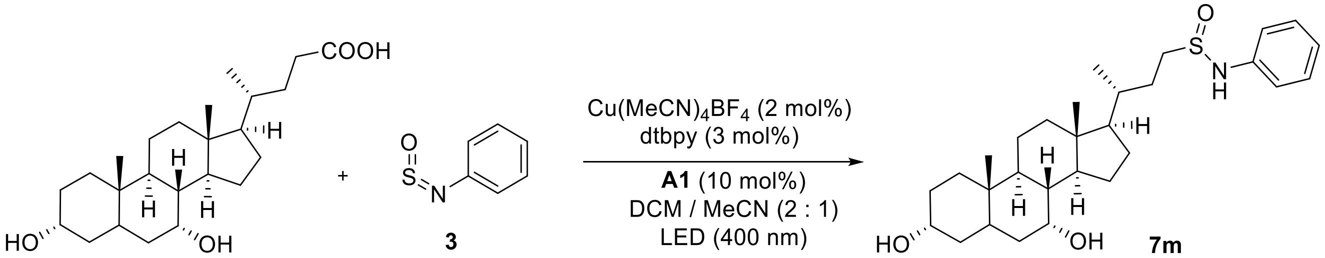

According to GP2, the reaction was carried out with chenodeoxycholic acid (78 mg, 0.2 mmol), sulfinylamine 3 prepared from aniline according the GP1 (56 mg, 0.4 mmol, 2 equiv.), acridine **A1** (5.8 mg, 0.03 mmol, 10 mol%), $Cu(MeCN)_4BF_4$ (1.3 mg, 0.004 mmol, 2 mol%), dtbpy (1.6 mg, 0.006 mmol, 3 mol%) and DCM : MeCN (2 : 1, v/v, 2 mL). Argon was passed on the surface of the reaction for 10 seconds. The test-tube was capped and the reaction mixture was irradiated with LED light (λ = 400 nm) while stirring at 25–27 °C for 16 h. For work-up, a saturated solution of EDTA (1.5 mL) was added, followed by DCM (5 mL). The reaction mixture was then extracted with DCM (3 × 5 mL). The organic phases were combined, dried over anhydrous Na_2SO_4, concentrated under reduced pressure and the remaining material was purified by flash chromatography on silica gel (EtOAc/hexane, 1 : 10 v/v) to give product **7m** (68 mg, 70%, 1 : 1 dr) as a colorless solid.

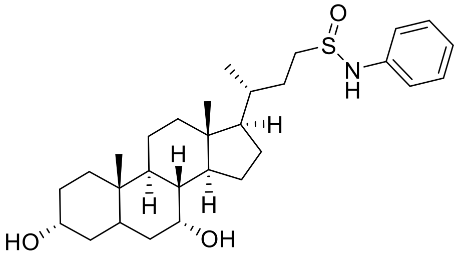

M.p.: 114–116 °C. – ^{1}H NMR (500 MHz, $CDCl_3$): 7.30–6.98 (5 H, m), 3.84 (1 H, s), 3.46 (1 H, dq, J = 10.8, 5.2 Hz), 3.17– 288 (2 H, m), 2.55 (2 H, s), 2.23 (1 H, q, J = 12.6 Hz), 2.04–1.08 (22 H, m), 1.05–0.84 (8 H, m), 0.67 (3 H, d, J = 5.6 Hz) ppm. – ^{13}C NMR (125 MHz, $CDCl_3$): 141.44, 141.39, 129.4, 123.01, 122.96, 118.40, 118.36, 71.9, 68.4, 55.8, 55.6, 53.0, 52.9, 50.4, 50.3, 42.7, 41.5, 39.8, 39.7, 39.62, 39.58, 39.43, 39.40, 35.4, 35.2, 35.1, 35.0, 34.7, 34.6, 32.8, 31.6, 30.7, 30.6, 29.7, 29.3, 29.0, 28.3, 23.7, 22.8, 22.7, 20.6, 18.6, 18.5, 14.1, 11.8 ppm. – IR: 2926, 2864, 1600, 1497, 1465, 1375, 1227, 1077, 1038, 1000, 978, 906 cm^{-1}. – HRMS: calcd for $C_{35}H_{53}NO_2S$: 574.3689, found 574.3695 [M+Na^+].

1-Cyclopropyl-*N*-phenylmethanesulfinamide (9) and *N*-phenylbut-3-ene-1-sulfinamide (9a)

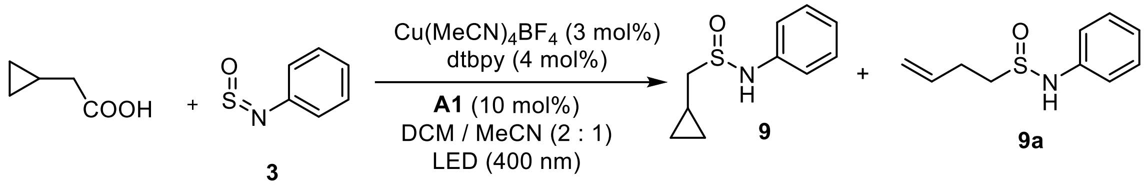

According to GP2, the reaction was carried out the reaction was carried out was followed with 2-cyclopropylacetic acid (20.0 mg, 0.2 mmol), sulfinylamine **3** (2, 2.5, 2.75, 3, 3.25, and 3.75 equiv.), acridine **A1** (5.8 mg, 0.03 mmol, 10 mol%), $Cu(MeCN)_4BF_4$ (1.9 mg, 0.006 mmol, 3 mol%), dtbpy (2.2 mg, 0.008 mmol, 4 mol%) and dichloromethane : MeCN (2 : 1, v/v, 2 mL). Argon was passed on the surface of the reaction for 10 seconds. The test-tube was capped and the reaction mixture was irradiated with LED light (λ = 400 nm) while stirring at 25–27 °C for 8 h. For work-up, a saturated solution of EDTA (1.5 mL) was added, followed by dichloromethane (5 mL). The reaction mixture was then extracted with dichloromethane (3 × 5 mL). The organic phases were combined, dried over anhydrous Na_2SO_4, concentrated under reduced pressure and the remaining material was purified by flash chromatography on silica gel (EtOAc/hexane, 4 : 6 v/v) to give an inseparable mixture of products **9** and **9a** (17.7 mg, 45%) as a colorless liquid.

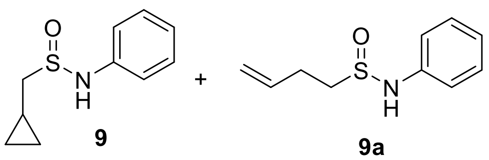

9: ^{1}H NMR (500 MHz, $CDCl_3$) δ 7.36 (1 H brs), 7.26-7.22 (5 H m), 3.02 (1 H, dd, J = 13.4, 7.2 Hz), 2.90 (1 H, dd, J = 13.4, 7.6 Hz), 1.14-1.06 (1 H, m), 0.74-0.65 (2 H, m), 0.42 – 0.34 (2 H, m) ppm. **9a**: ^{1}H NMR (500 MHz, $CDCl_3$) δ 7.43 (1 H, brs), 7.13 – 6.96 (5 H, m), 5.83 (1 H, ddt, J = 16.8, 10.2, 6.5 Hz), 5.29 – 5.03 (2 H, m), 3.11 (2 H, t, J = 7.5 Hz), 2.50 (2 H, dt, J = 8.8, 6.8 Hz) ppm. – ^{13}C NMR of 9 and 9a mixture (125 MHz, $CDCl_3$): δ 141.6, 141.4, 134.7, 129.4, 122.9, 122.9, 118.2, 118.2, 117.1, 61.1, 54.7, 27.6, 5.2, 4.8, 4.7 ppm. – IR: 3418, 3152, 3079, 2884, 1640, 1599, 1496, 1404, 1281, 1043, 886, 749 cm^{-1}.

N-(4-chlorophenethyl)-2-methylpropane-1-sulfinamide (12a)

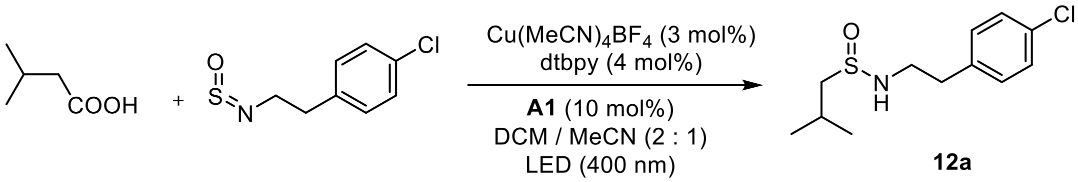

According to GP2, the reaction was carried out the reaction was carried out was followed with 3-methylbutanoic acid (20.4 mg, 0.2 mmol), ((4-chlorophenethyl)imino)-λ^4-sulfanone (70 mg, 0.4 mmol, 2.0 equiv.) prepared from 2-(4-chlorophenyl)ethylamine according to GP1, acridine **A1** (5.8 mg, 0.03 mmol, 10 mol%), $Cu(MeCN)_4BF_4$ (1.9 mg, 0.006 mmol, 3 mol%), dtbpy (2.2 mg, 0.008 mmol, 4 mol%) and dichloromethane : MeCN (2 : 1, v/v, 2 mL). Argon was passed on the surface of the reaction for 10 seconds. The test-tube was capped and the reaction mixture was irradiated with LED light (λ = 400 nm) while stirring at 25–27 °C for 14 h. For work-up, a saturated solution of EDTA (1.5 mL) was added, followed by dichloromethane (5 mL). The reaction mixture was then extracted with dichloromethane (3 × 5 mL). The organic phases were combined, dried over anhydrous Na_2SO_4, concentrated under reduced pressure and the remaining material was purified by flash chromatography on silica gel (EtOAc/hexane, 4 : 6 v/v) to give product **12a** (38.4 mg, 74%) as a colorless liquid.

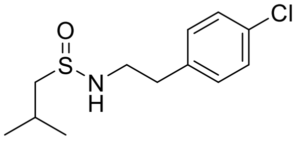

^{1}H NMR (500 MHz, $CDCl_3$) δ 7.29 (2 H, d, J = 8.4 Hz), 7.16 (2 H, d, J = 8.4 Hz), 3.75 (1 H, t, J = 6.4 Hz), 3.39 (2 H, qd, J = 6.8, 1.9 Hz), 2.88 (2 H, td, J = 7.0, 4.2 Hz), 2.71 – 2.47 (2 H, m), 2.03 (1 H, ddq, J = 13.3, 7.9, 6.6 Hz), 1.04 (6 H, t, J = 7.2 Hz) ppm. – ^{13}C NMR (125 MHz, $CDCl_3$): δ 136.9, 132.5, 130.3, 128.8, 64.6, 44.4, 36.5, 24.5, 22.4, 21 ppm. – IR: 3408, 3018, 2917, 1584, 1514, 1463, 1442, 1425, 1306, 808, 748 cm^{-1}. – HRMS: calcd for $C_{12}H_{18}ClNOS$: 260.0870, found 260.0965 [M+H$^+$].

N-(3,5-difluorophenyl)-2-methylpropane-1-sulfinamide (12b)

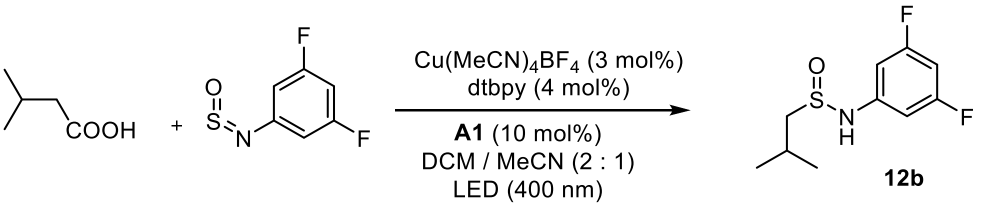

According to GP2, the reaction was carried out the reaction was carried out was followed with 3-methylbutanoic acid (20.4 mg, 0.2 mmol), ((3,5-difluorophenyl)imino)-λ^4-sulfanone (70 mg, 0.4 mmol, 2.0 equiv.) prepared from 3,5-difluoroaniline according to GP1, acridine **A1** (5.8 mg, 0.03 mmol, 10 mol%), $Cu(MeCN)_4BF_4$ (1.9 mg, 0.006 mmol, 3 mol%), dtbpy (2.2 mg, 0.008 mmol, 4 mol%) and DCM : MeCN (2 : 1, v/v, 2 mL). Argon was passed on the surface of the reaction for 10 seconds. The test-tube was capped and the reaction mixture was irradiated with LED light (λ = 400 nm) while stirring at 25–27 °C for 14 h. For work-up, a saturated solution of EDTA (1.5 mL) was added, followed by DCM

(5 mL). The reaction mixture was then extracted with DCM (3 × 5 mL). The organic phases were combined, dried over anhydrous Na_2SO_4, concentrated under reduced pressure and the remaining material was purified by flash chromatography on silica gel (EtOAc/hexane, 7 : 3 v/v) to give product **12b** (41 mg, 88%) as a colorless liquid.

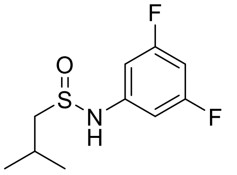

^{1}H NMR (500 MHz, $CDCl_3$) δ 8.10 (1 H, s), 6.54 (2 H, dd, J = 8.0, 2.2 Hz), 6.40 (1 H, tt, J = 9.1, 2.5 Hz), 3.00 (1 H, dd, J = 12.9, 6.2 Hz), 2.87 (1 H, dd, J = 12.9, 8.2 Hz), 2.14 (1 H, dp, J = 13.6, 6.8 Hz), 1.08 (6 H, dd, J = 30.1, 6.7 Hz) ppm. – ^{13}C NMR (125 MHz, $CDCl_3$): δ 164.7 (d, J = 14.9 Hz), 162.7 (d, J = 14.9 Hz), 144.3 (t, J = 12.7 Hz), 100.2 (d, J = 29.4 Hz), 97.7 (t, J = 25.7 Hz), 64.3, 24.6, 22.2, 21.7 ppm. – ^{19}F NMR (471 MHz, $CDCl_3$) δ -108.47 (t, J = 8.4 Hz). – IR: 3196, 2962, 2870, 1627, 1600, 1505, 1412, 1395, 1111, 1045, 1022, 991, 828, 802, 728 cm^{-1}. – HRMS: calcd for $C_{10}H_{13}F_2NOS$: 234.0759, found 234.0753 [M+H$^+$].

_N_-(4-fluorophenethyl)ethanesulfinamide (12c)

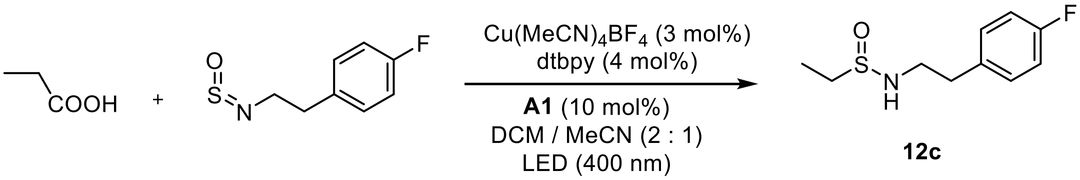

According to GP2, the reaction was carried out the reaction was carried out was followed with propionic acid (14.8 mg, 0.2 mmol), ((4-fluorophenethyl)imino)-λ^4-sulfanone (74 mg, 0.4 mmol, 2.0 equiv.) pepared from 2-(4-fluorophenyl)ethylamine according to GP1, acridine **A1** (5.8 mg, 0.03 mmol, 10 mol%), $Cu(MeCN)_4BF_4$ (1.9 mg, 0.006 mmol, 3 mol%), dtbpy (2.2 mg, 0.008 mmol, 4 mol%) and dichloromethane : MeCN (2 : 1, v/v, 2 mL). Argon was passed on the surface of the reaction for 10 seconds. The test-tube was capped and the reaction mixture was irradiated with LED light (λ = 400 nm) while stirring at 25–27 °C for 14 h. For work-up, a saturated solution of EDTA (1.5 mL) was added, followed by dichloromethane (5 mL). The reaction mixture was then extracted with dichloromethane (3 × 5 mL). The organic phases were combined, dried over anhydrous Na_2SO_4, concentrated under reduced pressure and the remaining material was purified by flash chromatography on silica gel (EtOAc/hexane, 4 : 6 v/v) to give product **12c** (30.1 mg, 70%) as a colorless liquid.

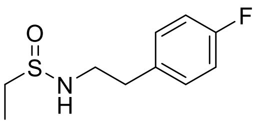

^{1}H NMR (500 MHz, $CDCl_3$) δ 7.19 (2 H, dd, J = 8.4, 5.5 Hz), 7.03-7.00 (2 H, m), 3.68 (1 H, brs), 3.39 (2 H, q, J = 6.8 Hz), 2.89 (2 H, td, J = 7.0, 5.0 Hz), 2.73 (2 H, qd, J = 7.5, 4.5 Hz), 1.22 (3 H, t, J = 7.5 Hz) ppm. – ^{13}C NMR (125 MHz, $CDCl_3$): δ 162.7, 160.8, 134.1, 134.0, 130.3 (d, J = 8.0 Hz), 115.5 (d, J = 21.2 Hz), 49.1, 44.4, 36.4, 7.5 ppm. – ^{19}F NMR (471MHz, $CDCl_3$): δ -116.4 ppm – IR: 3250, 3188, 2935, 1662, 1455, 1379, 1220, 1099, 1046, 900 cm^{-1}.

Methyl 3-((isobutylsulfinyl)amino)thiophene-2-carboxylate (12d)

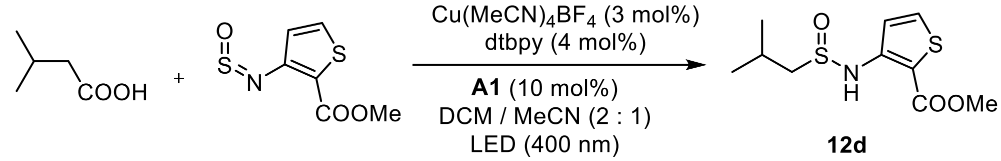

12d

According to GP2, the reaction was carried out the reaction was carried out was followed with 3-methylbutanoic acid (20.4 mg, 0.2 mmol), methyl 3-((oxo-λ^4-sulfaneylidene)amino)thiophene-2-carboxylate prepared from methyl 3-aminothiophene-2-carboxylate according to GP1 (81.2 mg, 0.4 mmol, 2.0 equiv.), acridine **A1** (5.8 mg, 0.03 mmol, 10 mol%), $Cu(MeCN)_4BF_4$ (1.9 mg, 0.006 mmol, 3 mol%), dtbpy (2.2 mg, 0.008 mmol, 4 mol%) and dichloromethane : MeCN (2 : 1, v/v, 2 mL). Argon was passed on the surface of the reaction for 10 seconds. The test-tube was capped and the reaction mixture was irradiated with LED light (λ = 400 nm) while stirring at 25–27 °C for 14 h. For work-up, a saturated solution of EDTA (1.5 mL) was added, followed by dichloromethane (5 mL). The reaction mixture was then extracted with dichloromethane (3 × 5 mL). The organic phases were combined, dried over anhydrous Na_2SO_4, concentrated under reduced pressure and the remaining material was purified by flash chromatography on silica gel (EtOAc/hexane, 7 : 3 v/v) to give product **12d** (47 mg, 90%) as pale yellow liquid.

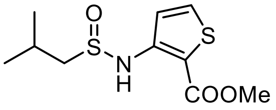

^{1}H NMR (500 MHz, $CDCl_3$) δ 8.89 (1 H, brs), 7.46 (1 H, d, *J* = 5.4 Hz), 7.16 (1 H, d, *J* = 5.4 Hz), 3.87 (3 H, s), 2.97 – 2.87 (2 H, m), 2.27 – 2.16 (1 H, m), 1.13 (6 H, t, *J* = 7.1 Hz) ppm. – ^{13}C NMR (125 MHz, $CDCl_3$): δ 164.7, 148.8, 132.3, 118.3, 107.9, 65.4, 51.9, 24.4, 22.5, 21.9. ppm. – IR: 3364, 2558, 1679, 1609, 1542, 1456, 1308, 1272, 1191, 1086, 1038 cm^{-1}. – HRMS: calcd for $C_{10}H_{15}NO_3S_2$: 262.0566, found 262.0565 [M+H$^+$].

***N*-(3-bromo-4-methylphenyl)ethanesulfinamide (12e)**

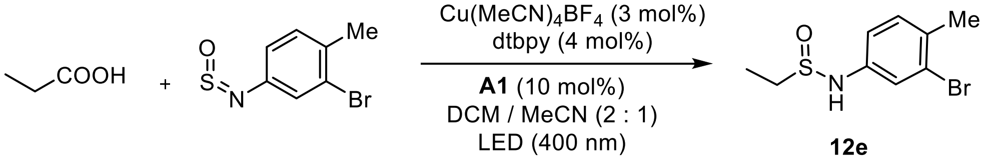

12e

According to GP2, the reaction was carried out the reaction was carried out was followed with propionic acid (14.8 mg, 0.2 mmol), ((3-bromo-4-methylphenyl)imino)-λ^4-sulfanone (92.4 mg, 0.4 mmol, 2.0 equiv.) prepared from 3-bromo-4-methylaniline according to GP1, acridine **A1** (5.8 mg, 0.03 mmol, 10 mol%), $Cu(MeCN)_4BF_4$ (1.9 mg, 0.006 mmol, 3 mol%), dtbpy (2.2 mg, 0.008 mmol, 4 mol%) and DCM : MeCN (2 : 1, v/v, 2 mL). Argon was passed on the surface of the reaction for 10 seconds. The test-tube was capped and the reaction mixture was irradiated with LED light (λ = 400 nm) while stirring at 25–27 °C for 14 h. For work-up, a saturated solution of EDTA (1.5 mL) was added, followed by DCM (5 mL). The reaction mixture was then extracted with DCM (3 × 5 mL). The organic phases were combined, dried over anhydrous Na_2SO_4, concentrated under reduced pressure and the remaining

material was purified by flash chromatography on silica gel (EtOAc/hexane, 3 : 7 v/v) to give product **12e** (46 mg, 88%) as a colorless liquid.

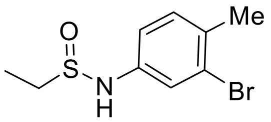

^{1}H NMR (500 MHz, $CDCl_3$) δ δ 7.32 (1 H, s), 7.22 (1 H, d, *J* = 2.4 Hz), 7.06 (1 H, d, *J* = 8.2 Hz), 6.88 (1 H, dd, *J* = 8.1, 2.4 Hz,), 3.27 – 2.75 (2 H , m), 2.32 (3 H, s), 1.32 (3 H, t, *J* = 7.5 Hz) ppm. – ^{13}C NMR (125 MHz, $CDCl_3$): δ 140.4, 132.2, 131.2, 125.2, 121.9, 117.1, 49.5, 22.0, 7.7 ppm. – IR: 3157, 2918, 1605, 1570, 1492, 1380, 1232, 1206, 1061, 1033, 907, 812, 764 cm^{-1}.

Computational data

Software

Quantum chemical calculations were performed using the Lonestar6 supercomputer at the Texas Advanced Computing Center (TACC) hosted by the University of Texas in Austin, Texas, and Bridges-2 supercomputer hosted by the Pittsburgh Supercomputing Center (PSC) and supported by Advanced Cyberinfrastructure Coordination Ecosystem: Services & Support (ACCESS) program. DFT geometry optimization, vibrational frequency, and IRC calculations were conducted using Gaussian 16 (rA.03).[5] The CREST utility of the xTB software suite[6] was used to locate initial starting geometries for optimization via DFT. Energy decomposition analysis was performed using the Absolutely Localized Molecular Orbital Energy Decomposition Analysis (ALMO-EDA2) and Complementary Occupied-Virtual orbital Pairs (COVP) methods as implemented in Q-Chem 5.3.1.[7] Final images of minima and transition state structure geometries were rendered using CYLview (v1.0.600)[8] and VMD (v1.9.4a40).[9] Molecular orbital energies were obtained using Multiwfn 3.8(dev).[10] Routine visualization and monitoring of calculations was performed with Chemcraft (v1.8-622b).[11]

Details of Computational Methods

Gaussian 16 DFT calculations

Geometries of ground state minima and transition state structures were optimized without constraints using MN15[12] density functional approximation and the def2-TZVP[13] basis set in dichloromethane solvent using the SMD solvation model.[14] Calculations were set to “tight” convergence criteria with an ultrafine grid. Frequency calculations at the same level of theory were used to confirm the nature of the isolated stationary points. Geometries with zero imaginary frequencies were deemed minima whereas those with exactly one imaginary frequency along the chemical path of interest were deemed transition state structures. Intrinsic reaction coordinate (IRC) calculations were performed to further corroborate that the located transition state structures connected reactants to products. The quasi-harmonic approximation at 1M concentration was applied via GoodVibes[15] to all structures to correct for potential errors associated with low magnitude vibrational frequencies using a cut-off frequency of 50 cm^{-1}. Single point corrections of the above geometries were calculated using PW6B95[16]-D3(BJ)[17] in dichloromethane solvent with the SMD solvation model**.** The def2-TZVPPD[18] basis set was used by appending diffuse functions obtained from the EMSL BSE[19] to the G16-available def2-TZVPP basis set. This level of theory provided the final electronic component to the reported free energies.

Distortion/Interaction-Activation Strain Analysis of TS3

Distortion/interaction-activation strain analysis[20] was performed on **TSA** and **TSB** at the MN15 / def2-TZVP / SMD (DCM) level of theory. Guided by previous work with similar systems,[21] fragment

definitions were created for **TSA** and **TSB**, with the red fragment representing the alkyl radical component and the green fragment representing the sulfinylamine:

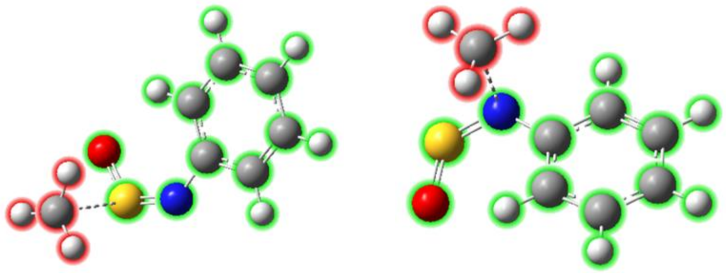

Figure S2. Division of **TSA** and **TSB** into fragments for distortion/interaction-activation strain analysis.

Energy Decomposition Analysis

The second generation Absolutely Localized Molecular Orbital Energy Decomposition Analysis (ALMO-EDA2) method[22] was employed to gain quantitative insight into the intermolecular forces governing the interaction energy of **TSA** and **TSB**. The results of the ALMO-EDA2 studies are summarized in Table S2.

Table S2. Summary table for ALMO-EDA2.[a]

Structure	Prep	ΔE_{Pauli}	ΔE_{Disp}	ΔE_{Elec}	ΔE_{CT}	ΔE_{Pol}	ΔE_{Sol}	Total ΔE_{int}
**TSA**	0.0	29.3	-6.0	-14.3	-10.5	-1.2	-0.8	-3.6
**TSB**	0.0	51.8	-10.2	-22.1	-14.3	-2.2	-1.0	2.1

[a] ΔE reported in kcal, mol.

Boltzmann Ensemble Averaging

To improve the accuracy of the DFT computational analysis of the reaction pathway, ensemble averaging was applied for the obtained structurally distinct conformers of intermediates **10** and **11**, as previously described.[23]

Reaction efficiency prediction

Data collection

The reaction efficiency data were collected for reactions with carboxylic acids and sulfinylamines that were conducted as described in GP2, and the yields were determined using 1,4-dimethoxybenzene as an internal standard. The alkyl and sulfinylamine fragments and the normalized yields for the reactions between the corresponding carboxylic acids and sulfinylamines are reported in Table S3. All yields were z-score normalized as previously described.[24]

Table S3. Experimental reaction efficiency data for the decarboxylative sulfinamidation.

Alkyl radical	Alkyl radical SMILES	Sulfinylamine fragment	Sulfinylamine fragment SMILES	Normalized yield[α]
a8	CC[C]1COC1	b1	O=[S][N]C1=CC(N=CS2)=C2C=C1	-0.71
a23	[C]CCCCCCCC	b1	O=[S][N]C1=CC(N=CS2)=C2C=C1	-0.50
a49	[C]1CCOCC1	b1	O=[S][N]C1=CC(N=CS2)=C2C=C1	-0.55
a2	[C]1CCCCCC1	b9	O=[S][N]CCC1=CC=C(Cl)C=C1	0.40
a34	CCC[C]CCC	b9	O=[S][N]CCC1=CC=C(Cl)C=C1	0.71
a35	CCCCCC[C]CCCCCCCC	b9	O=[S][N]CCC1=CC=C(Cl)C=C1	0.61
a2	[C]1CCCCCC1	b10	O=[S][N]C1=C(F)C=C(F)C(F)=C1	1.51
a21	C[C]1COC1	b10	O=[S][N]C1=C(F)C=C(F)C(F)=C1	0.56
a22	C[C]1CCC1	b10	O=[S][N]C1=C(F)C=C(F)C(F)=C1	0.98
a26	[C]CCNC(C)=O	b10	O=[S][N]C1=C(F)C=C(F)C(F)=C1	-0.82
a33	[C]CCCCCC/C=C\CCCCCCCC	b10	O=[S][N]C1=C(F)C=C(F)C(F)=C1	0.03
a48	CC(OC(N1CC[C]CC1)=O)(C)C	b10	O=[S][N]C1=C(F)C=C(F)C(F)=C1	0.03
a49	[C]1CCOCC2	b10	O=[S][N]C1=C(F)C=C(F)C(F)=C1	1.19
a1	[C]1CCCCC1	b11	O=[S][N]C1=CC=CN=C1	1.24
a14	C[C]1CC(OC)(OC)C1	b11	O=[S][N]C1=CC=CN=C1	0.24
a4	[C@H]1(C[C]2C3)C[C@@H](C2)C[C@@H]3C1	b11	O=[S][N]C1=CC=CN=C1	0.19
a40	[C][C@H]1C[C@@H](C(C)=O)C(C)1C	b11	O=[S][N]C1=CC=CN=C1	0.45
a48	CC(OC(N1CC[C]CC1)=O)(C)C	b11	O=[S][N]C1=CC=CN=C1	-0.40
a17	FC1(F)CC[C]CC1	b12	O=[S][N]C1=NC=C(Br)C=N1	0.29
a20	[C]CCC(C1=CC=CC=C1)=O	b12	O=[S][N]C1=NC=C(Br)C=N1	-0.92
a25	[C]CCCCCl	b12	O=[S][N]C1=NC=C(Br)C=N1	-0.03
a32	[C]CCCBr	b12	O=[S][N]C1=NC=C(Br)C=N1	-0.34
a51	[C]C1=CC=C(I)C=C1	b12	O=[S][N]C1=NC=C(Br)C=N1	-1.03
a52	C[C]1CCC(CC1)=O	b12	O=[S][N]C1=NC=C(Br)C=N1	-0.66
a1	[C]1CCCCC1	b13	[NH][S]=O	-1.08
a9	O=C1[C@H](C2)C[C@H]3C[C@@H]1C[C@@]2C3	b13	[NH][S]=O	-0.13
a10	C[C](C)CCCOC1=C(C)C=CC(C)=C1	b13	[NH][S]=O	-0.03
a2	[C]1CCCCCC1	b13	[NH][S]=O	-0.34
a16	O=C(C12CC[C](CC2)CC1)OC	b13	[NH][S]=O	-0.45
a23	[C]CCCCCCCC	b13	[NH][S]=O	0.98
a25	[C]CCCCCl	b13	[NH][S]=O	0.93
a34	CCC[C]CCC	b13	[NH][S]=O	-0.29
a1	[C]1CCCCC1	b14	O=[S][N]C1=CC=CC=C1	1.35
a10	C[C](C)CCCOC1=C(C)C=CC(C)=C1	b14	O=[S][N]C1=CC=CC=C1	1.51
a11	O=C1C2=C(C=CC([C])=C2)OCC3=CC=CC=C31	b14	O=[S][N]C1=CC=CC=C1	-0.82
a12	[C]CC1=CC=C(OC)C(OC)=C1	b14	O=[S][N]C1=CC=CC=C1	-0.82
a2	[C]1CCCCCC1	b14	O=[S][N]C1=CC=CC=C1	1.51
a16	O=C(C12CC[C](CC2)CC1)OC	b14	O=[S][N]C1=CC=CC=C1	1.24
a17	FC1(F)CC[C]CC1	b14	O=[S][N]C1=CC=CC=C1	1.35
a20	[C]CCC(C1=CC=CC=C1)=O	b14	O=[S][N]C1=CC=CC=C1	-0.29
a23	[C]CCCCCCCC	b14	O=[S][N]C1=CC=CC=C1	1.14
a25	[C]CCCCCl	b14	O=[S][N]C1=CC=CC=C1	1.45
a26	[C]CCNC(C)=O	b14	O=[S][N]C1=CC=CC=C1	0.03
a4	[C@H]1(C[C]2C3)C[C@@H](C2)C[C@@H]3C1	b14	O=[S][N]C1=CC=CC=C1	0.87
a29	[C]CCCCCC/C=C\C/C=C\CCCCC	b14	O=[S][N]C1=CC=CC=C1	-0.76
a30	[C]C(NC(OC(C)(C)C)=O)C(OC(C)(C)C)=O	b14	O=[S][N]C1=CC=CC=C1	-1.03
a31	[C]CCCCCCCCCC/C=C\CCCCCCCC	b14	O=[S][N]C1=CC=CC=C1	-0.50
a36	[C]CCCCBr	b14	O=[S][N]C1=CC=CC=C1	0.29
a37	[C]CC(NC(OC(C)(C)C)=O)C(OCC1=CC=CC=C1)=O	b14	O=[S][N]C1=CC=CC=C1	-1.08

a39	[C]CCC1=CC=CS1	b14	O=[S][N]C1=CC=CC=C1	0.45
a40	[C][C@H]1C[C@@H](C(C)=O)C(C)1C	b14	O=[S][N]C1=CC=CC=C1	0.93
a41	[C]CCCN1C(C=CC(Br)=C2)=C2C=C1	b14	O=[S][N]C1=CC=CC=C1	-1.40
a42	COC1=C(C(O)=C2C(OCC2=C1C)=O)C/C=C(C[C])\C	b14	O=[S][N]C1=CC=CC=C1	-0.03
a45	C1(C=CC=C2)=C2C[C]C1	b14	O=[S][N]C1=CC=CC=C1	0.19
a46	C[C](CC1)CCN1C(OC(C)(C)C)=O	b14	O=[S][N]C1=CC=CC=C1	0.82
a47	CC(OC(N1[C]CCC1)=O)(C)C	b14	O=[S][N]C1=CC=CC=C1	0.50
a6	[C]CC1=CC=C(F)C=C1	b14	O=[S][N]C1=CC=CC=C1	-0.18
a49	[C]1CCOCC4	b14	O=[S][N]C1=CC=CC=C1	1.51
a2	[C]1CCCCCC1	b15	O=[S][N]C1=CC(C)=CC(Cl)=C1	1.24
a36	[C]CCCCBr	b15	O=[S][N]C1=CC(C)=CC(Cl)=C1	-0.66
a39	[C]CCC1=CC=CS1	b15	O=[S][N]C1=CC(C)=CC(Cl)=C1	-1.61
a44	O=C(N1CC[C]CC1)C	b15	O=[S][N]C1=CC(C)=CC(Cl)=C1	-0.13
a9	O=C1[C@H](C2)C[C@H]3C[C@@H]1C[C@@]2C5	b16	O=[S][N]C1=CC=C(C(F)(F)F)C=C1	0.29
a32	[C]CCCBr	b16	O=[S][N]C1=CC=C(C(F)(F)F)C=C1	0.03
a16	O=C(C12CC[C](CC2)CC1)OC	b17	O=[S][N]C1=C(Cl)C=CC=C1	-0.24
a19	C[C@@]12C[C@H](C3)C[C@@](C[C@]3C2)(C)C1	b17	O=[S][N]C1=C(Cl)C=CC=C1	0.13
a27	[C]CCCCl	b17	O=[S][N]C1=C(Cl)C=CC=C1	-0.03
a28	[C]CCCCCCCCCCCCCCCC	b17	O=[S][N]C1=C(Cl)C=CC=C1	0.29
a48	CC(OC(N1CC[C]CC1)=O)(C)C	b17	O=[S][N]C1=C(Cl)C=CC=C1	0.13
a6	[C]CC1=CC=C(F)C=C1	b17	O=[S][N]C1=C(Cl)C=CC=C1	-0.92
a9	O=C1[C@H](C2)C[C@H]3C[C@@H]1C[C@@]2C6	b2	O=[S][N]C1CCCCC1	-1.82
a38	[C]CCCC1=CC=CC=C1	b2	O=[S][N]C1CCCCC1	-1.82
a14	C[C]1CC(OC)(OC)C1	b3	CC1=C(C(OCC)=O)SC([N][S]=O)=C1C(OCC)=O	1.03
a18	O=C(C12C[C](C2)C1)OC	b3	CC1=C(C(OCC)=O)SC([N][S]=O)=C1C(OCC)=O	0.19
a5	[C]CC1=C(F)C=CC(Br)=C1	b3	CC1=C(C(OCC)=O)SC([N][S]=O)=C1C(OCC)=O	0.29
a40	[C][C@H]1C[C@@H](C(C)=O)C(C)1C	b3	CC1=C(C(OCC)=O)SC([N][S]=O)=C1C(OCC)=O	1.14
a15	[C]CCCC1=CC=CC=C1	b4	O=[S][N]C1=CC=CC(Br)=C1	-1.82
a24	[C]CCCCCCCCCCCC	b4	O=[S][N]C1=CC=CC(Br)=C1	-2.30
a35	CCCCCC[C]CCCCCCCC	b4	O=[S][N]C1=CC=CC(Br)=C1	-2.72
a43	[C]C1=CC=CC=C1I	b4	O=[S][N]C1=CC=CC(Br)=C1	-2.30
a6	[C]CC1=CC=C(F)C=C1	b4	O=[S][N]C1=CC=CC(Br)=C1	0.71
a49	[C]1CCOCC1	b4	O=[S][N]C1=CC=CC(Br)=C1	-1.19
a13	C[C]1CCCCC1	b5	CC1=CC=CC([N][S]=O)=N1	0.56
a20	[C]CCC(C1=CC=CC=C1)=O	b5	CC1=CC=CC([N][S]=O)=N1	0.03
a3	[C]1CCC1	b5	CC1=CC=CC([N][S]=O)=N1	0.82
a39	[C]C1CCCC1	b5	CC1=CC=CC([N][S]=O)=N1	1.03
a10	C[C](C)CCCOC1=C(C)C=CC(C)=C1	b6	CC([N][S]=O)C1=CC=CC=C1	-0.76
a2	[C]1CCCCCC1	b6	CC([N][S]=O)C1=CC=CC=C1	1.03
a34	CCC[C]CCC	b6	CC([N][S]=O)C1=CC=CC=C1	0.29
a35	CCCCCC[C]CCCCCCCC	b6	CC([N][S]=O)C1=CC=CC=C1	1.19
a1	[C]1CCCCC1	b7	O=[S][N]C1=CC=C(C#N)C=C1	-2.30
a16	O=C(C12CC[C](CC2)CC1)OC	b7	O=[S][N]C1=CC=C(C#N)C=C1	0.56
a17	FC1(F)CC[C]CC1	b7	O=[S][N]C1=CC=C(C#N)C=C1	1.40
a4	[C@H]1(C[C]2C3)C[C@@H](C2)C[C@@H]3C1	b7	O=[S][N]C1=CC=C(C#N)C=C1	0.71
a32	[C]CCCBr	b7	O=[S][N]C1=CC=C(C#N)C=C1	0.71
a45	C1(C=CC=C2)=C2C[C]C1	b7	O=[S][N]C1=CC=C(C#N)C=C1	1.24
a6	[C]CC1=CC=C(F)C=C1	b7	O=[S][N]C1=CC=C(C#N)C=C1	1.08
a11	O=C1C2=C(C=CC([C])=C2)OCC3=CC=CC=C31	b8	O=[S][N]C1=CC=CC(OC(F)(F)F)=C1	1.56
a2	[C]1CCCCCC1	b8	O=[S][N]C1=CC=CC(OC(F)(F)F)=C1	-1.77
a30	[C]C(NC(OC(C)(C)C)=O)C(OC(C)(C)C)=O	b8	O=[S][N]C1=CC=CC(OC(F)(F)F)=C1	-0.66

a34	CCC[C]CCC	b8	O=[S][N]C1=CC=CC(OC(F)(F)F)=C1	-0.92
a39	[C]C1CCCC1	b8	O=[S][N]C1=CC=CC(OC(F)(F)F)=C1	-0.76
a50	C[C]1CCOCC1	b8	O=[S][N]C1=CC=CC(OC(F)(F)F)=C1	-0.66
a52	C[C]1CCC(CC1)=O	b8	O=[S][N]C1=CC=CC(OC(F)(F)F)=C1	0.13
A6	CC1[C]CCCC1	b8	O=[S][N]C1=CC=CC(OC(F)(F)F)=C1	-1.82
a53	[C]1CCC=CC1	b18	O=S=NC1=C(C#N)C=CC=C1	-2.77
a55	[C]C1CCOCC1	b18	O=S=NC1=C(C#N)C=CC=C1	-0.55
a57	C[C](C)CC=C	b18	O=S=NC1=C(C#N)C=CC=C1	-1.72
a71	CC[C]CC	b18	O=S=NC1=C(C#N)C=CC=C1	-0.87
a53	[C]1CCC=CC1	b19	BrC1=C(C)C=CC=C1N=S=O	-0.55
a54	[C]CC1=CC=C(OC)C=C1	b19	BrC1=C(C)C=CC=C1N=S=O	0.24
a55	[C]C1CCOCC1	b19	BrC1=C(C)C=CC=C1N=S=O	-1.03
a56	C[C]C	b19	BrC1=C(C)C=CC=C1N=S=O	-1.29
a57	C[C](C)CC=C	b19	BrC1=C(C)C=CC=C1N=S=O	0.66
a71	CC[C]CC	b19	BrC1=C(C)C=CC=C1N=S=O	0.71
a54	[C]CC1=CC=C(OC)C=C1	b20	CCC1=C(N=S=O)C=CC=C1	-0.98
a55	[C]C1CCOCC1	b20	CCC1=C(N=S=O)C=CC=C1	-0.08
a57	C[C](C)CC=C	b20	CCC1=C(N=S=O)C=CC=C1	0.50
a71	CC[C]CC	b20	CCC1=C(N=S=O)C=CC=C1	-0.34
a54	[C]CC1=CC=C(OC)C=C1	b21	O=S=NC1=CC(SC)=CC=C1	0.56
a56	C[C]C	b21	O=S=NC1=CC(SC)=CC=C1	0.61
a71	CC[C]CC	b21	O=S=NC1=CC(SC)=CC=C1	0.77
a57	C[C](C)CC=C	b22	O=S=NC1=CC(OC(F)(F)F)=CC=C1	1.08
a59	C[C](C)CCC	b22	O=S=NC1=CC(OC(F)(F)F)=CC=C1	0.45
a70	Br[C@]1(C2)C[C@@H](C[C@@]2C3)C[C@@H]3C1	b22	O=S=NC1=CC(OC(F)(F)F)=CC=C1	0.24
a71	CC[C]CC	b22	O=S=NC1=CC(OC(F)(F)F)=CC=C1	0.24
a57	C[C](C)CC=C	b23	BrC1=CC(N=S=O)=CC=C1C	0.56
a71	CC[C]CC	b23	BrC1=CC(N=S=O)=CC=C1C	-0.08
a54	[C]CC1=CC=C(OC)C=C1	b24	FC1=CC=C(CCN=S=O)C=C1	-0.55
a56	C[C]C	b24	FC1=CC=C(CCN=S=O)C=C1	-0.87
a57	C[C](C)CC=C	b24	FC1=CC=C(CCN=S=O)C=C1	1.35
a58	C[C]1CN(C(OC(C)(C)C)=O)CCC1	b24	FC1=CC=C(CCN=S=O)C=C1	-1.56
a59	C[C](C)CCC	b24	FC1=CC=C(CCN=S=O)C=C1	1.45
a71	CC[C]CC	b24	FC1=CC=C(CCN=S=O)C=C1	0.66
a53	[C]1CCC=CC1	b25	O=S=NCCCCC1=CC=CC=C1	1.14
a54	[C]CC1=CC=C(OC)C=C1	b25	O=S=NCCCCC1=CC=CC=C1	0.66
a59	C[C](C)CCC	b25	O=S=NCCCCC1=CC=CC=C1	0.50
a70	Br[C@]1(C2)C[C@@H](C[C@@]2C3)C[C@@H]3C1	b25	O=S=NCCCCC1=CC=CC=C1	-0.71

$^{\alpha}$ Z-score normalized yields are reported.

Feature generation

Computationally derived descriptors were collected for the alkyl radicals generated from the carboxylic acids and sulfinylamines (Table S4). All descriptors were derived from the geometries optimized at the PW6B95-D3BJ / def2-SVP level of theory in the gas phase. For the alkyl radicals, descriptors were collected for the radical center carbon atom. For sulfinylamines, descriptors were collected for the sulfur, nitrogen, and oxygen atom of the NSO moiety for the *Z* isomers, after computational studies indicated that *Z* isomers were substantially more stable than *E* isomers for all studied sulfinylamines

(e.g., by 5.1 kcal/mol for sulfinylamine **3**), while *Z*-**TSB** was lower in energy than *E*-**TSB** by 1.2 kcal/mol, indicating that the *Z*-sulfinylamine pathway is primarily responsible for the observed reactivity. DBStep[25] was used to collect the sterimol and buried volume parameters. Multiwfn[10] was used to generate the molecular orbital coefficients. All other features were collected from the Gaussian 16 output files. For Fukui indices (derived using Mulliken charges), the nucleophilic (f^-), radical (f^0), and electrophilic (f^+) indices were generated from the corresponding single point calculations of the anionic and cationic alkyl geometries at the PW6B95-D3BJ / def2-SVP level of theory. All parameters were z-score normalized as previously described.[24]

Table S4. Molecular descriptors for the carboxylic acid-derived alkyl radicals and sulfinylamines.

Feature description[a]	Definition
A Bmin	B_{min} Sterimol parameter for the radical carbon atom in the carboxylic acid-derived alkyl radical
A Bmax	B_{max} Sterimol parameter for the radical carbon atom in the carboxylic acid-derived alkyl radical
**A L**	L Sterimol parameter for the radical carbon atom in the carboxylic acid-derived alkyl radical
B(*Z*) Bmin S	B_{min} Sterimol parameter for the sulfur atom in the NSO group of *Z* sulfinylamine
B(*Z*) Bmax S	B_{max} Sterimol parameter for the sulfur atom in the NSO group of *Z* sulfinylamine
B(*Z*) L S	L Sterimol parameter for the sulfur atom in the NSO group atom of *Z* sulfinylamine
B(*Z*) Bmin N	B_{min} Sterimol parameter for the nitrogen atom in the NSO group atom of *Z* sulfinylamine
B(*Z*) Bmax N	B_{max} Sterimol parameter for the nitrogen atom in the NSO group atom of *Z* sulfinylamine
**B(*Z*) L N**	L Sterimol parameter for the nitrogen atom in the NSO group of *Z* sulfinylamine
**B(*Z*) Bmax O**	B_{max} Sterimol parameter for the oxygen atom in the NSO group of *Z* sulfinylamine
B(*Z*) Bmin O	B_{min} Sterimol parameter for the oxygen atom in the NSO group of *Z* sulfinylamine
B(*Z*) L O	L Sterimol parameter for the oxygen atom in the NSO group of *Z* sulfinylamine
A BV_c_rad	Buried Volume for the radical carbon atom in the carboxylic acid-derived alkyl radical
**B(*Z*) BV_S**	Buried Volume for the sulfur atom in the NSO group of *Z* sulfinylamine
**B(*Z*) BV_N**	Buried Volume for the nitrogen atom in the NSO group atom of *Z* sulfinylamine
**B(*Z*) BV_O**	Buried Volume for the oxygen atom in the NSO group of *Z* sulfinylamine

A H1a_ene	HOMO-1 (α space) energy for the alkyl radical
**A H1b_ene**	HOMO-1 (β space) energy for the alkyl radical
**A Ha_ene**	HOMO (α space) energy for the alkyl radical
A Hb_ene	HOMO (β space) energy for the alkyl radical
A Somo_ene	SOMO energy for the alkyl radical
**A Sumo_ene**	SUMO energy for the alkyl radical
**A La_ene**	LUMO (α space) energy for the alkyl radical
**A Lb_ene**	LUMO (β space) energy for the alkyl radical
A L1a_ene	LUMO+1 (α space) energy for the alkyl radical
A L1b_ene	LUMO+1 (β space) energy for the alkyl radical
**B(Z) H1a_ene**	HOMO-1 energy for *Z* sulfinylamine
**B(Z) Ha_ene**	HOMO energy for *Z* sulfinylamine
B(Z) La_ene	LUMO energy for *Z* sulfinylamine
**B(Z) L1a_ene**	LUMO+1 energy for *Z* sulfinylamine
**A α HOMO-1**	HOMO-1 (α space) coefficient for the radical carbon atom in the carboxylic acid-derived alkyl radical
**A α HOMO**	HOMO (α space) coefficient for the radical carbon atom in the carboxylic acid-derived alkyl radical
A α LUMO	LUMO (α space) coefficient for the radical carbon atom in the carboxylic acid-derived alkyl radical
A α LUMO+1	LUMO+1 (α space) coefficient for the radical carbon atom in the carboxylic acid-derived alkyl radical
A β HOMO-1	HOMO-1 (β space) coefficient for the radical carbon atom in the carboxylic acid-derived alkyl radical
A β HOMO	HOMO (β space) coefficient for the radical carbon atom in the carboxylic acid-derived alkyl radical
A β LUMO	LUMO (β space) coefficient for the radical carbon atom in the carboxylic acid-derived alkyl radical
A β LUMO+1	LUMO+1 (β space) coefficient for the radical carbon atom in the carboxylic acid-derived alkyl radical
A Somo	SOMO coefficient for the radical carbon atom in the carboxylic acid-derived alkyl radical
A Sumo	SUMO coefficient for the radical carbon atom in the carboxylic acid-derived alkyl radical
**B(Z) HOMO-1 S**	HOMO-1 coefficient for the sulfur atom in the NSO group of *Z* Sulfinylamine
**B(Z) HOMO S**	HOMO coefficient for the sulfur atom in the NSO group of *Z* sulfinylamine

**B(*Z*) LUMO S**	LUMO coefficient for the sulfur atom in the NSO group of *Z* sulfinylamine
**B(*Z*) LUMO+1 S**	LUMO+1 coefficient for the sulfur atom in the NSO group of *Z* sulfinylamine
**B(*Z*) HOMO-1 N**	HOMO-1 coefficient for the nitrogen atom in the NSO group atom of *Z* sulfinylamine
**B(*Z*) HOMO N**	HOMO coefficient for the nitrogen atom in the NSO group atom of *Z* sulfinylamine
B(*Z*) LUMO N	LUMO coefficient for the nitrogen atom in the NSO group atom of *Z* sulfinylamine
B(*Z*) LUMO+1 N	LUMO+1 coefficient for the nitrogen atom in the NSO group atom of *Z* sulfinylamine
**B(*Z*) HOMO-1 O**	HOMO-1 coefficient for the oxygen atom in the NSO group of *Z* sulfinylamine
**B(*Z*) HOMO O**	HOMO coefficient for the oxygen atom in the NSO group of *Z* sulfinylamine
B(*Z*) LUMO O	LUMO coefficient for the oxygen atom in the NSO group of *Z* sulfinylamine
B(*Z*) LUMO+1 O	LUMO+1 coefficient for the oxygen atom in the NSO group of *Z* sulfinylamine
**A Fukui electrophilic**	Fukui index for the radical carbon atom in the carboxylic acid-derived alkyl radical
**A Fukui nucleophilic**	Fukui index for the radical carbon atom in the carboxylic acid-derived alkyl radical
**A Fukui ave**	Fukui index for the radical carbon atom in the carboxylic acid-derived alkyl radical
**B(*Z*) Fukui electrophilic S**	Fukui index for the sulfur atom in the NSO group of *Z* sulfinylamine
**B(*Z*) Fukui nucleophilic S**	Fukui index for the sulfur atom in the NSO group of *Z* sulfinylamine
**B(*Z*) Fukui ave S**	Fukui index for the sulfur atom in the NSO group of *Z* sulfinylamine
**B(*Z*) Fukui electrophilic N**	Fukui index for the nitrogen atom in the NSO group atom of *Z* sulfinylamine
B(*Z*) Fukui nucleophilic N	Fukui index for the nitrogen atom in the NSO group atom of *Z* sulfinylamine
B(*Z*) Fukui ave N	Fukui index for the nitrogen atom in the NSO group atom of *Z* sulfinylamine
**B(*Z*) Fukui electrophilic O**	Fukui index for the oxygen atom in the NSO group of *Z* sulfinylamine
**B(*Z*) Fukui nucelophilc O**	Fukui index for the oxygen atom in the NSO group of *Z* sulfinylamine
**B(*Z*) Fukui ave O**	Fukui index for the oxygen atom in the NSO group of *Z* sulfinylamine
**A SD**	Spin Density for the radical carbon atom in the carboxylic acid-derived alkyl radical

[a] Features in bold were selected for model development.

Predictive model development

A set of ML algorithms available in the scikit-learn[26] and XGBoost[27] python packages were evaluated (Table S5) to develop a predictive model. The predictive performance of the various regression models in leave-one-out cross validation (LOOCV) is reported in Table S5 (see Table S6 for model details). The Support Vector Regression (SVR)[28] was identified as the best-performing model, with LOOCV $R^2 = 0.81$. Feature selection was then performed using forward feature selection.[29] In each round of feature selection, the best-performing feature was selected, and the best-performing feature combination was then iteratively selected to provide the model with LOOCV $R^2 = 0.85$, Pearson R = 0.98, $R^2 = 0.97$, RMSE = 0.18, MAE = 0.10. The relative importance of the features in the developed model was evaluated by means of feature importance analysis (Figure S3). To ensure that the ML algorithm was learning from the meaningful chemical features and not based on unrelated patterns within the dataset, the yields were randomly shuffled, and the predictive performance of a model trained on the Y-randomized dataset was tested. The straw model showed low predictive performance (LOOCV $R^2 = –0.44$, MAE = 0.99), pointing to the importance of the chemical features for the development of the predictive model. Additionally, the computationally produced features were replaced by random numbers for all alkyl radical and sulfinylamine fragments, and another straw model was generated. The resulting straw model also showed low predictive performance (LOOCV $R^2 = 0.29$, MAE = 0.69), supporting the conclusion.

Table S5. Predictive performance of evaluated machine learning algorithms based on LOOCV.

Method	R^2	MAE
XGBoost	0.50	0.55
Random Forest	0.65	0.48
Decision tree	0.32	0.68
Bagging	0.53	0.52
Extra Trees	0.72	0.43
Gradient Boosting	0.65	0.46
KNNR	0.59	0.50
Kernel ridge	0.73	0.42
LSVR	0.70	0.41
Ridge	0.73	0.43
SVR	0.81	0.37

Table S6. Hyperparameters

Models	Hyperparameters
Support Vector Regression (SVR)[28]	(kernel='rbf', degree=3, gamma='auto', coef0=0.0, tol=0.05, C=4.25, epsilon=0.05, shrinking=True, cache_size=200, verbose=False, max_iter=-1)
Bagging (BG)[30]	(base_estimator=None, n_estimators=10, max_samples=1.0, max_features=1.0, bootstrap=True, bootstrap_features=False, oob_score=False, warm_start=False, n_jobs=60, random_state=None, verbose=0)
Decision Tree (DT)[31]	(criterion='friedman_mse', splitter='best', max_depth=None, min_samples_split=2, min_samples_leaf=1, min_weight_fraction_leaf=0.0, max_features=None, random_state=None, max_leaf_nodes=None, min_impurity_decrease=0.0, ccp_alpha=0.0)
Extra Trees (ET)[32]	(n_estimators=100, criterion='friedman_mse', max_depth=None, min_samples_split=2, min_samples_leaf=1, min_weight_fraction_leaf=0.0, max_features='sqrt', max_leaf_nodes=None, min_impurity_decrease=0.0, bootstrap=False, oob_score=False, n_jobs=60, random_state=None, verbose=0, warm_start=False, ccp_alpha=0.0, max_samples=None)
Gradient Boosting (GB)[33]	(loss='squared_error', learning_rate=0.1, n_estimators=100, subsample=1.0, criterion='friedman_mse', min_samples_split=2,min_samples_leaf=1, min_weight_fraction_leaf=0.0, max_depth=3, min_impurity_decrease=0.0, init=None, random_state=None, max_features=None, alpha=0.9, verbose=0, max_leaf_nodes=None, warm_start=False, validation_fraction=0.1, n_iter_no_change=None, tol=0.0001, ccp_alpha=0.0)
Kernel Ridge[34]	(alpha=1, kernel='linear', gamma=None, degree=3, coef0=1, kernel_params=None)
K-Nearest Neighbor[35]	(n_neighbors=5, weights='uniform', algorithm='auto', leaf_size=30, p=2, metric='minkowski', metric_params=None, n_jobs=None)
Linear SVR[25]	(epsilon=0.0, tol=0.0001, C=1.0, loss='epsilon_insensitive',fit_intercept=True, intercept_scaling=1.0, dual=True, verbose=0, random_state=None, max_iter=1000)
Random Forest (RF)[36]	(n_estimators=100, criterion='friedman_mse', max_depth=None, min_samples_split=2, min_samples_leaf=1, min_weight_fraction_leaf=0.0, max_features='sqrt', max_leaf_nodes=None, min_impurity_decrease=0.0, bootstrap=True, oob_score=False, n_jobs=60, random_state=None, verbose=0, warm_start=False, ccp_alpha=0.0, max_samples=None)
Ridge[37	(alpha=1.0, fit_intercept=True, copy_X=True, max_iter=None, tol=0.001)
XGBoost (XGB)[38]	(base_score=0.5, colsample_bynode=1, colsample_bytree=1, gamma=0, gpu_id=-1, importance_type='gain', interaction_constraints='', learning_rate=0.3, max_delta_step=0, max_depth=10, min_child_weight=1, missing=np.nan, monotone_constraints='()', n_estimators=60, num_parallel_tree=1, random_state=0, reg_alpha=0, reg_lambda=1, scale_pos_weight=1, subsample=1, tree_method='exact', validate_parameters=1, verbosity=None)

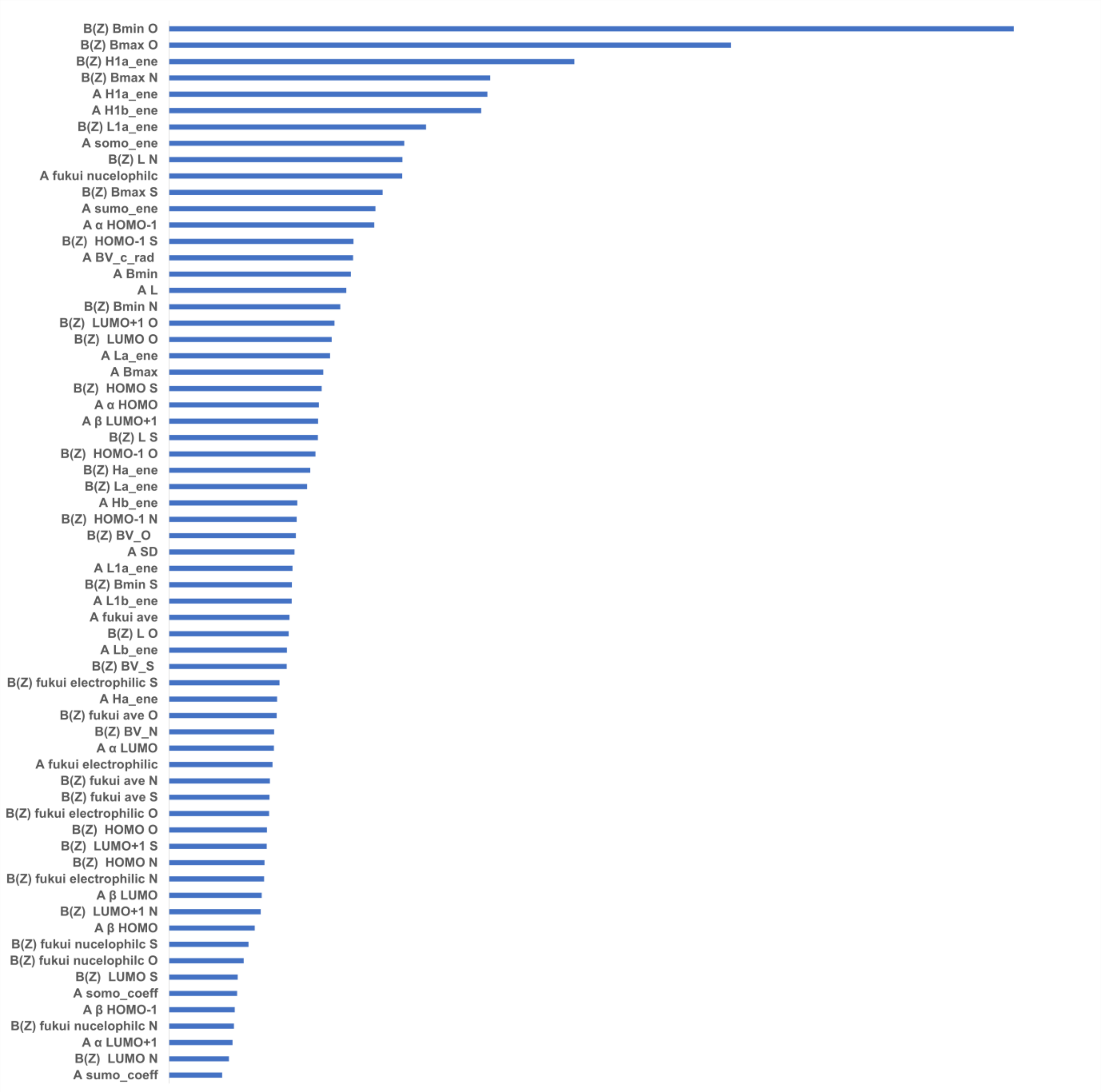

Figure S3. Feature importance analysis for the developed predictive model.

Table S7. Computationally-derived steric and MO energy descriptors for the alkyl radical fragments.[a]

	Steric				MO Energies									
Alkyl radical	Bmin	Bmax	L	BV_c_rad	h1a	h1b	A ha	A hb	somo	sumo	la	lb	l1a	l1b
a1	0.31435	-0.80381	-0.73281	0.12807	-0.48377	-0.48109	-0.87054	-0.89978	0.98289	1.1716	0.79265	0.79091	0.91674	0.93286
a2	0.79446	-0.0942	-0.66619	0.35386	-0.54922	-0.58637	-1.0374	-1.10074	0.56536	0.67135	0.90592	0.96438	0.63325	0.62113
a3	-1.40837	-0.82946	-1.06596	-0.33909	-0.82998	-0.77036	-1.32654	-1.31428	0.36466	0.6211	1.18839	1.17022	1.21637	1.35023
a4	1.3028	0.07679	-0.74947	1.3868	0.27346	0.27787	-0.41369	-0.42064	1.20112	0.89035	0.48208	0.51154	1.05803	1.02214
a5	-1.09771	0.82915	0.39987	-1.10731	0.64054	0.60406	0.81117	0.78541	-1.6872	-0.80241	-1.53637	-1.47531	-2.18582	-2.2517
a6	-0.70234	-0.27374	0.2333	-1.07227	0.88209	0.81753	0.69917	0.72954	-1.42405	-0.63591	-1.2556	-1.2919	-1.76774	-1.81795
a7	0.34259	0.30763	-0.74947	0.34348	-0.47903	-0.48563	-0.81111	-0.81905	0.97587	1.04785	0.85085	0.85962	0.78885	0.78024
a8	-0.05278	-0.76106	-0.88273	1.03903	-0.70635	-0.67901	0.21603	0.33025	0.21098	-0.11241	0.76625	0.75627	0.81656	0.85222
a9	0.76621	0.25633	-0.56624	1.39459	-0.4512	-0.43963	0.8846	0.85796	-0.13567	-0.68616	-1.0654	-0.96994	-0.33719	-0.31866
a10	1.18983	1.62426	0.59976	1.56329	1.85843	1.81652	1.72946	1.71123	0.84254	0.29109	-0.72501	-0.77177	-1.11976	-1.19018
a11	-0.25047	2.18852	1.03284	-1.12029	0.82992	0.97139	0.2826	0.42415	0.85447	-2.52592	-3.0106	-2.90199	-2.37522	-2.44849
a12	-0.30696	0.84625	0.74967	-1.09434	1.60834	1.59981	1.57946	1.62201	-0.78477	-0.03141	-0.78293	-0.81577	-1.38864	-1.43146
a13	0.22963	-0.79526	-0.73281	1.3894	-0.32379	-0.31913	-0.8714	-0.88984	1.85935	1.4686	0.76	0.75116	0.89147	0.89146
a14	0.25787	1.02579	-0.41633	0.95857	0.6124	0.54673	0.17631	0.1097	1.16533	0.89185	1.01976	0.9959	0.74957	0.72587
a15	-1.46485	-0.42763	0.8163	-1.23578	1.27667	1.22179	0.68546	0.68362	-0.95178	-0.10491	-0.91919	-0.96483	-1.75221	-1.78551
a16	1.75466	-0.44473	0.16667	1.34528	-0.09772	-0.07942	-0.17711	-0.22231	0.06222	0.21159	-0.36164	-0.40665	-0.21752	-0.20065
a17	0.28611	-0.80381	-0.51627	0.11379	-1.51575	-1.49401	-1.19997	-1.25987	-0.61846	-0.38616	0.76057	0.76706	0.93349	0.92266
a18	0.20139	-0.10275	-0.04987	0.36554	-0.69086	-0.8047	-0.7634	-0.60288	-0.71109	-0.84516	-0.76078	-0.74309	0.43837	0.40488
a19	1.10511	1.03434	-0.48296	1.3907	0.18525	0.17292	-0.37969	-0.39577	1.21305	0.8161	0.47072	0.45731	0.70725	0.66439
a20	-0.67409	-0.0087	0.88293	-1.23578	0.87861	0.81267	0.30003	0.2504	-0.98336	-0.68391	-2.48001	-2.24671	-2.18308	-2.27425
a21	-0.75882	-0.75251	-1.2825	0.834	-0.86918	-0.84778	0.21346	0.33084	0.23274	-0.02316	0.94822	0.93116	0.84244	0.89486
a22	0.9639	-0.8551	-1.0493	0.94949	-0.59221	-0.56985	-1.18311	-1.10864	1.50006	1.1431	1.08534	1.06858	0.98404	0.96468
a23	-0.53289	-1.23128	1.66581	-1.23838	-0.73101	-0.83871	-1.14111	-1.15457	-0.81495	0.21084	0.92494	0.88602	0.43289	0.40982
a24	-1.52133	1.22243	2.16553	-1.22799	-0.43982	-0.53843	-0.81369	-0.89423	-0.81354	0.19659	0.86164	0.82242	0.30896	0.26246
a25	-0.53289	-1.18854	0.38321	-1.23448	0.17165	0.11461	-0.46283	-0.54935	-1.42264	-0.44466	0.4324	0.42523	0.42071	0.41198
a26	-0.39168	-0.42763	0.05007	-1.22799	1.25675	1.22989	0.60717	0.54847	-1.95455	-0.96591	0.18513	0.14046	-0.08172	-0.1265
a27	-1.2954	-1.07739	-0.04987	-1.23448	0.06574	0.0495	-0.49283	-0.57889	-1.59667	-0.59391	0.41082	0.38463	0.47765	0.52444
a28	-0.53289	-1.19708	5.04721	-1.23838	-0.67505	-0.78202	-0.65969	-0.72954	-0.81003	0.21534	0.89599	0.85082	0.2374	0.19016
a29	0.51204	0.99159	2.06558	-1.23708	1.39429	1.34002	0.95089	0.89949	-0.89494	0.12384	-0.52232	-0.57075	-0.69559	-0.76415
a30	3.64683	2.14578	0.43318	-0.97365	0.69239	0.62868	0.36517	0.49465	-2.09841	-1.45716	-0.48683	-0.46911	-0.41454	-0.47282
a31	0.76621	3.04347	5.51361	-1.23578	-0.32537	-0.38068	0.86546	0.8094	-0.82407	0.20109	-0.05561	-0.10485	0.13265	0.07864
a32	-1.06947	-1.05174	0.01676	-1.23059	0.60607	0.59175	-0.01626	-0.09068	-1.63527	-0.68991	-0.48513	-0.50829	0.23222	0.25473
a33	-1.43661	2.83829	3.96449	-1.23448	-0.36773	-0.46587	0.84489	0.7939	-0.84512	0.17409	-0.06867	-0.1162	0.13874	0.0879
a34	-0.44816	0.55557	-0.4663	0.24745	-0.93811	-0.94625	-1.34169	-1.36693	0.58079	0.88135	1.01976	0.98823	0.5078	0.48335
a35	-0.64585	3.77018	1.66581	0.40966	-0.60138	-0.64856	-0.90654	-0.9384	0.8285	0.97735	0.78499	0.76138	0.28673	0.23743
a36	-0.56113	-1.16289	0.4665	-1.23838	0.6946	0.64099	0.01089	-0.06406	-1.45422	-0.51741	-0.44879	-0.45548	0.17132	0.15556
a37	1.72642	2.05173	1.29935	-0.98922	0.86597	0.81364	0.44831	0.45984	-1.45633	-0.60066	-1.50145	-1.54771	-2.21414	-2.30514
a38	-1.43661	0.58121	0.38321	-1.23189	1.246	1.19296	0.69489	0.66694	-0.61004	0.3916	-0.98278	-1.0148	-1.77231	-1.84513
a39	-0.53289	-0.64137	0.43318	-1.23059	1.41263	1.35784	1.20289	1.15896	-1.50615	-0.47016	-1.04354	-1.09032	-0.14018	-0.19323

a40	-0.30696	0.58121	0.13336	-0.94121	-0.35856	-0.35606	0.7166	0.67835	-0.9188	-0.75816	-1.13069	-1.17578	-0.15236	-0.19941
a41	-1.06947	1.13693	1.44927	-1.05541	2.3213	2.32768	1.4826	1.75189	-1.26054	-1.19316	-1.41657	-1.46452	-1.24095	-1.31437
a42	0.70973	1.77815	0.91624	-0.99312	1.55111	1.54507	1.15231	1.11567	-1.3651	-0.48291	-1.88044	-1.92759	-2.04149	-2.12658
a43	-1.1542	0.8035	-0.24976	-0.88152	1.02342	0.96848	0.7306	0.81232	-0.24163	-3.66892	-1.53268	-1.47645	-2.32102	-2.15994
a44	-0.53289	-0.35923	-0.09984	0.08914	1.63932	1.70574	2.21603	0.86878	-0.27111	-0.29766	0.10394	-2.39662	-0.69833	-0.7555
a45	-0.67409	0.08534	-0.1165	-0.09902	1.44077	1.39056	0.8306	0.86995	0.24116	0.41485	-0.9561	-0.89726	-1.56585	-1.62857
a46	2.20652	-0.14549	0.61641	1.34009	0.91845	0.85575	0.90546	0.9071	0.83973	0.38785	0.61607	0.58791	0.1293	0.08234
a47	0.08842	0.08534	-0.03322	0.09433	-0.06516	0.3812	-0.03511	-0.04446	2.87264	1.32685	0.74978	0.73839	0.12047	0.26153
a48	2.23476	-0.16259	0.59976	0.09303	0.88304	0.8198	0.82231	0.87405	0.01169	0.01209	0.63594	0.61517	0.12352	0.08018
a49	-0.19399	0.15374	-0.88273	0.08005	-0.28268	-0.32464	0.30231	0.35015	0.12888	0.15234	1.07767	1.1211	1.10523	1.10185
a50	0.20139	0.15374	-0.88273	1.3323	-0.26213	-0.30941	0.39517	0.38964	0.97096	0.54835	0.97746	0.97318	0.90669	0.89269
a51	-0.67409	-0.80381	0.36656	-1.1138	0.96145	0.90369	0.29631	0.60376	0.02923	-3.56167	-1.58832	-1.5267	-2.28813	-2.12565
a52	0.39908	-0.79526	-0.53293	1.35696	-1.30075	-1.25722	0.78803	0.74124	0.20326	-0.23316	-1.1375	-1.16556	0.20604	0.20623
a53	-0.39168	-0.80381	-0.71616	0.08005	-0.56186	-0.58605	0.7846	0.77283	0.44957	0.7786	-0.04709	-0.06226	0.23984	0.24763
a54	-0.44816	-0.42763	0.74967	-1.09434	1.26561	1.21628	1.43317	1.51056	-0.78618	-0.07941	-0.90727	-0.9353	-1.3591	-1.40242
a55	-0.78706	0.47007	-0.4663	-1.01907	-0.25897	-0.31297	0.41374	0.3873	-1.18546	-0.27591	1.00954	0.9817	0.74531	0.74842
a56	-1.18244	-0.78671	-1.31581	-0.15353	-1.96187	-1.96014	-2.15426	-2.10115	0.57448	1.0996	1.22303	1.20685	1.34913	1.42252
a57	-0.13751	-0.80381	-0.53293	1.28299	-1.33269	-1.34436	0.28831	0.258	1.11832	0.87385	-0.27079	-0.23744	0.25689	0.22971
a58	1.38752	0.0084	0.56644	1.31933	0.94785	0.88815	0.98374	0.99339	0.61448	0.3646	0.64986	0.6339	0.14118	0.10242
a59	0.87918	-0.77816	-0.4663	1.30246	-1.05446	-0.97702	-1.42883	-1.48716	1.559	1.41385	0.7566	0.72561	0.91187	0.88126
a60	0.17315	0.50427	-0.38301	1.40238	0.82739	0.80943	0.14089	0.07957	0.16748	-0.60591	-0.62338	-0.44895	-0.10212	-0.13175
a61	-0.70234	0.17084	-0.86607	0.24226	-1.01652	-0.98739	-1.50254	-1.50354	0.63974	0.8941	0.98768	1.03366	1.05712	1.04006

[a] See Table S4 for the definition of the descriptors.

Table S8. Computationally-derived descriptors for the alkyl radical fragments based on MO coefficients, charges, and spin density.[a]

	MO Coefficients										Charges and spin density			
Alkyl radical	α homo-1	α homo	α lumo	α lumo+1	β homo-1	β homo	β lumo	A β lumo+1	somo	sumo	fukui electrophilic	A fukui nucelophilc	fukui ave	SD
a1	0.4261	0.37812	0.70402	0.23696	0.69951	0.69229	0.04498	0.178283	0.15716	0.13405	0.60804389	0.36620348	0.49575	0.23678
a2	-0.29636	-0.56014	0.68861	0.5297	0.05302	-0.355	0.25527	0.26685	0.19885	0.03966	0.521405163	-0.018606	0.23862	0.28055
a3	2.21112	2.65007	0.68861	0.7192	1.10931	2.83538	-0.0418	1.348402	0.35384	0.2491	1.231015079	0.81620059	1.04561	0.26649
a4	-0.00715	0.2502	-0.17659	-0.67633	0.20394	0.60214	-0.0169	-0.673763	-0.55734	-0.17948	-1.01666117	-1.1276057	-1.11781	-0.86178
a5	-0.5867	-0.44142	-0.22753	-0.78975	-0.62588	-0.62226	0.32403	-0.503559	1.10944	0.44513	0.394370249	0.67505326	0.56684	0.93724
a6	-0.88327	0.26294	-0.79183	-0.54735	-0.94106	-0.42974	-0.39518	-0.565685	0.66604	0.81477	0.190945335	0.93271314	0.61413	0.77774
a7	0.40772	0.54178	0.52592	-0.15307	0.60226	0.96257	0.03419	-0.131319	0.08332	0.05288	0.656540446	0.33753293	0.50284	0.21717
a8	1.16793	0.29744	0.69256	1.161	1.31343	-0.28557	0.01629	0.789014	-0.64508	-0.20073	-1.06933117	-0.7275532	-0.91868	-0.57131
a9	0.40447	-0.822	-0.56319	-0.34003	0.64554	-0.66424	0.01724	0.11359	-0.5259	-0.47402	-1.00125286	-1.3165823	-1.2164	-0.90236
a10	-0.90588	-0.71619	-0.91899	-0.95125	-0.95425	-0.71277	-0.49902	-0.907376	-0.50128	-0.59769	-2.05798118	-1.9064665	-2.05187	-0.45941
a11	-0.75887	-0.8657	-0.86607	-0.9521	-0.94417	1.15633	-0.4752	-0.930481	-2.49041	-1.68331	-1.18877639	-0.8628344	-1.05158	-2.49083
a12	-0.52479	1.0715	-0.77841	-0.50351	-0.87607	-0.54486	-0.38427	-0.519476	-0.21416	0.83397	-0.49890672	0.99499019	0.31953	0.79993
a13	0.67969	0.32362	0.28385	-0.28544	0.71534	0.89916	-0.08104	-0.259421	-0.42992	-0.51397	-1.13681077	-1.4614653	-1.36238	-0.43656
a14	-0.63109	-0.74079	0.03941	0.04407	-0.6293	-0.58206	-0.23941	-0.047115	-0.286	-0.38638	-0.58239335	-0.8837935	-0.77368	-0.39466
a15	-0.91366	-0.29209	-0.94544	-0.89298	-0.95813	-0.64617	-0.50697	-0.285606	0.89946	0.7997	0.436228024	0.64455236	0.56974	0.96059
a16	0.51826	-0.65798	-0.85501	-0.90684	0.86626	-0.62155	-0.47994	0.235274	-0.18331	0.0284	-0.851713	-0.9480959	-0.93838	-0.68246
a17	1.16256	-0.02953	1.63359	0.0698	1.51337	0.26721	0.26689	-0.027348	0.36161	0.20808	0.824006766	0.33127672	0.57932	0.22659
a18	-0.90701	0.41793	-0.52568	-0.51681	-0.9392	0.00844	-0.37835	-0.555673	-0.44688	0.68356	-1.02956893	0.62701396	-0.14025	-2.54011
a19	-0.19968	0.0285	-0.41511	-0.81152	0.01657	0.36817	-0.24783	-0.80777	-0.6253	-0.16617	-1.04113837	-1.0848324	-1.10552	-0.86776
a20	-0.8998	-0.69832	-0.91978	-0.97133	-0.9558	-0.62014	6.89151	-0.945114	1.18658	-5.04797	1.106040476	-0.0016077	0.52741	0.90371
a21	1.06191	0.33831	1.12852	1.54792	1.20191	-0.27777	0.11029	1.216192	-0.60339	-0.15334	-1.17856289	-0.8303381	-1.02848	-0.54559
a22	1.59623	1.88237	0.75851	0.04661	1.81366	2.36798	-0.0105	-0.060208	-0.36326	-0.44102	-0.85030424	-1.1944136	-1.0758	-0.44568
a23	-0.90758	-0.18735	-0.47947	-0.03513	-0.94448	-0.09747	-0.39933	-0.169826	1.17799	1.0207	1.48885334	1.43083956	1.51336	0.99053
a24	-0.90532	-0.78449	-0.26781	-0.68	-0.91097	-0.6607	-0.3314	-0.705853	1.16786	1.01365	1.659101961	1.45936009	1.61067	0.98469
a25	-0.76029	-0.88091	-0.45775	1.13215	-0.85079	-0.72481	-0.25387	0.606231	1.22003	0.96537	1.589368351	1.19014797	1.42643	0.98896
a26	-0.53355	-0.83456	-0.66902	-0.09339	-0.7706	-0.70179	-0.43145	-0.198835	1.11427	1.06525	1.059287261	1.56435041	1.38303	0.89543
a27	-0.59024	-0.87578	-0.04628	1.67718	-0.80658	-0.71932	-0.24036	0.960758	1.16927	1.02696	1.59359463	1.45703464	1.57808	0.99178
a28	-0.91281	-0.72911	-0.76103	-0.71055	-0.95549	-0.59534	-0.47022	-0.761304	1.17693	1.02099	0.994413872	1.25263507	1.17728	0.99066
a29	-0.89867	-0.84712	-0.94623	-0.97076	-0.95487	-0.72269	-0.50436	-0.942033	1.16821	1.01443	0.124504702	0.82637255	0.52277	0.99092
a30	-0.83365	1.4749	0.37231	-0.86328	-0.86242	-0.34437	-0.04417	-0.82574	0.0083	0.93463	0.263989531	1.58092862	1.01244	0.76727
a31	-0.75209	-0.87897	-0.94781	-0.86442	-0.68172	-0.72658	-0.50756	-0.882731	1.17775	1.02119	0.171310746	0.97586149	0.62894	0.99077
a32	-0.59872	-0.87879	-0.52331	1.25688	-0.87282	-0.72269	-0.36519	0.902483	1.09378	0.98535	1.336038122	1.30640548	1.3706	0.97727
a33	-0.90814	-0.82164	-0.94229	-0.79512	-0.94944	-0.71773	-0.50009	-0.838832	1.16044	1.01658	0.346296321	1.04994583	0.75406	0.99059
a34	0.41451	0.28558	-0.0139	-0.38585	0.89774	0.76668	-0.23586	-0.370066	0.29095	0.24235	0.623434591	0.29255422	0.46181	0.32775
a35	-0.69046	0.07468	-0.54305	-0.92353	-0.67024	0.42697	-0.35701	-0.884528	0.17754	0.05199	0.524152245	0.28694313	0.41124	0.4104
a36	-0.8051	-0.88233	-0.79775	0.66546	-0.90632	-0.72641	-0.28647	0.341555	1.19966	0.89771	1.339894602	0.91711012	1.15419	0.9816
a37	-0.81612	-0.14772	-0.9411	-0.9668	-0.9209	-0.5964	-0.47852	-0.94229	0.81479	0.90741	0.31375397	0.58787139	0.47946	0.96607
a38	-0.88567	-0.56439	-0.9024	-0.94955	-0.95239	-0.6738	-0.39542	-0.760277	1.06575	0.95343	0.577033566	0.83430909	0.74338	0.91743
a39	-0.91055	-0.86941	-0.9028	-0.8466	-0.95456	-0.71365	-0.49428	-0.837292	1.27891	1.01316	0.783346437	1.48022913	1.20406	1.01712

Go to table of contents

a40	-0.60621	-0.70982	-0.91899	-0.37906	-0.62402	-0.57674	-0.48907	-0.489953	0.39847	0.58007	1.000048912	0.87247648	0.96683	0.61514
a41	5.60702	1.52834	-0.93676	-0.9487	-0.95719	-0.71436	-0.49891	-0.906349	-5.8685	0.87637	-1.29327115	0.55199944	-0.30827	0.79319
a42	-0.55137	-0.67497	-0.94307	-0.95153	-0.87189	-0.70639	-0.4797	-0.927913	0.84812	0.83602	-0.3998533	0.0672256	-0.1533	0.81488
a43	-0.69498	-0.38834	-0.60149	-0.8135	-0.70592	-0.533	-0.40146	-0.776963	-2.44825	-1.79542	-1.05099969	-0.715956	-0.90342	-2.67388
a44	-0.89047	0.17482	-0.64059	-0.05945	-0.9316	-0.47827	-0.4247	0.181877	-0.1806	0.13434	-0.09721648	0.21413404	0.07362	0.18671
a45	-0.8926	-0.10154	-0.42656	-0.87743	-0.93253	-0.49474	0.38152	-0.861424	0.08085	-0.19701	0.485252865	0.32715092	0.4152	0.34317
a46	-0.88284	-0.36145	-0.21963	-0.81577	-0.91795	-0.52219	-0.26798	-0.772856	-0.48656	-0.43015	-1.383467	-1.3593405	-1.42294	-0.4474
a47	-0.6513	-0.68222	0.38139	-0.04163	0.78017	-0.49545	-0.19472	-0.486359	-1.16115	-0.91426	-0.17402911	0.44739919	0.16771	-1.79446
a48	-0.88864	0.18172	-0.01627	-0.68085	-0.92617	-0.44409	-0.21914	-0.67633	-0.15752	0.15461	-0.1423144	0.19820096	0.04314	0.19331
a49	-0.42443	0.13732	2.35308	0.00023	-0.35476	-0.23969	0.57582	-0.08511	0.00995	0.12719	0.359204083	0.26190329	0.31841	0.21475
a50	-0.46161	-0.30837	0.59858	-0.2416	-0.40299	-0.36279	0.01214	-0.283039	-0.42132	-0.44308	-1.17814026	-1.3610059	-1.32579	-0.42678
a51	-0.91168	1.24136	-0.89055	-0.78296	-0.9558	0.29608	-0.49013	-0.747441	-2.68626	-1.82637	-1.2271651	-0.7701615	-1.01796	-2.69039
a52	1.42915	-0.79723	-0.59715	0.08281	1.70291	-0.6375	-0.33105	0.132844	-0.30202	-0.49547	-1.02678663	-1.3222984	-1.2318	-0.43496
a53	0.33931	-0.60986	-0.6785	0.76446	0.5565	-0.57692	-0.40715	0.668613	0.28541	0.24058	0.653828584	0.48141078	0.58221	0.31441
a54	-0.84213	1.48959	-0.78038	-0.49078	-0.93579	-0.47721	-0.34859	-0.528204	-0.24019	0.80028	-0.47224595	0.93641886	0.29943	0.78998
a55	-0.56253	-0.4899	0.42957	1.10443	-0.55469	-0.43328	-0.13094	0.502517	1.02253	0.96762	1.507237655	1.53533479	1.58073	0.97076
a56	1.52075	3.19271	1.53368	3.39232	1.98427	3.54013	0.19031	2.758551	0.48821	0.38746	0.488087994	0.00175293	0.23412	0.23572
a57	0.7433	-0.33403	0.06508	0.08904	1.22285	-0.14476	-0.04974	0.009876	-0.21699	-0.44044	-1.08880728	-1.3586354	-1.2818	-0.38467
a58	-0.87196	-0.25352	-0.22714	-0.76853	-0.92074	-0.11908	-0.2534	-0.639106	-0.25326	-0.36455	-1.05128144	-1.3550947	-1.26189	-0.43937
a59	1.77929	-0.14347	0.11207	-0.55159	2.064	0.22311	-0.18547	-0.561834	-0.25574	-0.28866	-1.16803241	-1.5046737	-1.40151	-0.38228
a60	-0.71887	-0.86269	0.15156	-0.58554	-0.76424	-0.70232	0.53682	-0.55824	-0.67547	-0.65899	-1.06094905	-1.2700133	-1.2188	-0.98282
a61	0.89865	1.43032	0.76878	0.01493	1.31405	1.81874	0.23666	-0.238883	0.33806	0.09948	0.725464019	0.12500179	0.41661	0.41874

[a] See Table S4 for the definition of the descriptors.

Table S9. Computationally derived steric and MO energy descriptors for the sulfinylamine fragments.[a]

	Sterimol Parameters									Buried Volume			MO Energies			
**Sulfinylamine**	**Bmin S**	**Bmax S**	**L S**	**Bmin N**	**Bmax N**	**L N**	**Bmin O**	**B (Z) Bmax O**	**L O**	**BV_S**	**B (Z) BV_N**	**BV_O**	**h1a**	**ha**	**la**	**l1a**
b1	-0.67309	0.75368	0.334997	-0.17542	0.09619	0.636353	-1.31233	1.18774361	-0.02116	-0.1306	0.116336	0.474738	0.966901	0.779285	0.132279	-1.15761
b2	4.136598	-1.03103	-0.325	3.566033	-0.63396	-0.51582	0.076029	-0.8360958	-0.37663	-0.17317	0.521788	-1.21827	-1.12692	-1.65483	2.274673	2.72881
b3	0.964252	3.903168	1.98498	1.608963	3.736181	2.416977	1.686529	4.96557715	1.723873	-0.00291	0.681836	1.672409	0.412187	0.846445	-1.51186	-0.7194
b4	-0.57075	0.036296	-0.385	-0.46323	0.149877	-0.51582	0.520305	-0.1614826	-0.50589	-0.10932	0.100331	0.452559	0.458471	0.237604	-0.52538	-0.33075
b5	-0.46842	-0.39238	-0.445	0.285063	0.235776	-0.2365	0.686908	0.48421849	-0.40894	-0.27958	0.020308	-1.15912	0.814234	0.314807	0.383913	-0.32758
b6	-0.77542	-0.03369	0.274997	1.148476	-0.26888	0.042811	-0.03504	-0.6529865	0.657465	0.869636	1.140637	-0.47896	1.029074	0.190528	1.443581	0.466359
b7	-0.57075	-0.54111	0.784992	-0.46323	-0.53732	0.845838	-0.09057	0.07945061	0.722095	-0.15189	0.094996	0.437773	-1.60012	-0.67817	-2.04019	-0.79831
b8	-0.05909	1.331086	0.394996	1.781646	0.600849	0.880752	-1.31233	0.86007438	-0.05347	-0.10932	0.089661	0.496917	-0.00022	-0.17101	-0.73089	-0.18323
b9	1.066586	-0.33114	1.774982	0.285063	-0.56953	1.998007	-0.09057	-0.084384	1.756188	-0.15189	-0.070386	-1.19609	0.080602	0.616718	1.349343	-0.04086
b10	1.066586	-0.44488	-0.055	-0.80859	1.019609	-0.55073	-1.20126	-0.2193066	-0.21505	1.997567	0.212364	1.066181	-0.6254	0.164166	-0.55645	-0.4989
b11	-0.57075	-0.69858	-0.415	-0.46323	-0.56953	-0.58564	-0.03504	-0.2964053	-0.50589	-0.17317	0.070989	0.43038	0.373502	-0.36433	-0.75696	-0.60161
b12	0.554917	-0.84731	0.394996	-0.17542	-0.48363	0.426868	0.464771	-0.6529865	0.26968	0.124777	-0.10773	-1.16651	-0.175	-0.72336	-1.13591	-1.65015
b13	-0.62192	-1.29349	-2.93497	-1.72957	-2.11572	-2.08695	-1.31233	-1.6070822	-2.8326	-2.59928	-3.999542	-2.97781	-3.03492	-3.36272	0.915251	3.05162
b14	-0.57075	-0.60235	-0.385	-0.46323	-0.52658	-0.51582	0.187098	-0.4216906	-0.40894	-0.15189	0.097664	0.363843	0.076457	0.430299	0.315741	0.136806
b15	-0.77542	1.322337	0.094999	-0.40567	1.706805	-0.27142	0.742443	0.64805311	0.398941	-0.15189	0.102999	0.363843	0.997987	0.254551	-0.25169	-0.03888
b16	-0.57075	-0.73358	0.544994	-0.52079	-0.45142	0.461782	-0.20164	-0.4795146	0.592834	-0.1306	0.094996	0.422987	-1.08064	-0.64176	-1.21812	-0.51754
b17	1.066586	-0.23491	-0.415	-0.34811	-0.00045	-0.51582	-1.20126	-0.3831413	-0.57052	2.91268	0.449767	0.896141	0.605612	0.483651	0.392936	-0.13802
b18	-0.72425	1.05988	-0.385	-0.46323	0.461262	-0.51582	0.076029	0.05053862	-0.40894	-0.19445	0.70851	0.393415	-0.93902	-0.73215	-1.75146	-1.5173
b19	-0.21259	0.001302	0.094999	0.227502	0.182089	-0.16667	0.686908	0.17582392	0.301995	-0.19445	0.820543	0.349057	1.08641	0.639941	-0.1765	-0.12295
b20	-0.77542	1.602292	-0.385	-0.0603	1.255833	-0.55073	1.297787	1.41903954	0.140418	-0.15189	0.897899	0.326878	0.825287	0.896659	0.439052	0.444944
b21	-0.67309	1.156114	0.334997	0.572868	1.191408	0.077726	1.186718	0.35893319	0.915988	-0.1306	0.124338	0.363843	1.446318	2.242388	0.242557	0.216121
b22	1.373587	0.849914	0.724993	1.954329	1.008872	0.53161	3.185959	0.02162663	1.206826	-0.1306	0.116336	0.415594	-0.09348	-0.13084	-0.6938	-0.2217
b23	-0.46842	0.578708	0.244998	-0.86615	-0.11856	0.147554	0.853512	-0.113296	0.26968	-0.15189	0.076324	0.445166	0.785911	0.666304	-0.05519	-0.2209
b24	1.117752	-0.39238	1.354986	0.285063	-0.56953	1.544122	-1.20126	-0.2096693	1.303773	-0.17317	-0.070386	-1.19609	0.134484	0.435948	1.446588	0.019817
b25	1.782922	-0.27865	2.464975	0.457746	-0.53732	2.801033	-1.20126	-0.200032	2.499443	-0.17317	-0.105063	-1.21827	1.23286	0.549557	1.932812	0.571054

[a] See Table S4 for the definition of the descriptors.

Table S10. Computationally derived steric and MO coefficients descriptors for the sulfinylamine fragments.[a]

	MO coefficients											
**Sulfinylamine**	**B (Z) α homo-1 S**	**α homo S**	**B (Z) α lumo S**	**B (Z) α lumo+1 S**	**B (Z) α homo-1 N**	**α homo N**	**B (Z) α lumo N**	**α lumo+1 N**	**α homo-1 O**	**α homo O**	**α lumo O**	**α lumo+1 O**
b1	-0.5130459	0.210075	-0.610003003	-0.326761218	-0.391559033	-0.33432972	-0.430852443	-0.394851	-0.38999026	-0.07779	-0.13593	-0.3962751
b2	2.77584643	0.911877	1.645967794	1.570770966	2.794968991	2.899153814	1.824683085	0.4846183	2.77991722	1.398511	0.638757	0.83394072
b3	-0.5517165	0.55825	-1.593742668	0.213511278	-0.406158034	-0.41957159	-1.415000042	0.7813041	-0.43638011	0.01054	-0.7756	-0.1588118
b4	0.70979578	-0.61876	-0.508516793	-0.316403662	0.087741972	-0.7307136	-0.488796528	-0.340267	0.19235045	-0.39954	-0.07343	-0.3845753
b5	0.83170267	-0.00057	-0.417314518	-0.263076248	-0.150478222	0.143797977	-0.821354995	-0.285447	0.83637979	0.046744	0.647011	-0.3743921
b6	-0.5920378	-1.82141	1.543669694	-0.258597305	-0.435651963	-1.39129203	1.830770596	-0.271566	-0.46135167	-1.09366	0.756669	-0.3561924
b7	-0.5988759	0.276519	-1.131777439	-0.059704243	-0.437328875	-0.00993195	-1.07342304	0.3968596	-0.47546603	0.057686	-0.53506	-0.2004112
b8	-0.0532423	0.029122	-0.470357978	-0.283091524	-0.203942134	-0.1712103	-0.486316431	-0.311563	-0.40617736	-0.88978	-3.4805	3.54895212
b9	-0.5842565	-1.91209	1.675737082	-0.332079963	-0.425886415	-1.56380094	1.824457622	-0.415556	-0.45858802	-1.1167	0.803834	-0.3967084
b10	-0.289039	-0.37782	0.45627878	-0.121429676	-0.110725536	0.309126691	0.068774379	0.0997033	-0.21351141	-0.41707	0.657623	-0.2675769
b11	-0.0610236	0.545123	-0.520965768	-0.267975092	-0.052921381	0.285376798	-0.469857606	-0.293682	-0.06220153	0.271357	-0.12767	-0.3804587
b12	-0.4573979	-0.79729	0.215688804	-0.325641482	-0.375776328	0.233826642	-0.417099178	-0.379088	-0.38890454	-0.48387	0.748416	-0.3694088
b13	3.11751584	2.559849	2.314694262	3.998078125	3.618530236	2.703631437	2.226458806	4.0377888	3.47250786	3.585391	1.596793	1.87674256
b14	-0.5962821	0.574608	-0.511493722	-0.316403662	-0.436342456	0.150794069	-0.36839909	-0.338149	-0.47704526	0.22768	-0.16128	-0.3860919
b15	-0.3406785	0.105663	-0.528543405	-0.316683596	-0.325271674	-0.26087075	-0.460839071	-0.313445	-0.27727279	-0.09114	-0.15184	-0.3791587
b16	-0.5960463	0.523917	-0.654386306	-0.317383431	-0.436638382	0.342266077	-0.681342244	-0.328268	-0.47714396	0.254811	-0.16305	-0.3819753
b17	-0.3286529	-0.5513	0.765338119	-0.323961879	-0.122957132	0.623582643	0.38352124	-0.352737	-0.25891425	-0.52737	0.907597	-0.3694088
b18	-0.4439575	0.356494	-0.731515825	-0.193512665	-0.367293125	0.150794069	-0.899590784	-0.020289	-0.40903971	0.146375	-0.21198	-0.2771101
b19	0.19269362	-0.23928	-0.581316234	-0.317383431	-0.164978582	-0.55157681	-0.568836024	-0.33862	-0.10128745	-0.23916	-0.13357	-0.3767754
b20	-0.4163692	0.411831	-0.614062451	-0.319063035	-0.368674111	-0.16439831	-0.45610434	-0.367324	-0.40114356	0.088198	-0.19016	-0.3858753
b21	1.8640206	-1.6237	-0.506893013	-0.317943299	0.438512578	-1.48224123	-0.381926892	-0.321915	0.91386071	-1.02953	-0.13887	-0.3811087
b22	0.06842876	-0.13244	-0.56724348	-0.317243464	-0.181155854	-0.50941614	-0.439194588	-0.328268	-0.16149556	-0.1824	-0.18722	-0.3793754
b23	0.06842876	-0.13244	-0.56724348	-0.317243464	-0.181155854	-0.50941614	-0.439194588	-0.328268	-0.16149556	-0.1824	-0.18722	-0.3793754
b24	-0.5851997	-1.91027	1.68250283	-0.336558906	-0.42460407	-1.55864592	1.828966889	-0.430143	-0.45937763	-1.11546	0.787916	-0.3984418
b25	-0.6198618	-1.91936	1.671948264	-0.348176164	-0.454295283	-1.58147528	1.864139174	-0.460729	-0.47980891	-1.12124	0.718938	-0.4038583

[a] See Table S4 for the definition of the descriptors.

Table S11. Computationally derived steric and MO energy descriptors for the sulfinylamine fragments.[a]

	Fukui indices and Mulliken charges								
**Sulfinylamine**	**fukui electrophilic S**	**fukui nucelophilc S**	**fukui ave S**	**fukui electrophili c N**	**B (Z) fukui nucelophilc N**	**B(Z) fukui ave N**	**fukui electrophilic O**	**fukui nucelophilc O**	**fukui ave O**
b1	-0.15678348	-0.6036247	-0.41551	-0.2084029	-0.2812047	-0.26439	-0.2315194	-0.696205104	-0.39208
b2	0.22076954	1.81656882	1.088105	1.41863853	1.7187675	1.708437	0.83888262	1.591110516	1.145035
b3	-0.0643942	-1.6066313	-0.87774	-0.3944712	-1.1187363	-0.77442	-0.2345956	-1.477588665	-0.62576
b4	-0.21789673	-0.4402034	-0.36998	-0.4913602	-0.3936764	-0.49754	-0.39994143	-0.555615184	-0.48585
b5	0.05679449	-0.1896496	-0.06197	0.02317903	-0.6370461	-0.28281	0.08157116	-0.027388073	0.057457
b6	-1.35899984	1.25907263	-0.22355	-1.3581146	1.3083732	-0.25563	-1.06354483	1.168670717	-0.509
b7	-0.01762168	-0.9723189	-0.51739	-0.0439763	-0.8695632	-0.4343	-0.03562921	-0.879086301	-0.28875
b8	0.04990716	-0.3404098	-0.14488	-0.2826518	-0.35311	-0.34537	-0.10793446	-0.475368177	-0.22741
b9	-2.03652908	0.5238236	-1.04429	-1.3008987	0.9543599	-0.38449	-1.43924769	0.768657514	-0.92932
b10	0.0717249	0.19689321	0.148844	0.09666339	-0.0640958	0.031755	-0.33306307	0.392016843	-0.1517
b11	0.37080482	-0.2377733	0.116037	0.15553583	-0.3113538	-0.04618	0.27650366	-0.391219178	0.106474
b12	-0.30865853	0.00844296	-0.19519	0.22235138	-0.7658837	-0.21587	-0.337866	0.280120352	-0.18867
b13	3.24822254	3.40576414	3.872778	3.64166784	3.302658	3.867292	3.69024566	3.266798892	3.932584
b14	0.31608358	-0.3029542	0.046732	0.06503963	-0.2481537	-0.07442	0.20379213	-0.416838311	0.040453
b15	-0.02866028	-0.5036845	-0.28065	-0.2342286	-0.3729289	-0.32372	-0.19718783	-0.571026078	-0.32745
b16	0.19331457	-0.5114218	-0.14114	0.1824447	-0.540592	-0.13609	0.1874099	-0.57571635	-0.01972
b17	-0.13892831	0.2781642	0.054922	0.13761074	-0.0014181	0.087172	-0.5030232	0.593462058	-0.22869
b18	-0.02866028	-0.7205048	-0.39349	-0.1943219	-1.0322352	-0.60626	0.05643918	-0.671216596	-0.15325
b19	-0.28035443	-0.6423991	-0.51559	-0.7454546	-0.6100308	-0.76079	-0.23043112	-0.630620123	-0.3718
b20	0.05865785	-0.5718841	-0.25968	-0.385891	-0.4945412	-0.47734	-0.01321067	-0.618795908	-0.19372
b21	-0.88332597	-0.5074359	-0.83526	-1.149831	-0.3555475	-0.90001	-1.00818771	-0.537208823	-0.96927
b22	0.0699559	-0.3559723	-0.14001	-0.1416511	-0.3851453	-0.27034	-0.15367119	-0.461139704	-0.25996
b23	-0.1905597	-0.597089	-0.43395	-0.4400485	-0.3535162	-0.44602	-0.32866642	-0.669640034	-0.4623
b24	-1.83710316	0.69571484	-0.82588	-1.2233154	1.0410641	-0.29447	-1.32567492	0.882485293	-0.80436
b25	-1.98244467	0.48393546	-1.03007	-0.529674	1.0529613	0.153826	-1.11846663	0.761090016	-0.67374

[a] See Table S4 for the definition of the descriptors.

Optimized structures

Me

E(UPW6B95D3) = -39.8998234998

Charge = 0 Multiplicity = 2

Single point geometry:

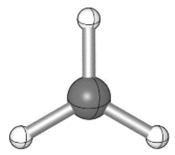

C	-1.36514	0.14095	0.21838
H	-0.85706	-0.75755	-0.09246
H	-0.85709	0.85943	0.84108
H	-2.38124	0.32102	-0.09335

PhNSO (*Z*)

E(RPW6B95D3) = -760.743096375

Charge = 0 Multiplicity = 1

Single point geometry:

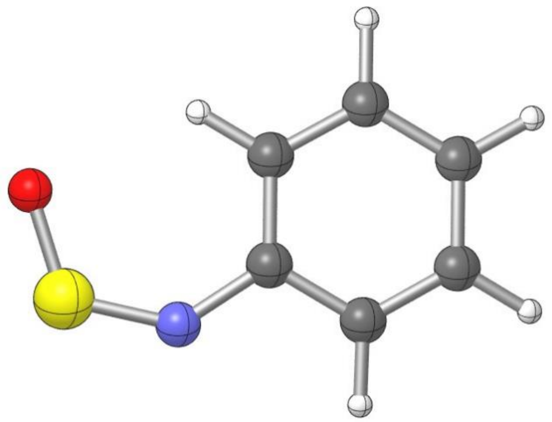

S	2.78324	2.18439	0.89753
N	2.25117	0.82538	0.55561
O	1.87357	3.30968	1.01858
C	-1.41101	0.48208	0.0501
C	-1.47733	-0.87741	-0.23923
C	-0.31541	-1.64395	-0.25998
C	0.90724	-1.05158	0.00766
C	0.97679	0.315	0.29861
C	-0.19234	1.08676	0.31974
H	-2.31551	1.07692	0.06586
H	-2.43416	-1.33925	-0.44825
H	-0.3646	-2.70181	-0.48453
H	1.82527	-1.62561	-0.00193
H	-0.14018	2.14217	0.54482

PhNSO (*E*)

E(RPW6B95D3) = -760.734055004

Charge = 0 Multiplicity = 1

Single point geometry:

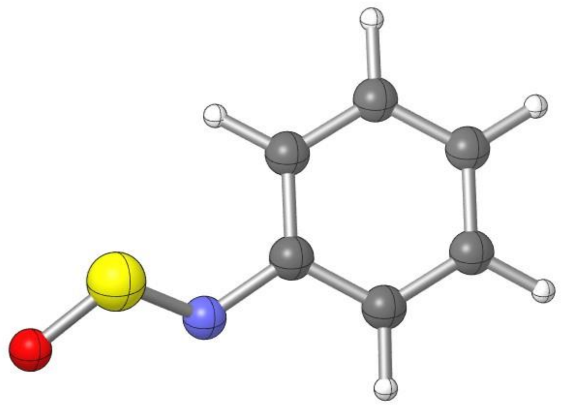

S	2.71576	2.13425	0.42583
N	2.27334	0.74647	0.81999
O	4.00534	2.49894	0.9593
C	-1.36137	0.51119	0.07414
C	-1.44464	-0.82126	-0.31441
C	-0.30326	-1.61776	-0.30651
C	0.91699	-1.08666	0.08226
C	1.00417	0.25822	0.44387
C	-0.14327	1.05497	0.45699
H	-2.25018	1.12931	0.09164
H	-2.39836	-1.24215	-0.60639
H	-0.3654	-2.65931	-0.59569
H	1.81382	-1.6929	0.10059
H	-0.08581	2.08275	0.79649

TSA
E(UPW6B95D3) = -800.648249287
Charge = 0 Multiplicity = 2
Single point geometry:

C	-0.85031	-4.21169	0.36239
C	-0.53608	-3.18201	-0.51304
C	-0.75259	-1.85277	-0.12236
C	-1.28468	-1.58199	1.14437
C	-1.60099	-2.61849	2.00627
C	-1.38346	-3.93784	1.6178
H	-0.67968	-5.23723	0.05926
H	-0.12789	-3.39814	-1.48969
H	-1.44084	-0.54837	1.42739
H	-2.01465	-2.39959	2.98266
H	-1.62858	-4.74955	2.29118
N	-0.45661	-0.74549	-0.91663
S	0.07231	-0.59342	-2.31924
O	0.25882	-1.7763	-3.14563
C	2.62155	-0.32412	-1.67416
H	2.53917	0.53293	-1.02395
H	2.6325	-1.31411	-1.24204
H	2.97132	-0.18984	-2.68627

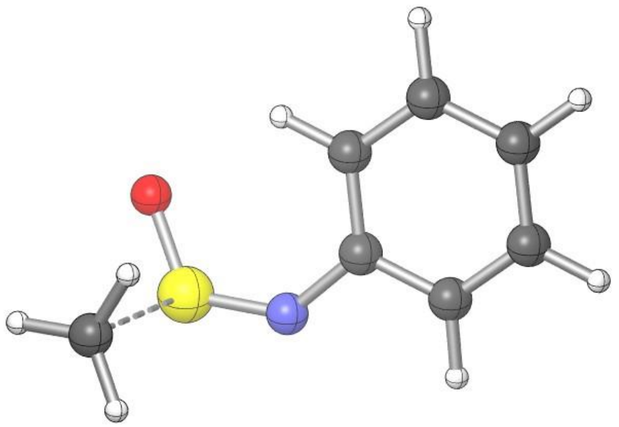

TSB
E(UPW6B95D3) = -800.634378242
Charge = 0 Multiplicity = 2
Single point geometry:

C	-1.53316	3.10599	-0.35349
C	-0.85977	2.04349	-0.94344
C	-0.99649	0.76166	-0.40624
C	-1.78763	0.55962	0.72651
C	-2.46202	1.62464	1.30218
C	-2.33656	2.90194	0.76254
H	-1.42609	4.10001	-0.76958
H	-0.22756	2.20339	-1.80496
H	-1.86396	-0.44078	1.13499
H	-3.08207	1.46007	2.17448
H	-2.85872	3.73575	1.21478
N	-0.35268	-0.38385	-0.93053
S	-0.04919	-0.71671	-2.39965
O	-0.04834	0.3756	-3.37371
C	1.56948	-0.20505	0.25588
H	1.11747	-0.18383	1.2357
H	2.03625	-1.12106	-0.07263
H	1.88994	0.7276	-0.18457

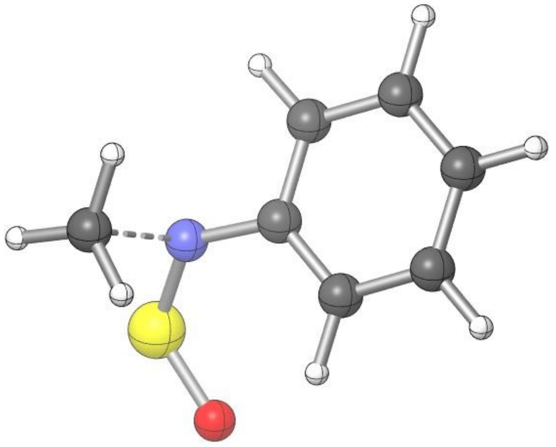

10a

E(UPW6B95D3) = -800.685602803

Charge = 0 Multiplicity = 2

Single point geometry:

S	2.58905	2.24412	1.59679
N	2.25952	0.77765	1.01335
O	1.84984	3.37927	1.01432
C	-1.2663	0.55357	-0.07349
C	-1.31088	-0.80335	-0.39958
C	-0.17212	-1.59752	-0.23907
C	0.99244	-1.04268	0.24241
C	1.06232	0.33893	0.56831
C	-0.10615	1.13133	0.40098
H	-2.15292	1.16356	-0.19356
H	-2.22786	-1.23981	-0.77407
H	-0.20632	-2.65065	-0.48789
H	1.88665	-1.63758	0.38091
H	-0.0764	2.18283	0.64588
C	4.24613	2.35226	0.95196
H	4.82991	1.51149	1.32013
H	4.18385	2.34451	-0.13607
H	4.6603	3.29382	1.30841

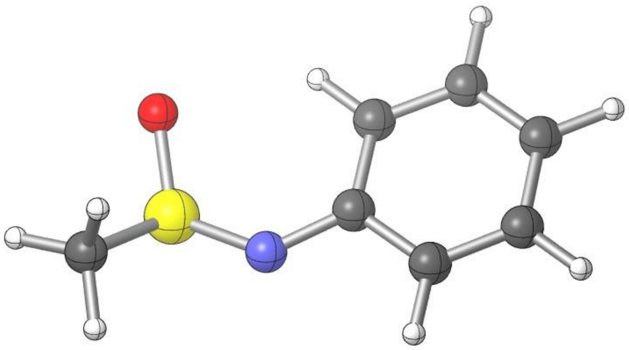

10b

E(UPW6B95D3) = -800.681894425

Charge = 0 Multiplicity = 2

Single point geometry:

C	-2.45706	2.14891	-0.74143
C	-1.39393	1.33156	-1.05421
C	-0.31006	1.1854	-0.14128
C	-0.3746	1.88456	1.09627
C	-1.44198	2.70378	1.38827
C	-2.48793	2.84393	0.47218
H	-3.27985	2.24759	-1.43812
H	-1.39695	0.77563	-1.98386
H	0.44832	1.75599	1.7882
H	-1.47275	3.23662	2.32998
H	-3.33016	3.48226	0.70571
N	0.79433	0.44546	-0.34985 q
S	0.99949	-0.39981	-1.75539
O	2.17508	-1.25923	-1.5686
C	1.54411	0.97418	-2.76088
H	0.74082	1.70394	-2.85902
H	2.41543	1.4117	-2.27306
H	1.81128	0.57044	-3.73682

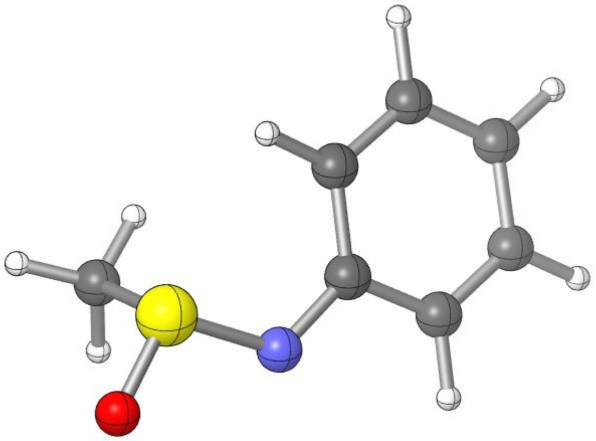

11a

E(UPW6B95D3) = -800.709415754

Charge = 0 Multiplicity = 2

Single point geometry:

C	-2.04929	2.27949	-0.89608
C	-0.88952	1.5375	-1.05029
C	-0.07423	1.27401	0.05609
C	-0.44583	1.76556	1.30932
C	-1.61429	2.505	1.45014
C	-2.42171	2.76994	0.35254
H	-2.67104	2.47105	-1.76203
H	-0.62057	1.16044	-2.02832
H	0.17513	1.5769	2.17484
H	-1.88764	2.87723	2.42989
H	-3.32955	3.3486	0.46531
N	1.09352	0.50023	-0.07352
S	1.89751	0.30312	-1.50118
O	1.91303	1.57038	-2.25654
C	1.73128	-0.08314	1.09939
H	2.23609	0.67718	1.70022
H	0.98912	-0.59776	1.70979
H	2.47483	-0.80965	0.77058

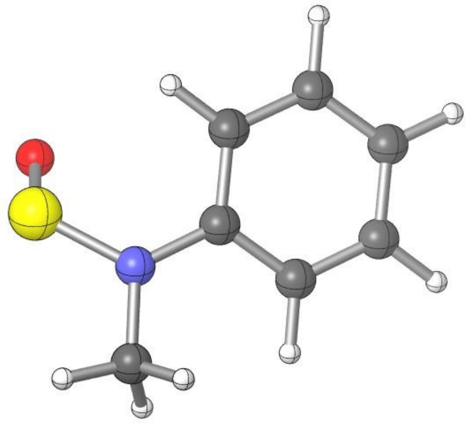

11b

E(UPW6B95D3) = -800.711209470

Charge = 0 Multiplicity = 2

Single point geometry:

S	2.58705	2.30805	0.37207
N	2.30894	0.69403	0.63692
O	3.76475	2.69803	1.16968
C	-1.37621	0.4785	0.34222
C	-1.5362	-0.78999	-0.20573
C	-0.41124	-1.56379	-0.46355
C	0.86178	-1.07755	-0.19553
C	1.02164	0.19783	0.3524
C	-0.11195	0.96973	0.63112
H	-2.24333	1.08717	0.56709
H	-2.52574	-1.17321	-0.41939
H	-0.51927	-2.55592	-0.88444
H	1.72752	-1.68727	-0.41716
H	-0.00353	1.94016	1.09925
C	3.45291	-0.20304	0.69827
H	3.21942	-1.03832	1.35789
H	3.72106	-0.58504	-0.2906
H	4.29782	0.34216	1.11288

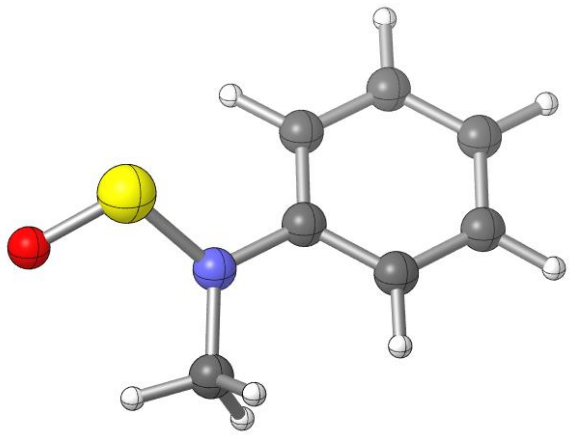

X-Ray crystallographic data

N-Phenylcyclohexanesulfinamide (1a)

CCDC 2170582

Bond precision:	C–C = 0.0023 A		Wavelength = 1.54184	
Cell:	a = 6.1241(2)	b = 8.1468(2)	c = 11.8684(4)	
	α = 95.121(3)	β = 91.181(3)	γ = 105.039(3)	
Temperature:	100 K			

	Calculated	Reported
Volume	568.98(3)	568.98(3)
Space group	P -1	P -1
Hall group	-P 1	-P 1
Moiety formula	$C_{12}H_{17}NOS$	$C_{12}H_{17}NOS$
Sum formula	$C_{12}H_{17}NOS$	$C_{12}H_{17}NOS$
Mr	223.33	223.32
D_x,g cm^{-3}	1.304	1.304
Z	2	2
Mu (mm^{-1})	2.298	2.298
F000	240.0	240.0
F000'	241.23	
h,k,l_{max}	7,10,14	7,10,14
N_{ref}	2372	2266
T_{min},T_{max}	0.750,0.867	0.491,1.000
T_{min}'	0.633	

Correction method = # Reported T Limits: T_{min} = 0.491 T_{max} = 1.000 AbsCorr = GAUSSIAN

Data completeness = 0.955 Theta(max) = 75.928

R(reflections) = 0.0380(2127) wR2(reflections) = 0.0985(2266)

S = 1.048 N_{par} = 139

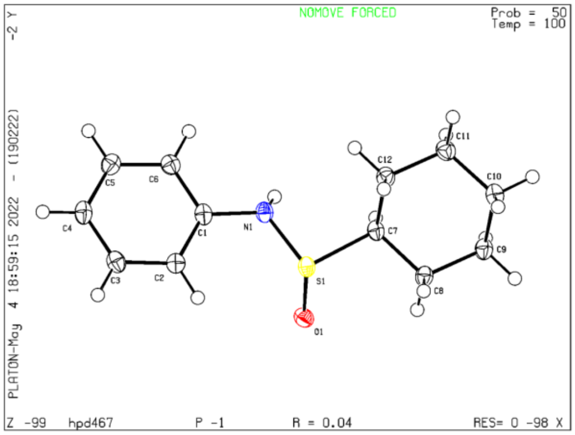

N-Phenyltetrahydro-2H-pyran-4-sulfinamide (1n)
CCDC 2170572

Bond precision:	C–C = 0.0019 A		Wavelength = 1.54184
Cell:	a = 5.99937(10)	b = 7.77287(15)	c = 11.90456(17)
	α = 90.8904(14)	β = 95.3034(13)	γ = 100.9668(15)
Temperature:	100 K		

	Calculated	Reported
Volume	542.355(16)	542.355(16)
Space group	P -1	P -1
Hall group	-P 1	-P 1
Moiety formula	$C_{11}H_{15}$ NO_2S	$C_{11}H_{15}$ NO_2S
Sum formula	$C_{11}H_{15}$ NO_2S	$C_{11}H_{15}$ NO_2S
Mr	225.30	225.30
D_x,g cm^{-3}	1.380	1.380
Z	2	2
Mu (mm^{-1})	2.489	2.489
F000	240.0	240.0
F000'	241.30	
h,k,l_{max}	7,9,14	7,9,14
N_{ref}	2265	2156
T_{min},T_{max}	0.714,0.859	0.367,1.000
T_{min}'	0.558	

Correction method = # Reported T Limits: T_{min}=0.367 T_{max}=1.000 AbsCorr = GAUSSIAN

Data completeness= 0.952 Theta(max)= 75.948

R(reflections)= 0.0321(2062) wR2(reflections)= 0.0835(2156)

S = 1.087 N_{par} = 140

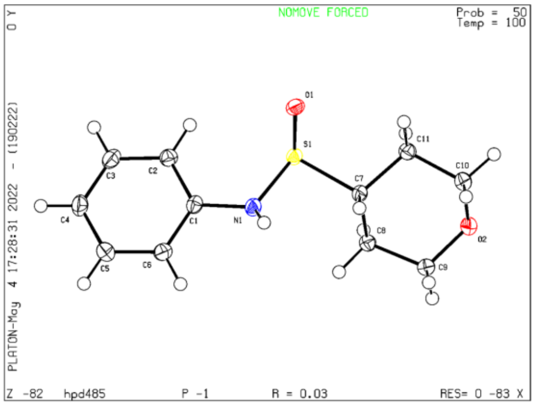

Go to table of contents

Methyl 4-((phenylamino)sulfinyl)bicyclo[2.2.2]octane-1-carboxylate (1t)

CCDC 2170584

Bond precision:	C–C = 0.0023 A	Wavelength = 1.54184
Cell:	a = 5.8837(1) b = 9.3010(1) c = 14.8817(2)	
	α = 72.840(1) β = 81.889(1) γ = 75.062(1)	
Temperature:	100 K	
	Calculated	Reported
Volume	749.973(19)	749.973(19)
Space group	P -1	P -1
Hall group	-P 1	-P 1
Moiety formula	$C_{16}H_{21}NO_3S$	$C_{16}H_{21}NO_3S$
Sum formula	$C_{16}H_{21}NO_3S$	$C_{16}H_{21}NO_3S$
Mr	307.40	307.40
D_x,g cm^{-3}	1.361	1.361
Z	2	2
Mu (mm^{-1})	2.002	2.002
F000	328.0	328.0
F000'	329.56	
h,k,l_{max}	7,11,18	7,11,18
N_{ref}	3155	2989
T_{min},T_{max}	0.766,0.883	0.388,1.000
T_{min}'	0.663	

Correction method = # Reported T Limits: T_{min} = 0.388 T_{max} = 1.000 AbsCorr = GAUSSIAN

Data completeness = 0.947 Theta(max) = 76.355

R(reflections) = 0.0384(2788) wR2(reflections) = 0.1014(2989)

S = 1.070 N_{par} = 192

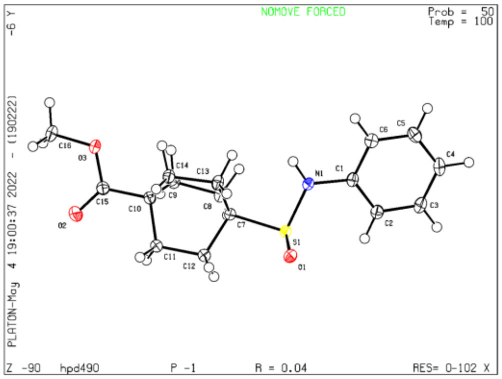

Go to table of contents

N-(6-Methylpyridin-2-yl)cyclopent-3-ene-1-sulfinamide (4e)
CCDC 2176484

Bond precision:	C–C = 0.0029 A		Wavelength = 1.54184
Cell:	a = 6.3363(2)	b = 21.0331(7)	c = 8.3324(2)
	α = 90	β = 95.395(3)	γ = 90
Temperature:	100 K		

	Calculated	Reported
Volume	1105.56(6)	1105.55(6)
Space group	P 21/n	P 1 21/n 1
Hall group	-P 2yn	-P 2yn
Moiety formula	$C_{11}H_{14}N_2OS$	$C_{11}H_{14}N_2OS$
Sum formula	$C_{11}H_{14}N_2OS$	$C_{11}H_{14}N_2OS$
Mr	222.30	222.30
D_x,g cm^{-3}	1.336	1.336
Z	4	4
Mu (mm^{-1})	2.394	2.394
F000	472.0	472.0
F000'	474.50	
h,k,l_{max}	7,26,10	7,26,10
N_{ref}	2315	2229
T_{min},T_{max}	0.744,0.862	0.804,1.000
T_{min}'	0.615	

Correction method = # Reported T Limits: T_{min} = 0.804 T_{max} = 1.000 AbsCorr = GAUSSIAN

Data completeness = 0.963 Theta(max) = 76.442

R(reflections) = 0.0417(2070) wR2(reflections) = 0.1050(2229)

S = 1.059 N_{par} = 137

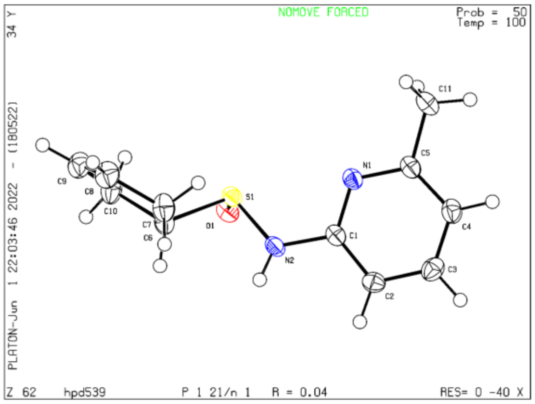

Go to table of contents

N-(5-Bromopyrimidin-2-yl)-5-chloropentane-1-sulfinamide (4f)

CCDC 2170581

Bond precision: C–C = 0.0123 A Wavelength = 1.54184

Cell: a = 5.5464(6) b = 11.0627(10) c = 11.4651(8)

α = 70.025(7) β = 84.920(8) γ = 76.652(9)

Temperature: 100 K

	Calculated	Reported
Volume	643.26(11)	643.26(11)
Space group	P -1	P -1
Hall group	-P 1	-P 1
Moiety formula	$C_9H_{13}BrClN_3O_2S$	$C_9H_{13}BrClN_3O_2S$
Sum formula	$C_9H_{13}BrClN_3O_2S$	$C_9H_{13}BrClN_3O_2S$
Mr	342.63	342.64
D_x,g cm^{-3}	1.769	1.769
Z	2	2
Mu (mm^{-1})	7.759	7.759
F000	344.0	344.0
F000'	344.73	
h,k,l_{max}	6,13,13	6,13,13
N_{ref}	2445	2379
T_{min},T_{max}	0.513,0.572	0.632,1.000
T_{min}'	0.191	

Correction method = # Reported T Limits: T_{min} = 0.632 T_{max} = 1.000 AbsCorr = GAUSSIAN

Data completeness = 0.973 Theta(max) = 69.977

R(reflections) = 0.0790(2011) wR2(reflections) = 0.1791(2379)

S = 1.085 N_{par} = 157

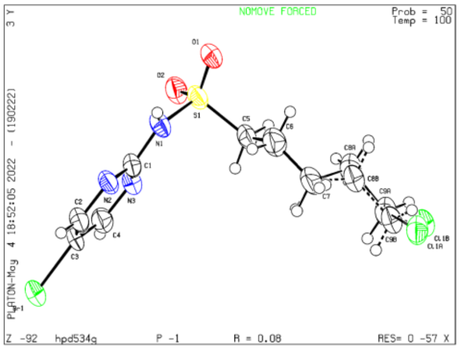

Go to table of contents

(6a*S*,6b*R*,8a*R*,10*S*,12a*R*,12b*R*,14b*S*)-10-Hydroxy-2,2,6a,6b,9,9,12a-heptamethyl-*N*-phenyl-1,3,4,5,6,6a,6b,7,8,8a,9,10,11,12,12a,12b,13,14b-octadecahydropicene-4a(2*H*)-sulfinamide (7k)

CCDC 2170585

Bond precision: C–C = 0.0079 A Wavelength = 1.54184

Cell: a = 14.1503(2) b = 19.7477(4) c = 25.7609(4)

α = 90 β = 90 γ = 90

Temperature: 100 K

	Calculated	Reported
Volume	7198.5(2)	7198.51(19)
Space group	P 21 21 21	P 21 21 21
Hall group	P 2ac 2ab	P 2ac 2ab
Moiety formula	$C_{35}H_{53}NO_2S$	$C_{35}H_{53}NO_2S$
Sum formula	$C_{35}H_{53}NO_2S$	$C_{35}H_{53}NO_2S$
Mr	551.84	551.84
D_x,g cm^{-3}	1.018	1.018
Z	8	8
Mu (mm^{-1})	0.993	0.993
F000	2416.0	2416.0
F000'	2424.48	
h,k,l_{max}	17,24,32	17,24,32
N_{ref}	15126[8263]	14140
T_{min},T_{max}	0.919,0.947	0.521,1.000
T_{min}'	0.808	

Correction method = # Reported T Limits: T_{min} = 0.521 T_{max} = 1.000 AbsCorr = GAUSSIAN

Data completeness = 1.71/0.93 Theta(max) = 76.474

R(reflections) = 0.0698(13023) wR2(reflections) = 0.1577(14140)

S = 1.062 N_{par} = 721

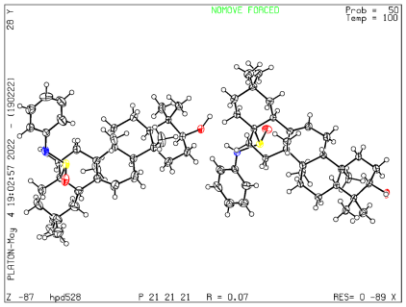

NMR Spectroscopic data

***N*-Phenylcyclohexanesulfinamide (1a)**

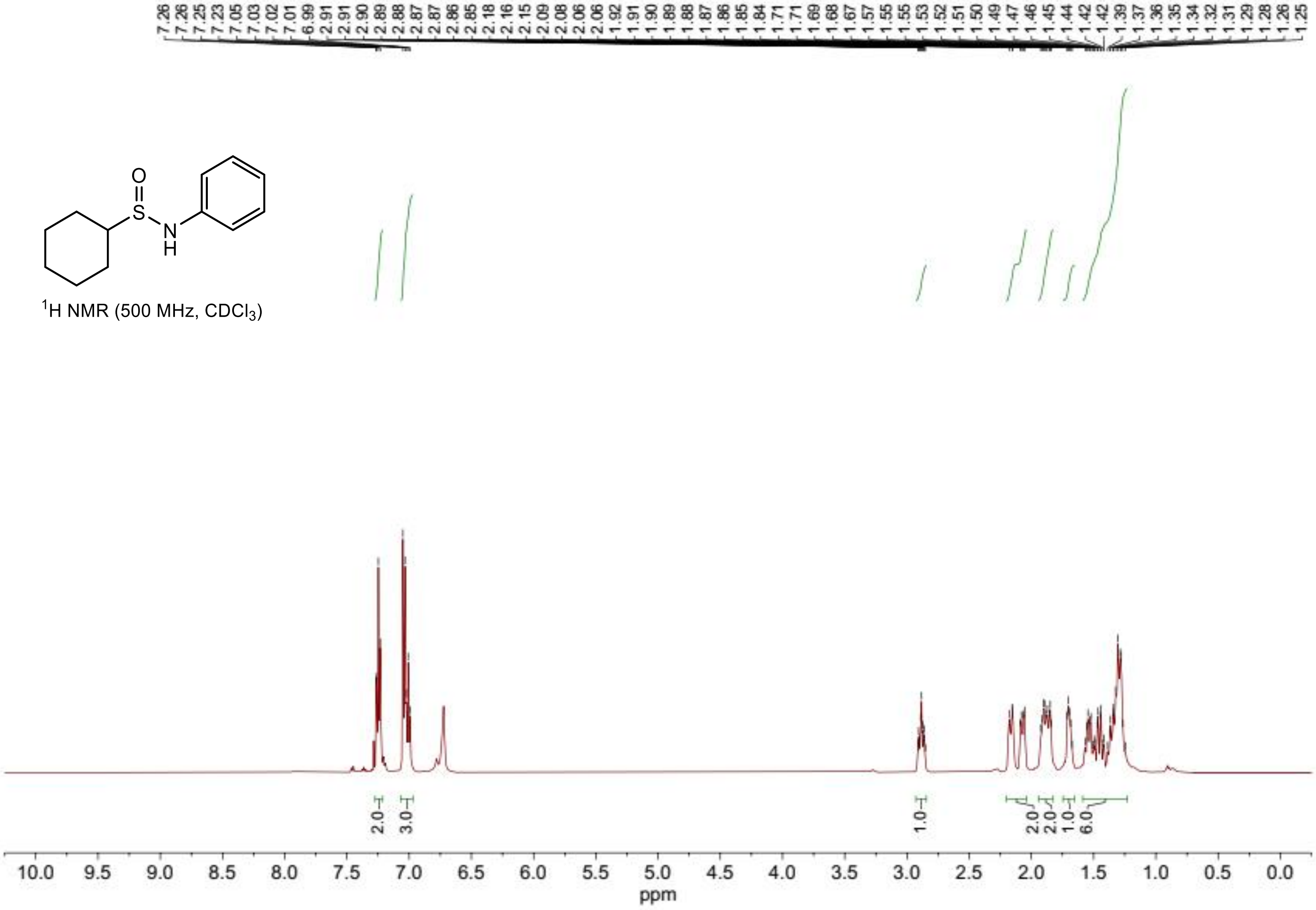

Go to table of contents

N-Phenylcyclohexanesulfinamide (1a)

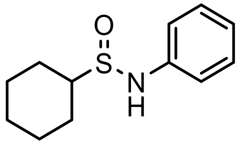

^{13}C NMR (125 MHz, $CDCl_3$)

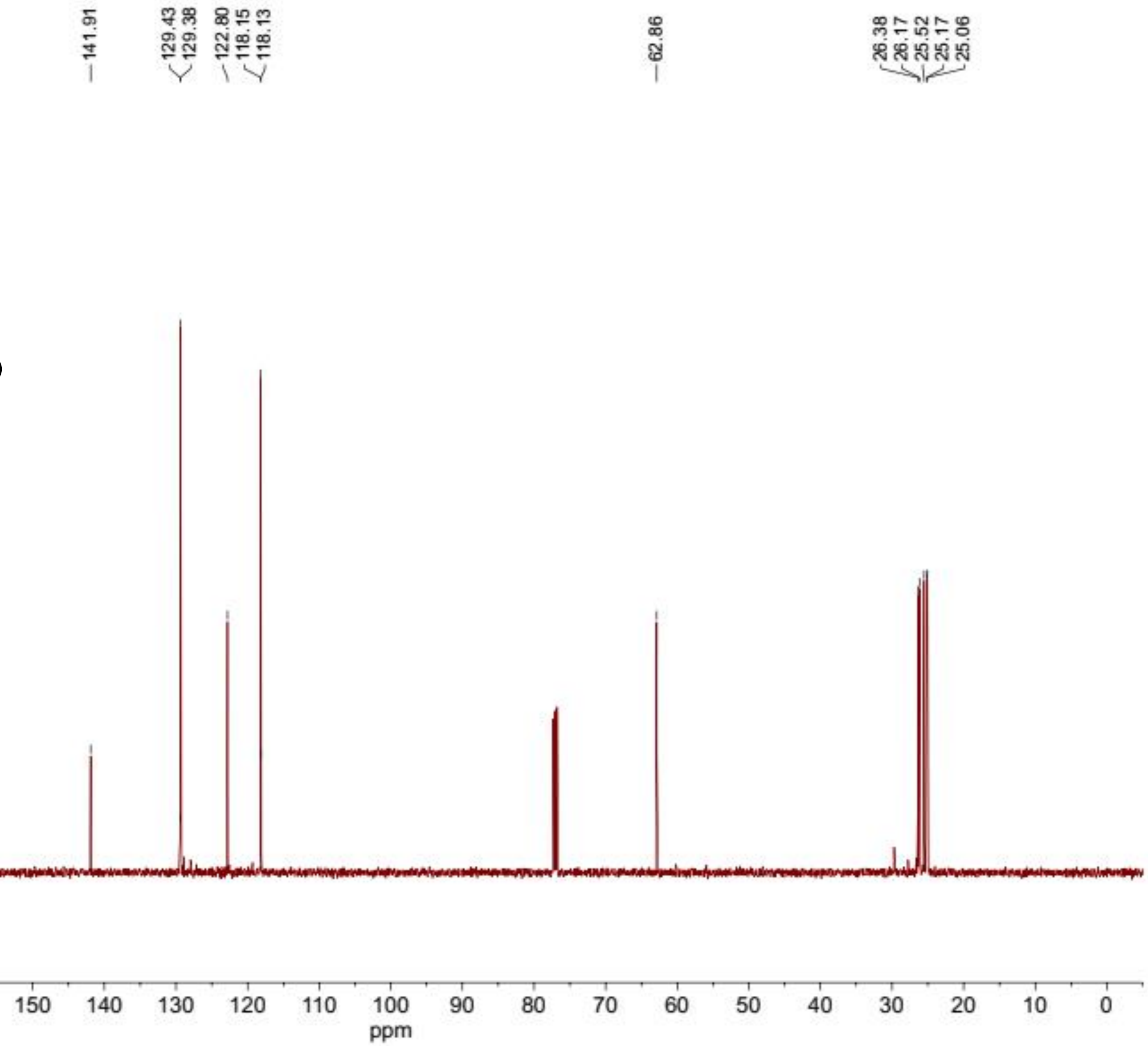

_N_-Phenylnonane-1-sulfinamide (1b)

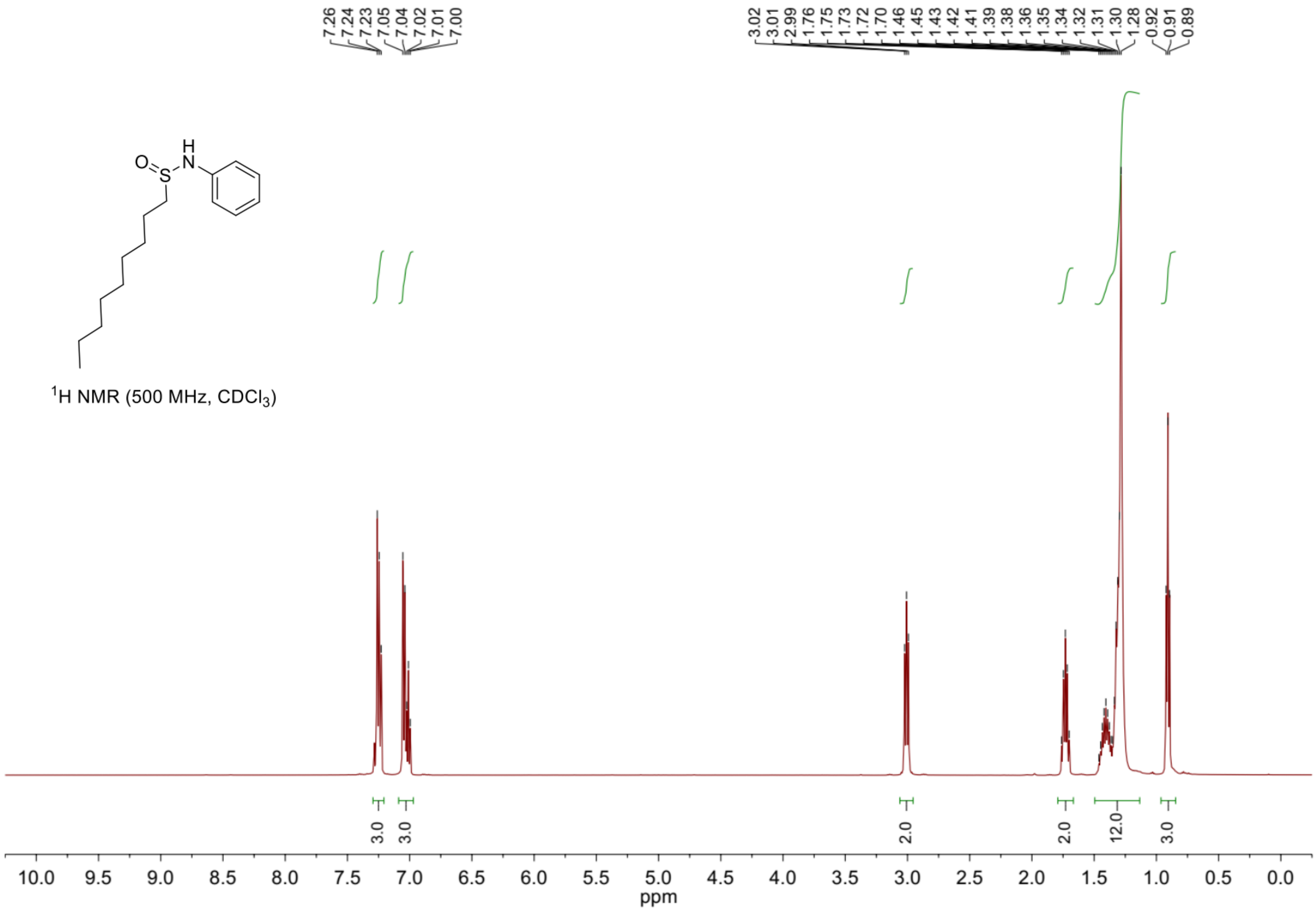

Go to table of contents

N-Phenylnonane-1-sulfinamide (1b)

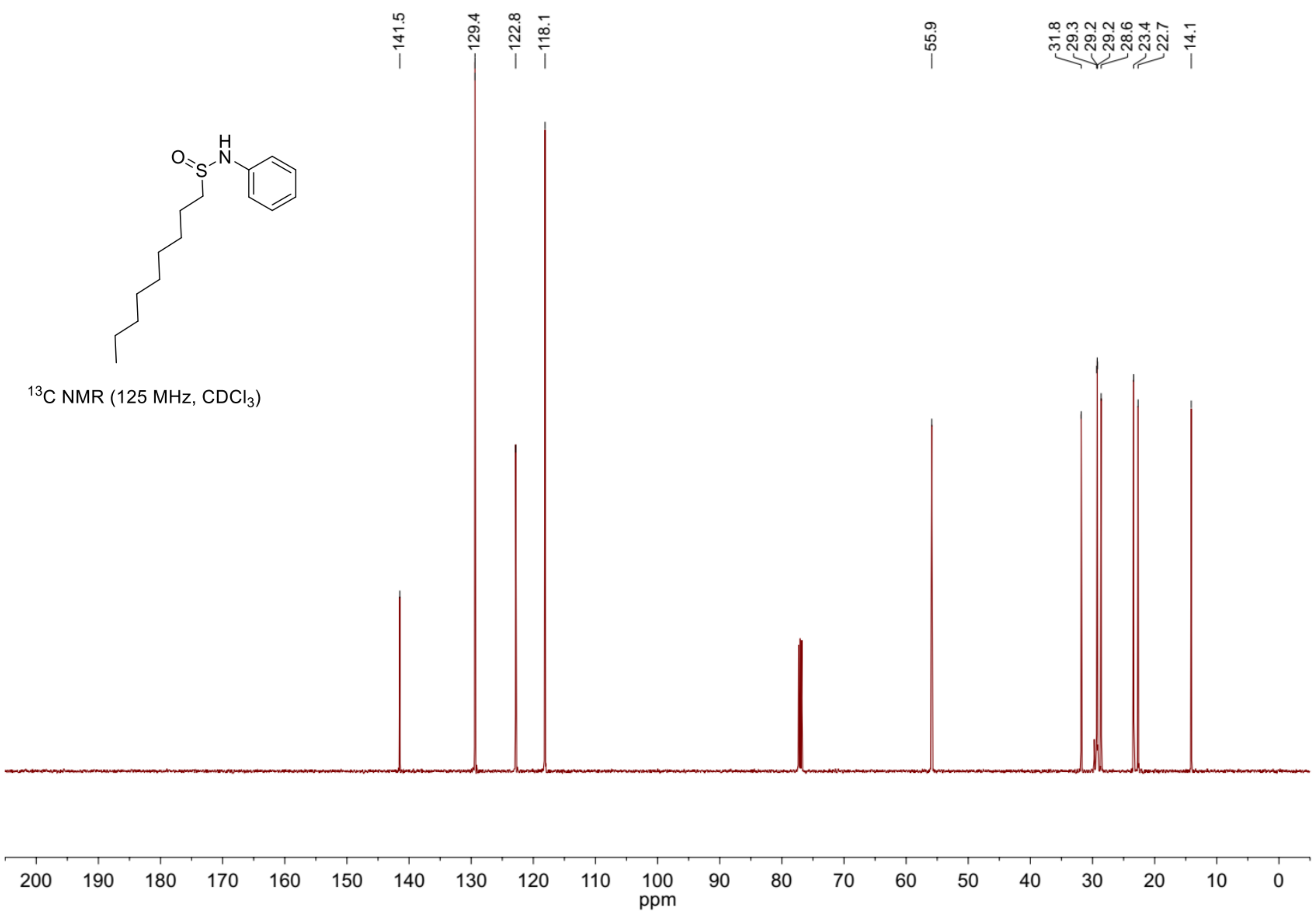

Go to table of contents

1-Cyclopentyl-*N*-phenylmethanesulfinamide (1c)

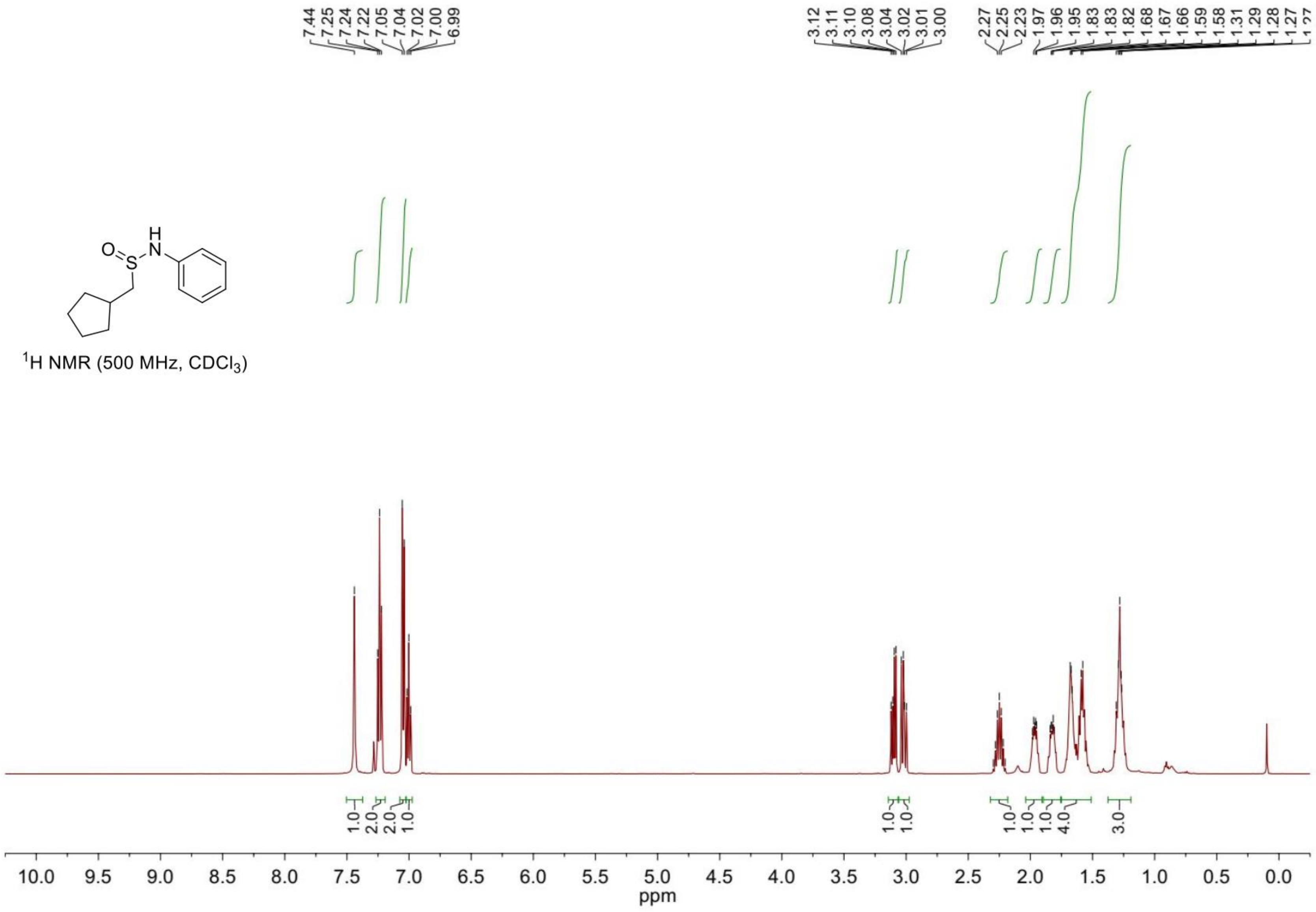

^{1}H NMR (500 MHz, $CDCl_3$)

Go to table of contents

1-Cyclopentyl-*N*-phenylmethanesulfinamide (1c)

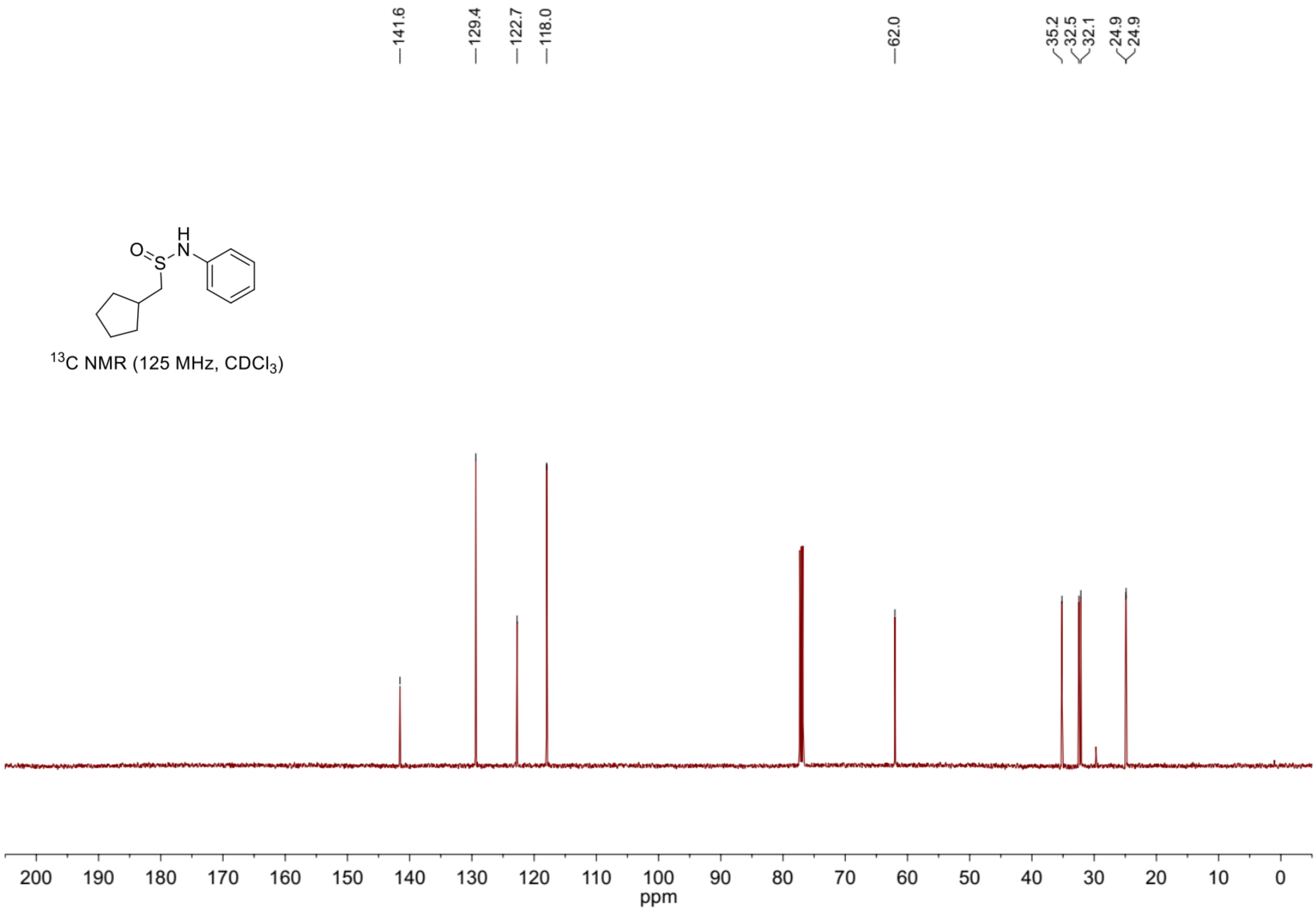

Go to table of contents

5-Chloro-*N*-phenylpentane-1-sulfinamide (1d)

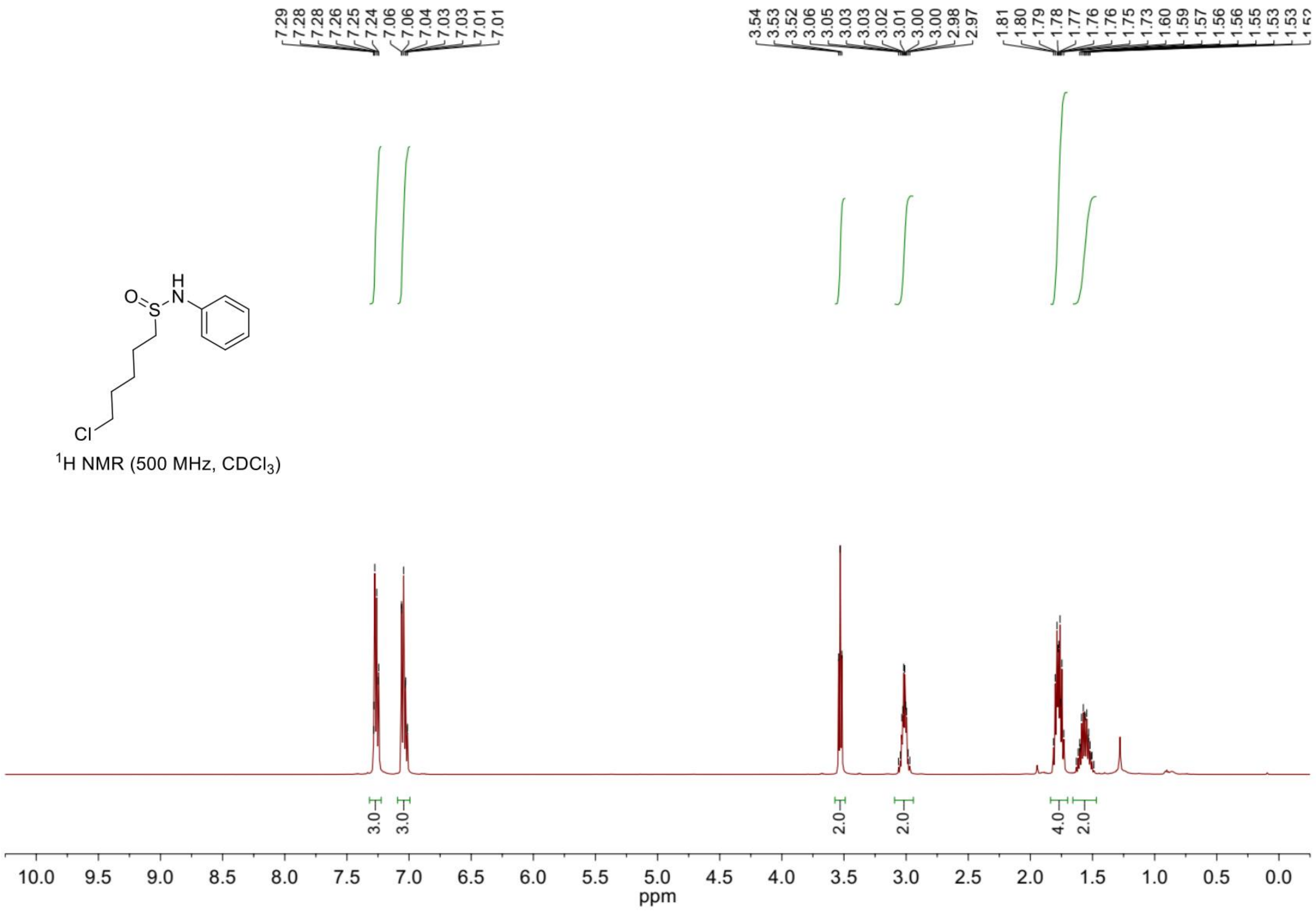

Go to table of contents

5-Chloro-*N*-phenylpentane-1-sulfinamide (1d)

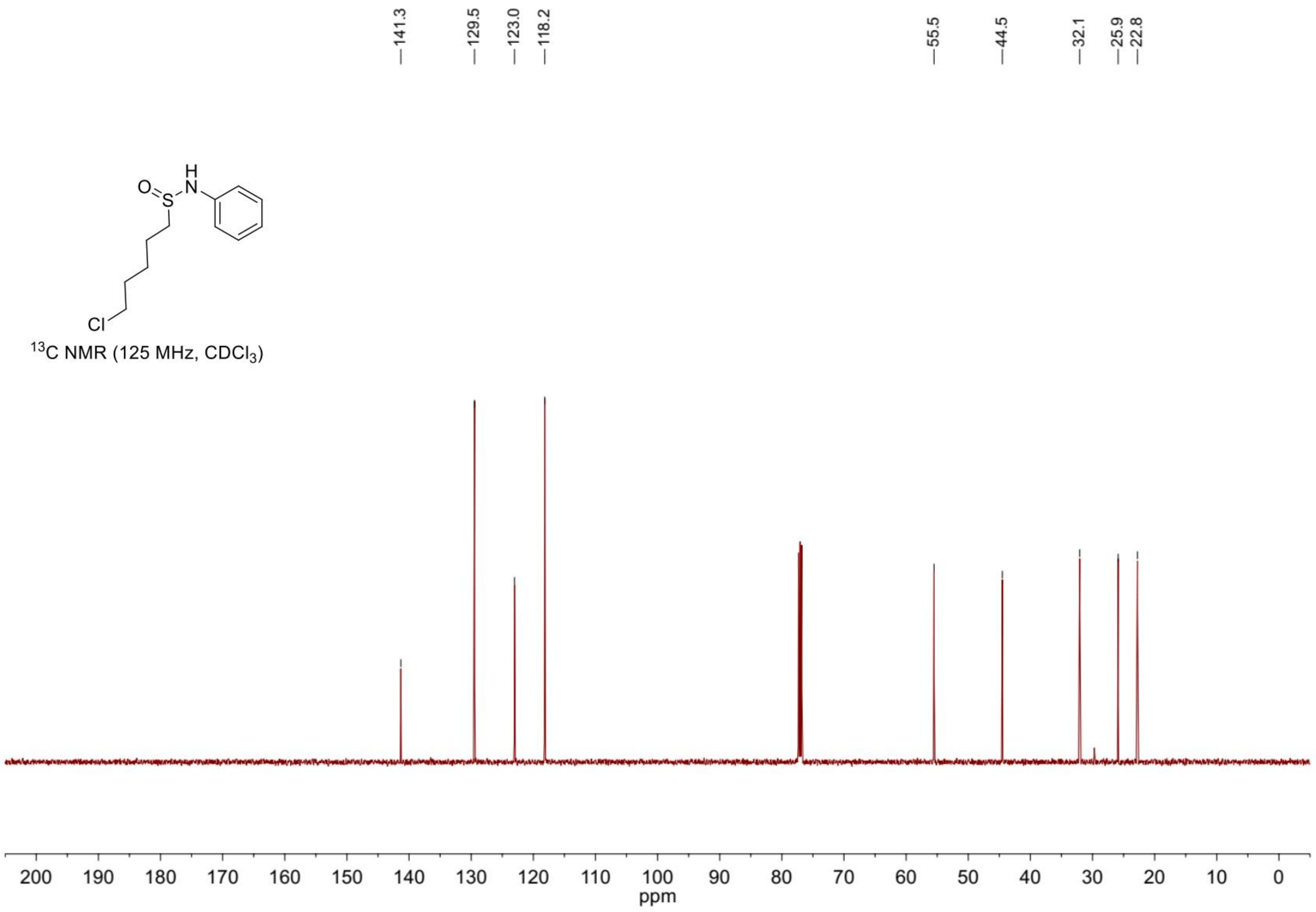

Go to table of contents

5-Bromo-*N*-phenylpentane-1-sulfinamide (1e)

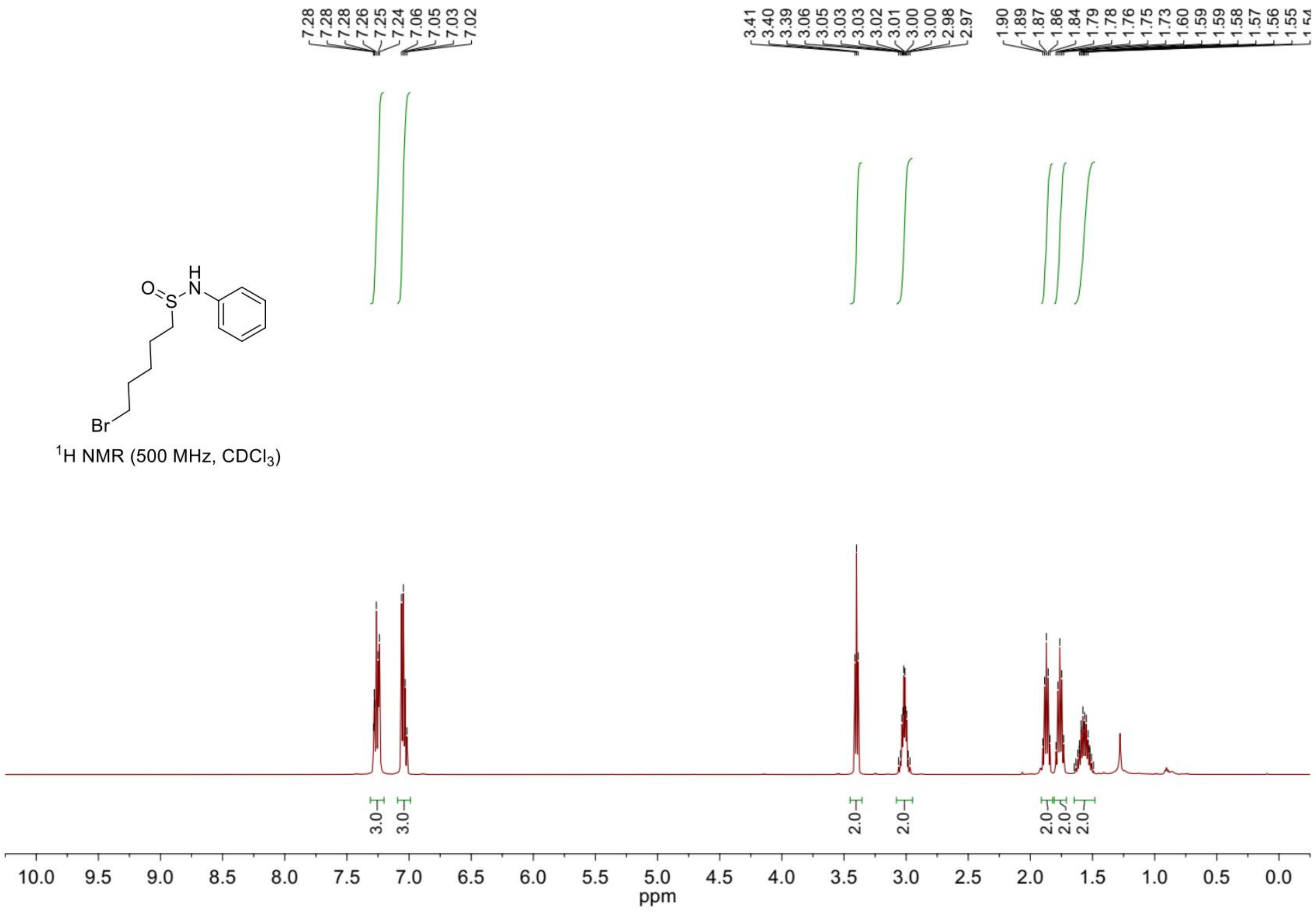

^{1}H NMR (500 MHz, $CDCl_3$)

Go to table of contents

5-Bromo-*N*-phenylpentane-1-sulfinamide (1e)

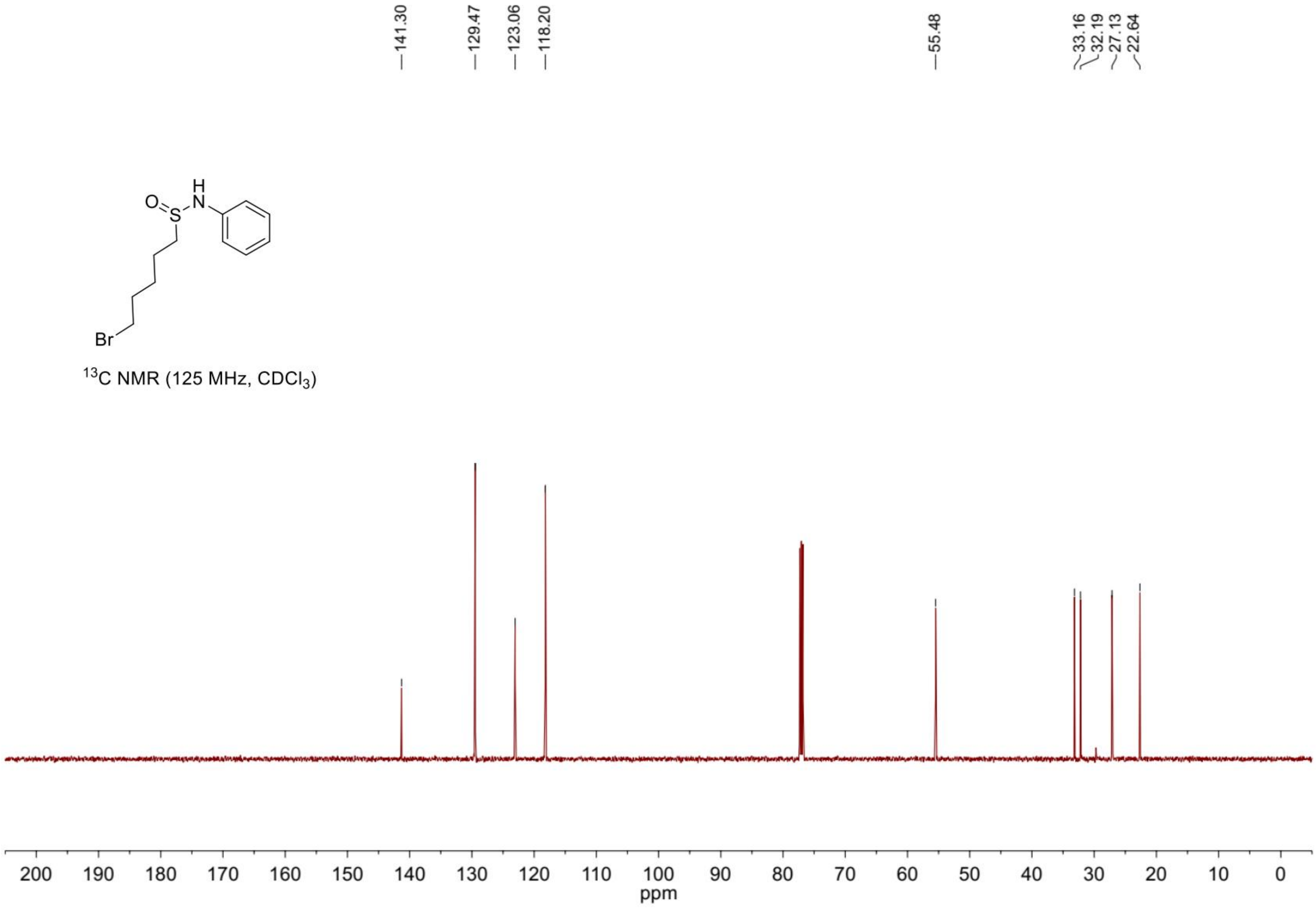

Go to table of contents

2-(4-Fluorophenyl)-*N*-phenylethane-1-sulfinamide (1f)

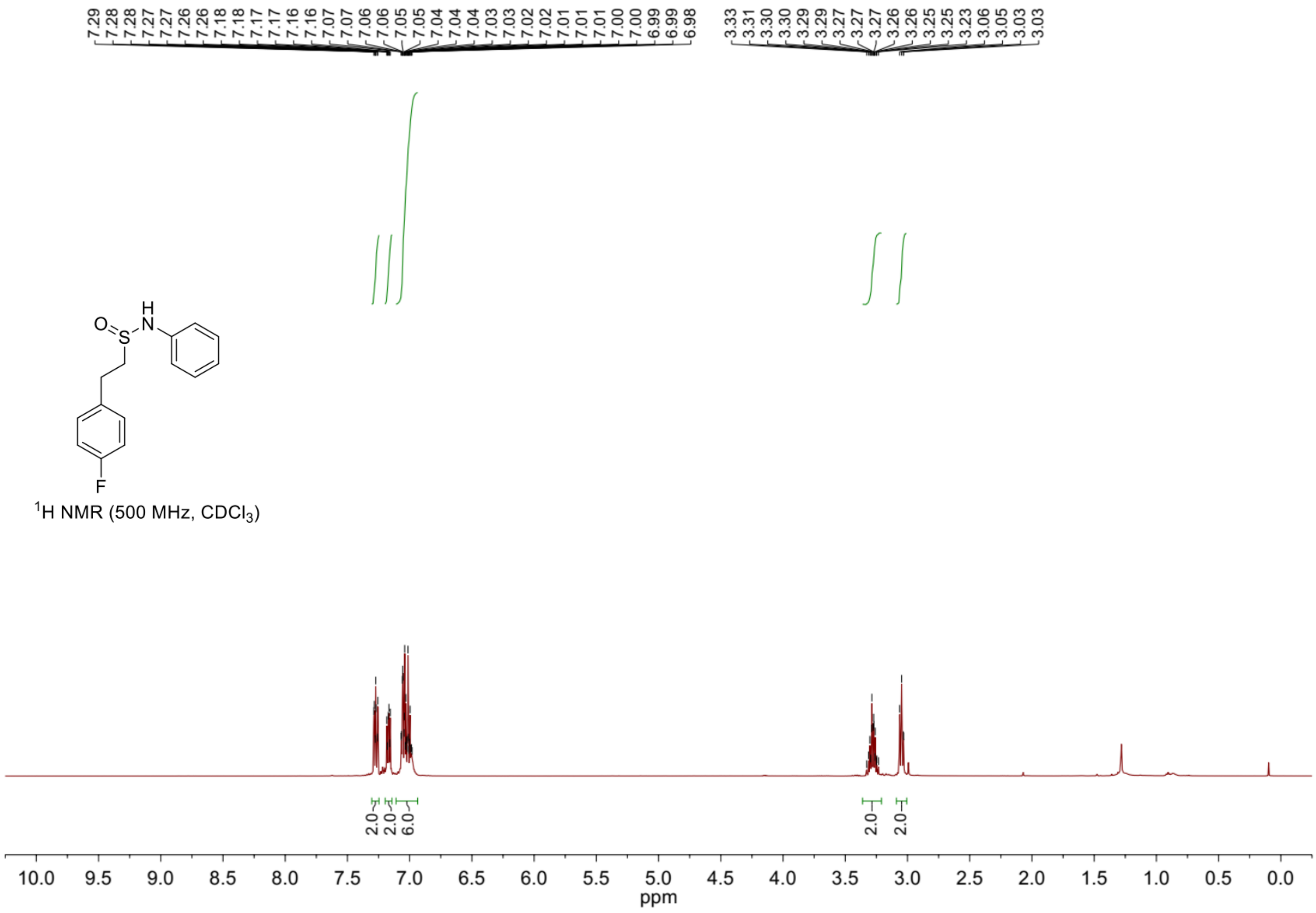

2-(4-Fluorophenyl)-*N*-phenylethane-1-sulfinamide (1f)

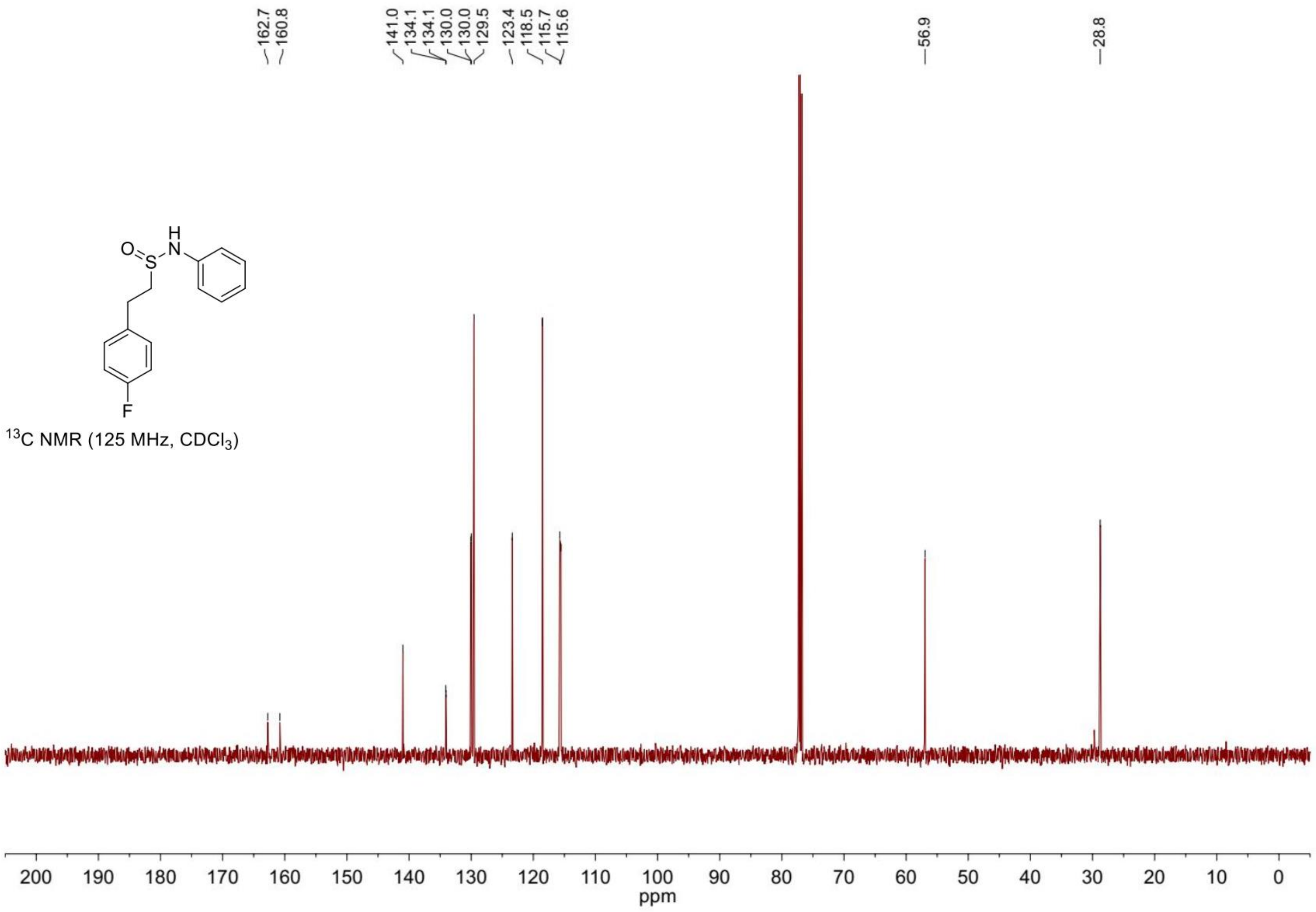

Go to table of contents

4-Oxo-*N*,4-diphenylbutane-1-sulfinamide (1g)

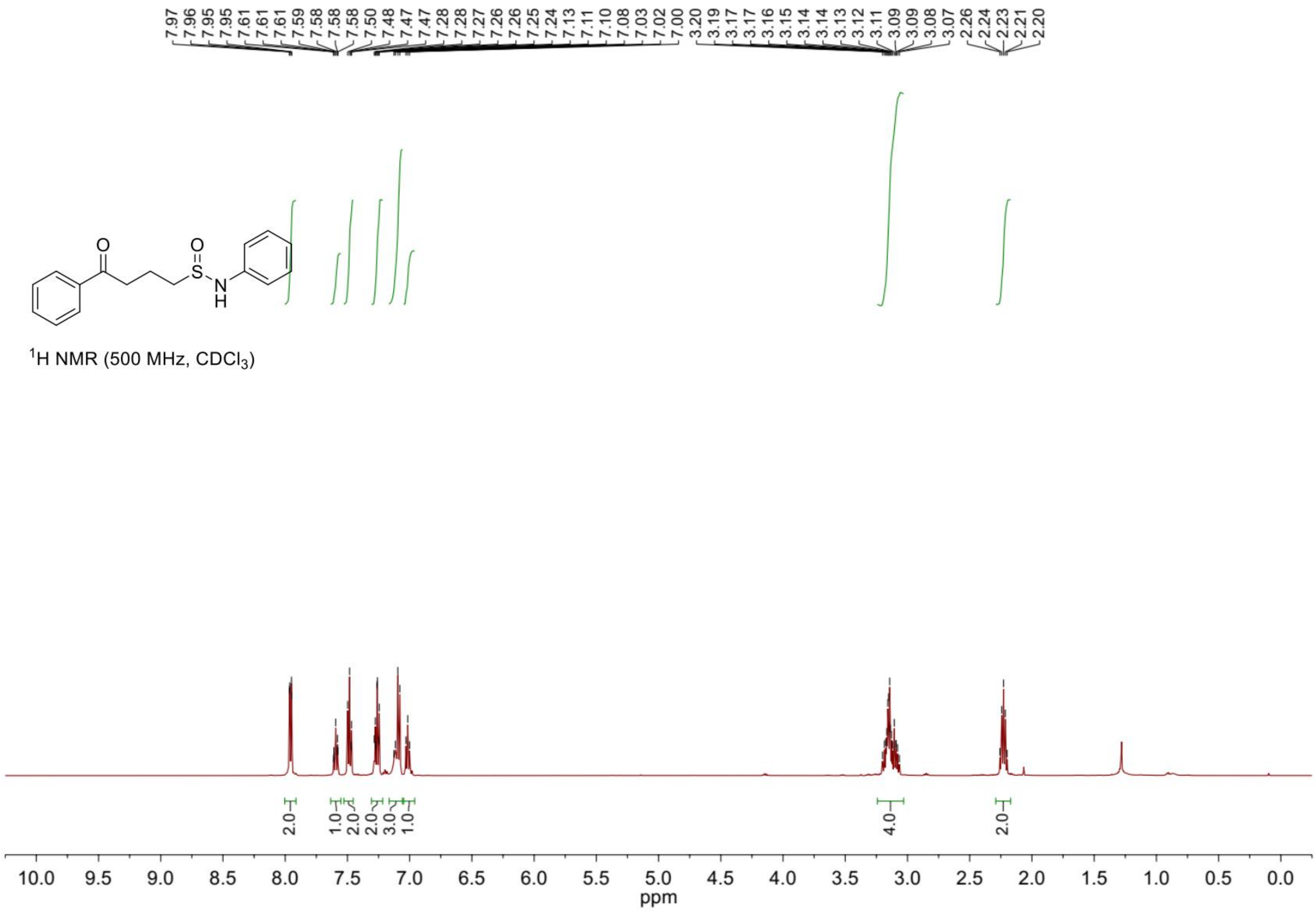

Go to table of contents

4-Oxo-*N*,4-diphenylbutane-1-sulfinamide (1g)

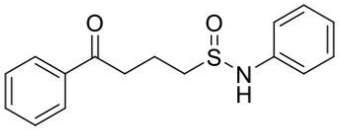

^{13}C NMR (125 MHz, $CDCl_3$)

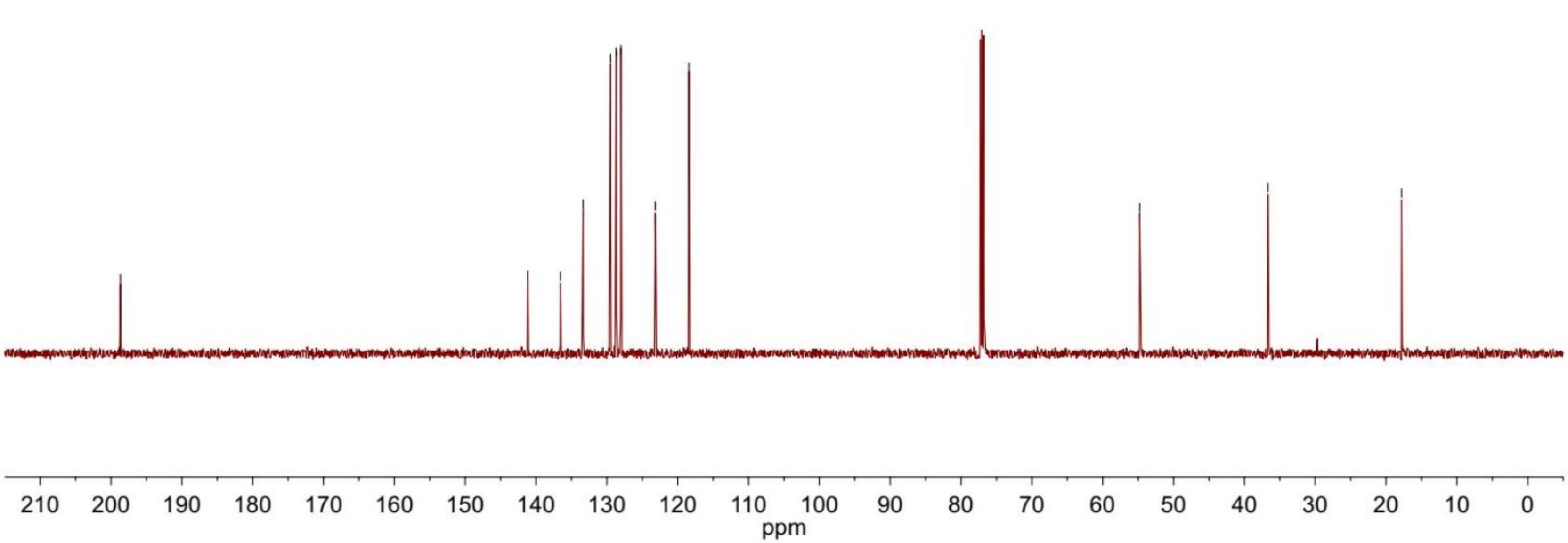

Go to table of contents

Methyl 5-((phenylamino)sulfinyl)pentanoate (1h)

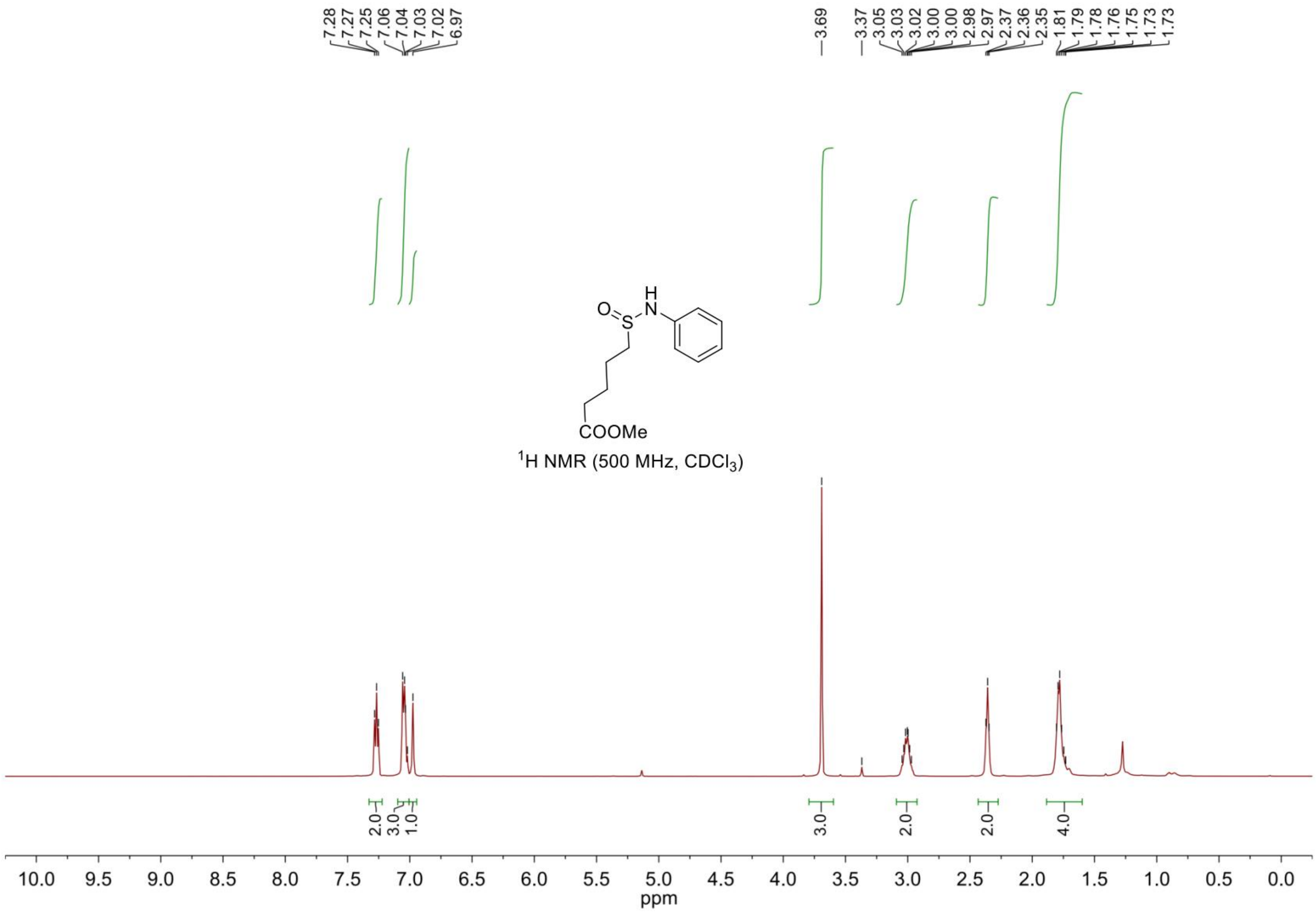

Go to table of contents

Methyl 5-((phenylamino)sulfinyl)pentanoate (1h)

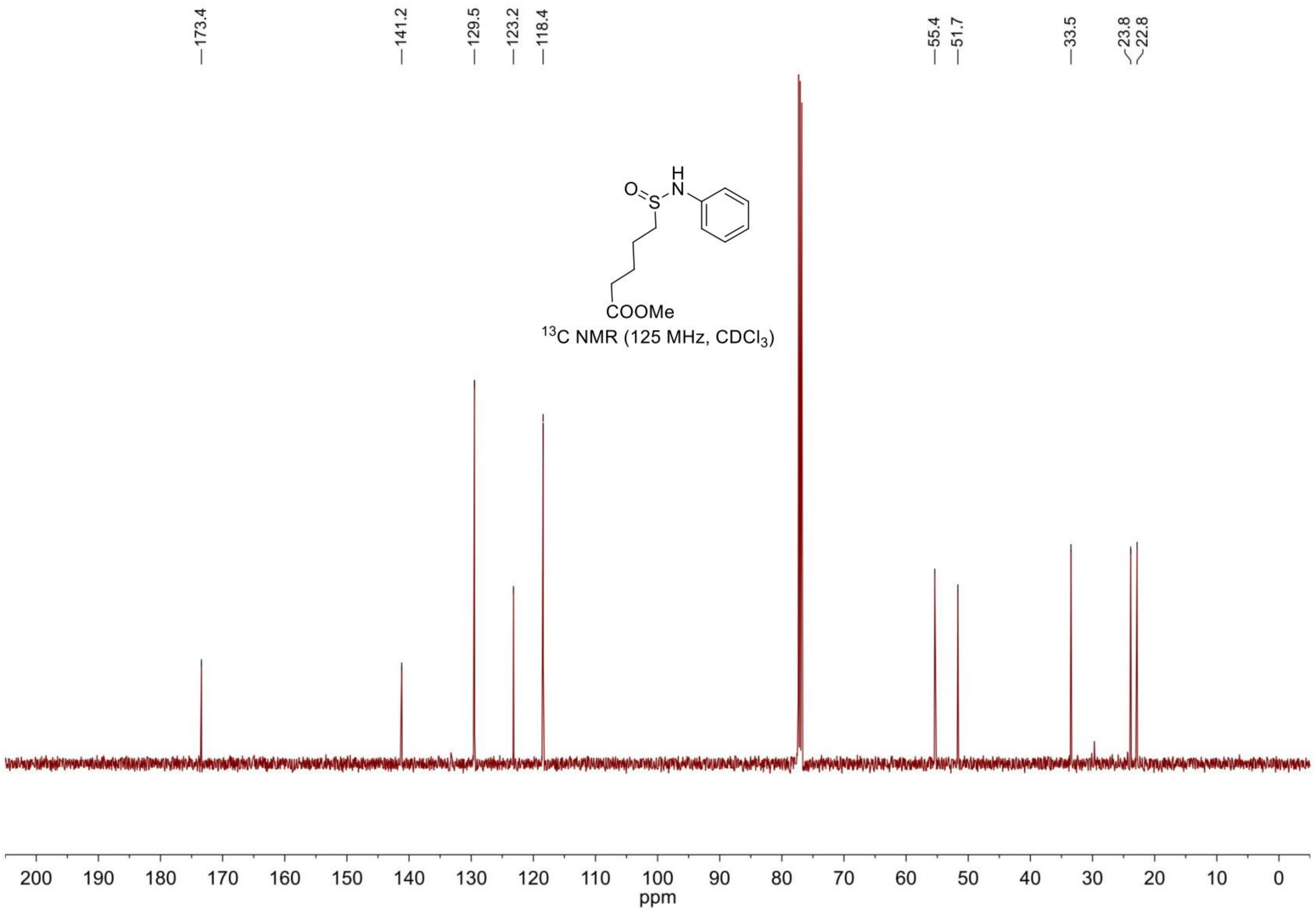

Go to table of contents

N-Phenyl-3-(thiophen-2-yl)propane-1-sulfinamide (1i)

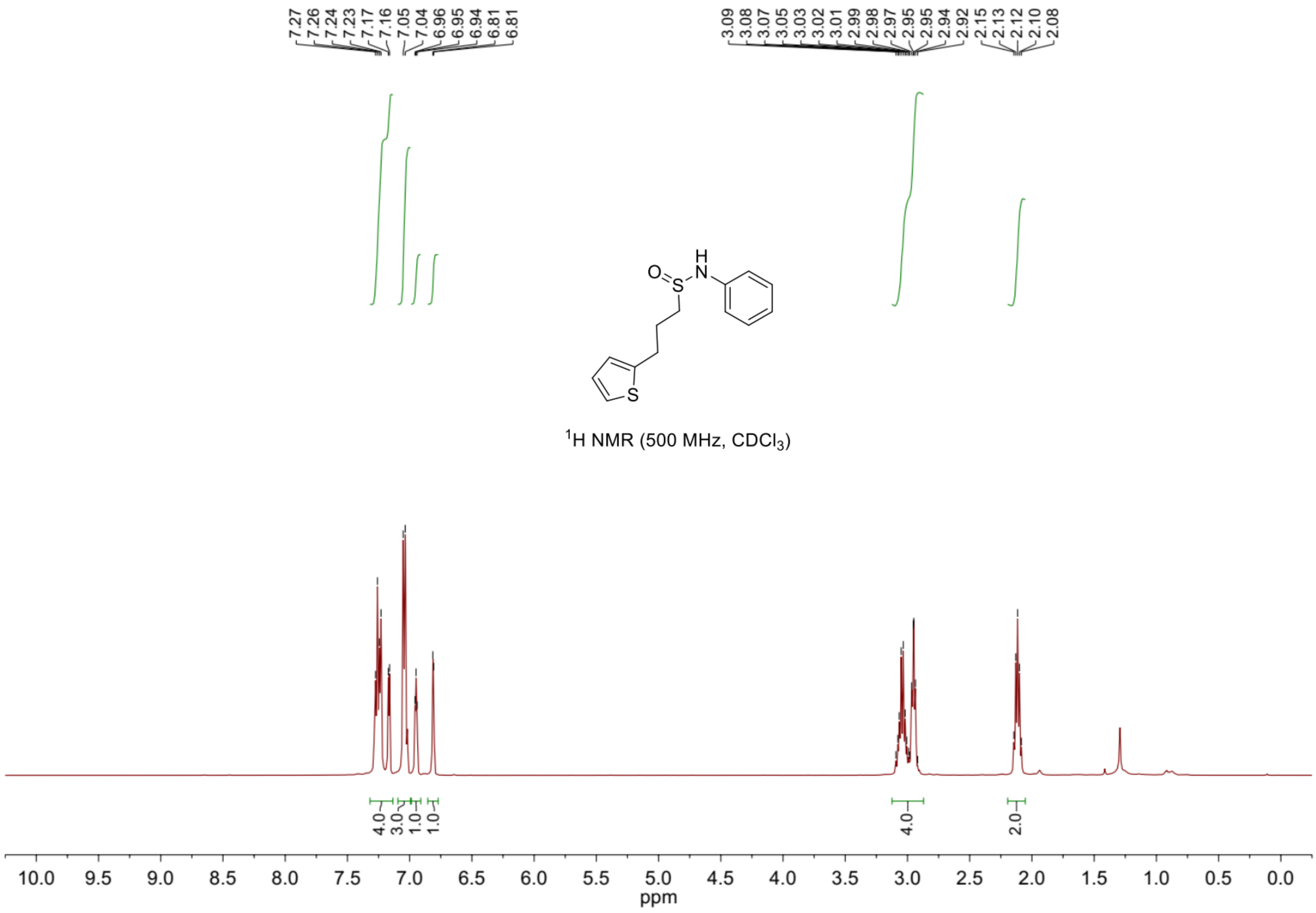

Go to table of contents

_N_-Phenyl-3-(thiophen-2-yl)propane-1-sulfinamide (1i)

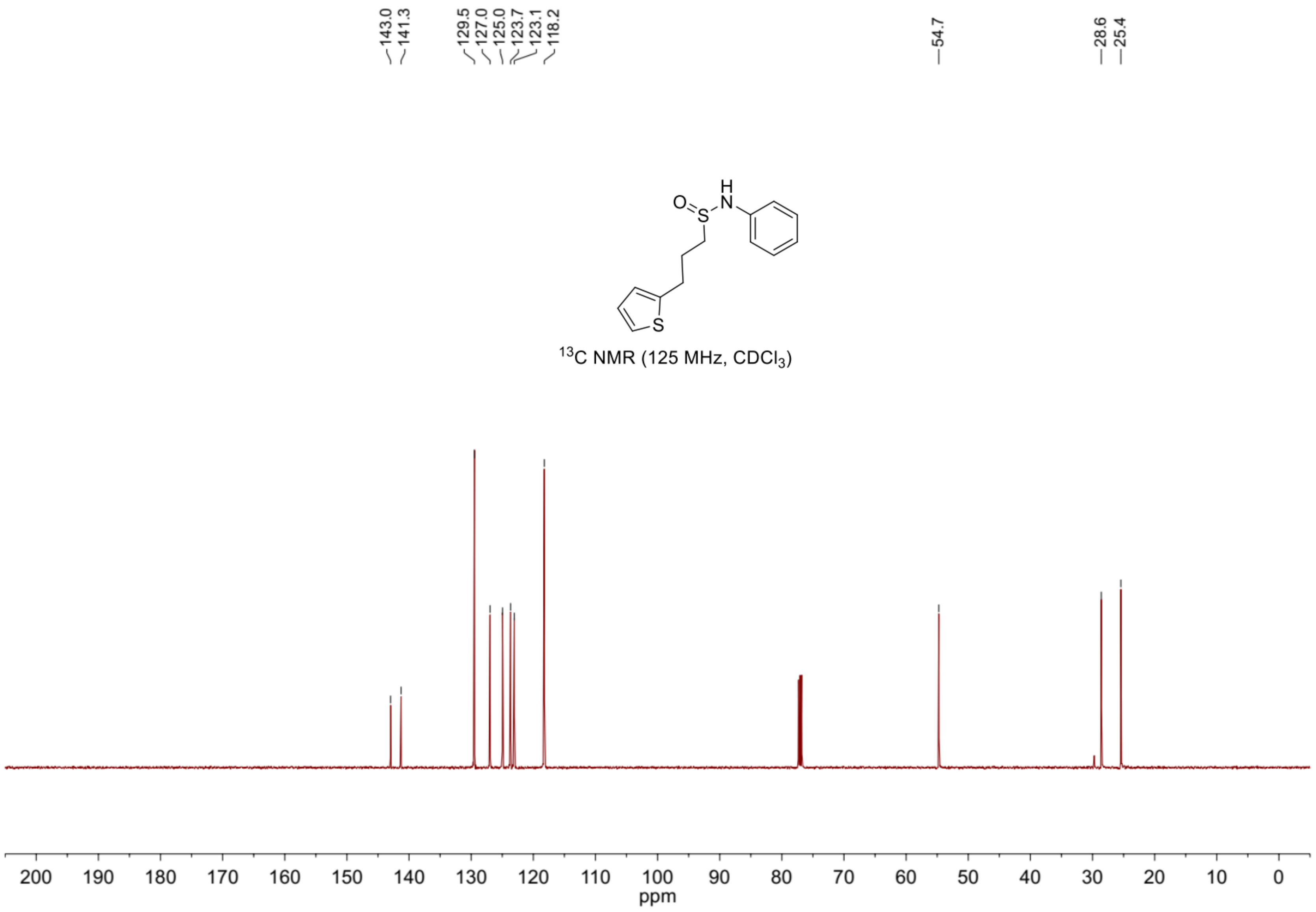

Go to table of contents

4,4-Difluoro-*N*-phenylcyclohexane-1-sulfinamide (1j)

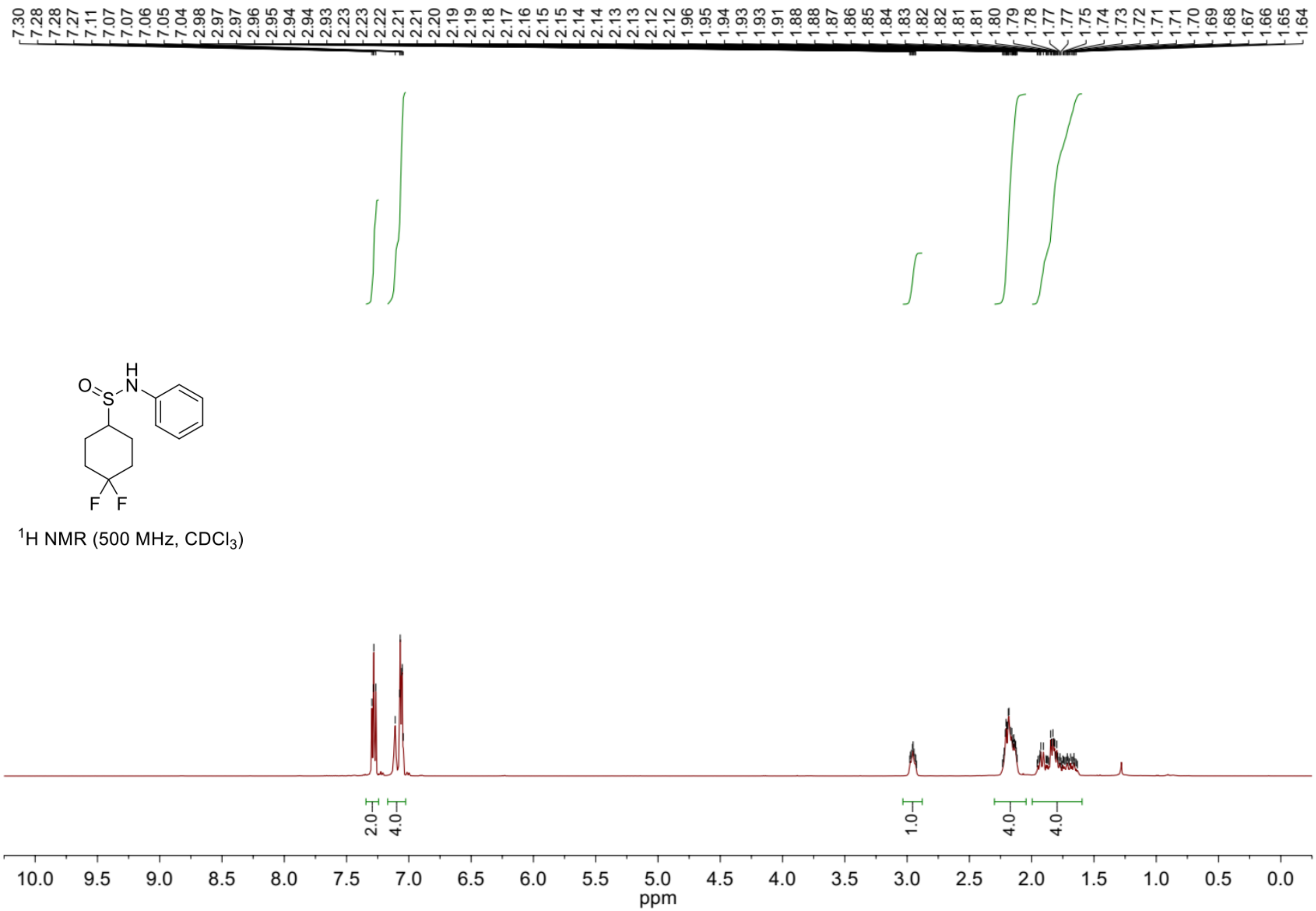

Go to table of contents

4,4-Difluoro-*N*-phenylcyclohexane-1-sulfinamide (1j)

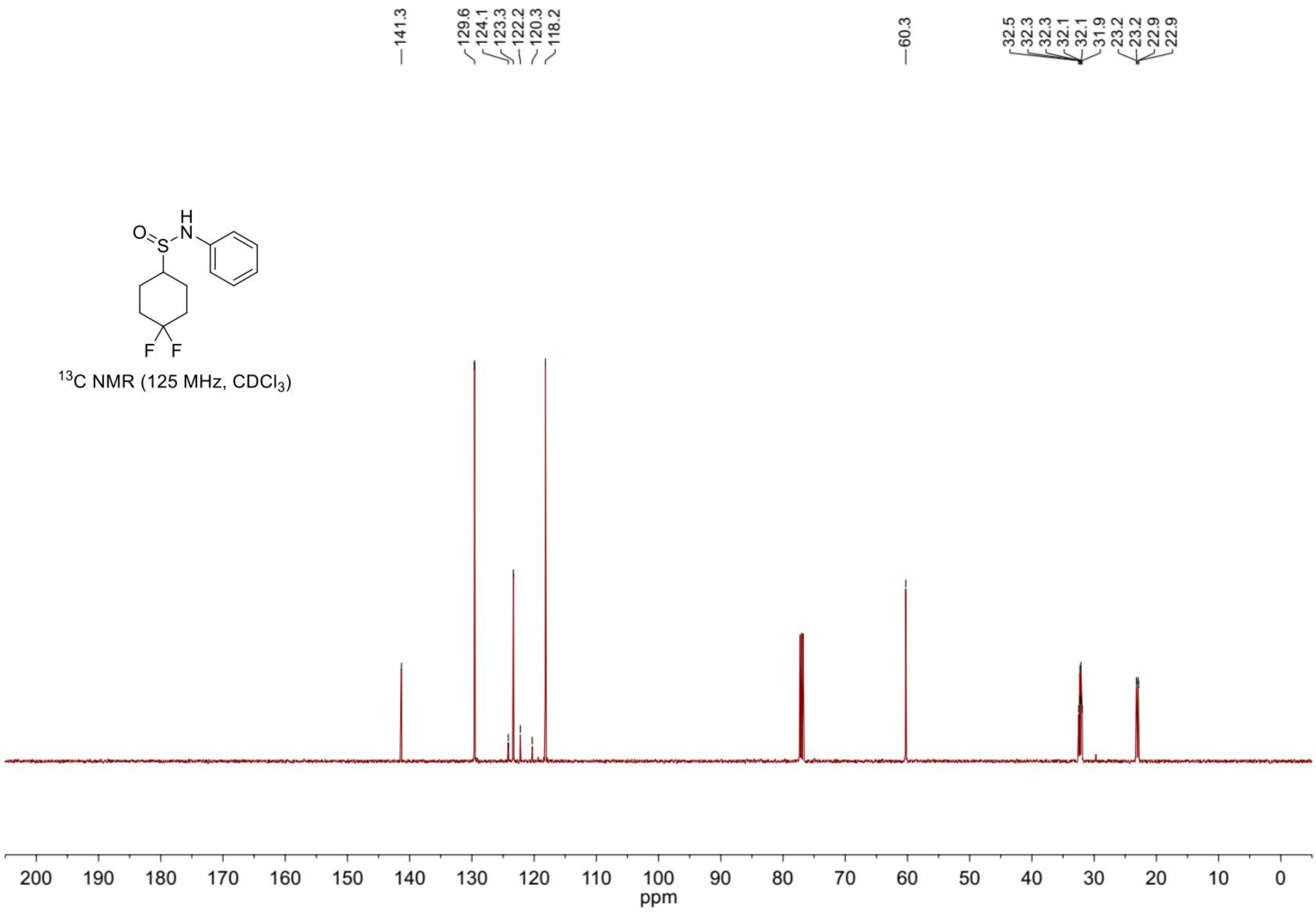

Go to table of contents

N-Phenylcycloheptanesulfinamide (1k)

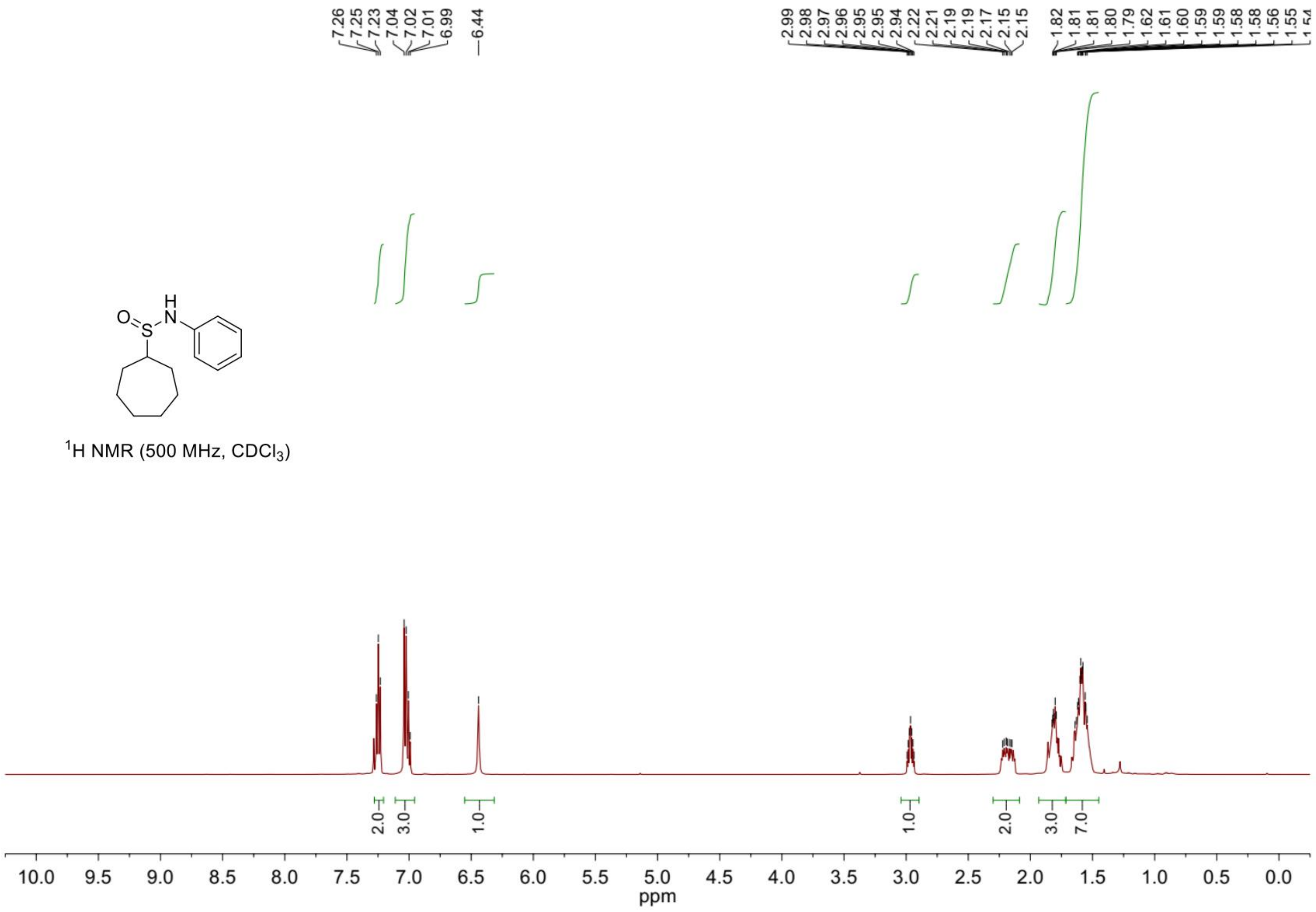

N-Phenylcycloheptanesulfinamide (1k)

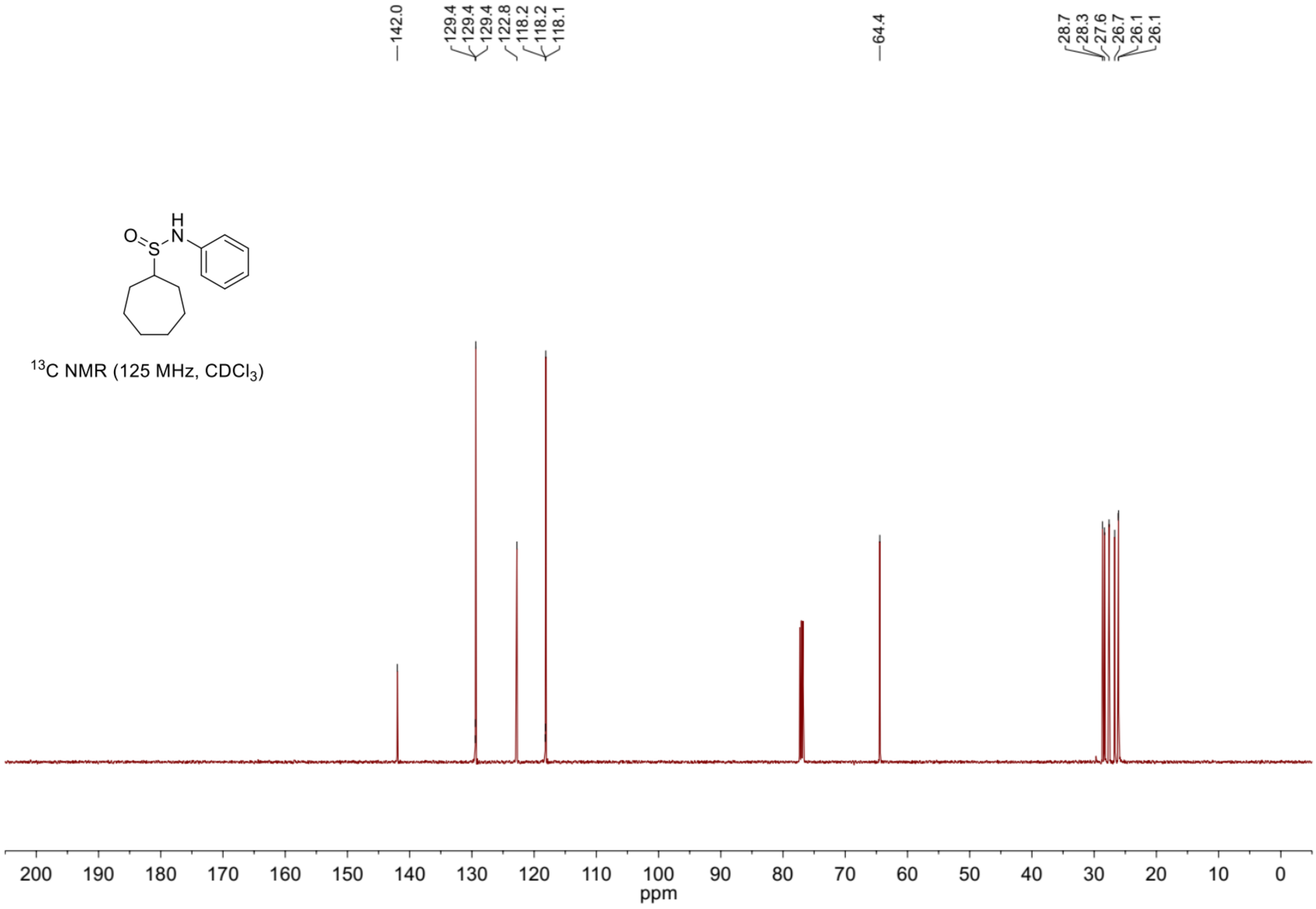

Go to table of contents

N-Phenylcyclopent-3-ene-1-sulfinamide (1l)

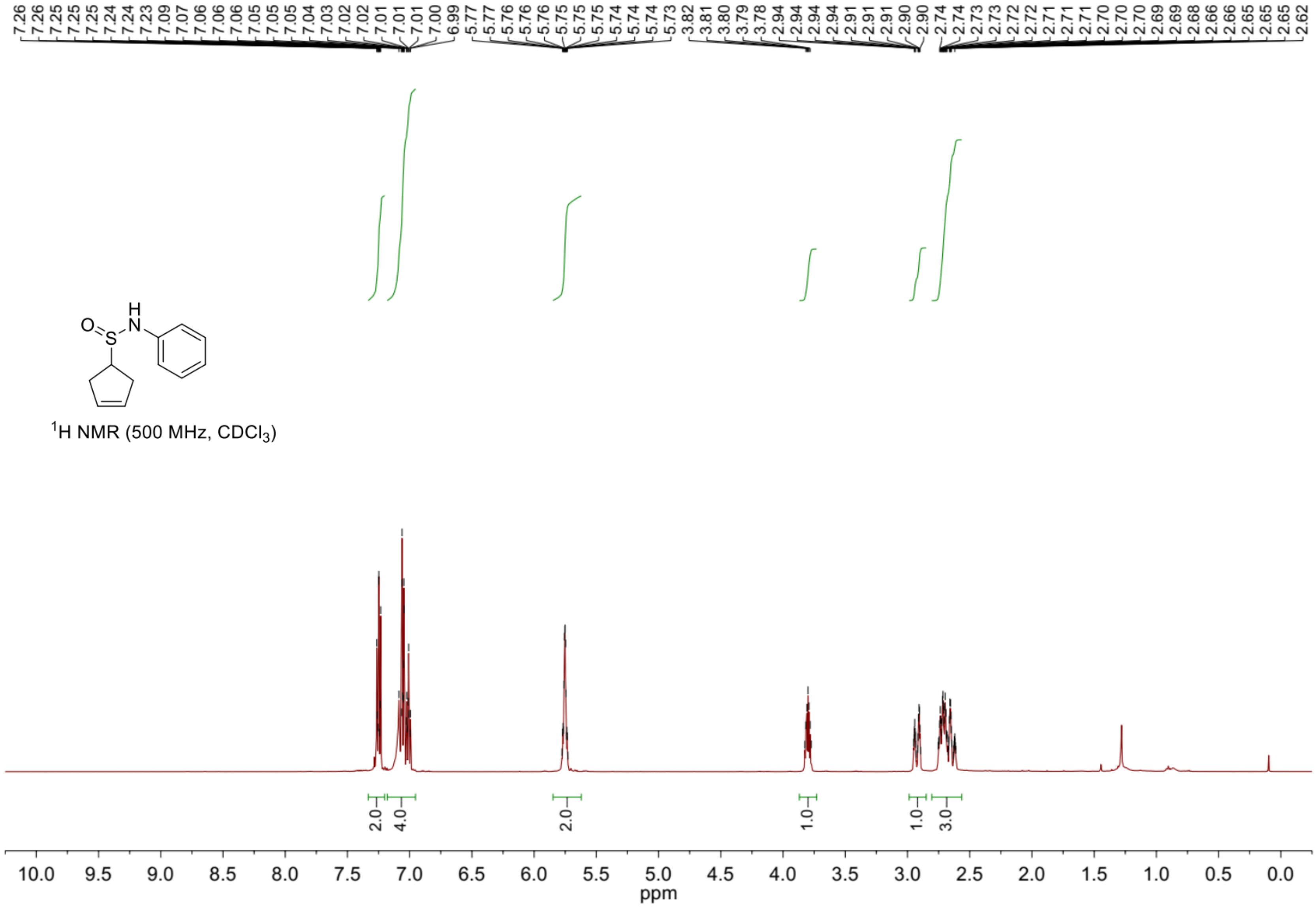

Go to table of contents

N-Phenylcyclopent-3-ene-1-sulfinamide (1l)

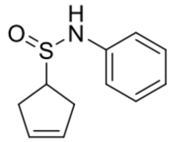

^{13}C NMR (125 MHz, $CDCl_3$)

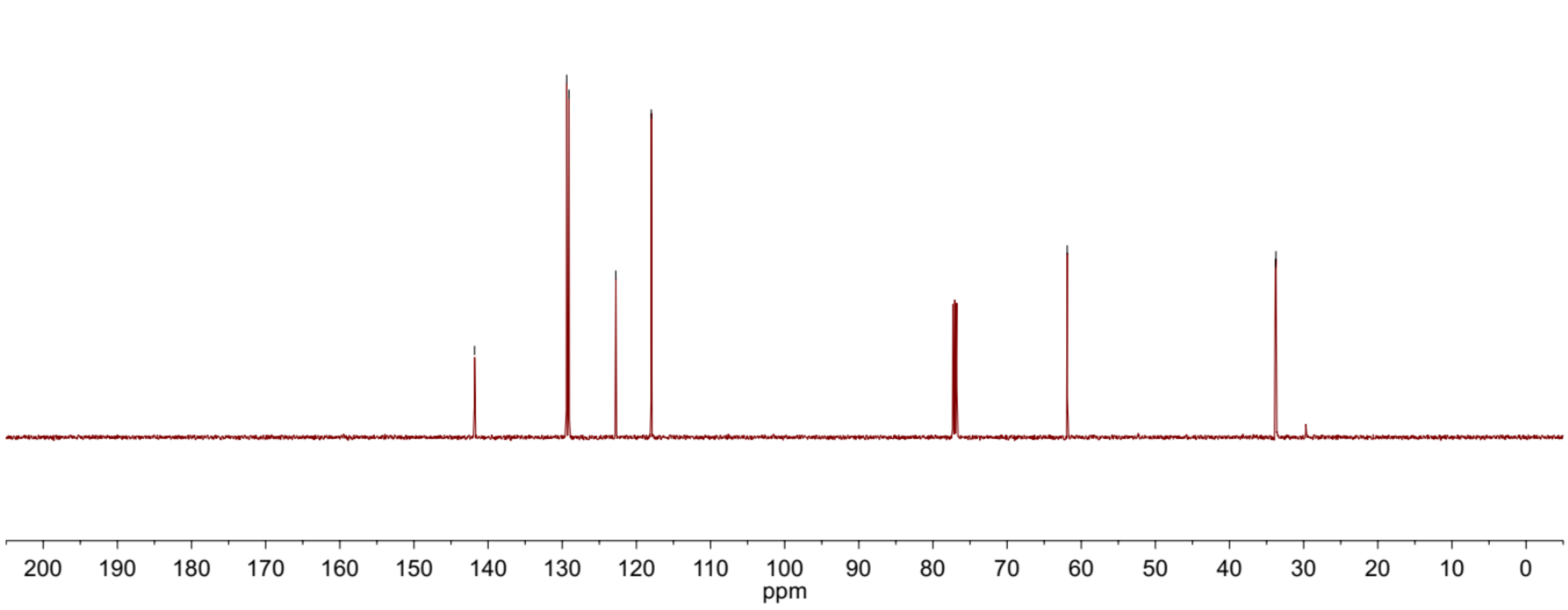

Go to table of contents

N-Phenyl-2,3-dihydro-1H-indene-2-sulfinamide (1m)

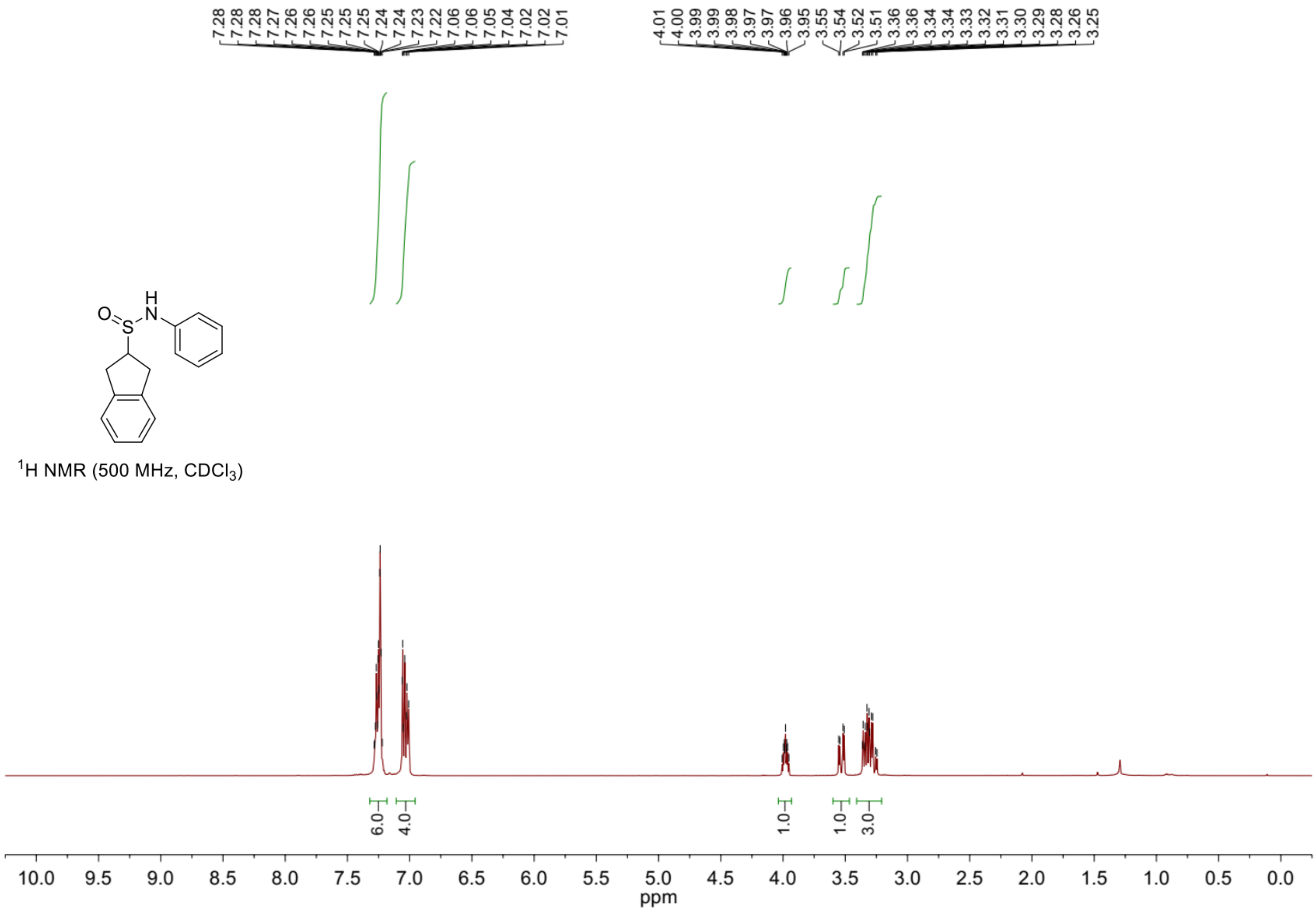

Go to table of contents

N-Phenyl-2,3-dihydro-1H-indene-2-sulfinamide (1m)

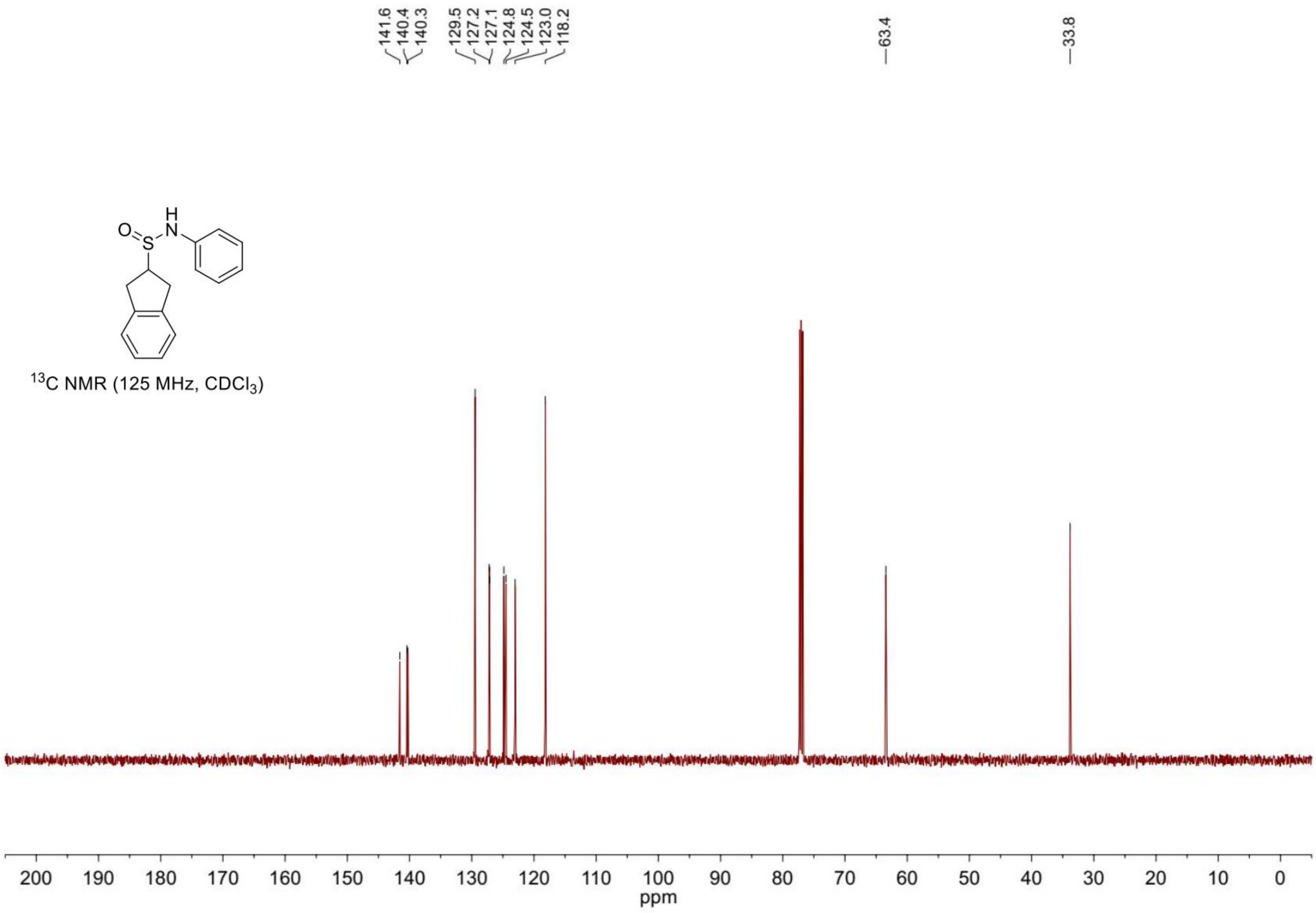

^{13}C NMR (125 MHz, $CDCl_3$)

Go to table of contents

_N_-Phenyltetrahydro-2_H_-pyran-4-sulfinamide (1n)

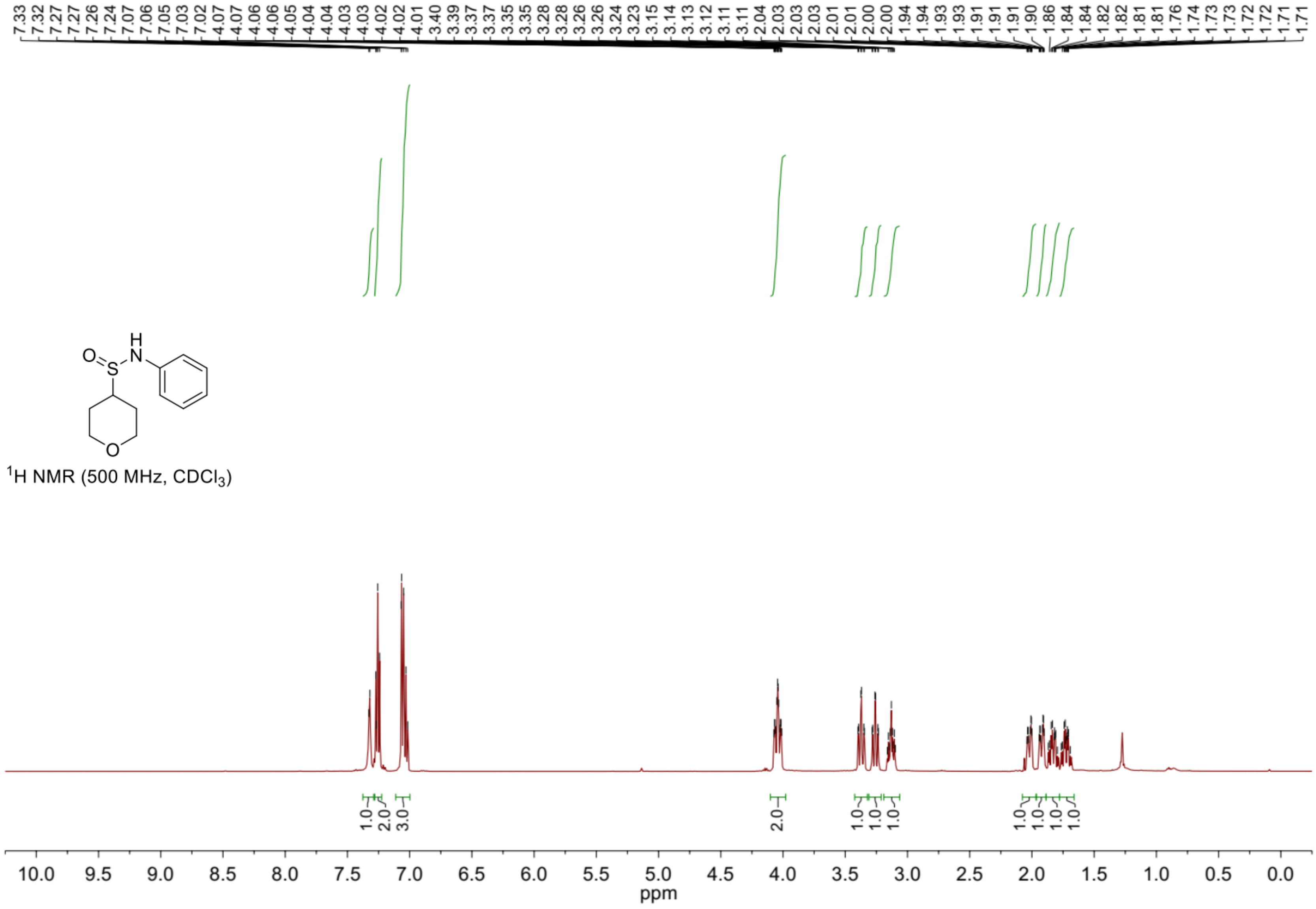

Go to table of contents

N-Phenyltetrahydro-2H-pyran-4-sulfinamide (1n)

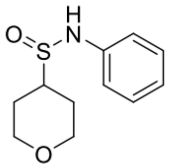

^{13}C NMR (125 MHz, $CDCl_3$)

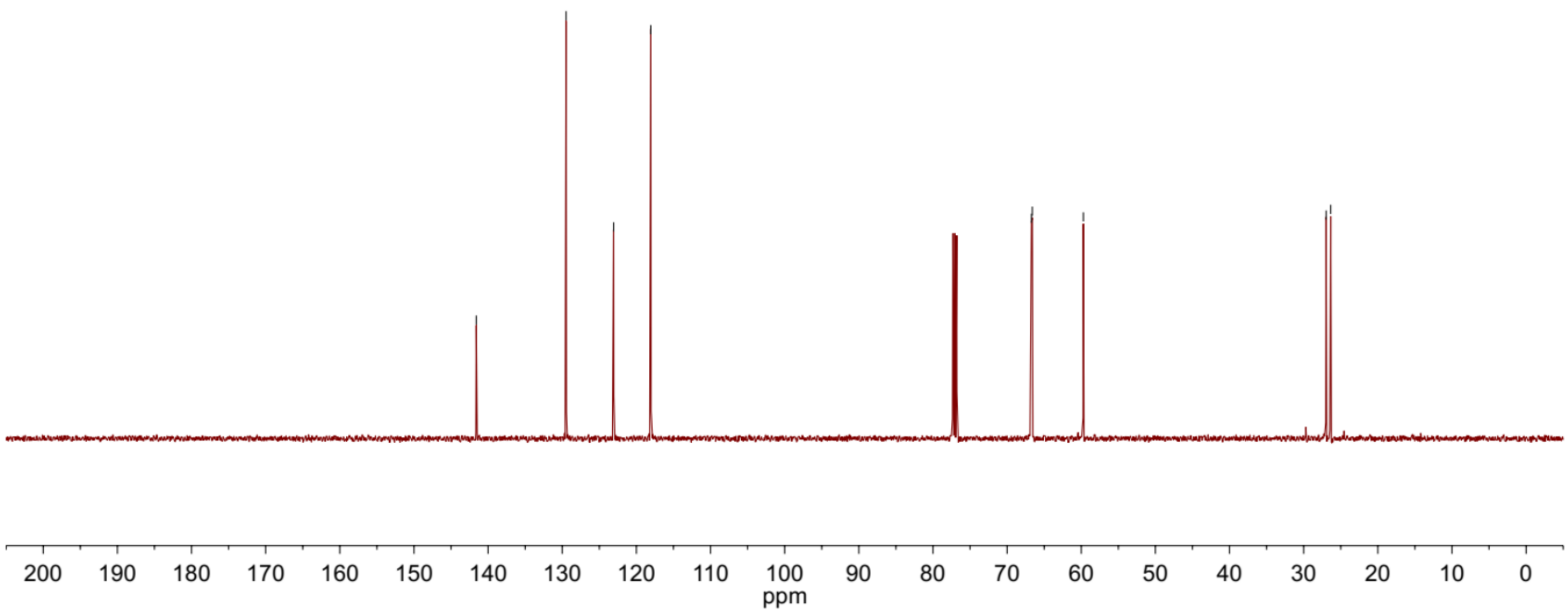

Go to table of contents

Tert-butyl 3-((phenylamino)sulfinyl)piperidine-1-carboxylate (1o)

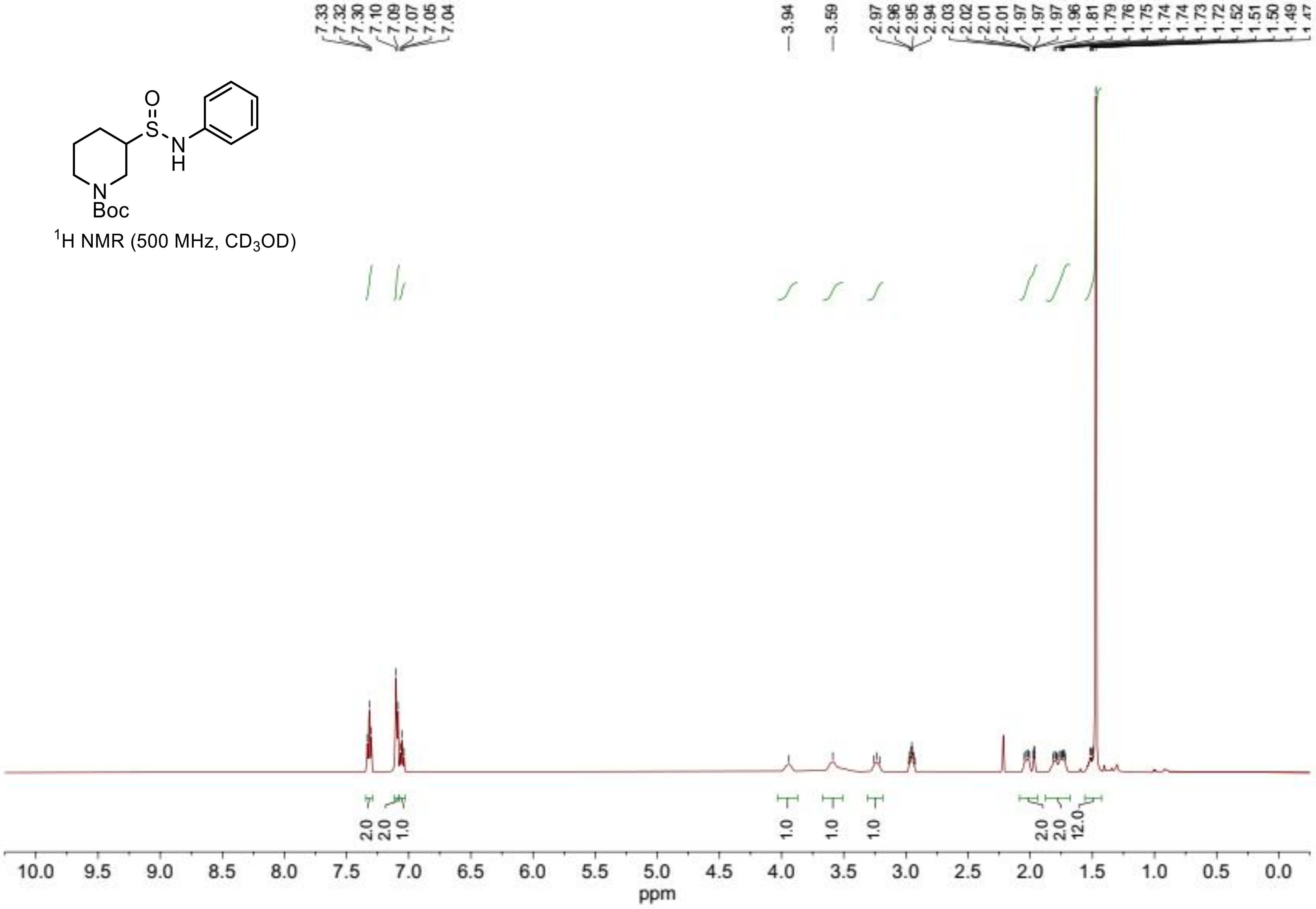

Go to table of contents

Tert-butyl 3-((phenylamino)sulfinyl)piperidine-1-carboxylate (1o)

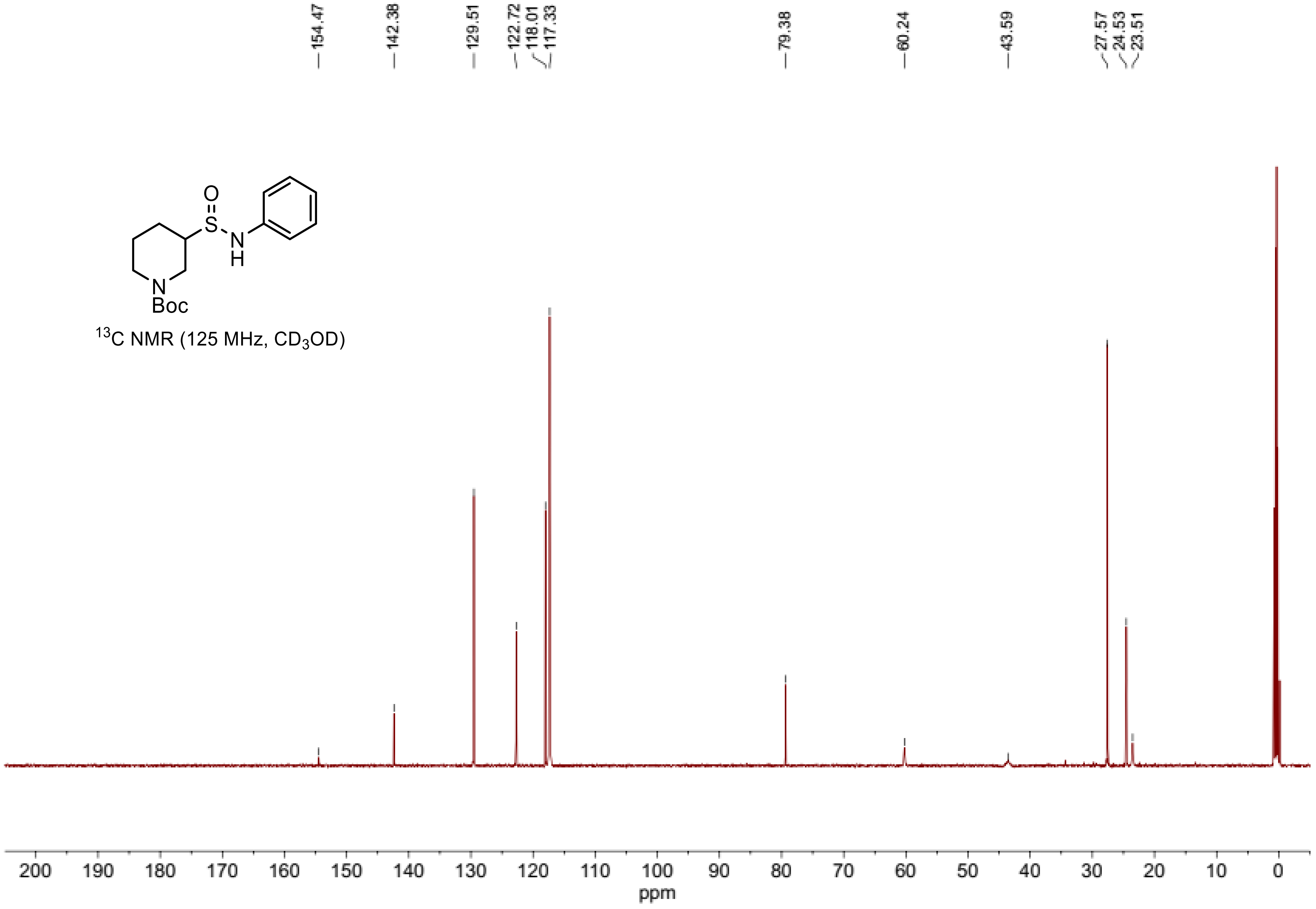

^{13}C NMR (125 MHz, CD_3OD)

Go to table of contents

(3s,5s,7s)-*N*-Phenyladamantane-1-sulfinamide (1p)

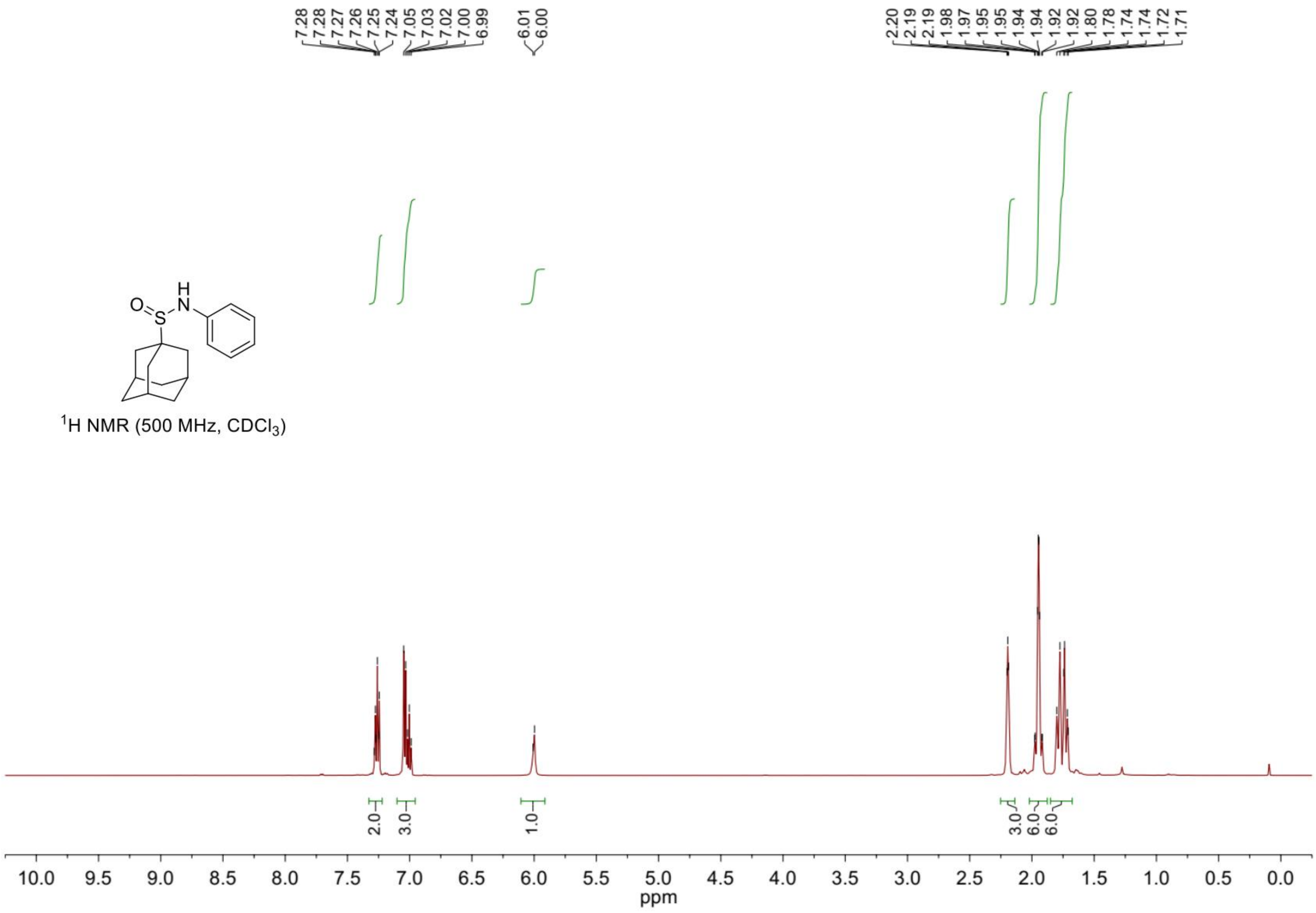

Go to table of contents

(3s,5s,7s)-*N*-Phenyladamantane-1-sulfinamide (1p)

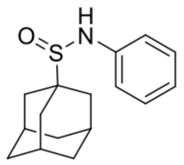

^{13}C NMR (125 MHz, $CDCl_3$)

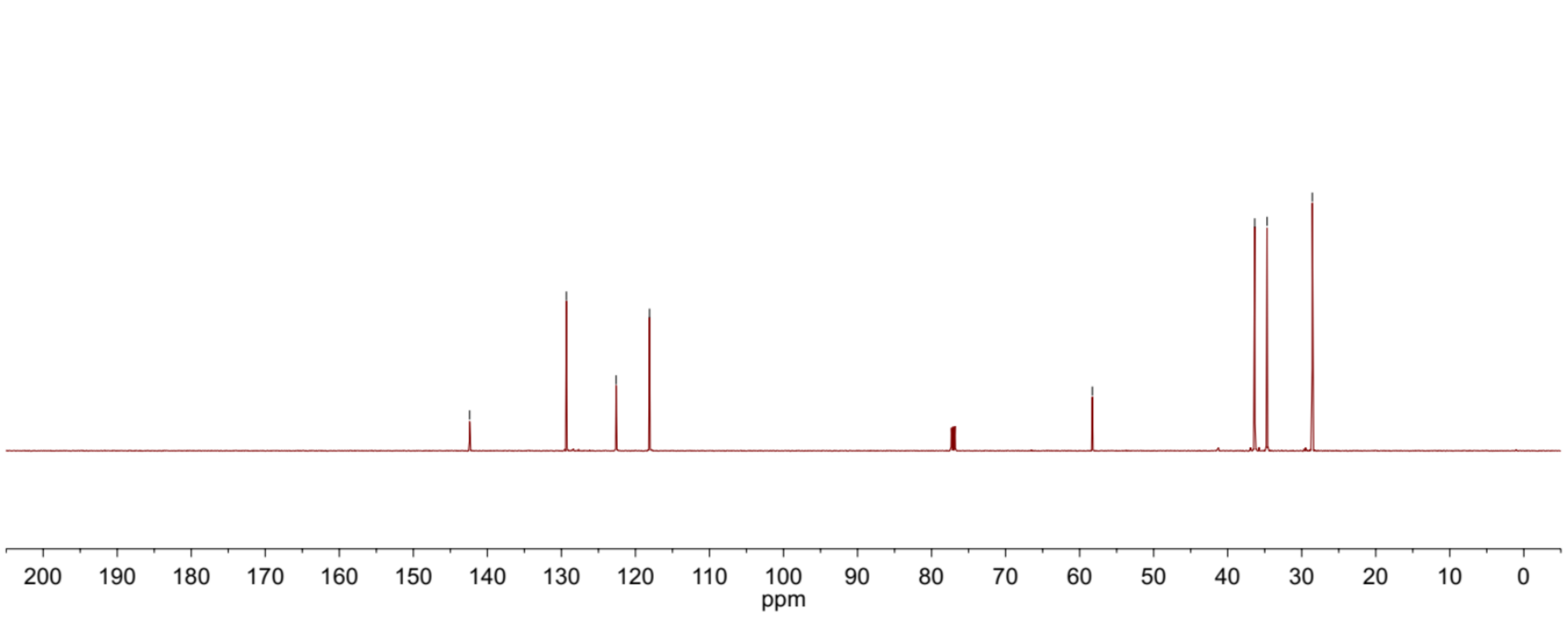

Go to table of contents

3-Methyl-*N*-phenyloxetane-3-sulfinamide (1q)

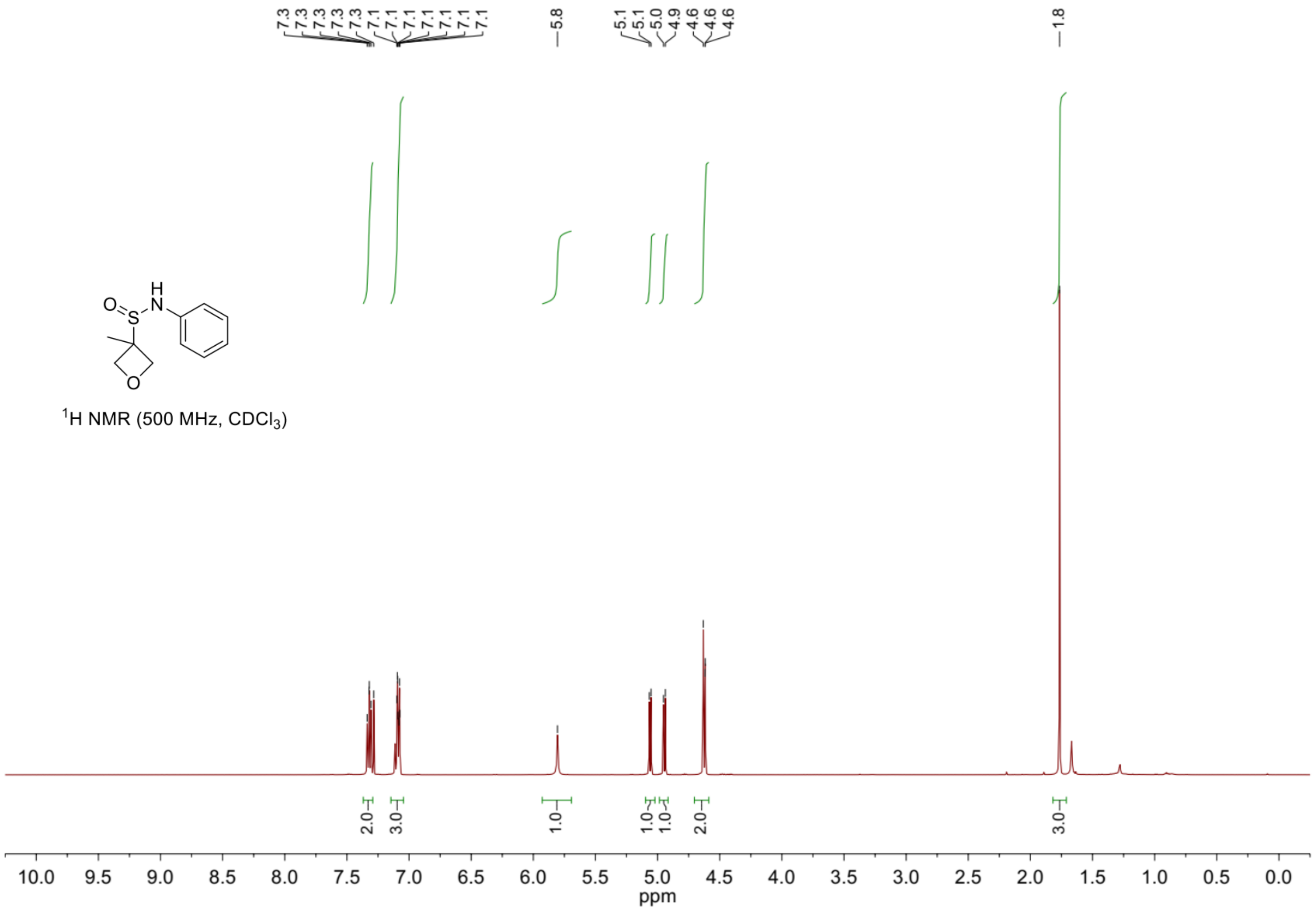

Go to table of contents

3-Methyl-*N*-phenyloxetane-3-sulfinamide (1q)

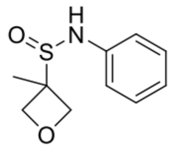

^{13}C NMR (125 MHz, $CDCl_3$)

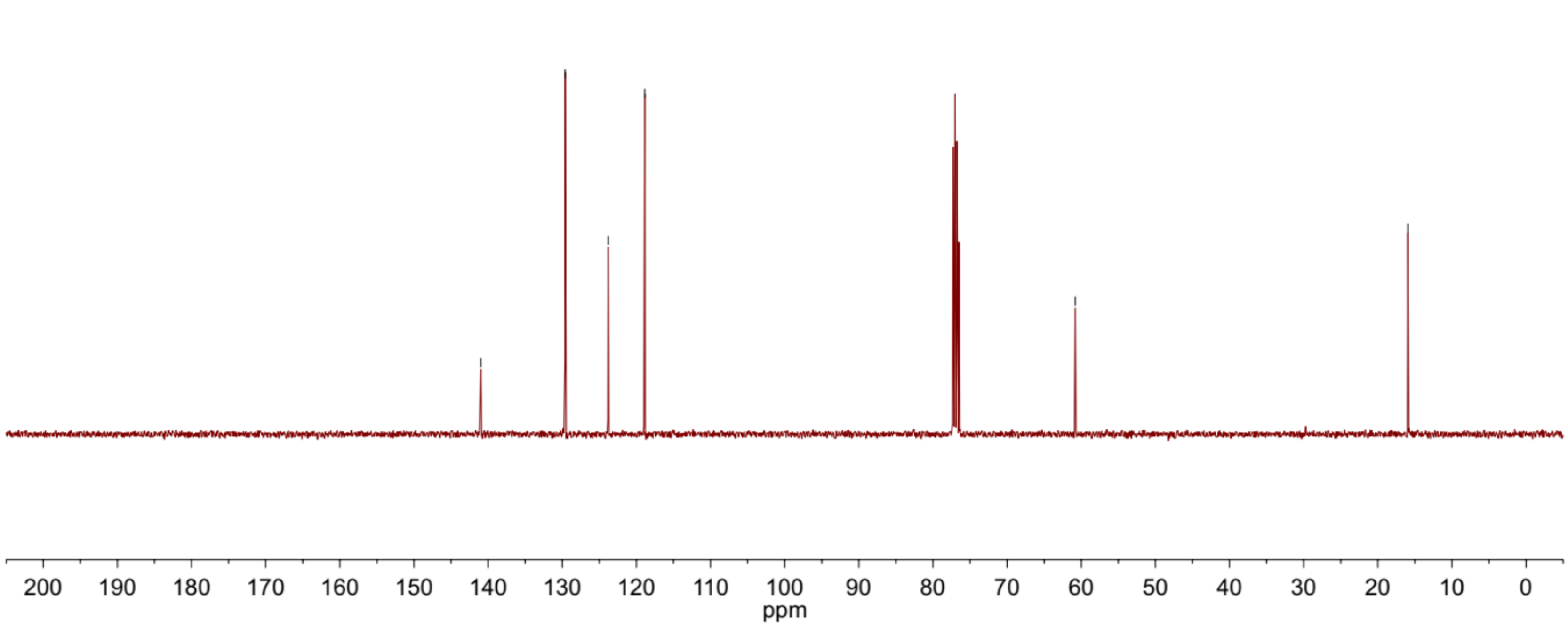

1-Methyl-4-oxo-*N*-phenylcyclohexane-1-sulfinamide (1r)

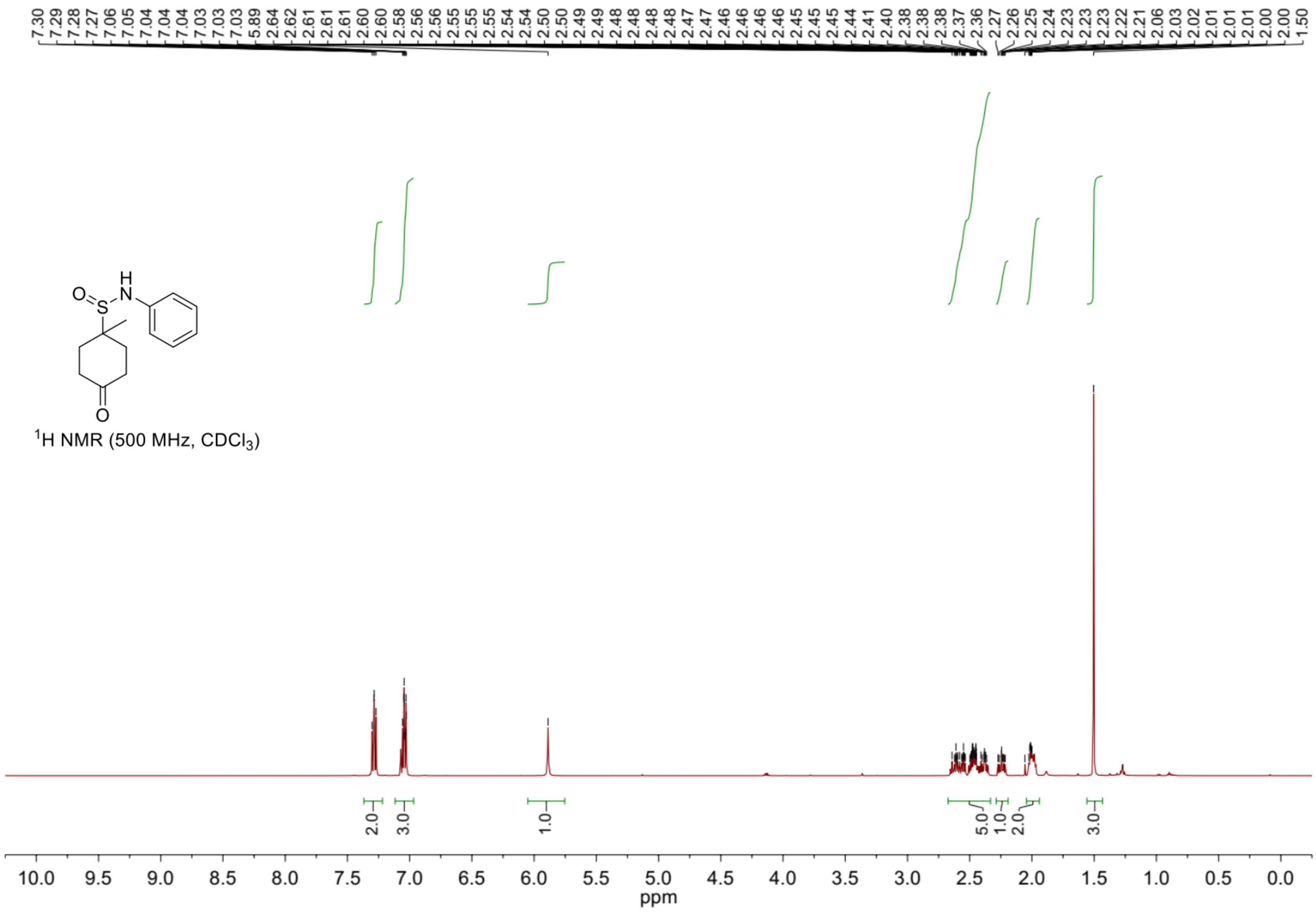

1-Methyl-4-oxo-*N*-phenylcyclohexane-1-sulfinamide (1r)

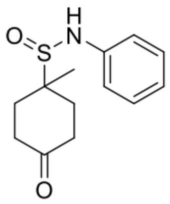

^{13}C NMR (125 MHz, $CDCl_3$)

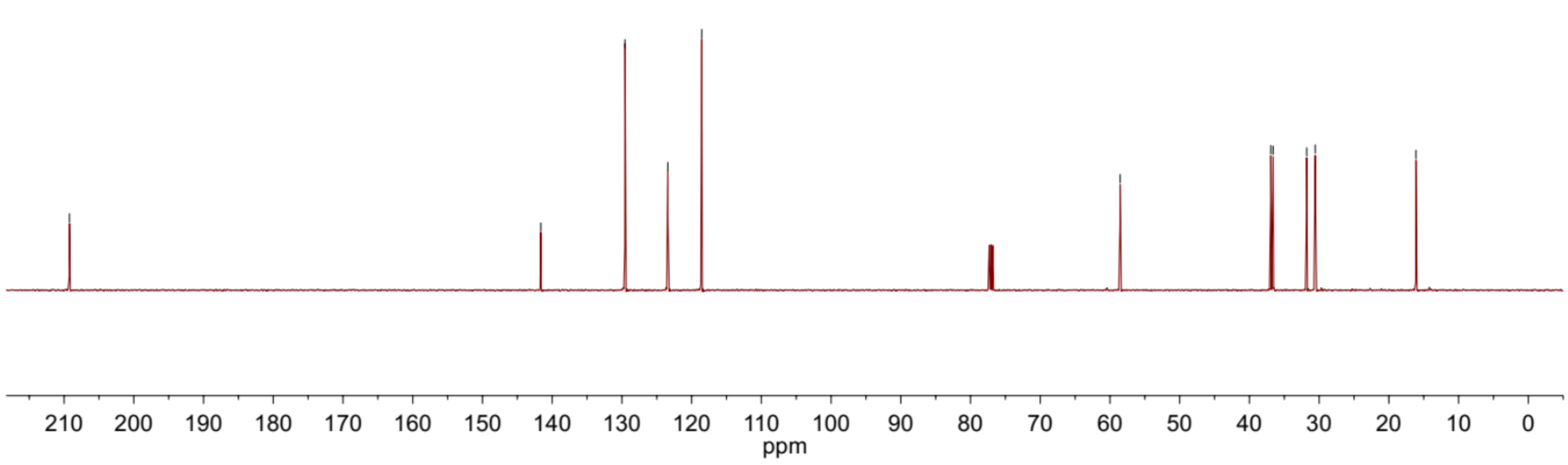

Go to table of contents

(1s,3*R*,5*S*,7s)-4-Oxo-*N*-phenyladamantane-1-sulfinamide (1s)

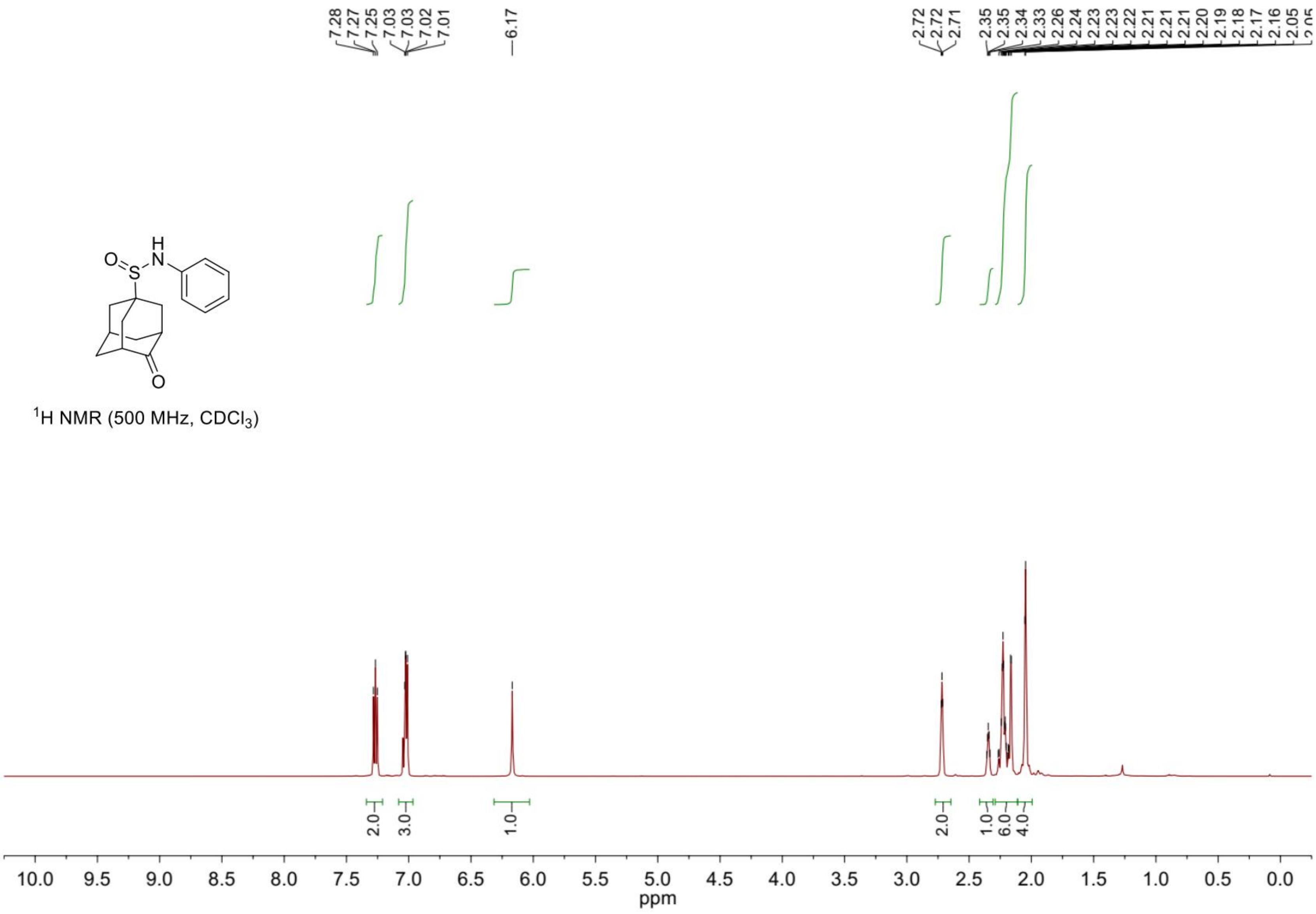

Go to table of contents

(1s,3*R*,5*S*,7s)-4-Oxo-*N*-phenyladamantane-1-sulfinamide (1s)

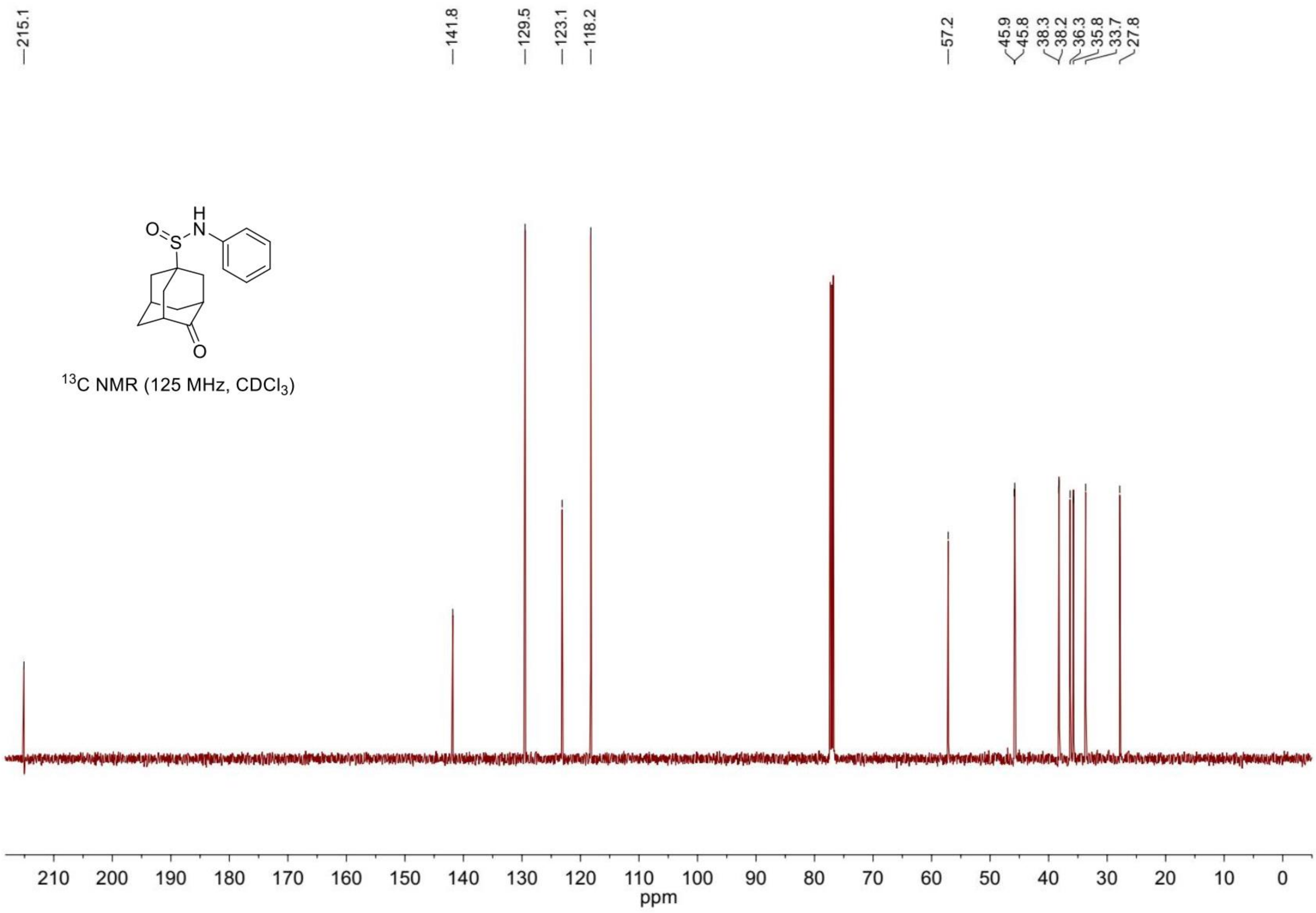

Go to table of contents

Methyl 4-((phenylamino)sulfinyl)bicyclo[2.2.2]octane-1-carboxylate (1t)

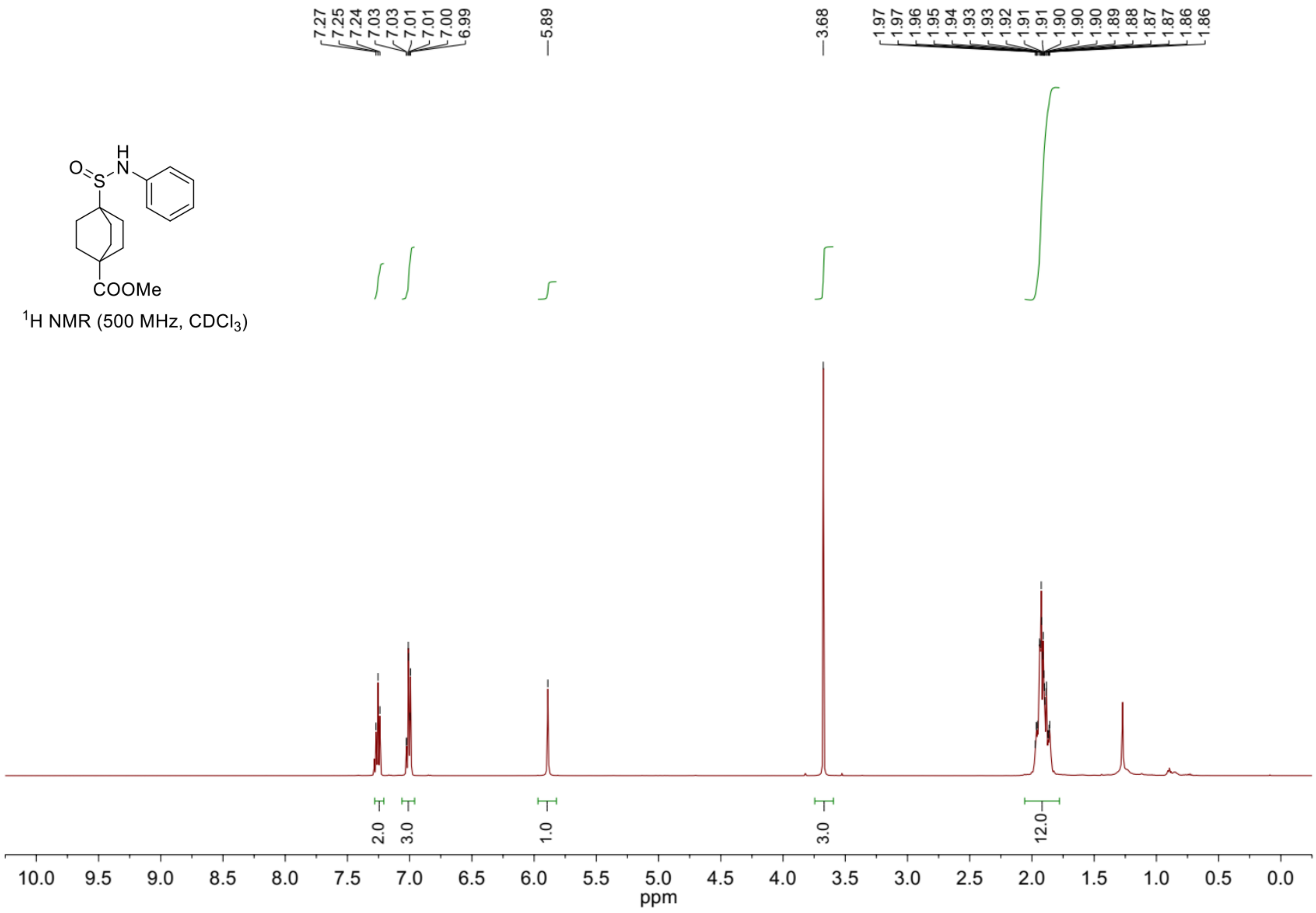

Go to table of contents

Methyl 4-((phenylamino)sulfinyl)bicyclo[2.2.2]octane-1-carboxylate (1t)

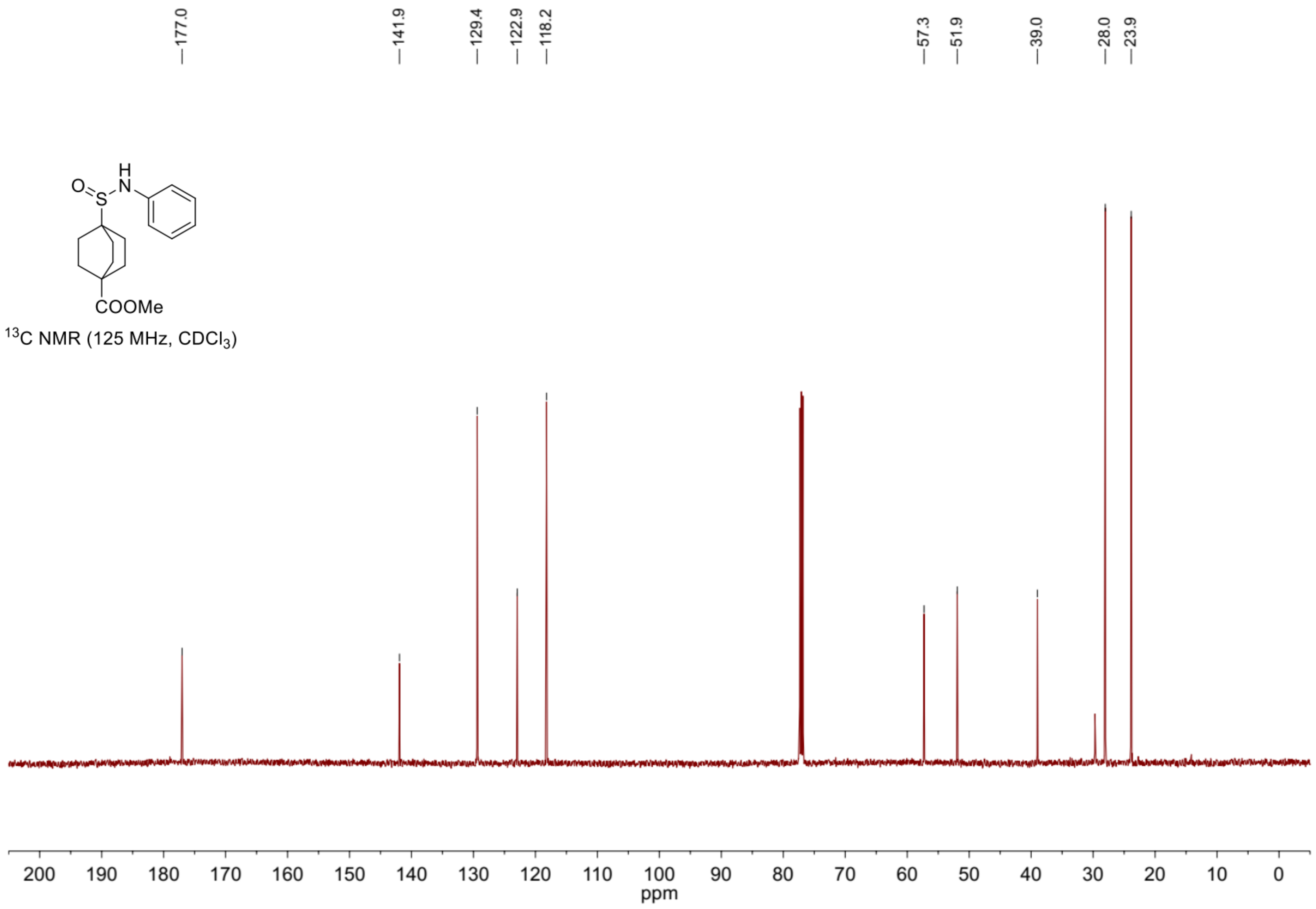

Go to table of contents

tert-Butyl 4-methyl-4-((phenylamino)sulfinyl)piperidine-1-carboxylate (1u)

^{1}H NMR (500 MHz, $CDCl_3$)

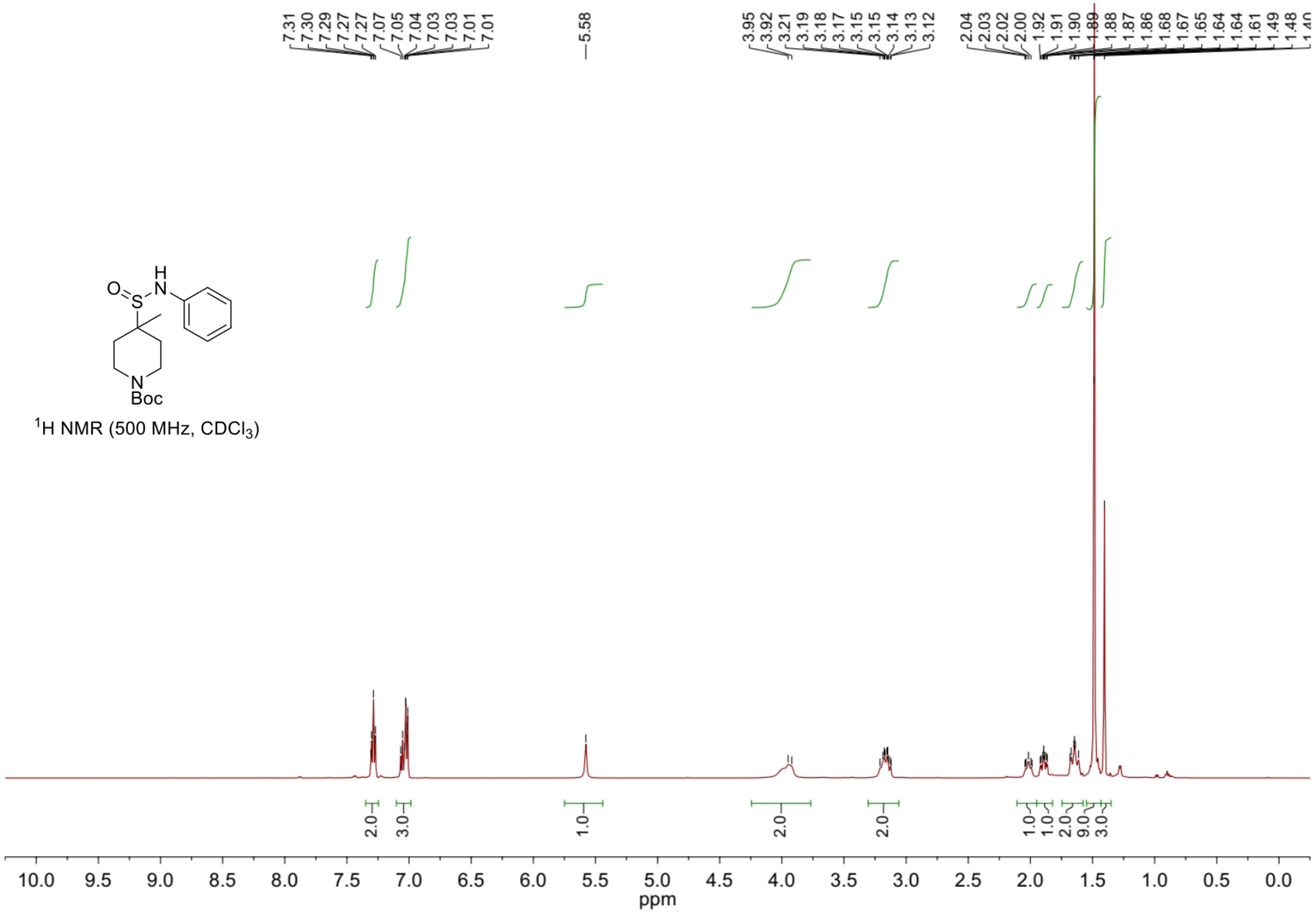

Go to table of contents

tert-Butyl 4-methyl-4-((phenylamino)sulfinyl)piperidine-1-carboxylate (1u)

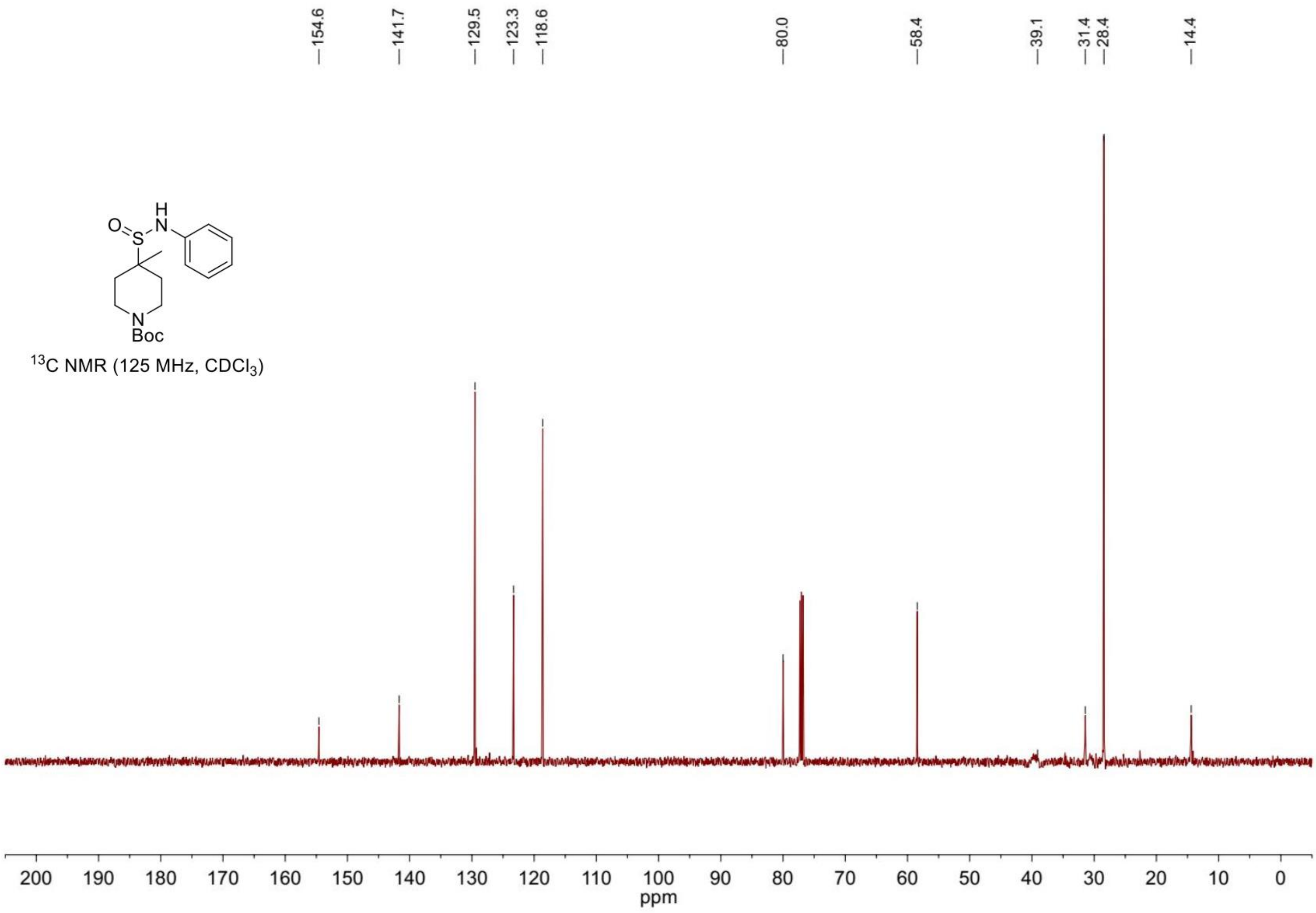

Go to table of contents

N-(4-Cyanophenyl)-4,4-difluorocyclohexane-1-sulfinamide (4a)

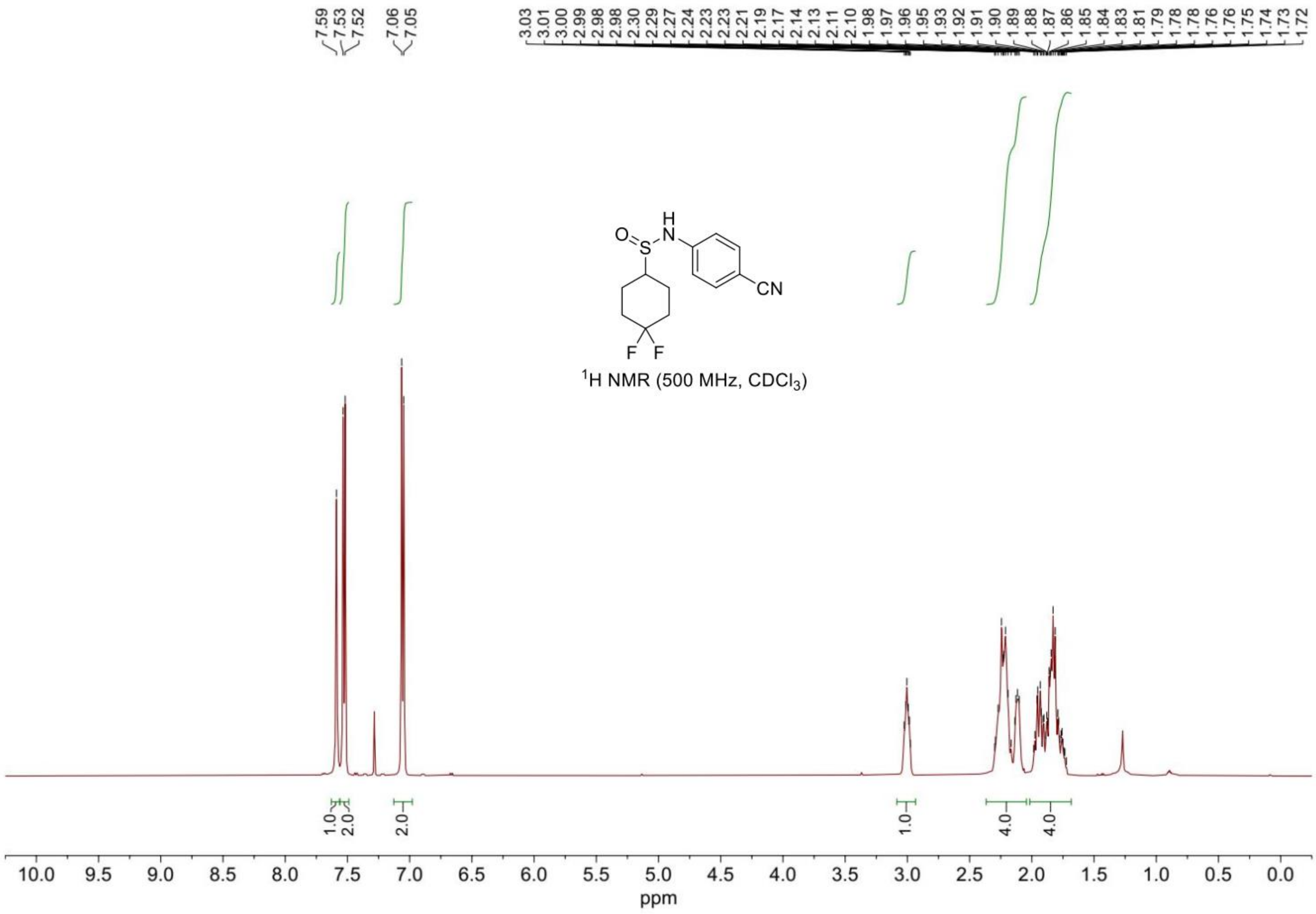

Go to table of contents

N-(4-Cyanophenyl)-4,4-difluorocyclohexane-1-sulfinamide (4a)

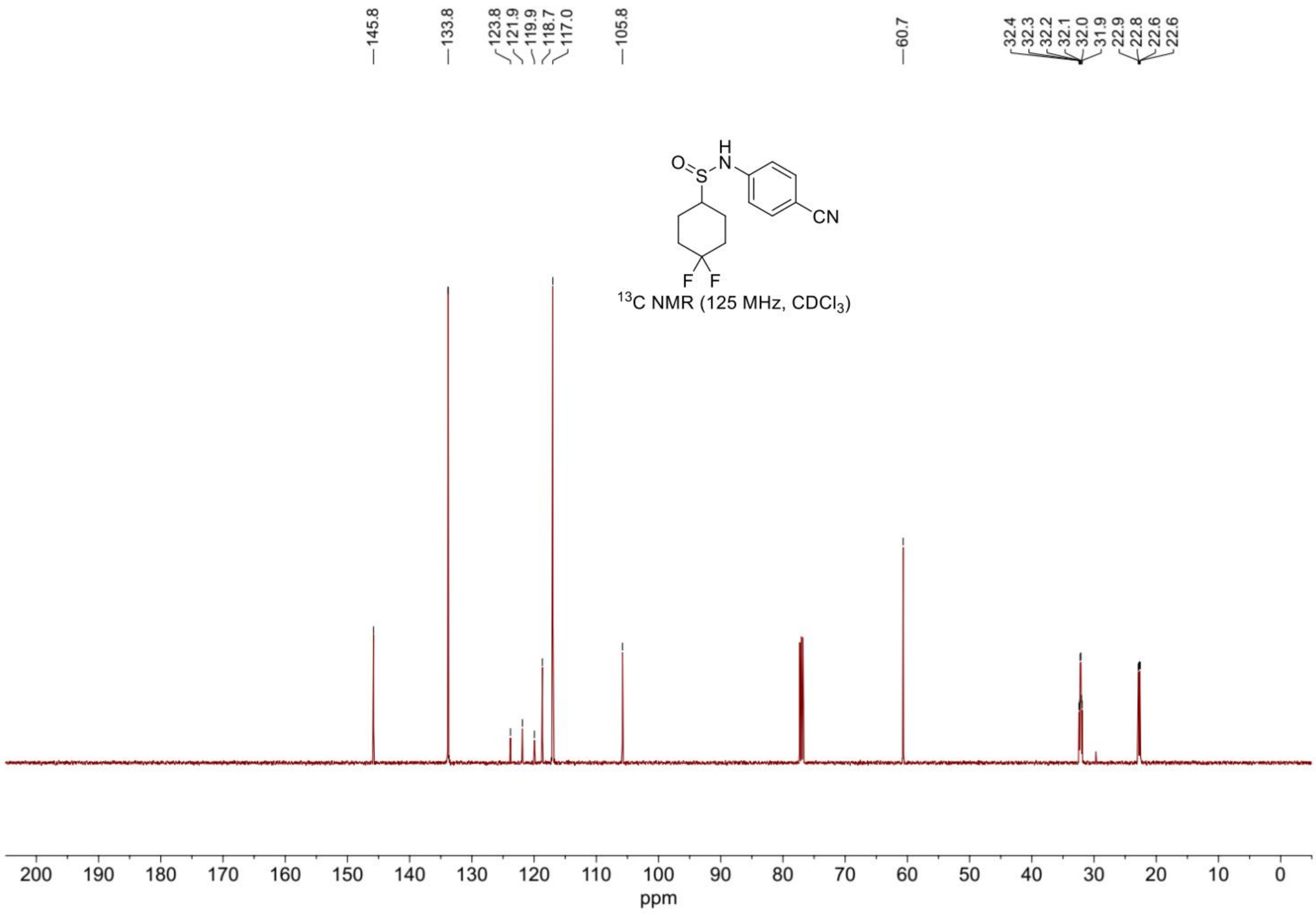

Go to table of contents

4-Methyl-*N*-(3-(trifluoromethoxy)phenyl)tetrahydro-2*H*-pyran-4-sulfinamide (4b)

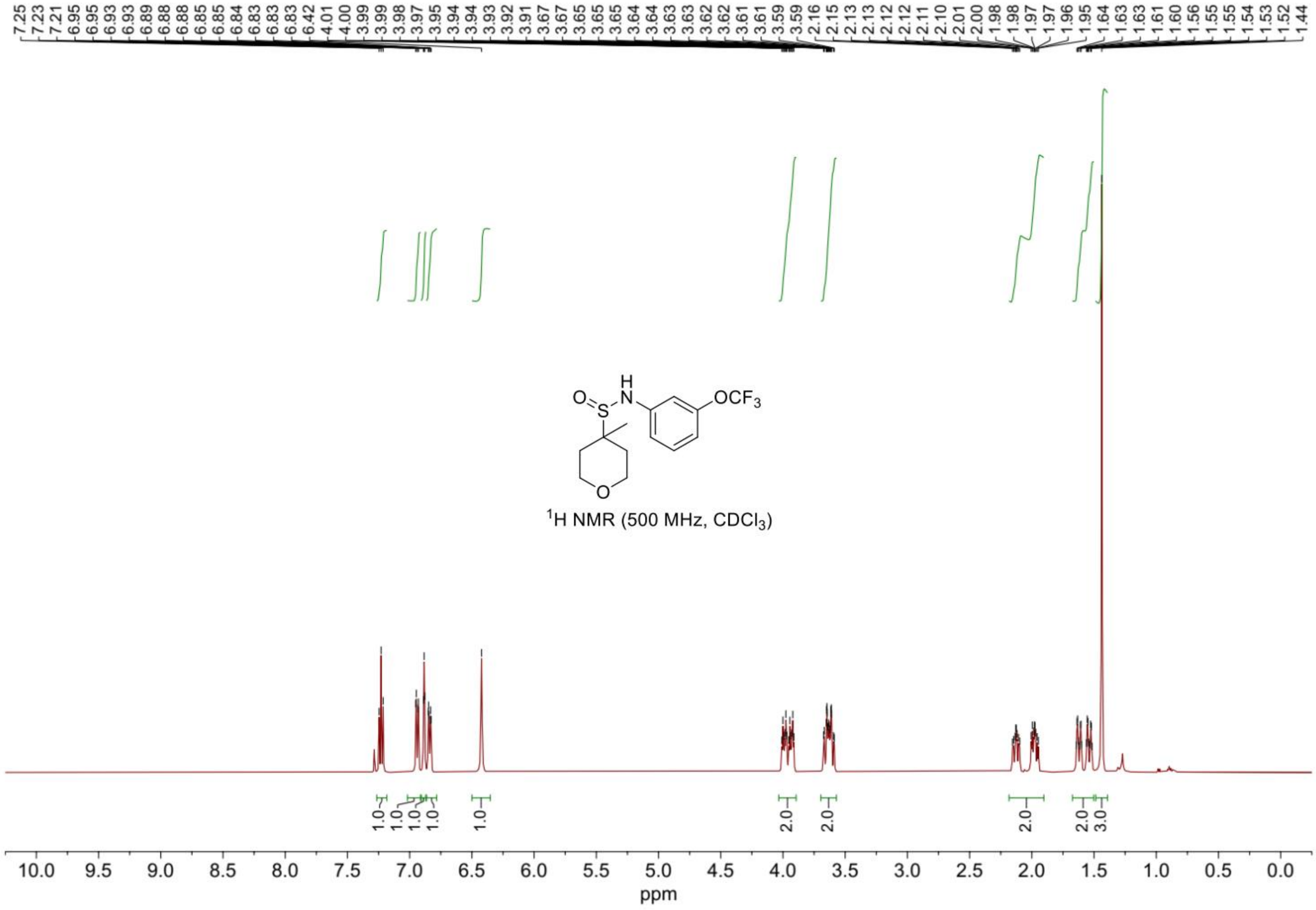

Go to table of contents

4-Methyl-*N*-(3-(trifluoromethoxy)phenyl)tetrahydro-2*H*-pyran-4-sulfinamide (4b)

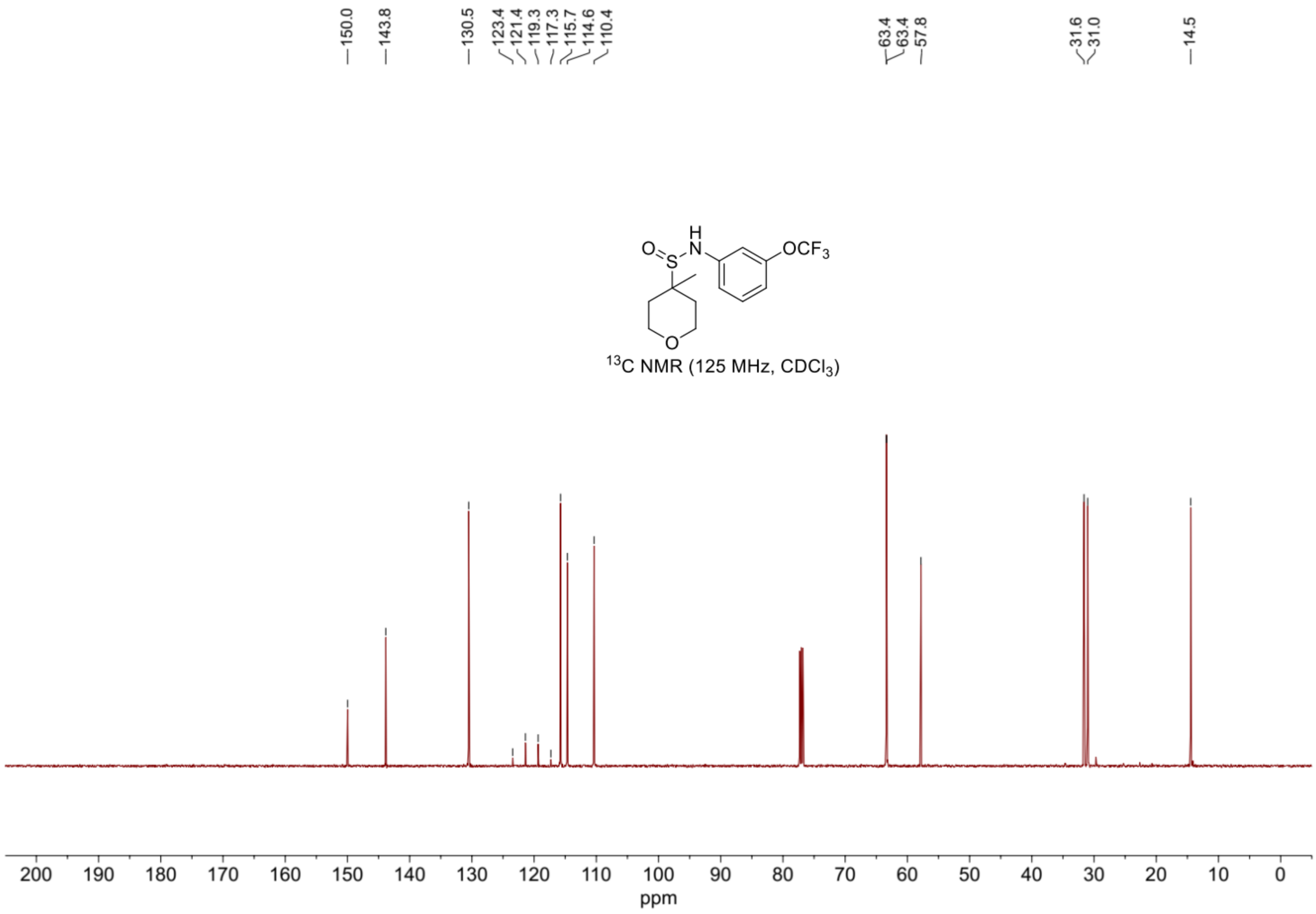

Go to table of contents

N-(2,4,5-Trifluorophenyl)tetrahydro-2H-pyran-4-sulfinamide (4c)

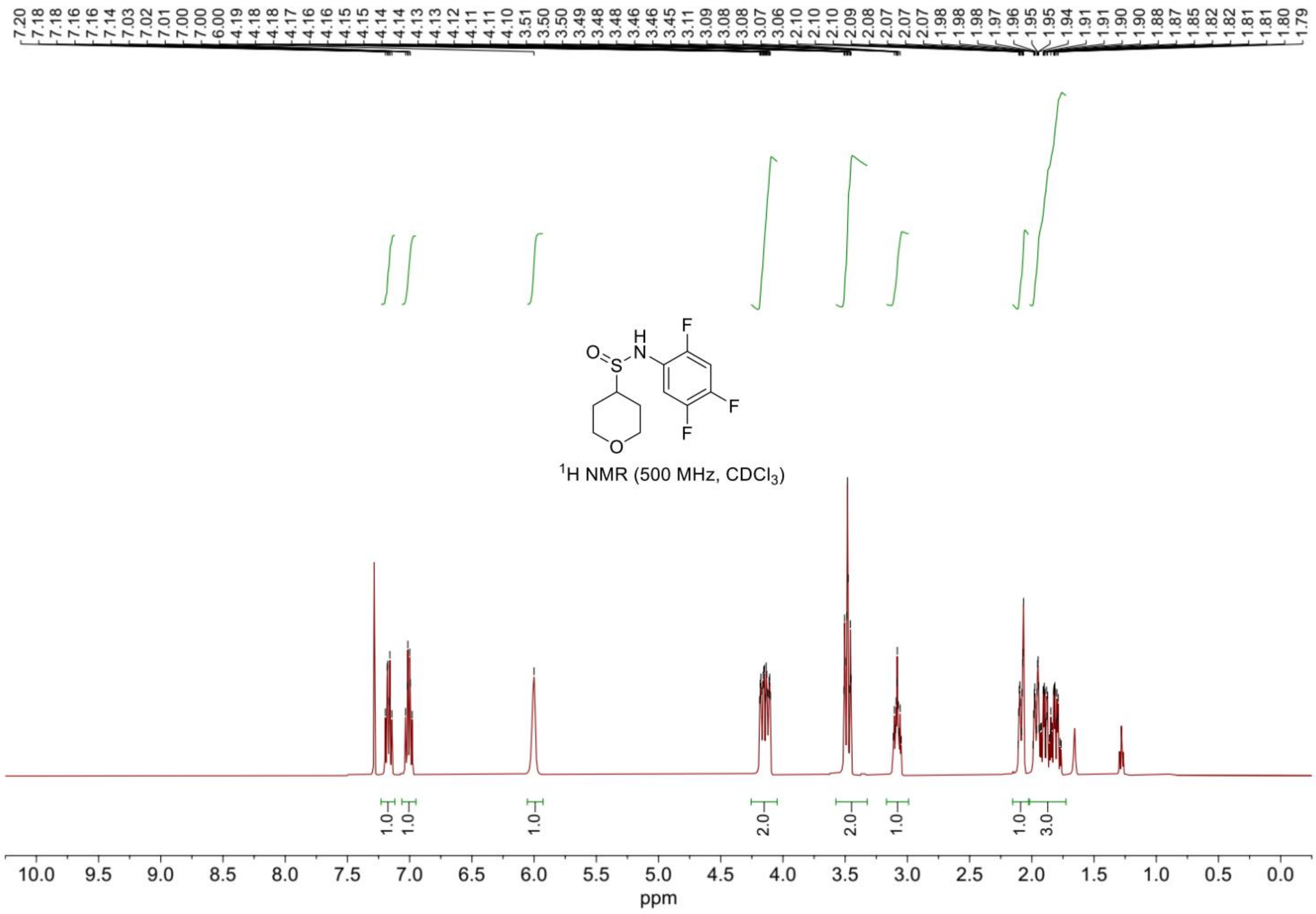

Go to table of contents

N-(2,4,5-Trifluorophenyl)tetrahydro-2H-pyran-4-sulfinamide (4c)

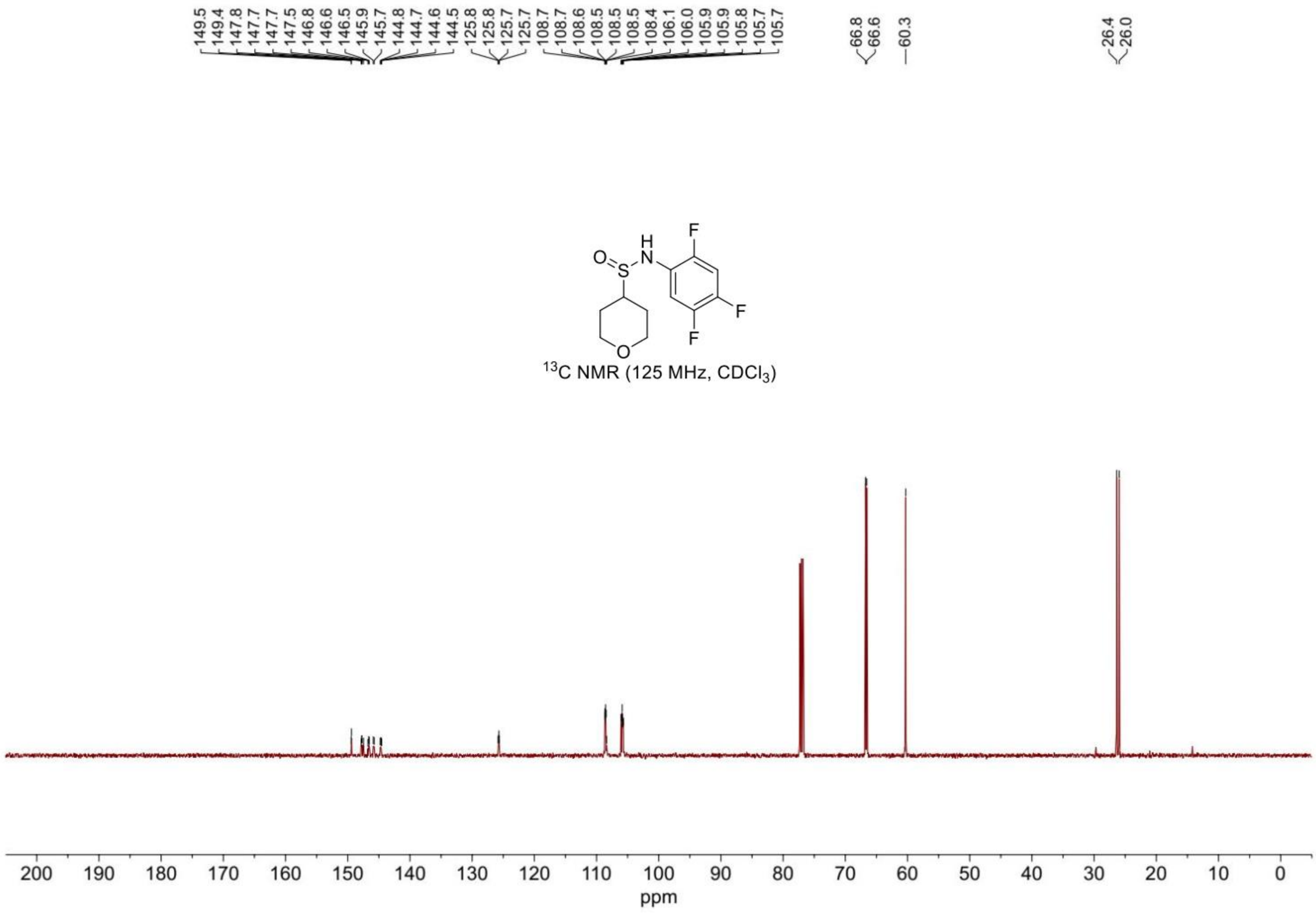

Go to table of contents

3,3-Dimethoxy-1-methyl-*N*-(pyridin-3-yl)cyclobutane-1-sulfinamide (4d)

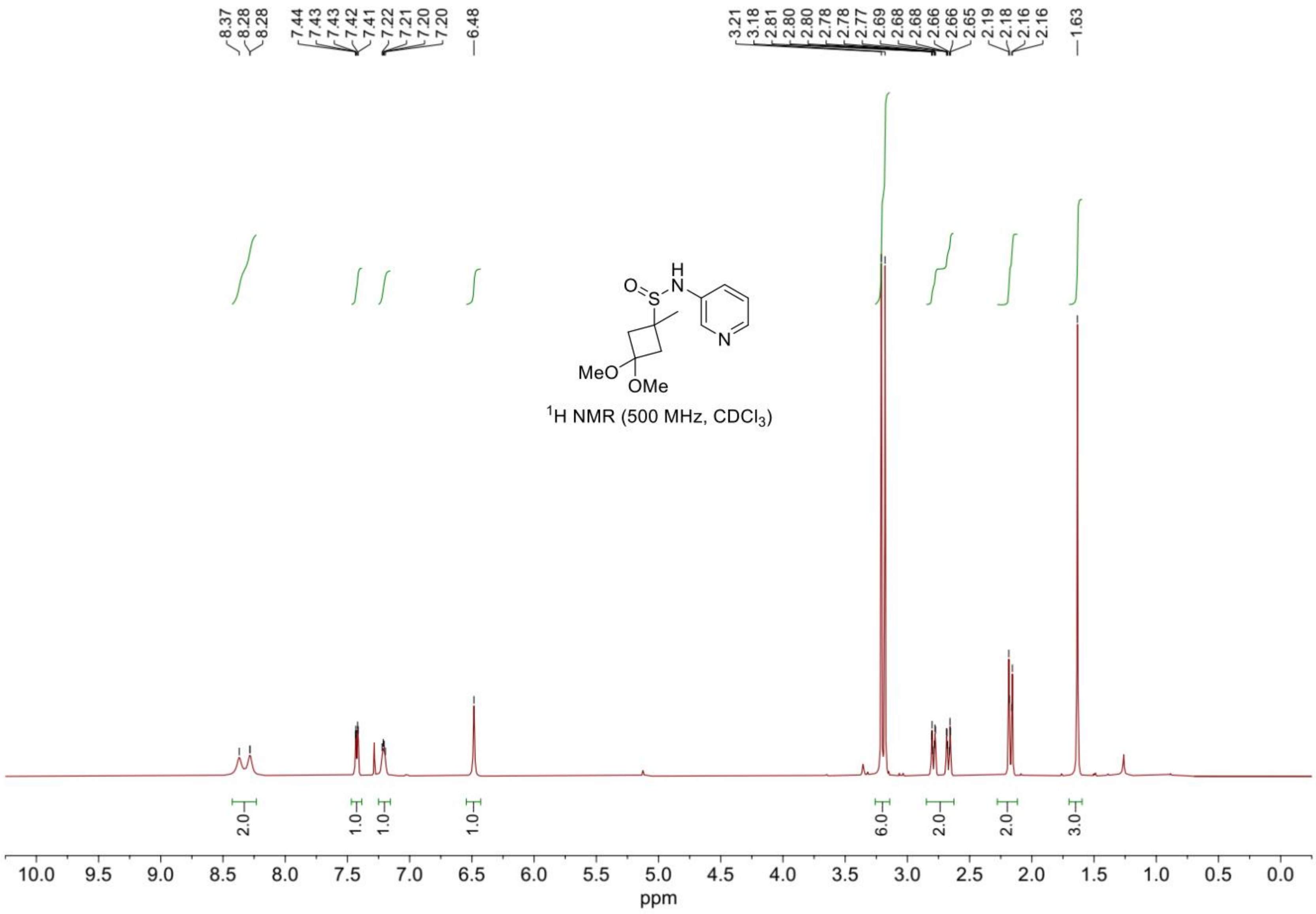

Go to table of contents

3,3-Dimethoxy-1-methyl-*N*-(pyridin-3-yl)cyclobutane-1-sulfinamide (4d)

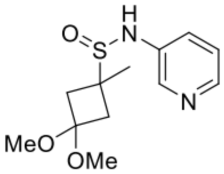

^{13}C NMR (125 MHz, $CDCl_3$)

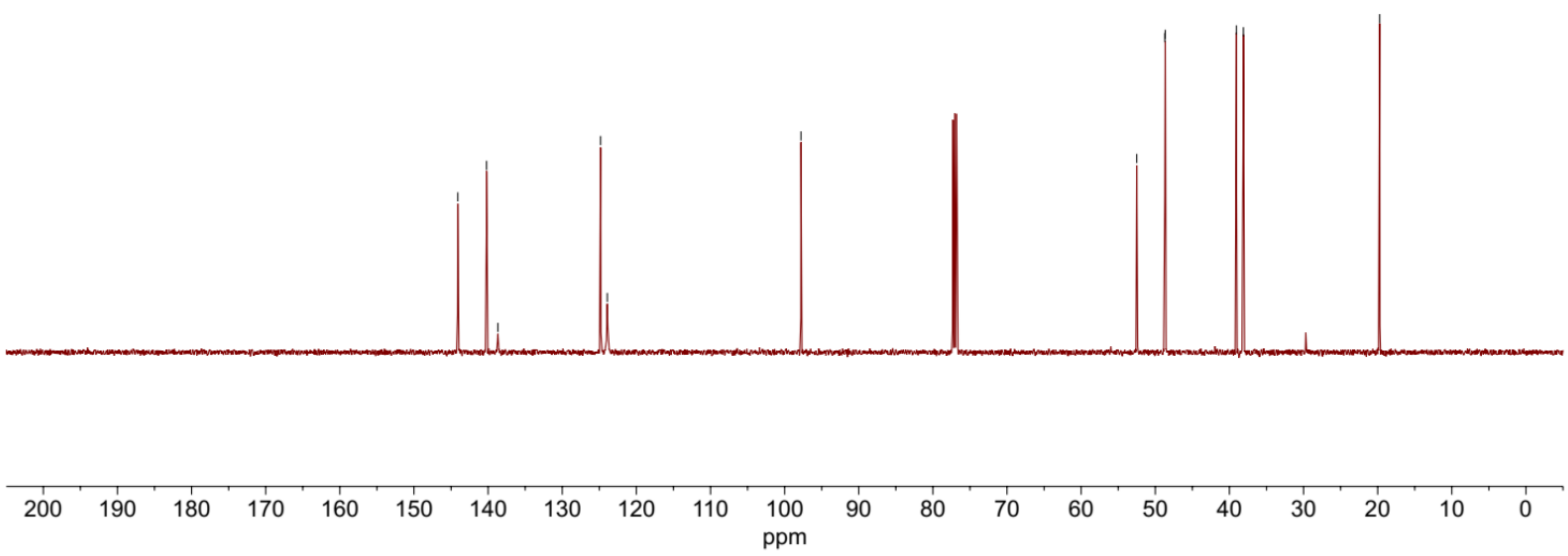

Go to table of contents

N-(6-Methylpyridin-2-yl)cyclopent-3-ene-1-sulfinamide (4e)

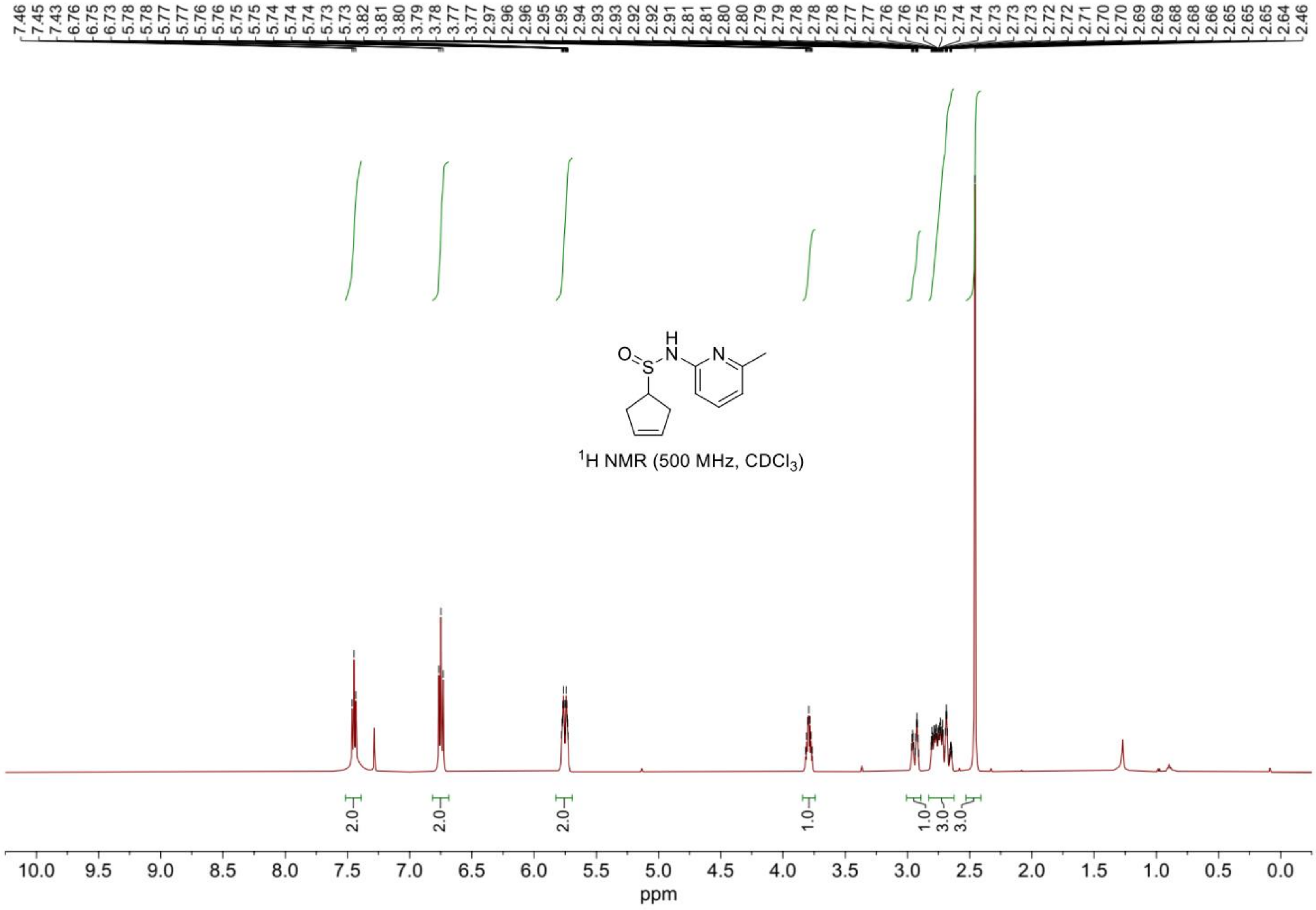

Go to table of contents

N-(6-Methylpyridin-2-yl)cyclopent-3-ene-1-sulfinamide (4e)

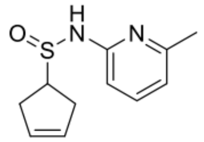

^{13}C NMR (125 MHz, $CDCl_3$)

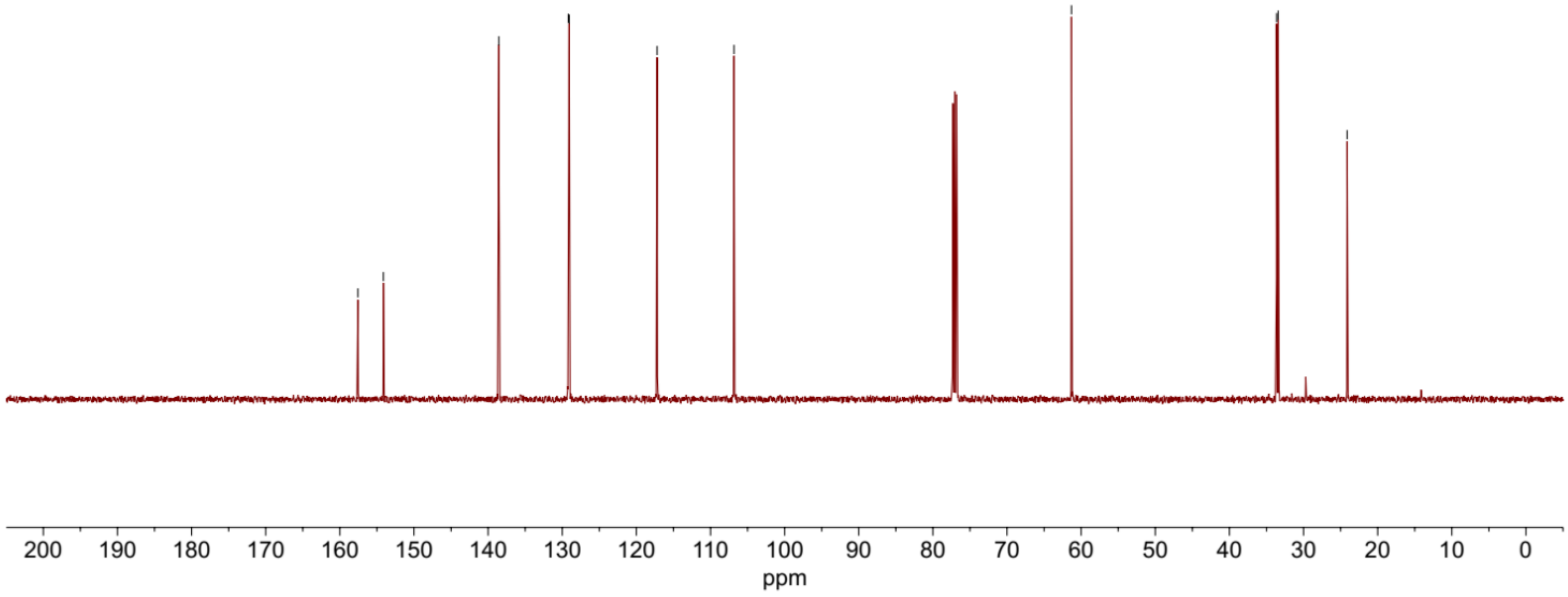

Go to table of contents

N-(5-Bromopyrimidin-2-yl)-5-chloropentane-1-sulfinamide (4f)

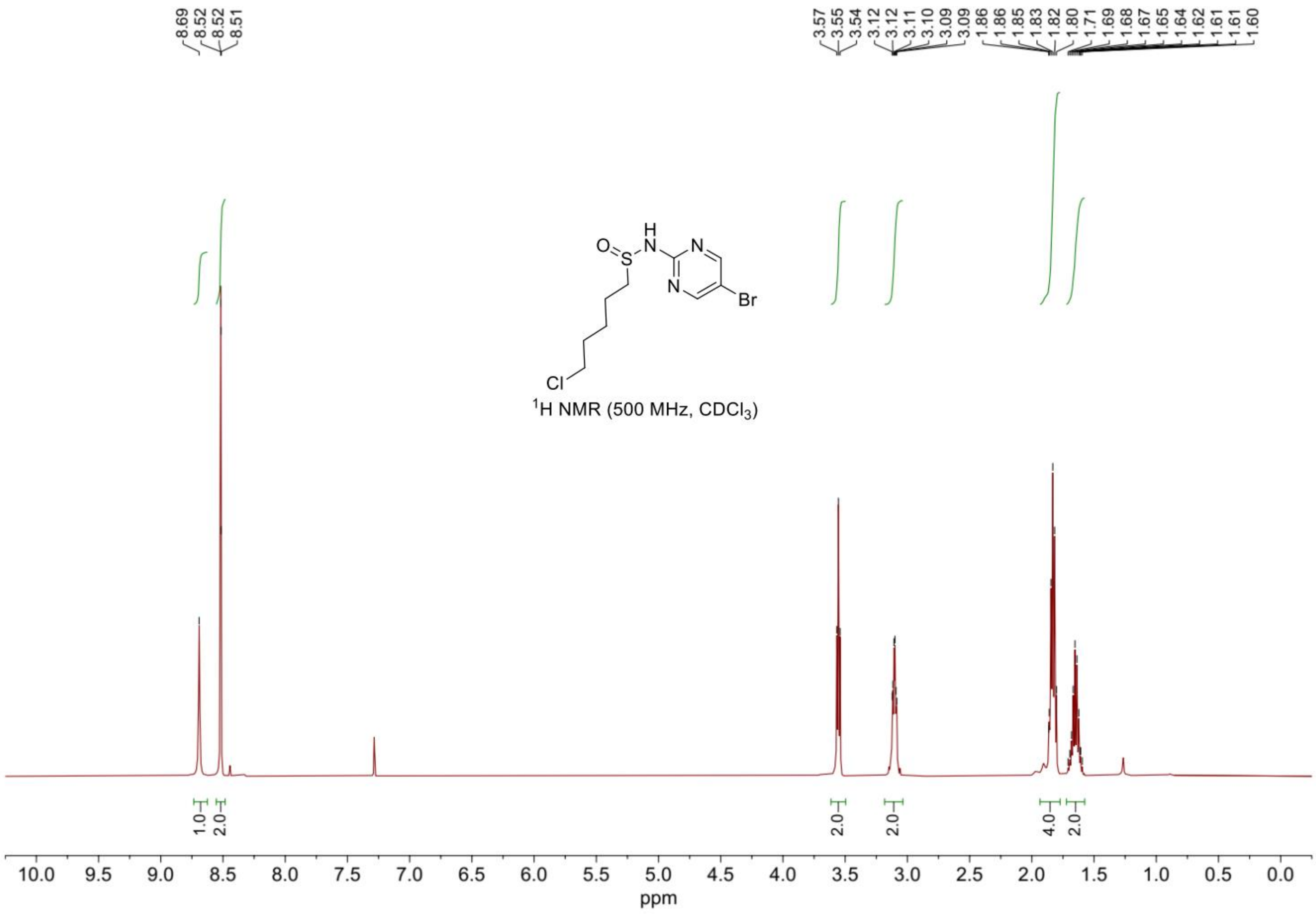

Go to table of contents

N-(5-Bromopyrimidin-2-yl)-5-chloropentane-1-sulfinamide (4f)

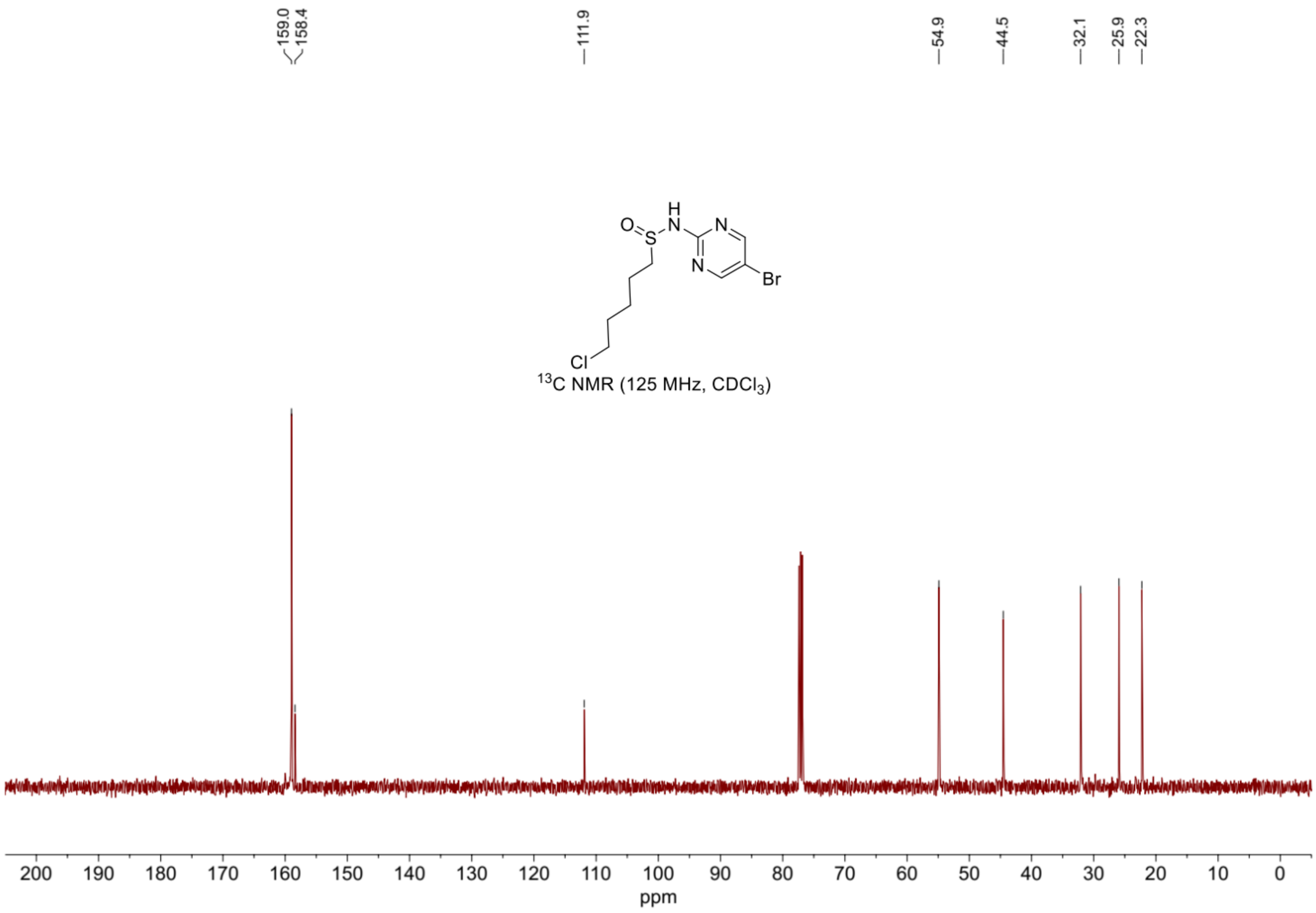

Go to table of contents

_N_-(Benzo[d]thiazol-5-yl)-1-methylcyclohexane-1-sulfinamide (4g)

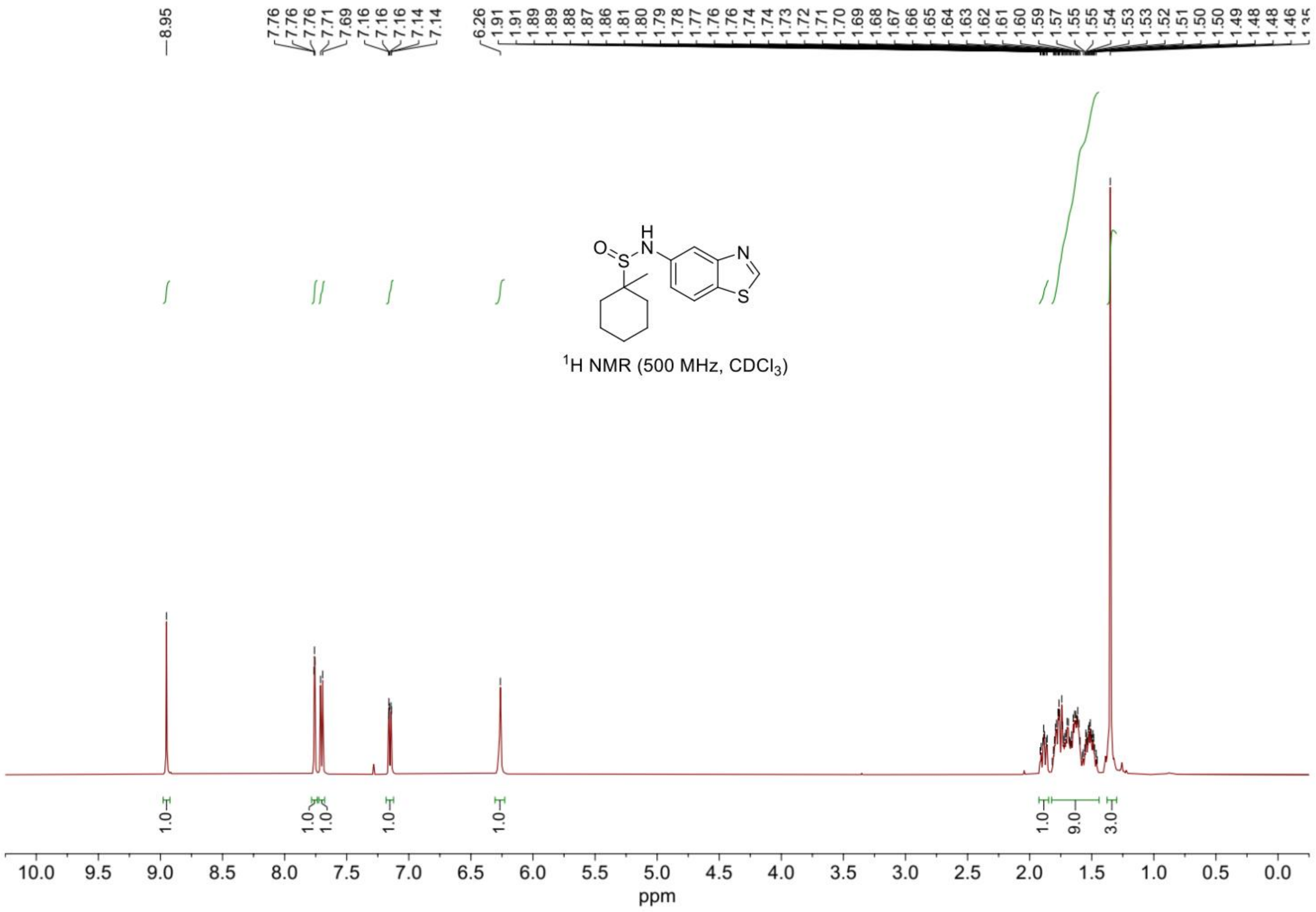

N-(Benzo[d]thiazol-5-yl)-1-methylcyclohexane-1-sulfinamide (4g)

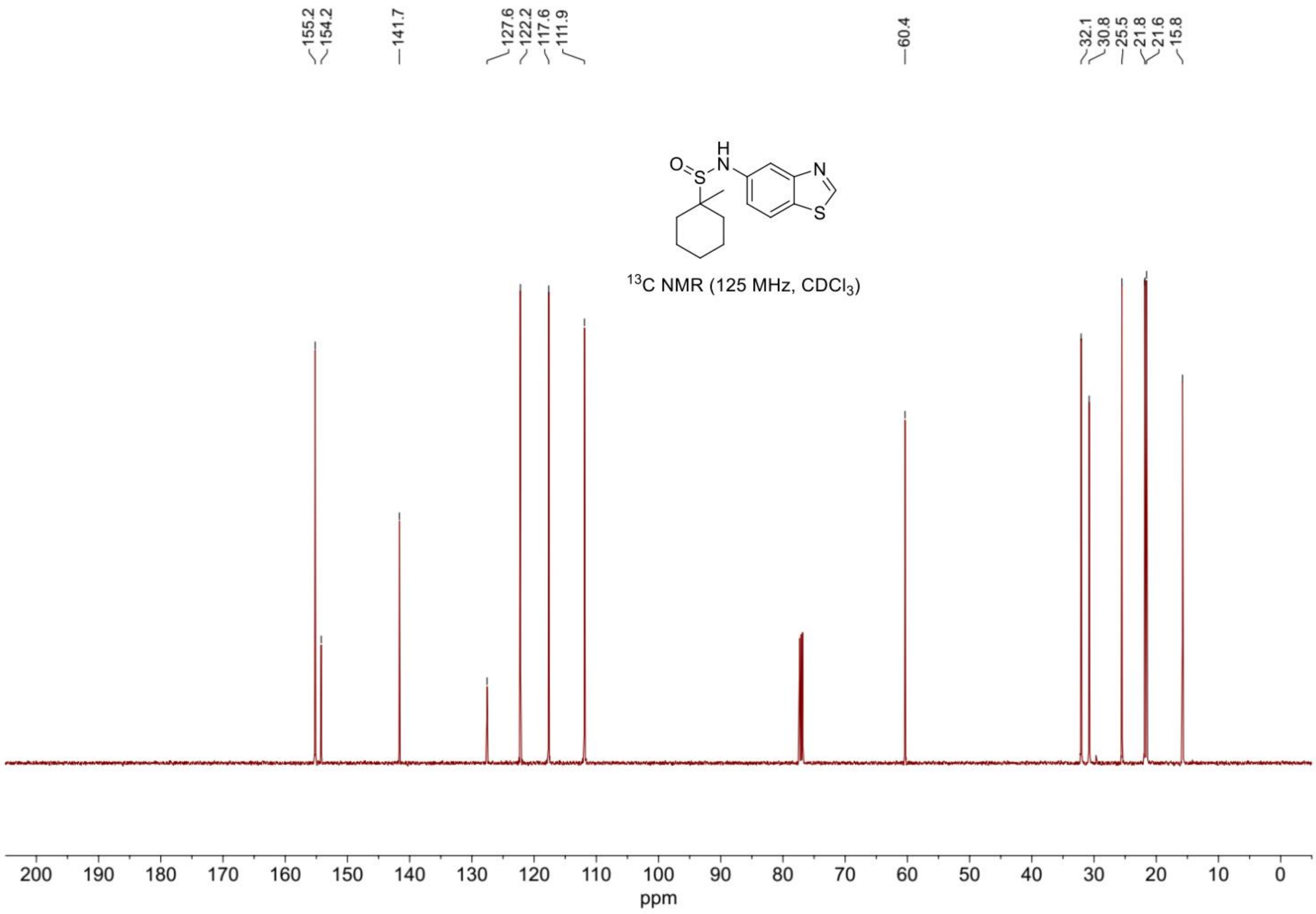

Go to table of contents

_N_-(4-Chlorophenethyl)-5-(2,5-dimethylphenoxy)-2-methylpentane-2-sulfinamide (4h)

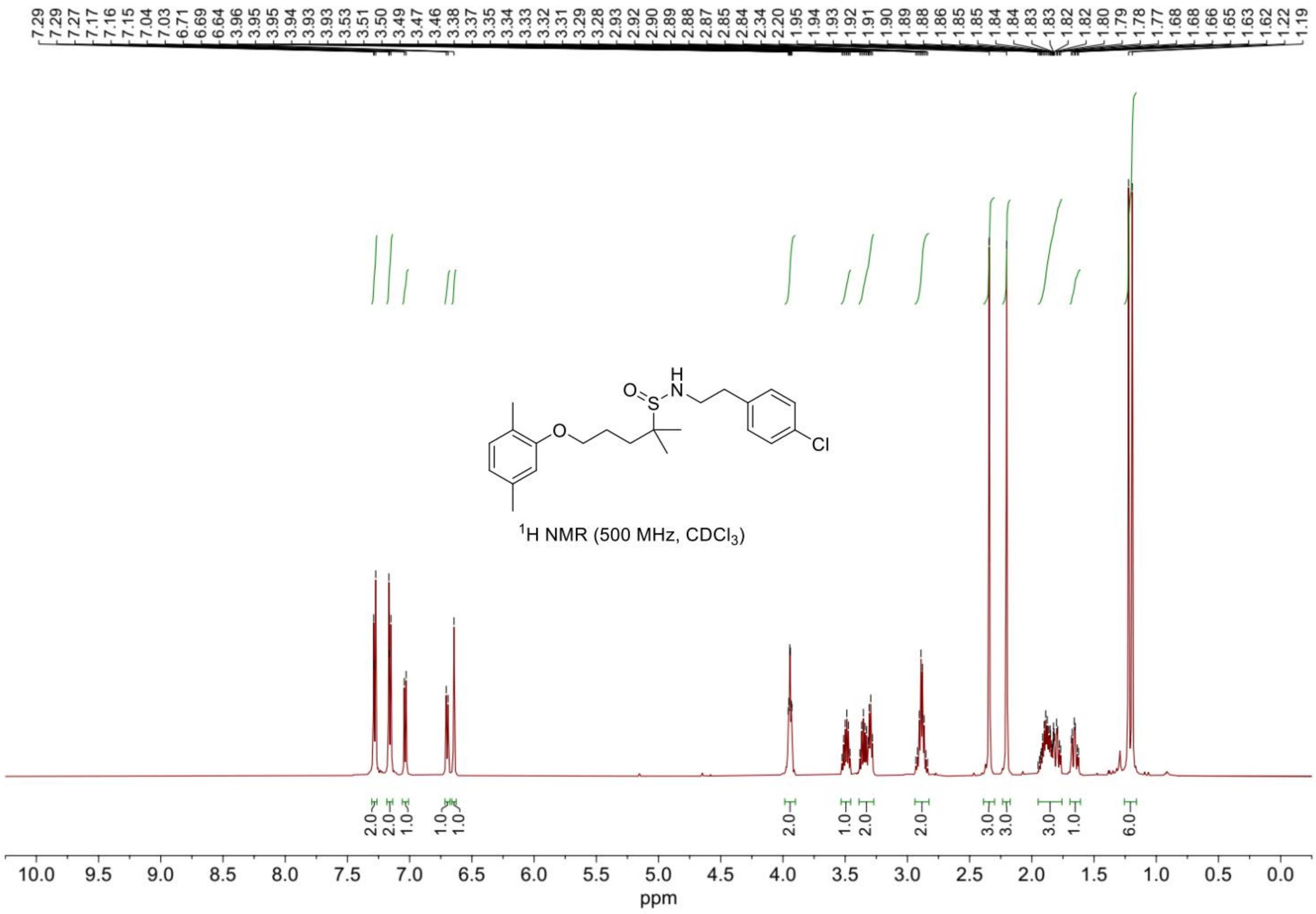

Go to table of contents

N-(4-Chlorophenethyl)-5-(2,5-dimethylphenoxy)-2-methylpentane-2-sulfinamide (4h)

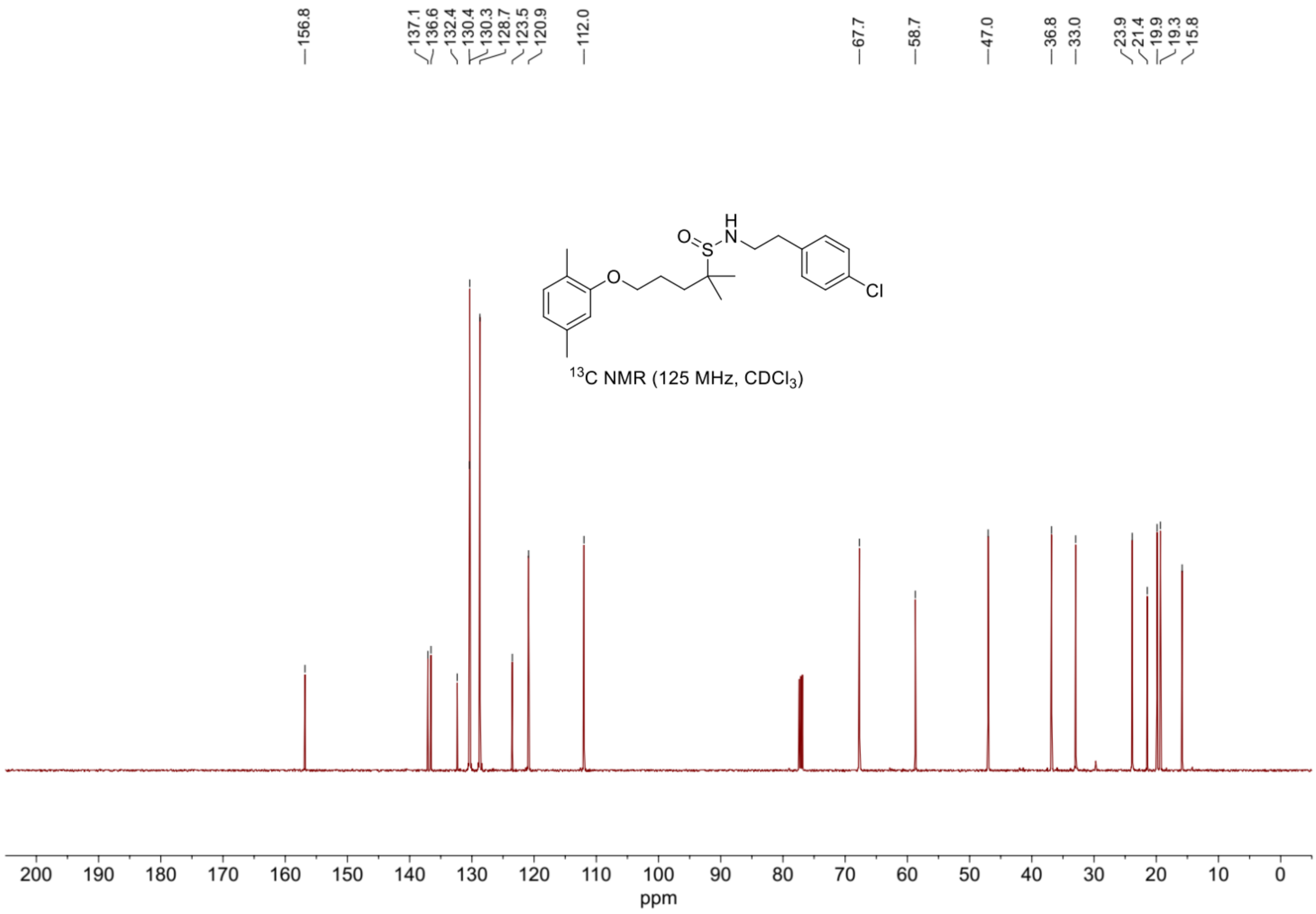

Go to table of contents

_N_-(1-Phenylethyl)pentadecane-7-sulfinamide (4i-1)

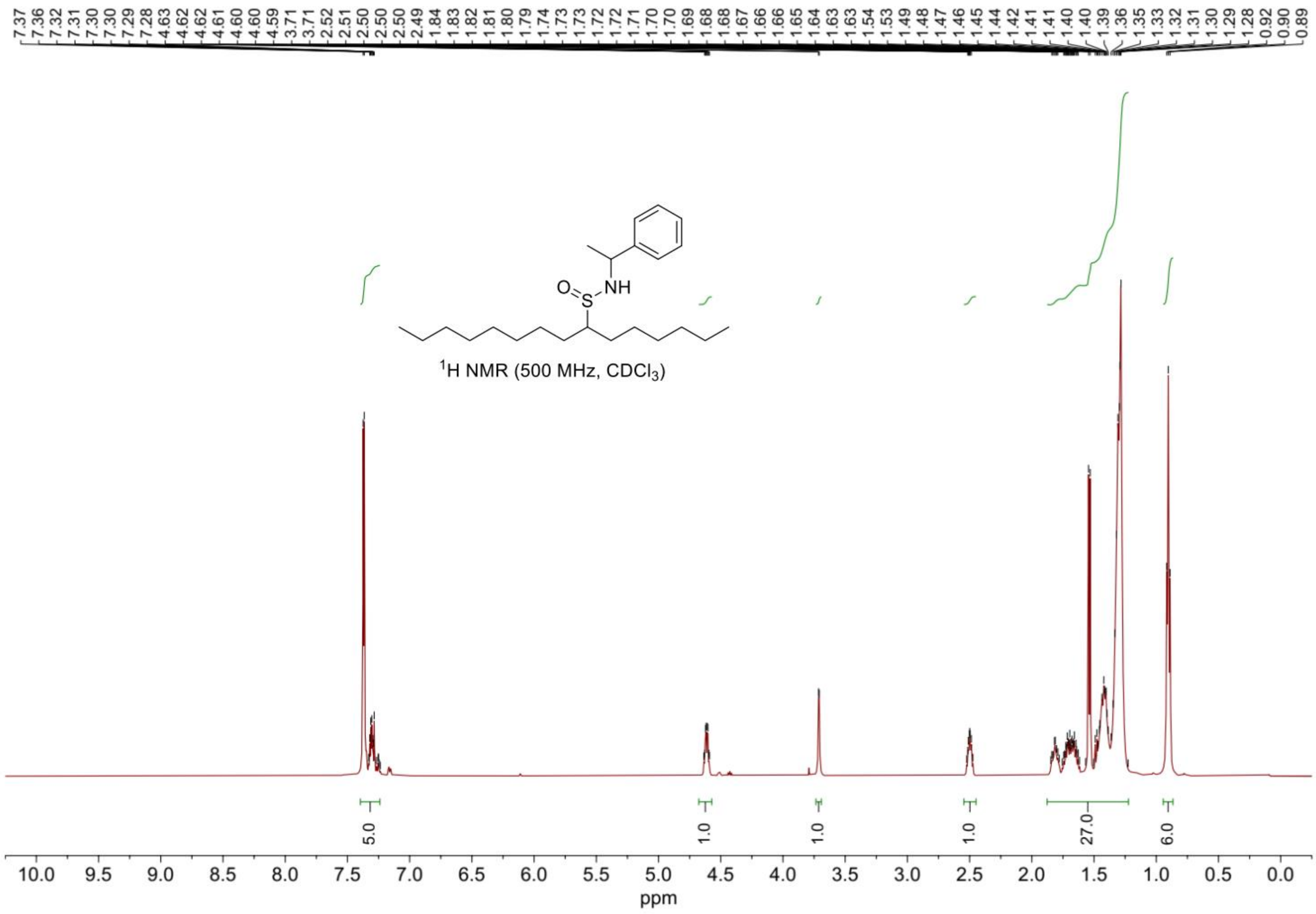

Go to table of contents

N-(1-Phenylethyl)pentadecane-7-sulfinamide (4i-1)

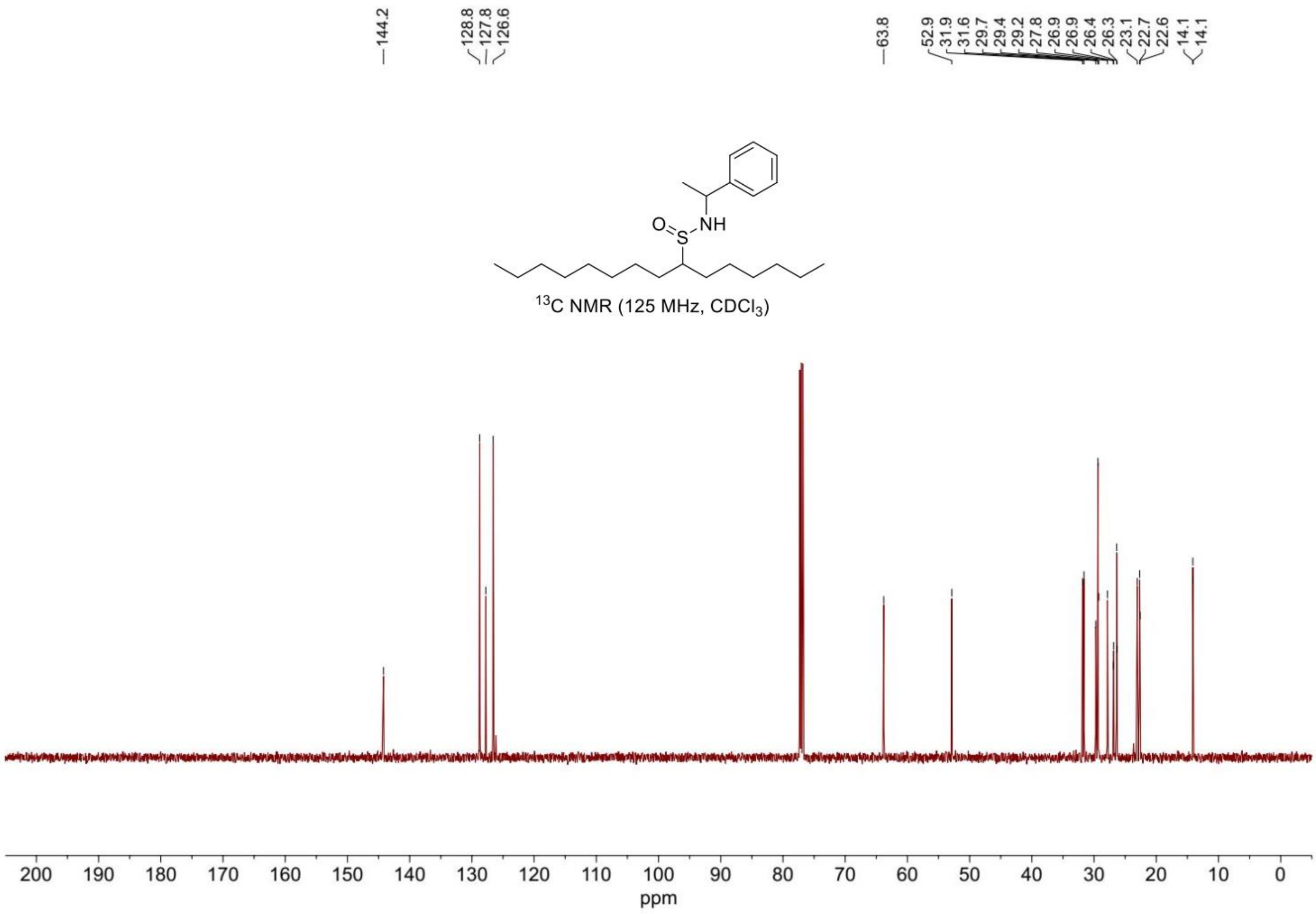

Go to table of contents

N-(1-Phenylethyl)pentadecane-7-sulfinamide (4i-2)

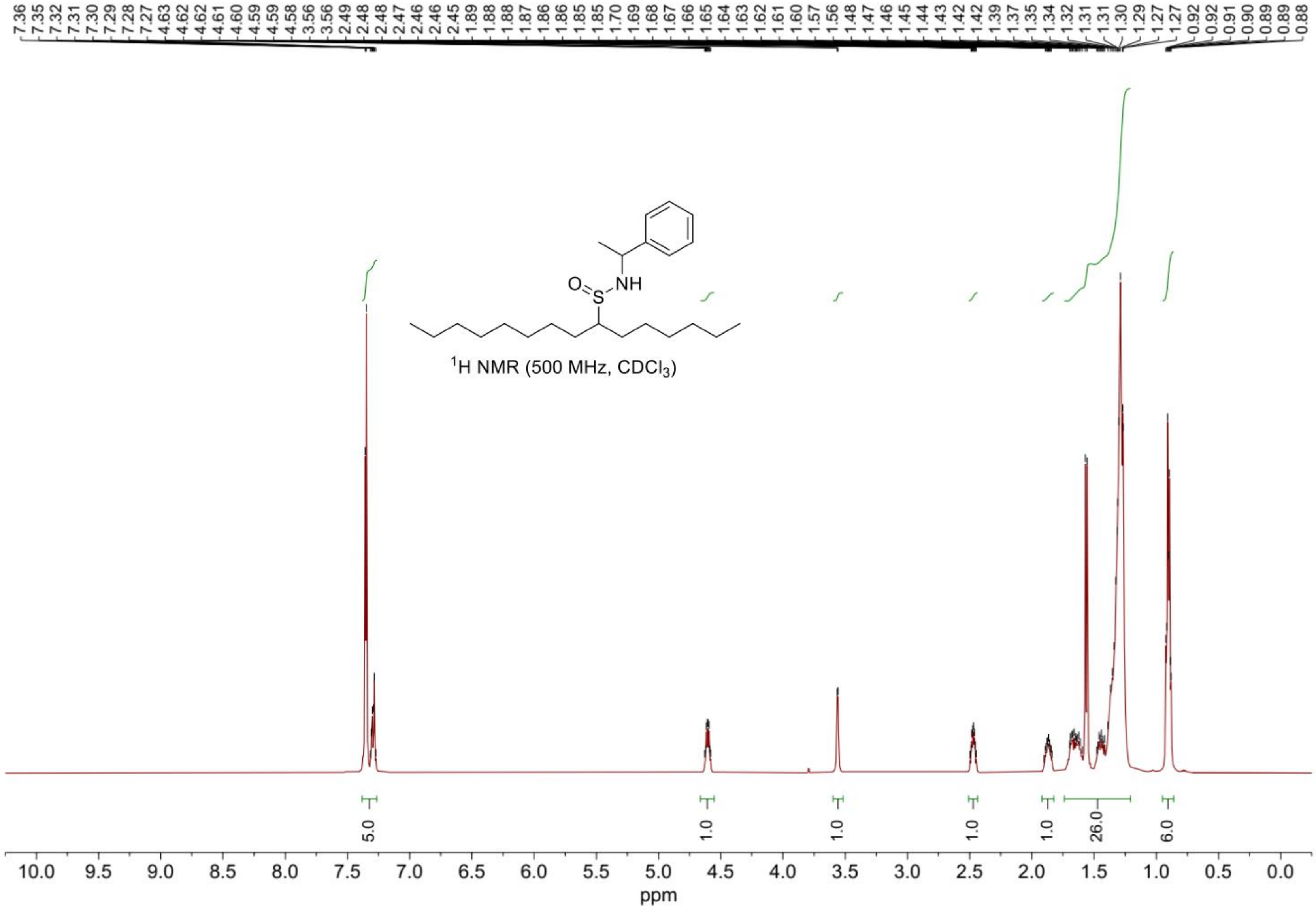

Go to table of contents

_N_-(1-Phenylethyl)pentadecane-7-sulfinamide (4i-2)

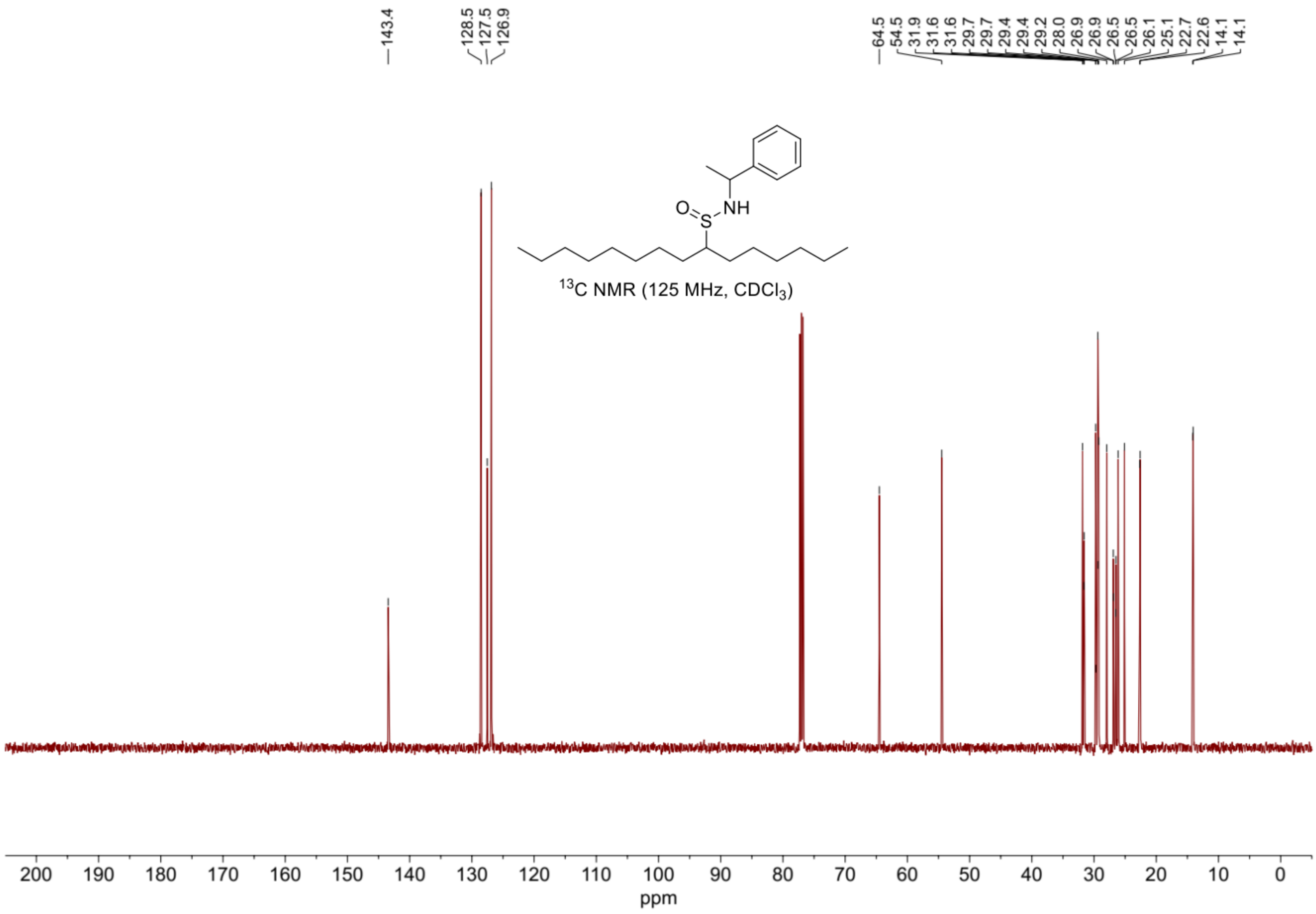

Go to table of contents

Nonane-1-sulfinamide (6a)

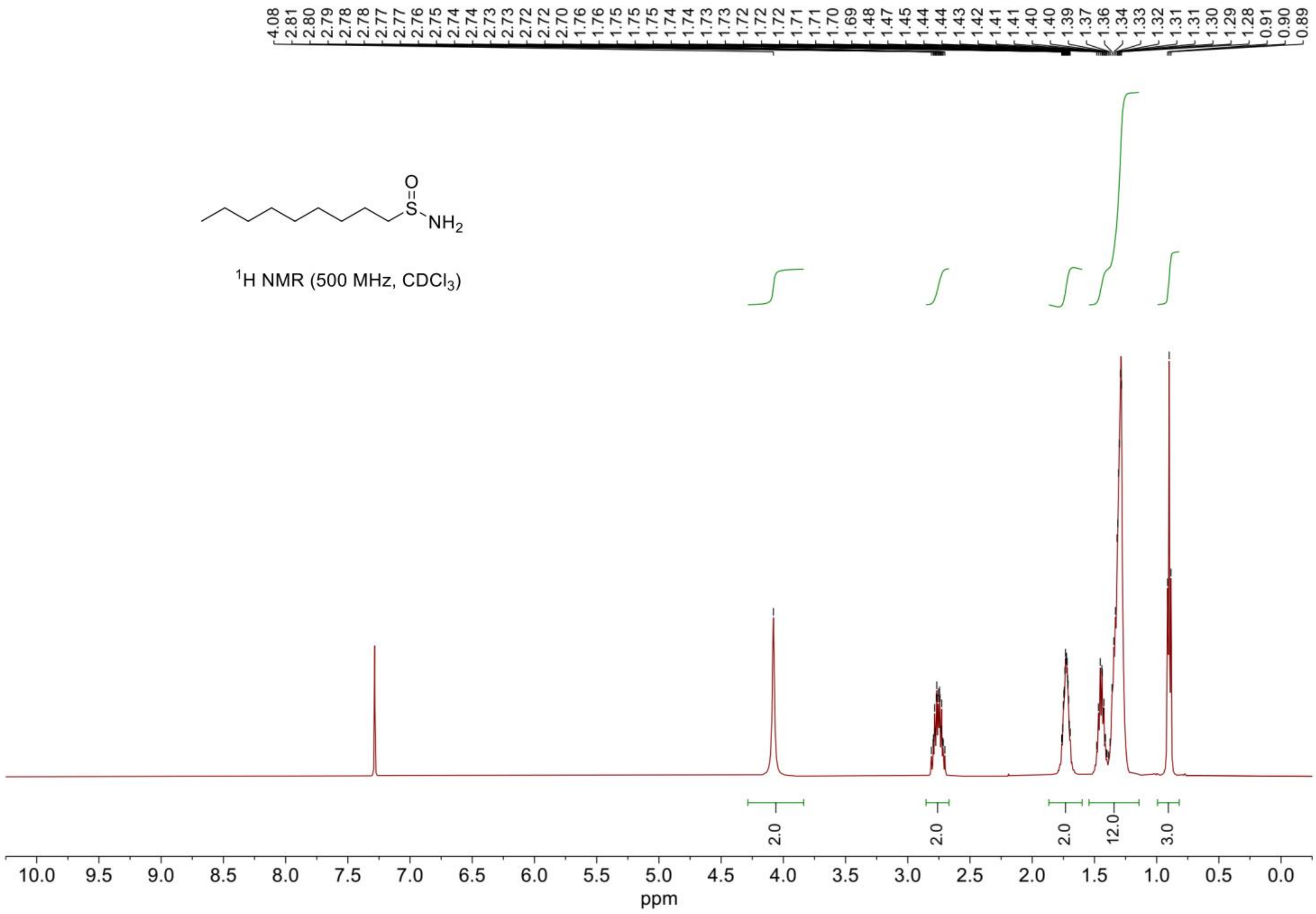

Go to table of contents

Nonane-1-sulfinamide (6a)

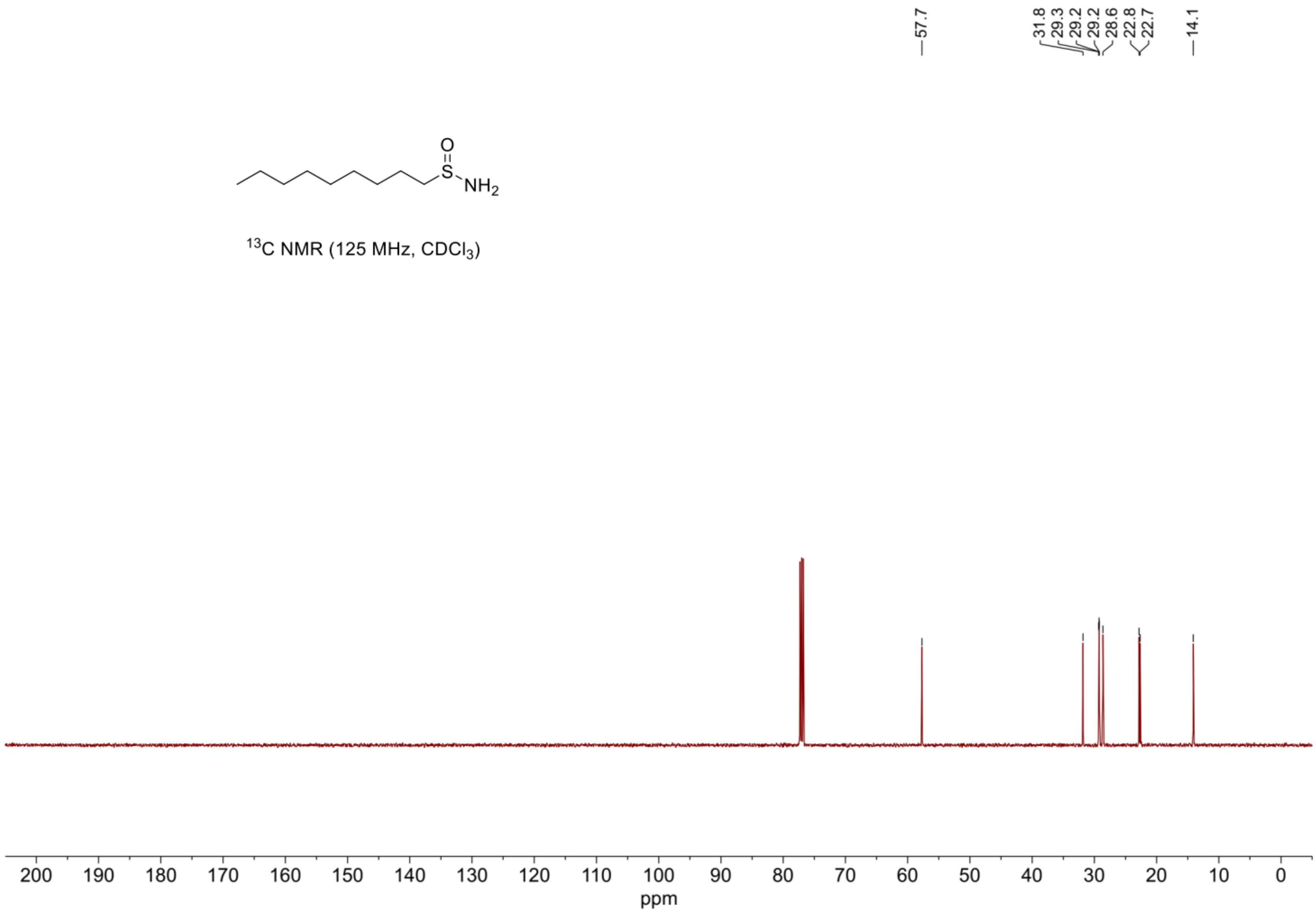

Go to table of contents

5-Chloropentane-1-sulfinamide (6b)

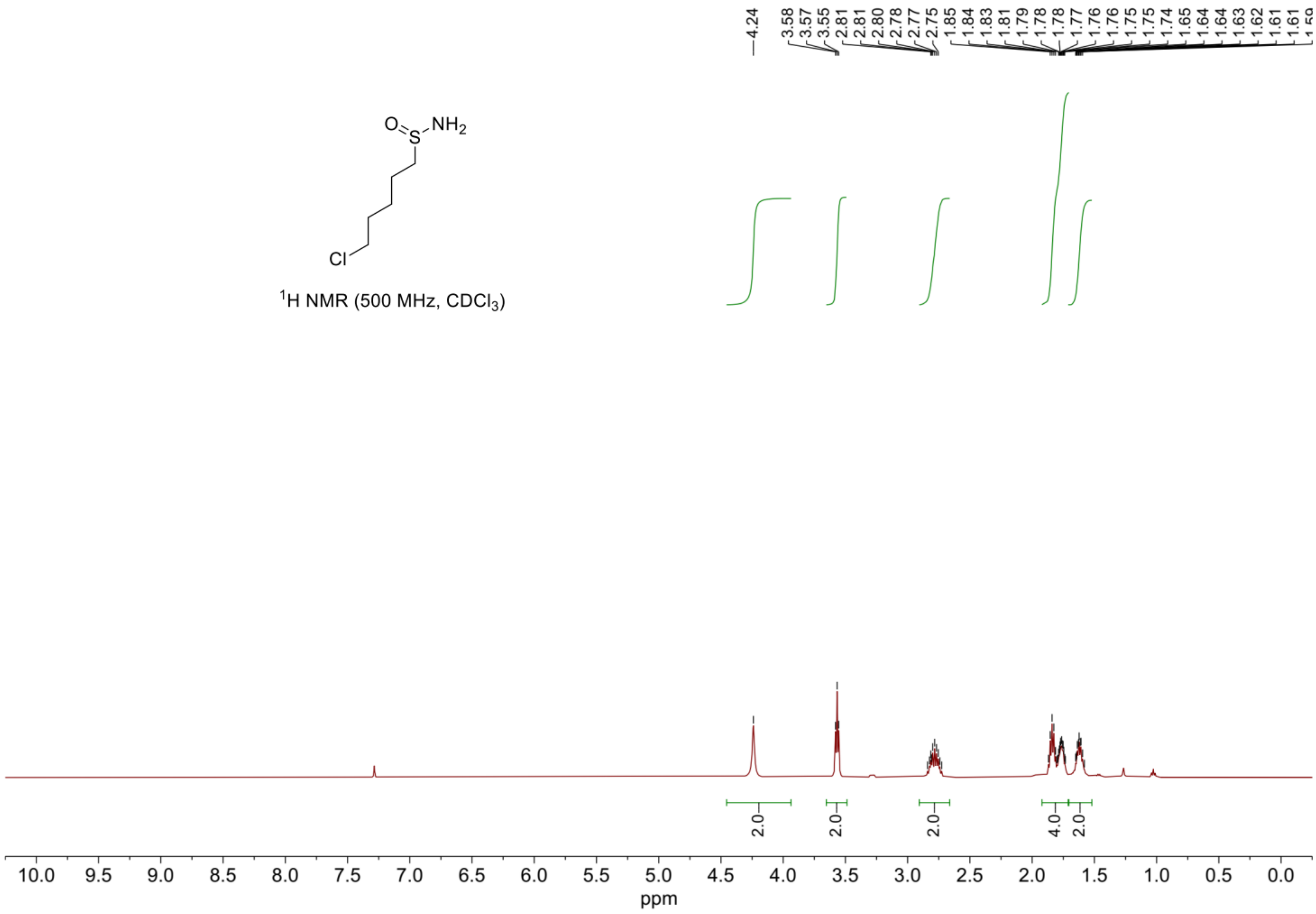

Go to table of contents

5-Chloropentane-1-sulfinamide (6b)

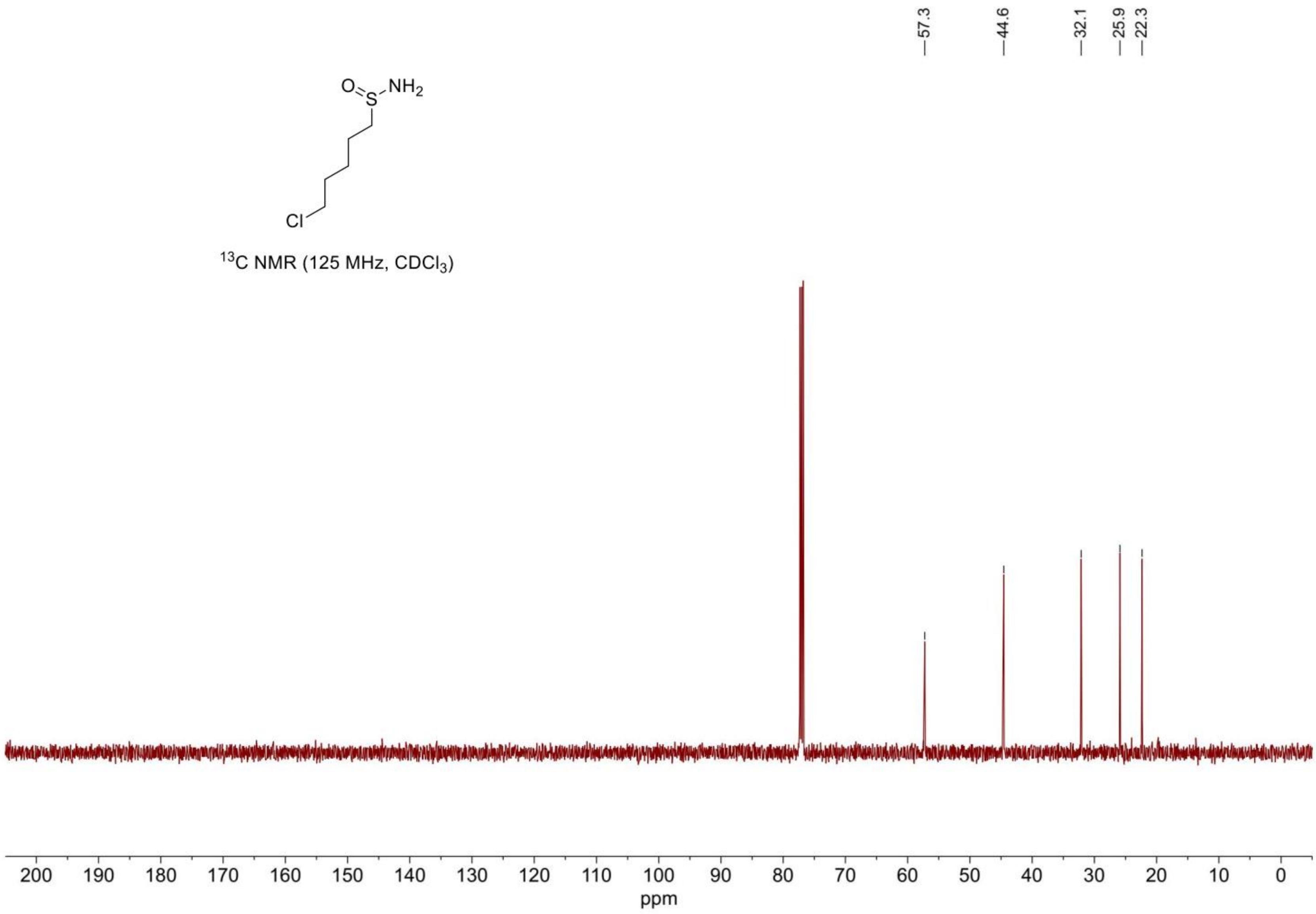

Go to table of contents

Cycloheptanesulfinamide (6c)

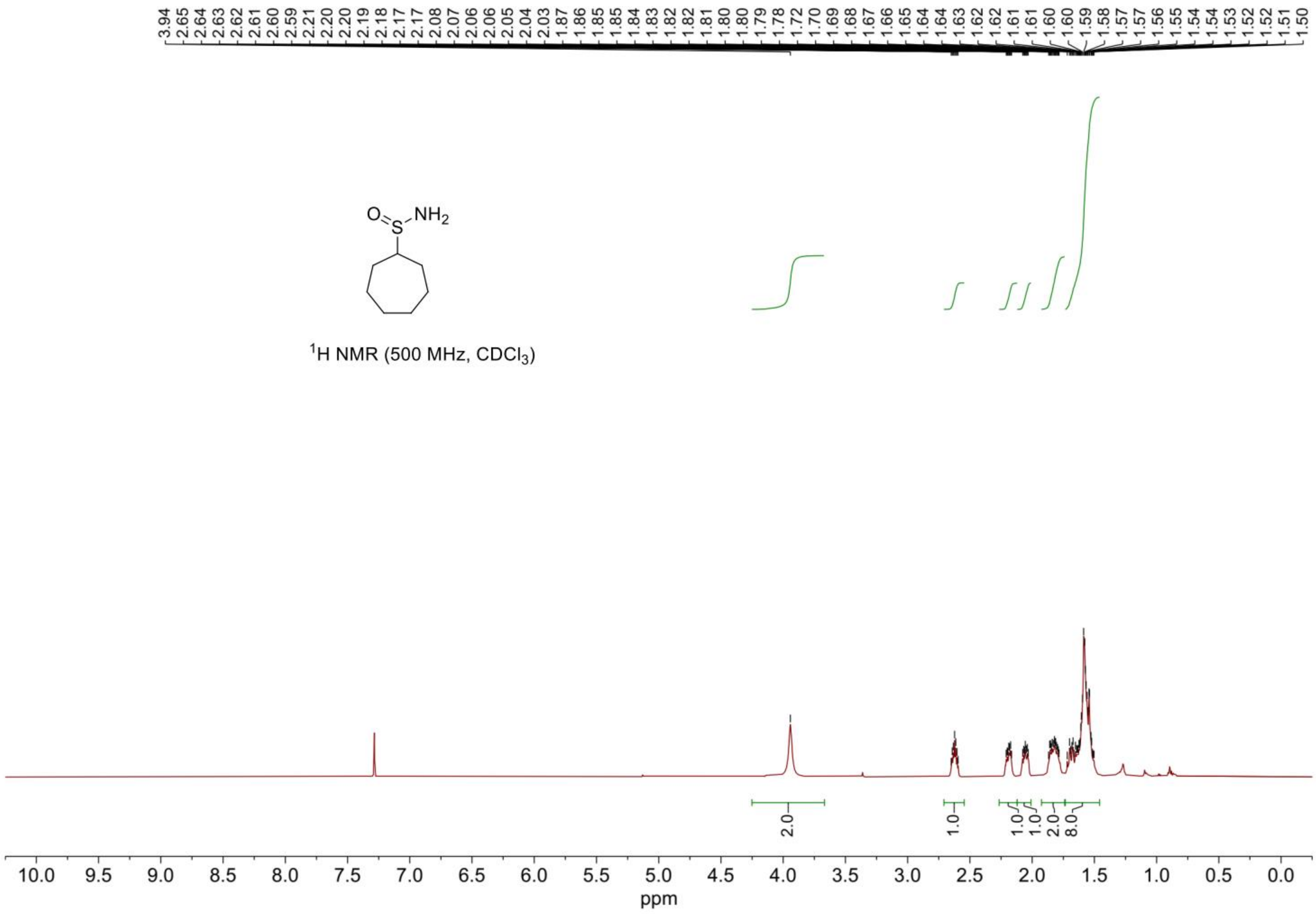

Go to table of contents

Cycloheptanesulfinamide (6c)

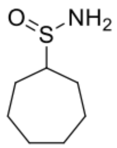

^{13}C NMR (125 MHz, $CDCl_3$)

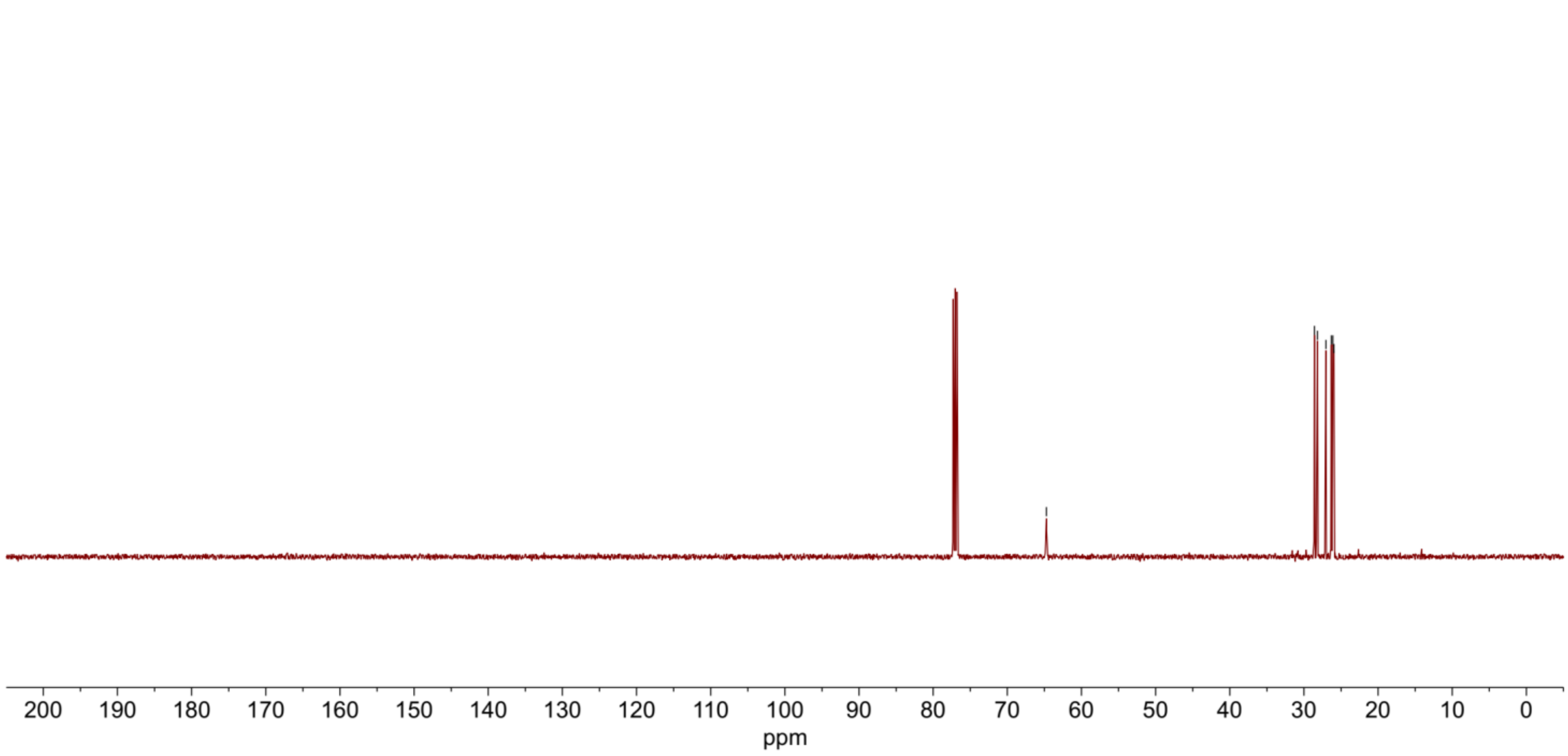

Go to table of contents

(1s,3*R*,5*S*,7s)-4-Oxoadamantane-1-sulfinamide (6d)

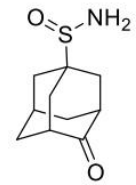

^1H NMR (500 MHz, $CDCl_3$)

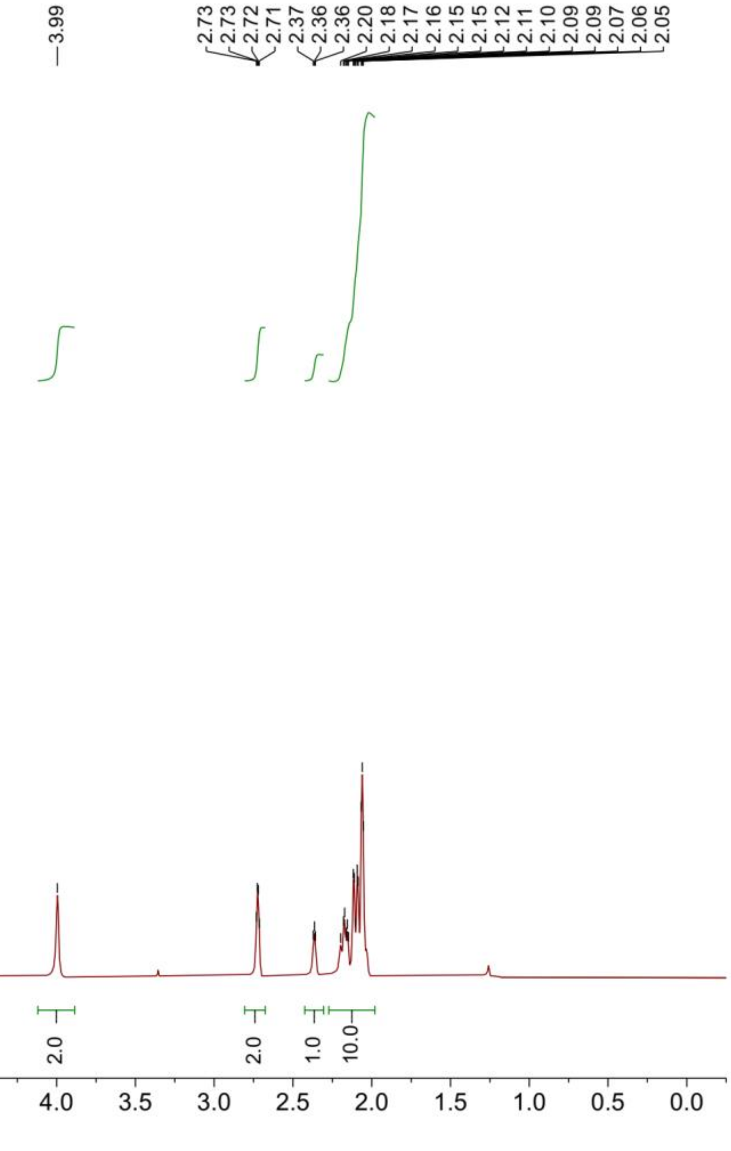

Go to table of contents

(1s,3*R*,5*S*,7s)-4-Oxoadamantane-1-sulfinamide (6d)

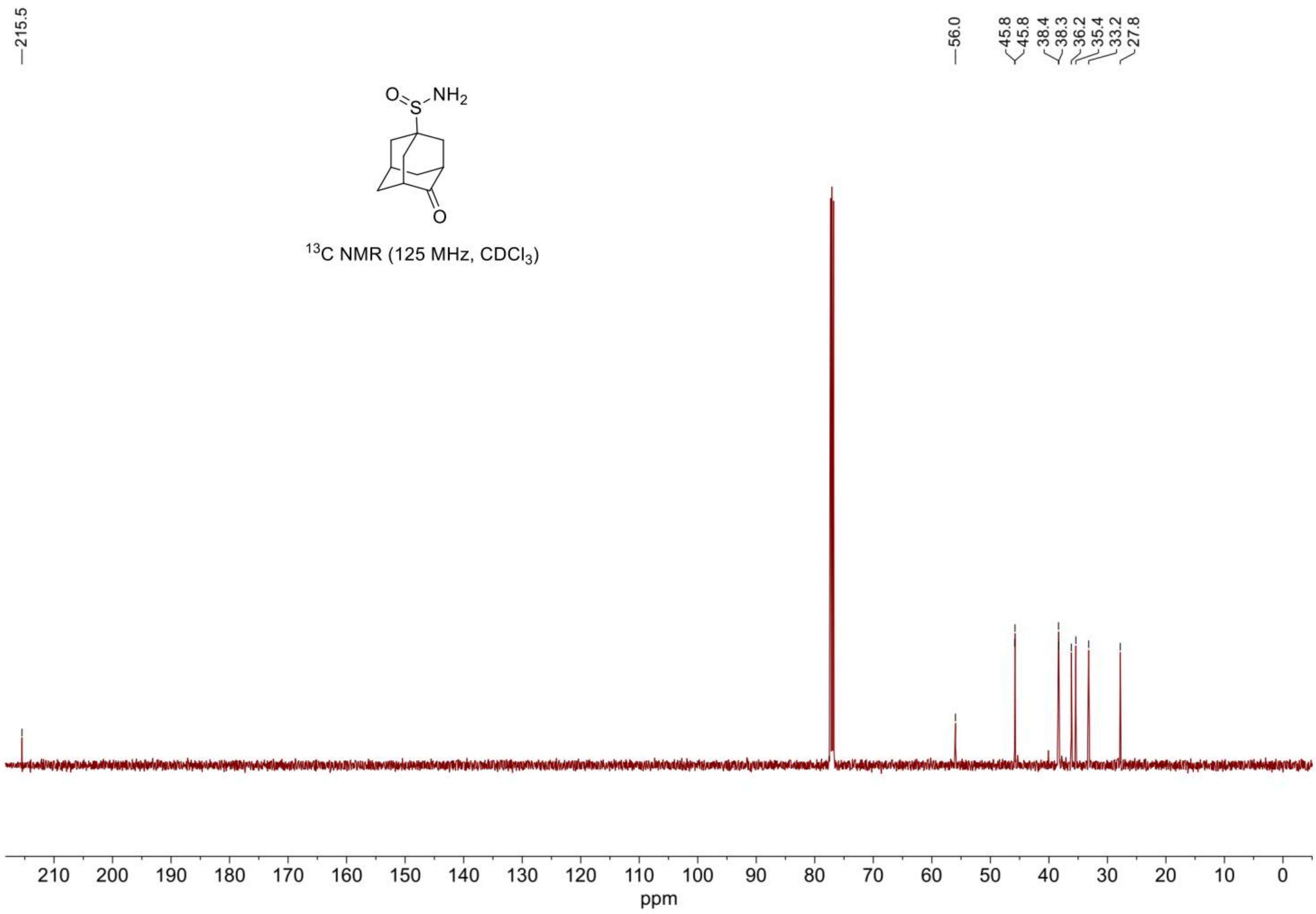

Go to table of contents

Methyl 4-(aminosulfinyl)bicyclo[2.2.2]octane-1-carboxylate (6e)

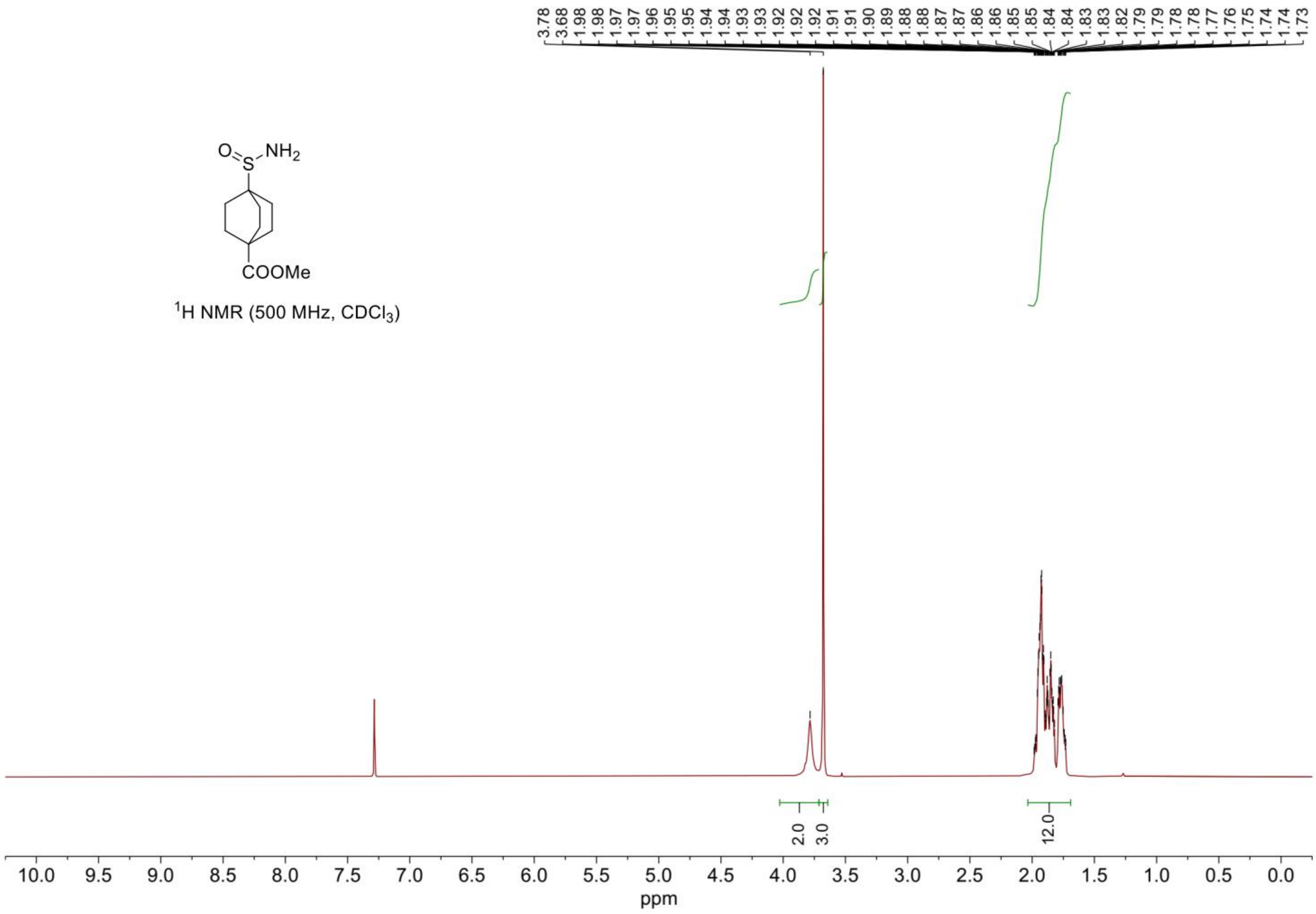

Go to table of contents

Methyl 4-(aminosulfinyl)bicyclo[2.2.2]octane-1-carboxylate (6e)

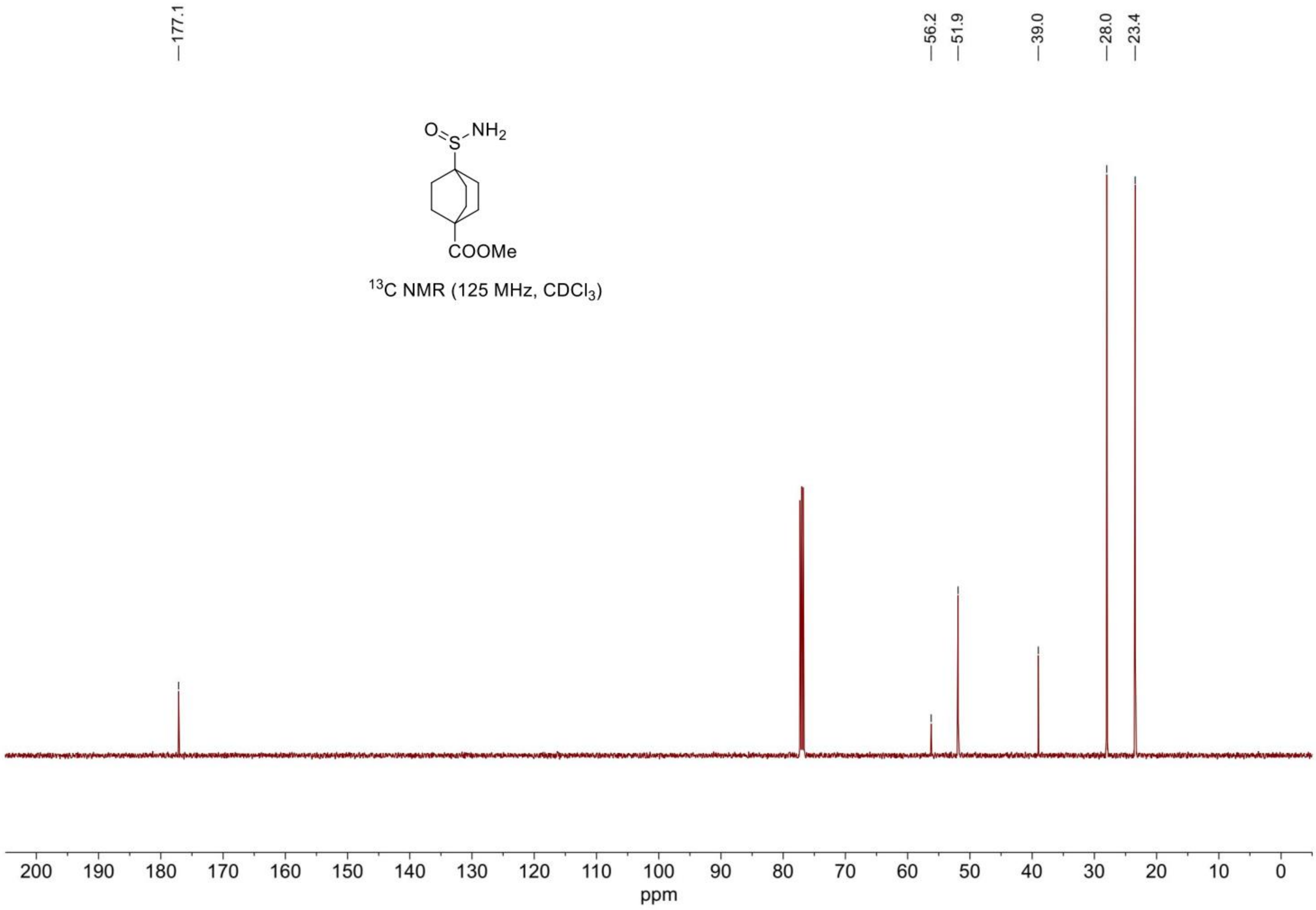

Go to table of contents

5-(2,5-Dimethylphenoxy)-2-methylpentane-2-sulfinamide (6f)

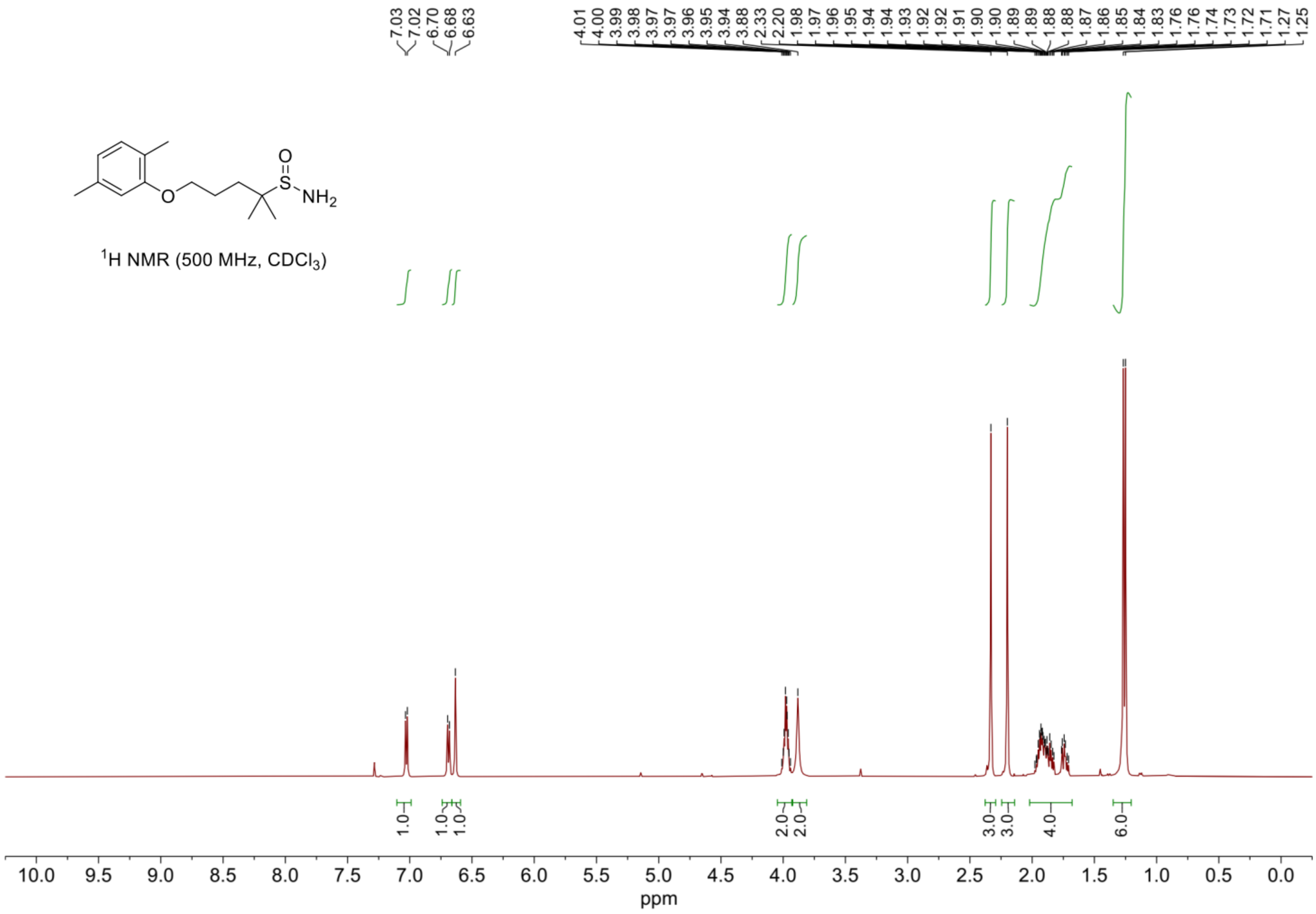

Go to table of contents

5-(2,5-Dimethylphenoxy)-2-methylpentane-2-sulfinamide (6f)

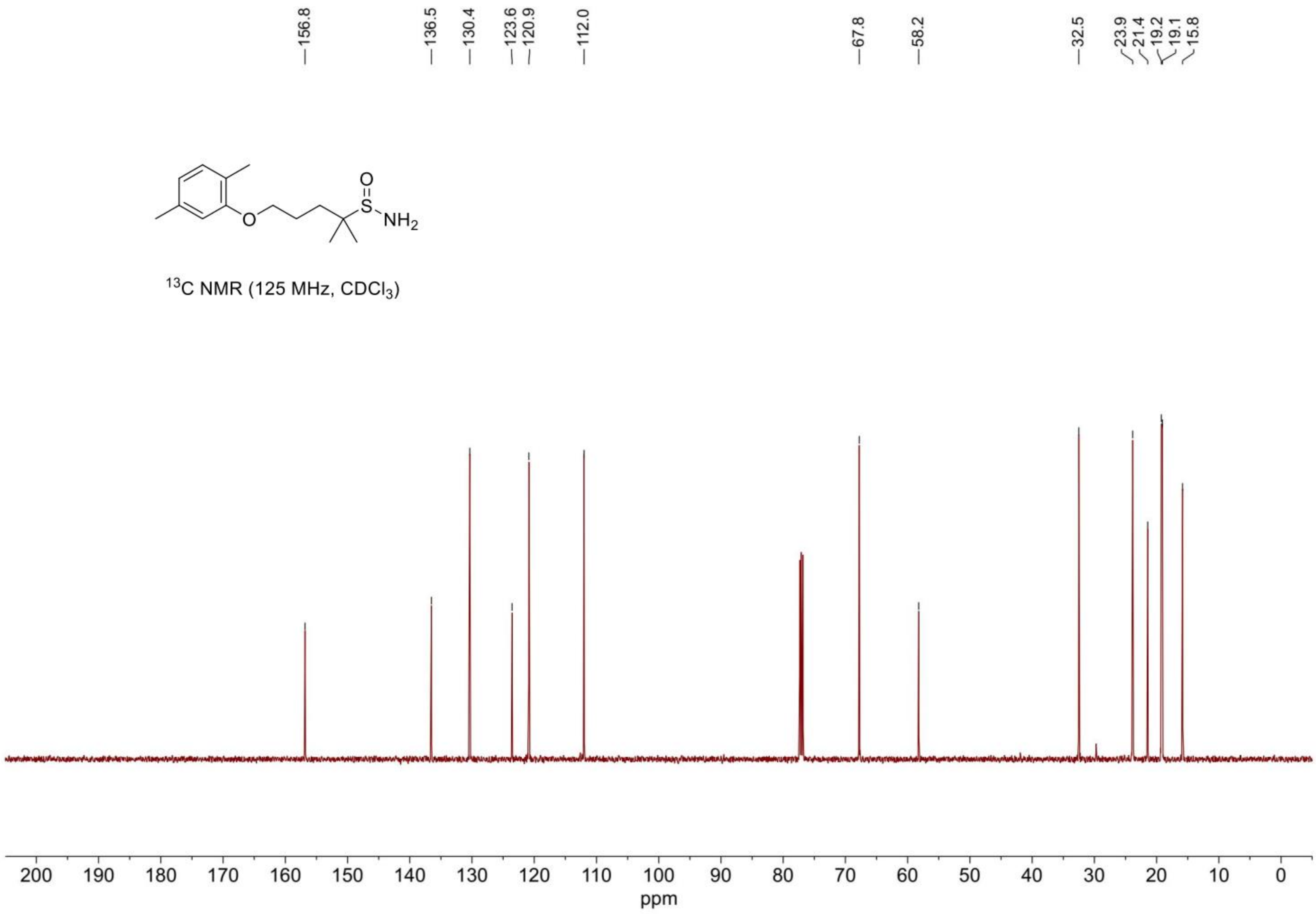

Go to table of contents

5-(2,5-Dimethylphenoxy)-2-methyl-*N*-phenylpentane-2-sulfinamide (7a)

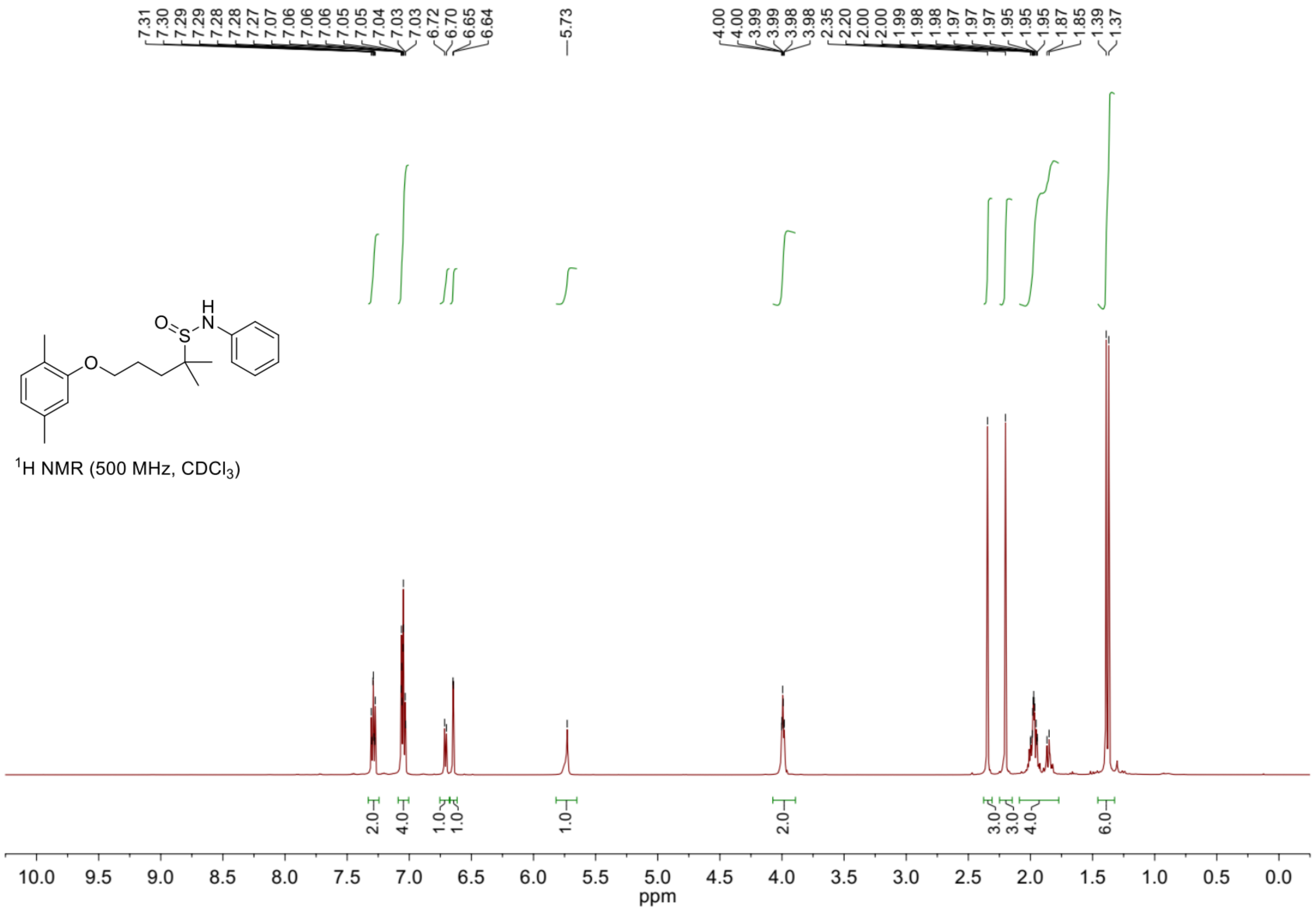

Go to table of contents

5-(2,5-Dimethylphenoxy)-2-methyl-*N*-phenylpentane-2-sulfinamide (7a)

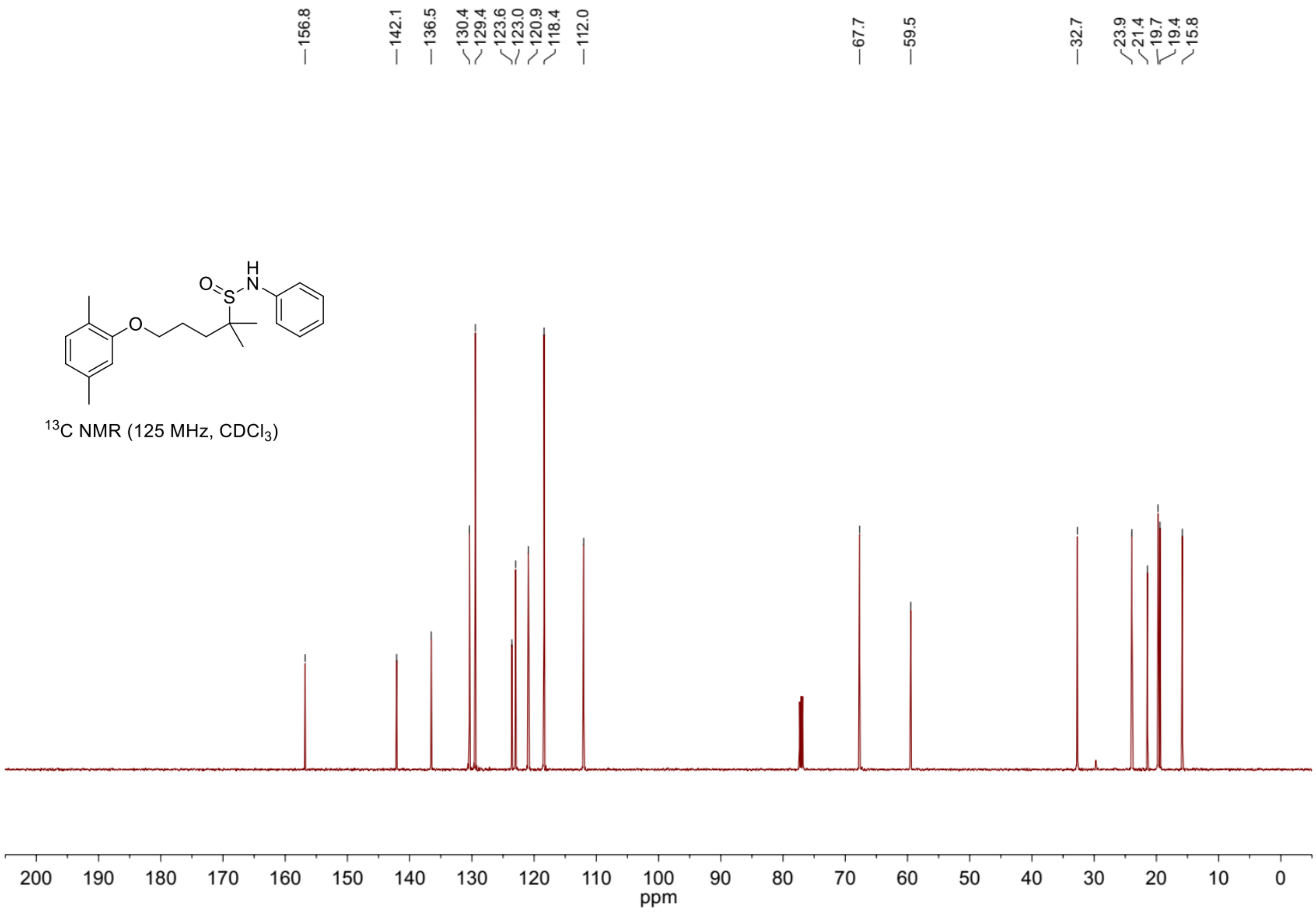

Go to table of contents

(*E*)-5-(4-Hydroxy-6-methoxy-7-methyl-3-oxo-1,3-dihydroisobenzofuran-5-yl)-3-methyl-*N*-phenylpent-3-ene-1-sulfinamide (7b)

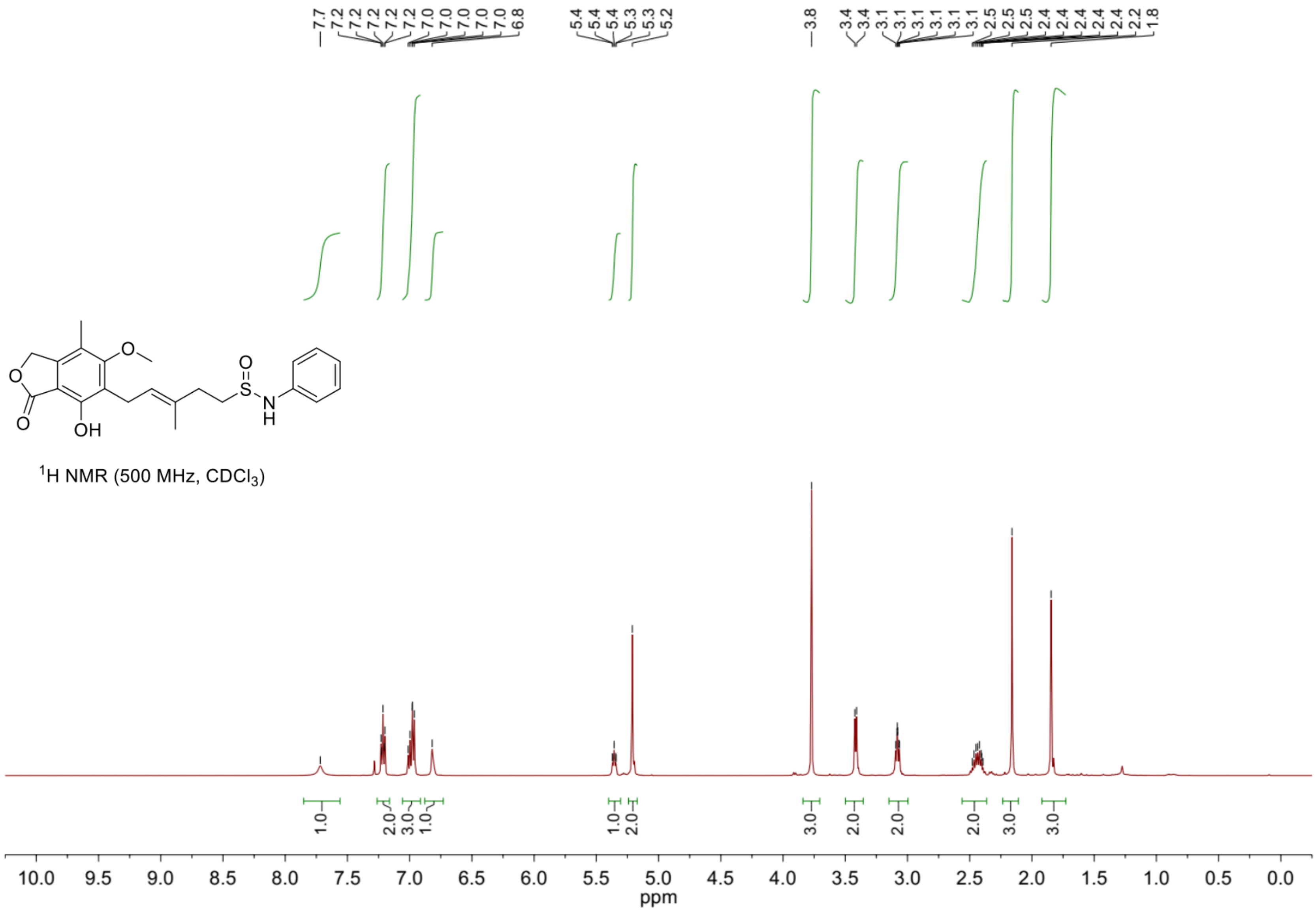

Go to table of contents

(*E*)-5-(4-Hydroxy-6-methoxy-7-methyl-3-oxo-1,3-dihydroisobenzofuran-5-yl)-3-methyl-*N*-phenylpent-3-ene-1-sulfinamide (7b)

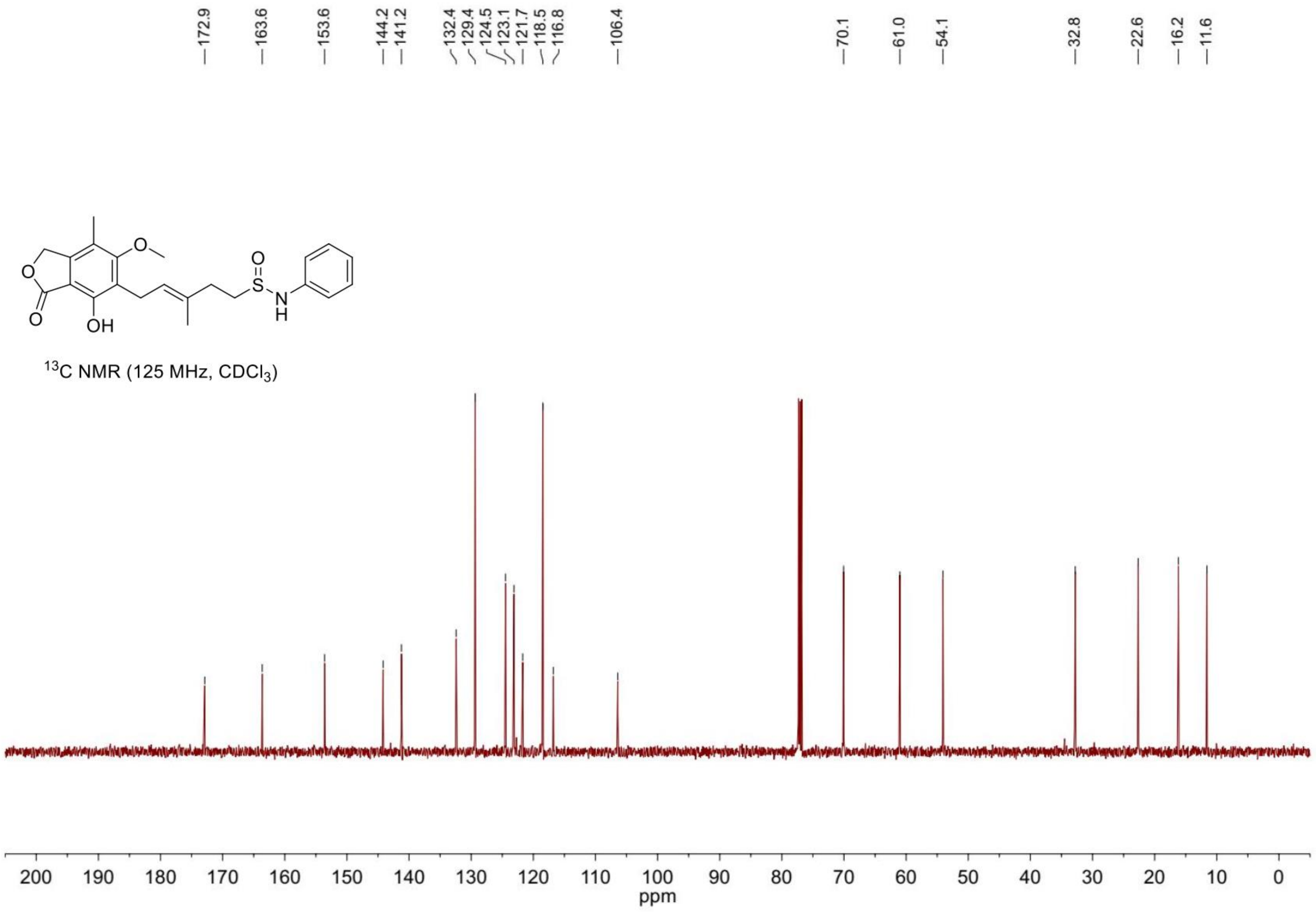

Go to table of contents

Tert-butyl 2-((phenylamino)sulfinyl)pyrrolidine-1-carboxylate (7c)

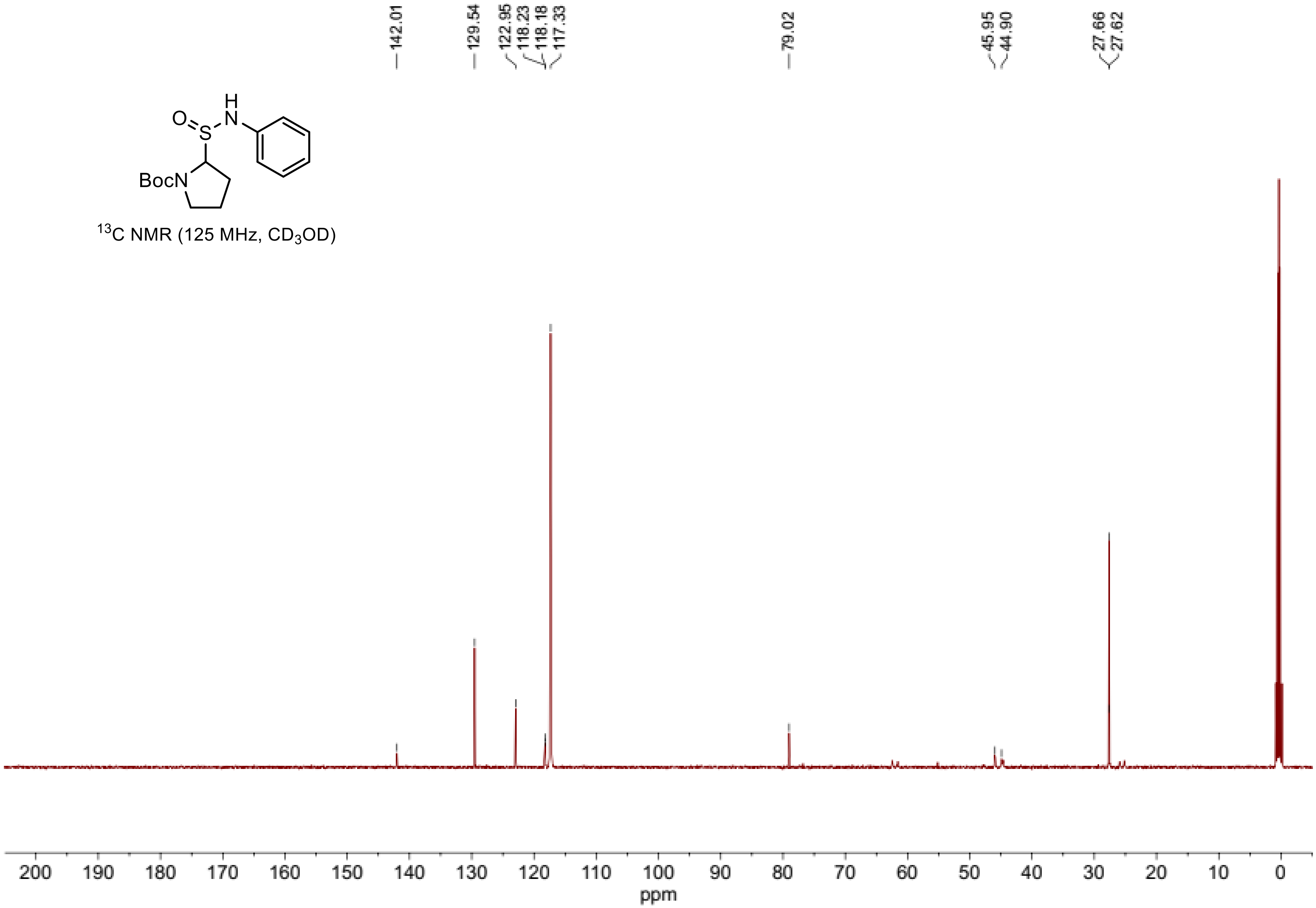

^{13}C NMR (125 MHz, CD_3OD)

Go to table of contents

Tert-butyl 2-((phenylamino)sulfinyl)pyrrolidine-1-carboxylate (7c)

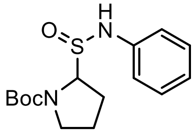

^{1}H NMR (500 MHz, CD_3OD)

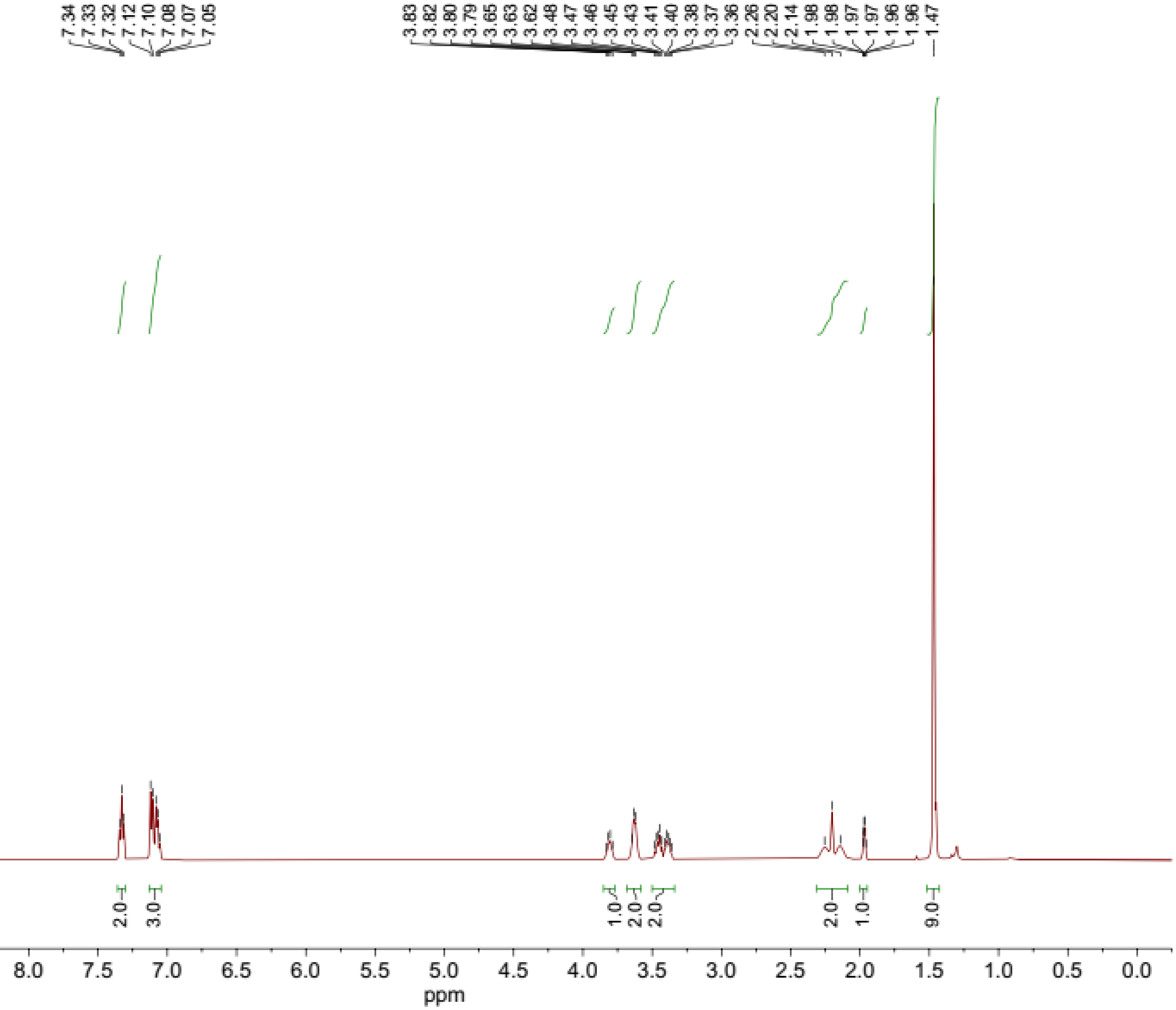

Go to table of contents

Tert-butyl 2-((phenylamino)sulfinyl)pyrrolidine-1-carboxylate (7c)

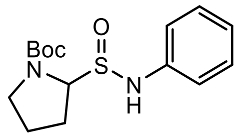

^{1}H NMR (500 MHz, CD_3OD)

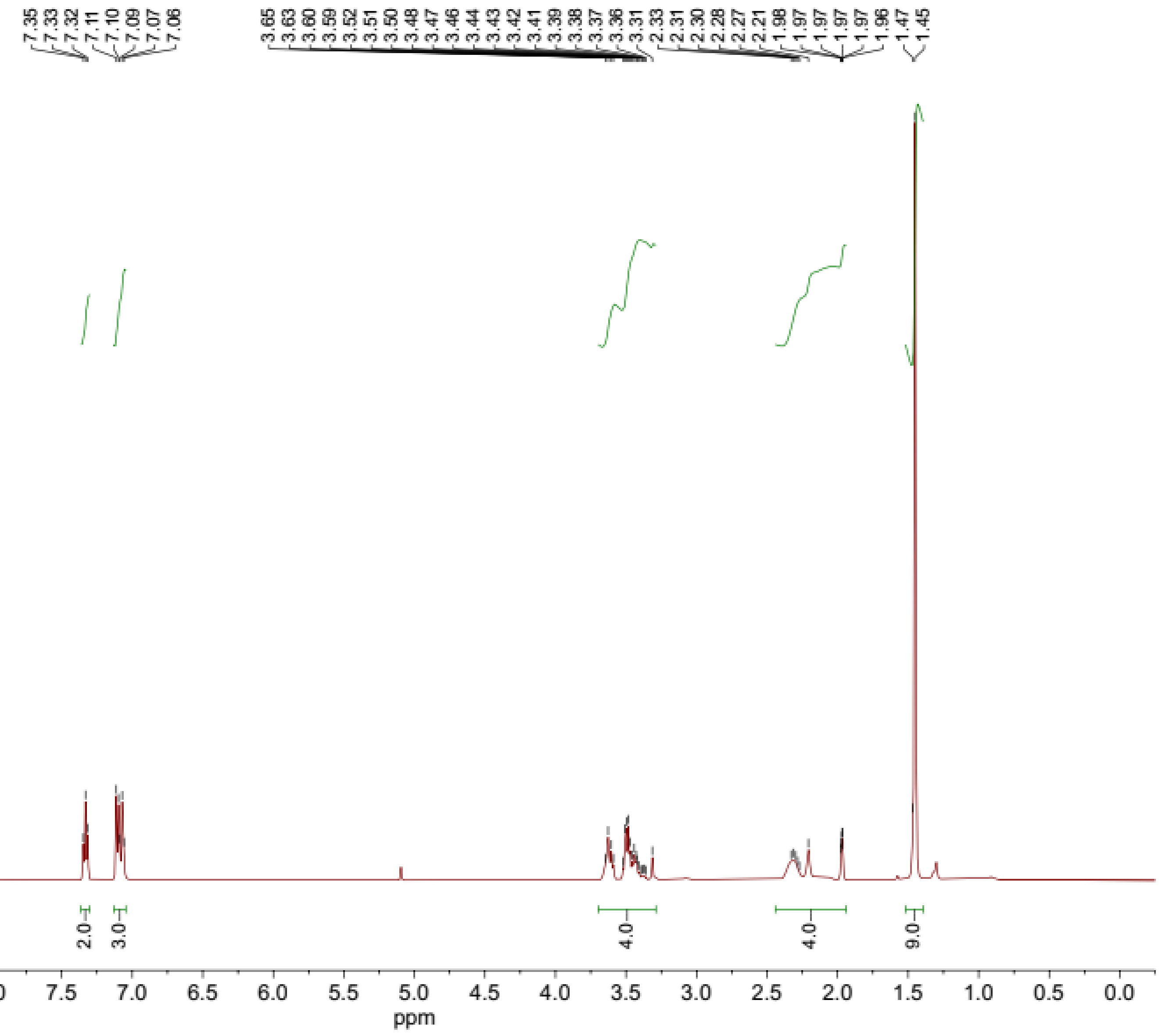

Tert-butyl 2-((phenylamino)sulfinyl)pyrrolidine-1-carboxylate (7c)

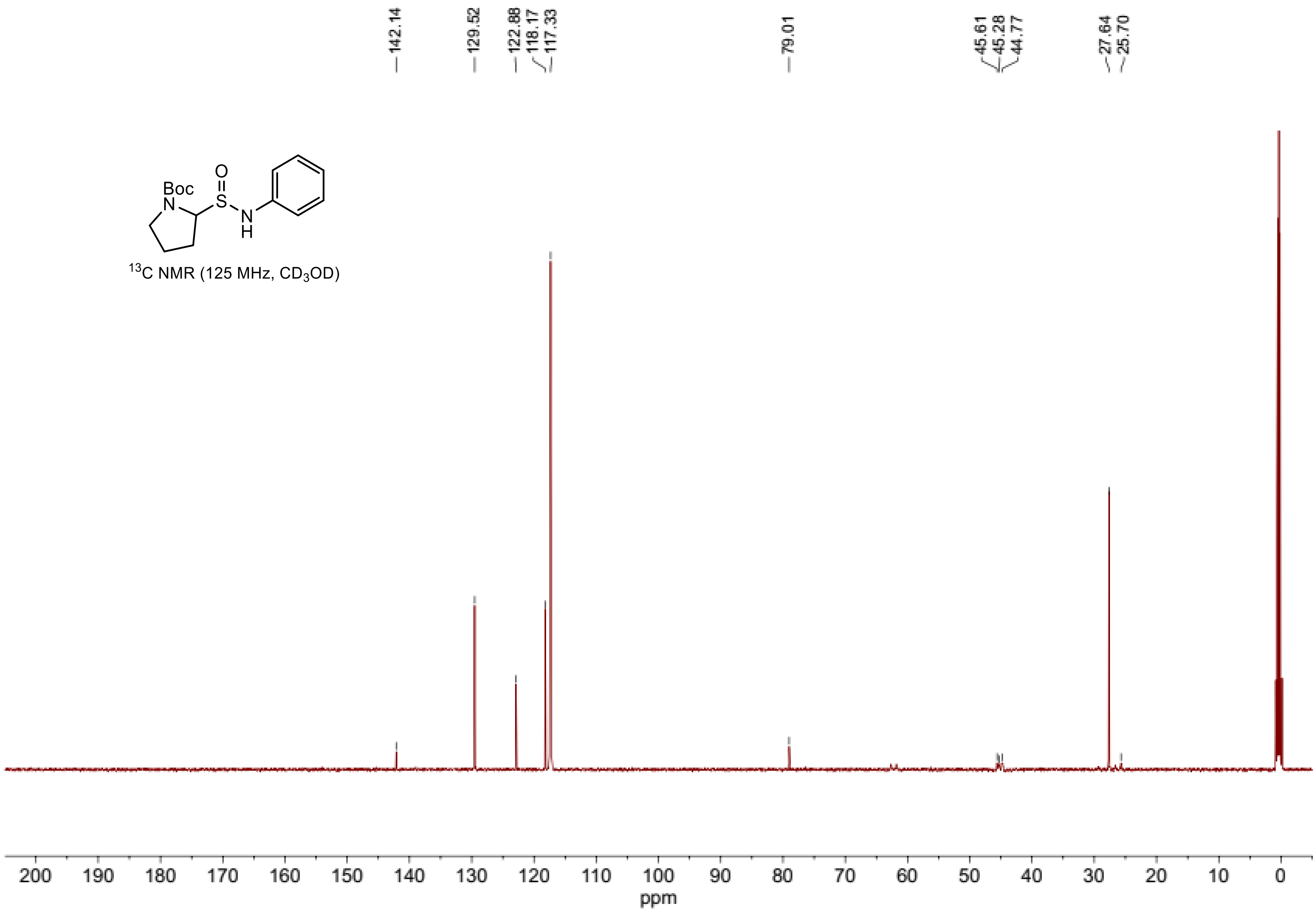

Go to table of contents

tert-Butyl (_tert_-butoxycarbonyl)((phenylamino)sulfinyl)alaninate (7d)

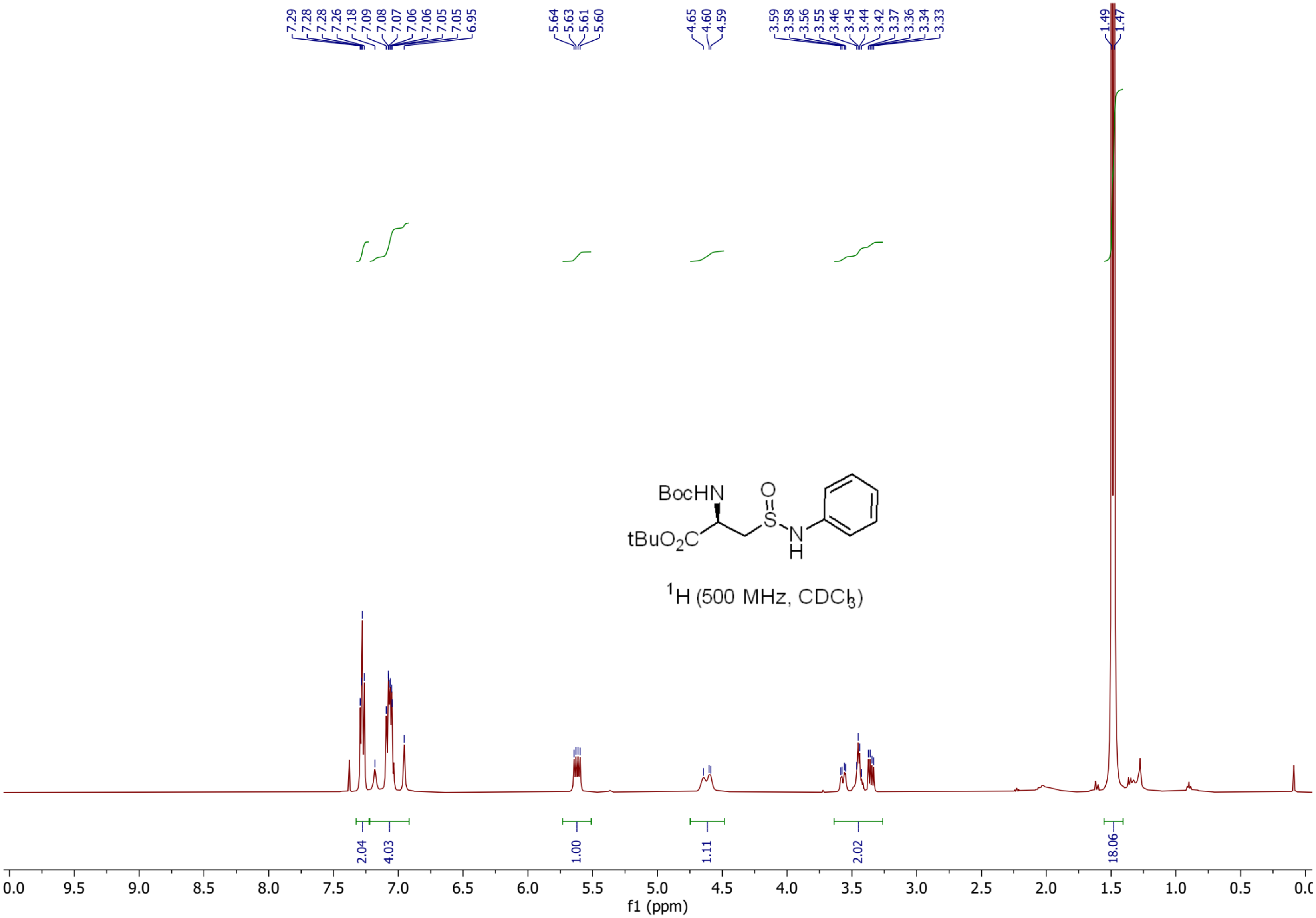

Go to table of contents

tert-Butyl (_tert_-butoxycarbonyl)((phenylamino)sulfinyl)alaninate (7d)

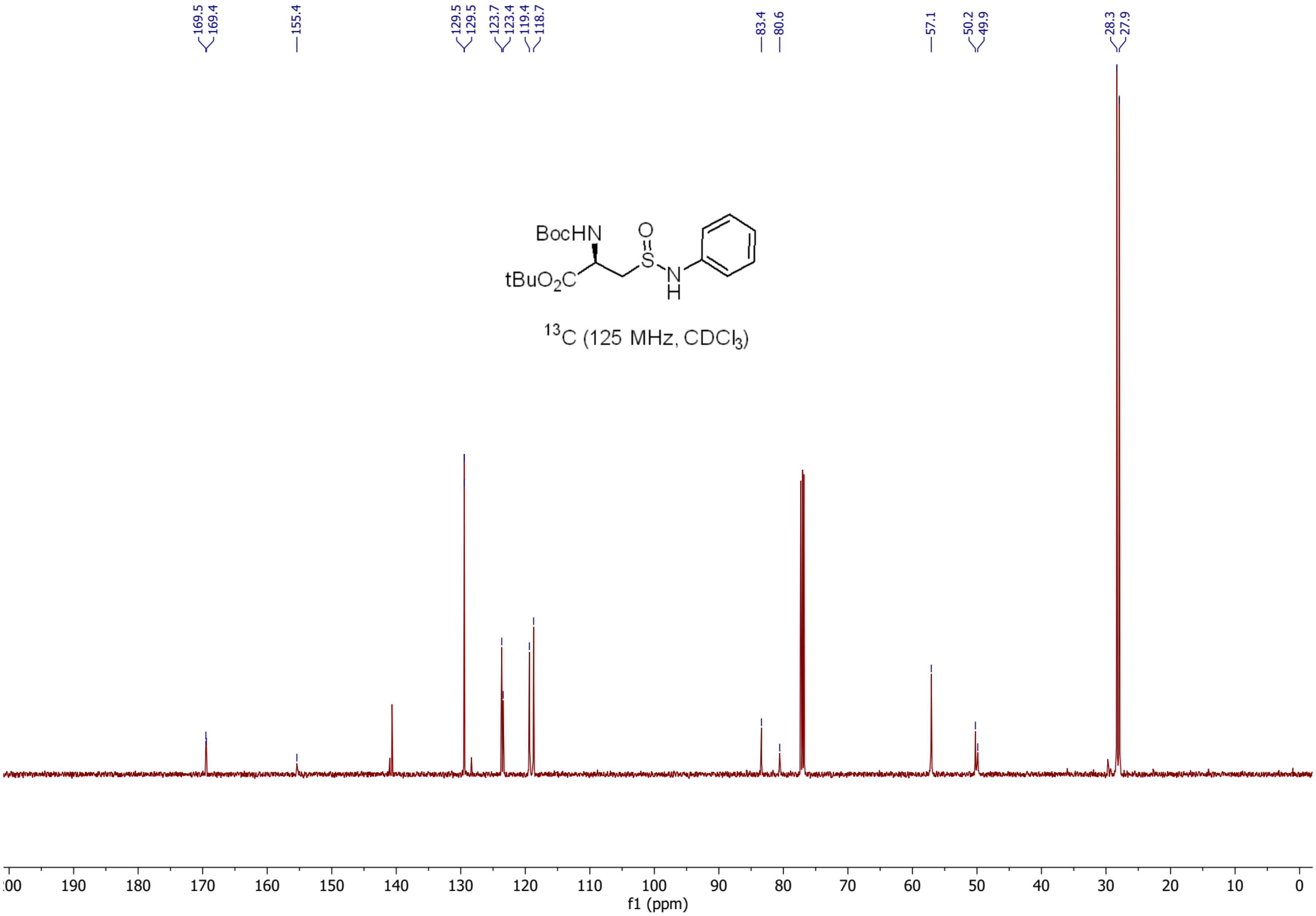

Go to table of contents

2-(4,5-Diphenyloxazol-2-yl)-*N*-phenylethane-1-sulfinamide (7e)

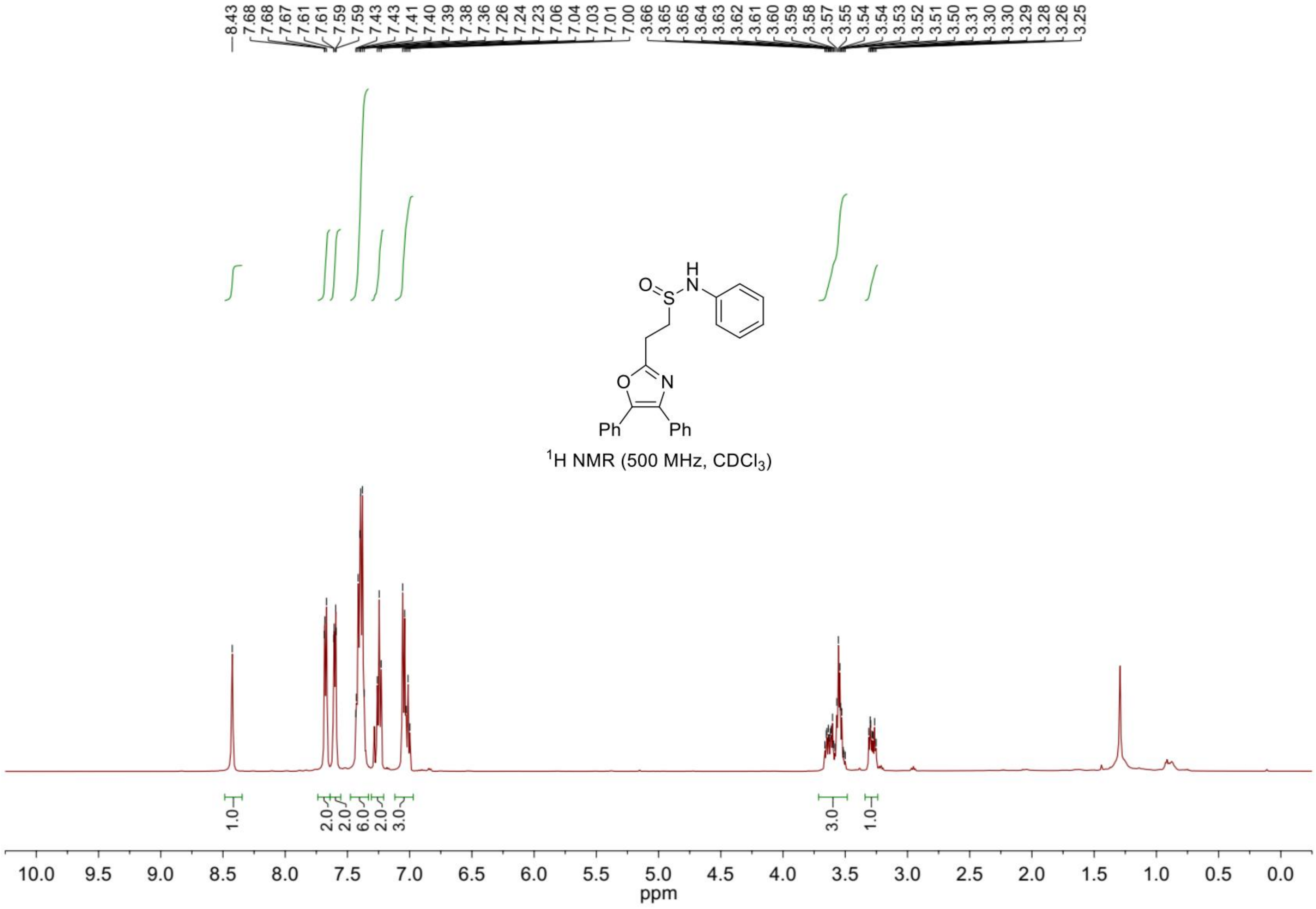

Go to table of contents

2-(4,5-Diphenyloxazol-2-yl)-*N*-phenylethane-1-sulfinamide (7e)

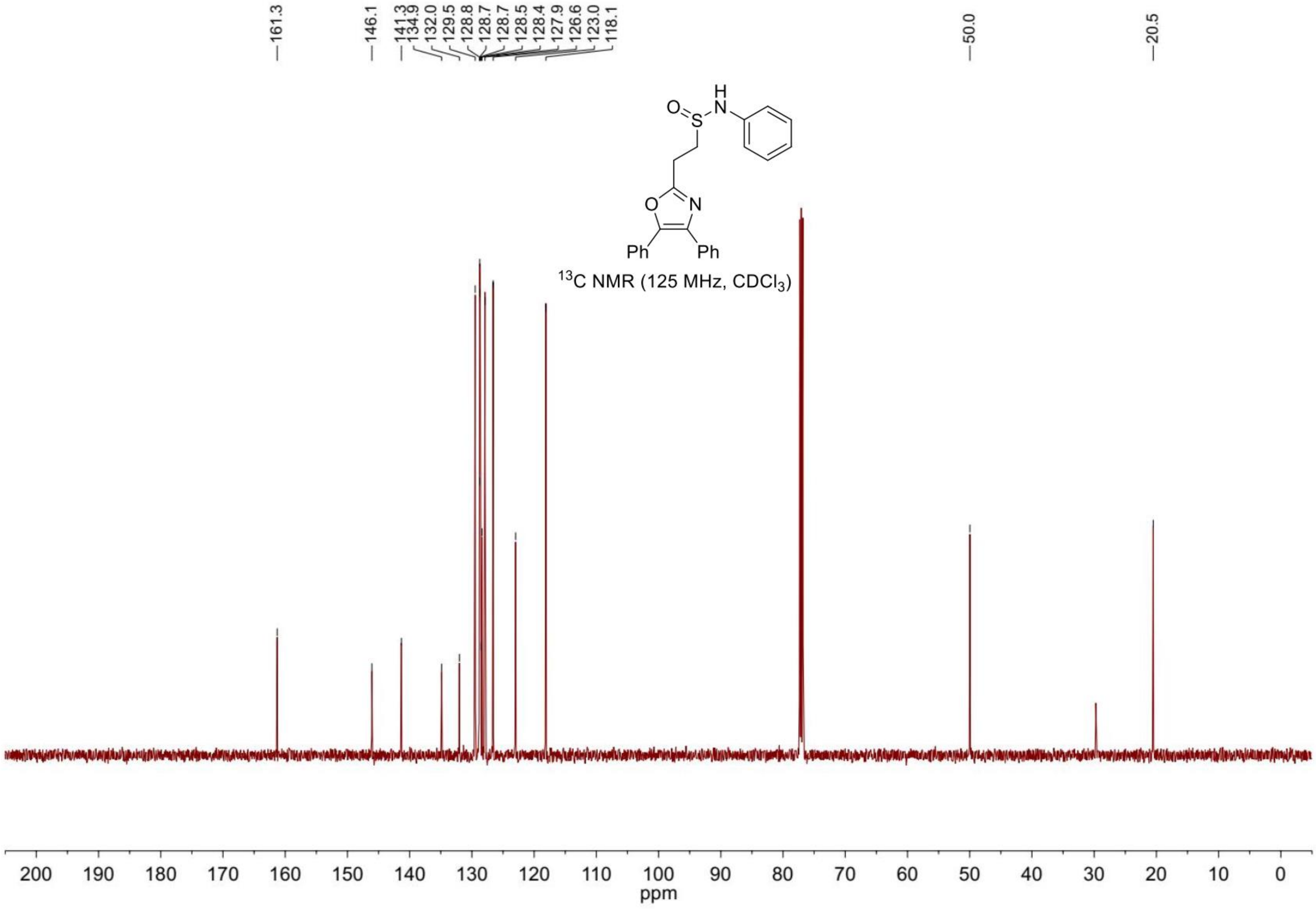

Go to table of contents

1-(11-Oxo-6,11-dihydrodibenzo[*b*,*e*]oxepin-2-yl)-*N*-phenylmethanesulfinamide (7f)

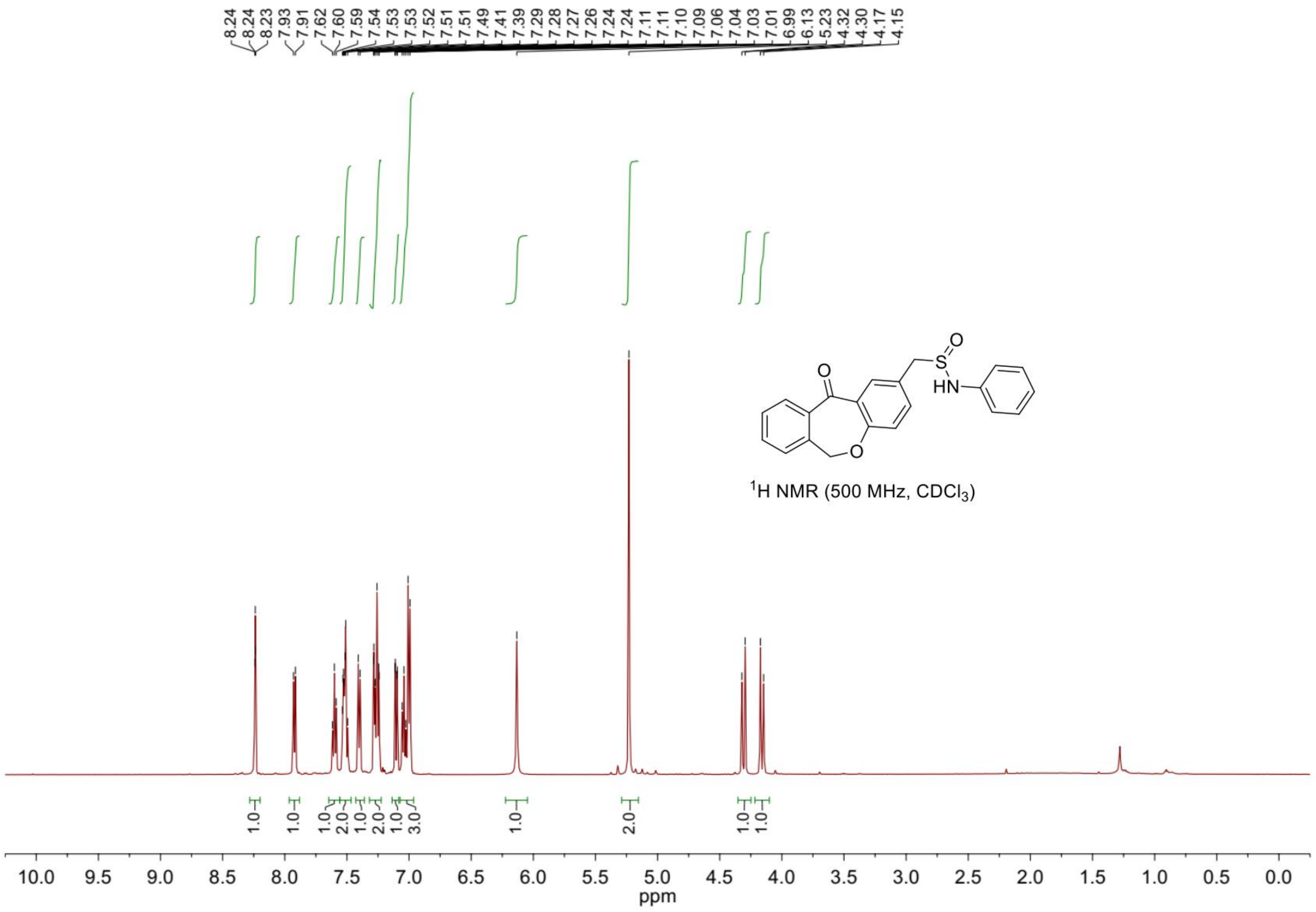

Go to table of contents

1-(11-Oxo-6,11-dihydrodibenzo[*b*,*e*]oxepin-2-yl)-*N*-phenylmethanesulfinamide (7f)

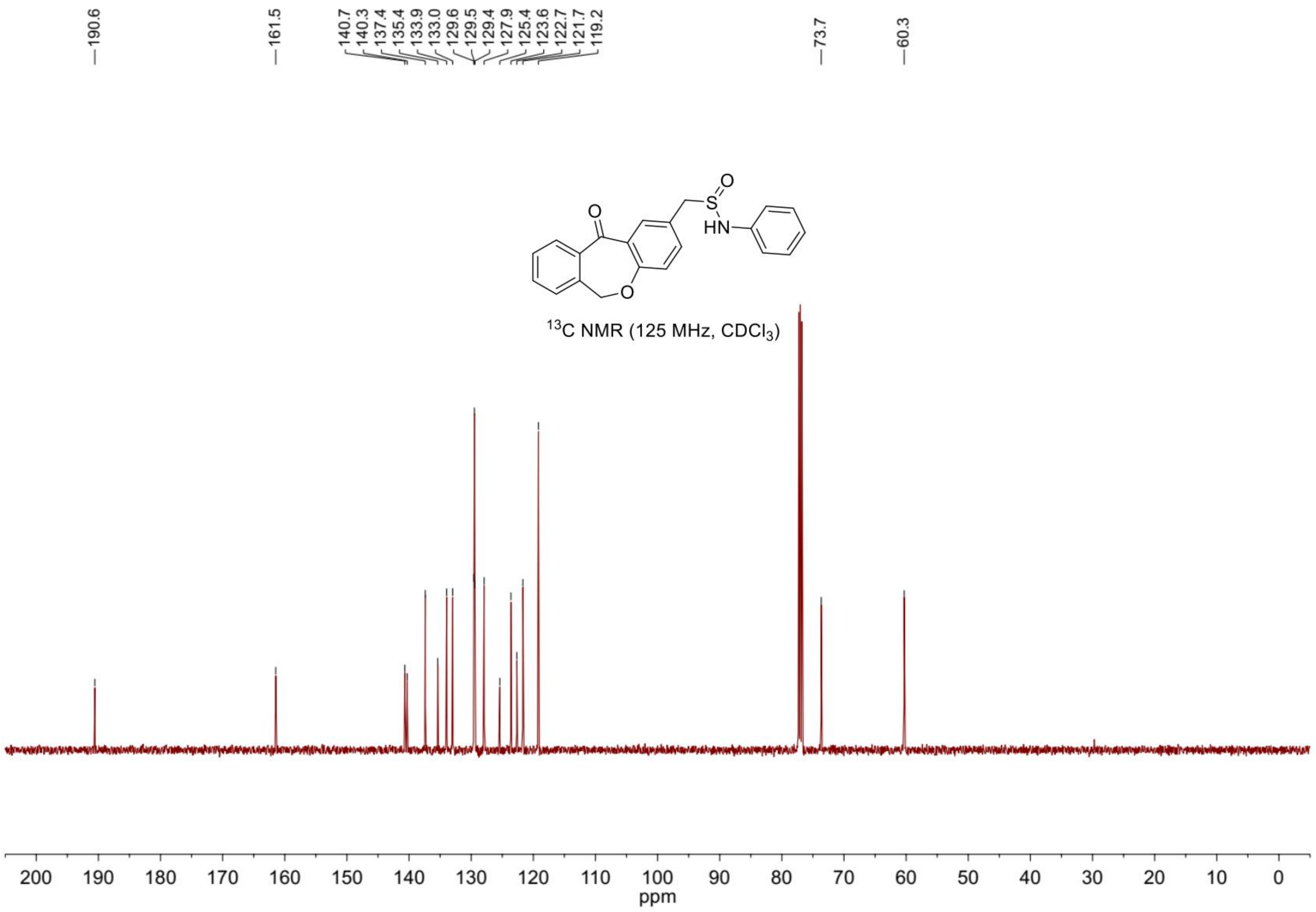

Go to table of contents

(*Z*)-*N*-Phenylhenicos-12-ene-1-sulfinamide (7g)

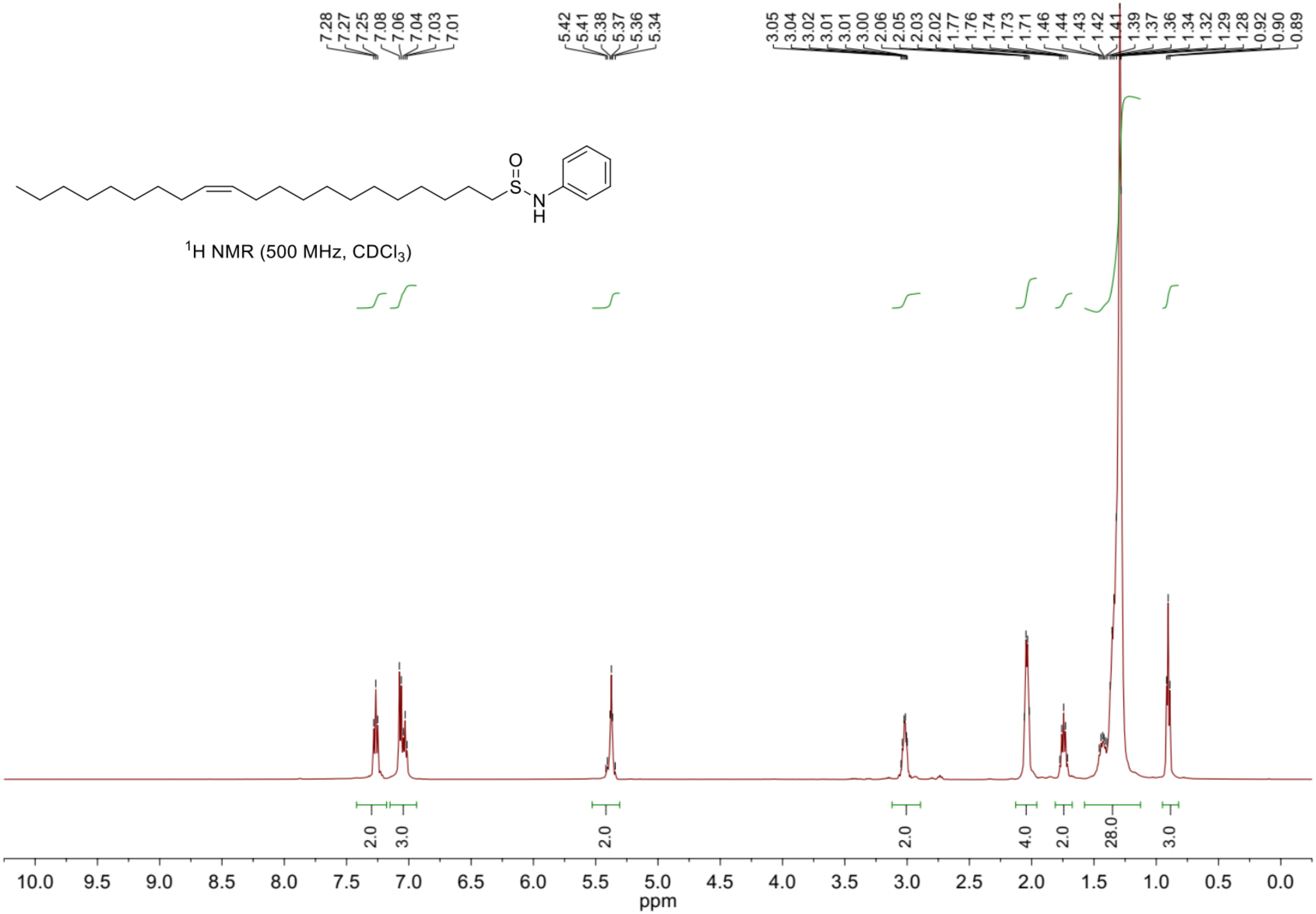

Go to table of contents

(*Z*)-*N*-Phenylhenicos-12-ene-1-sulfinamide (7g)

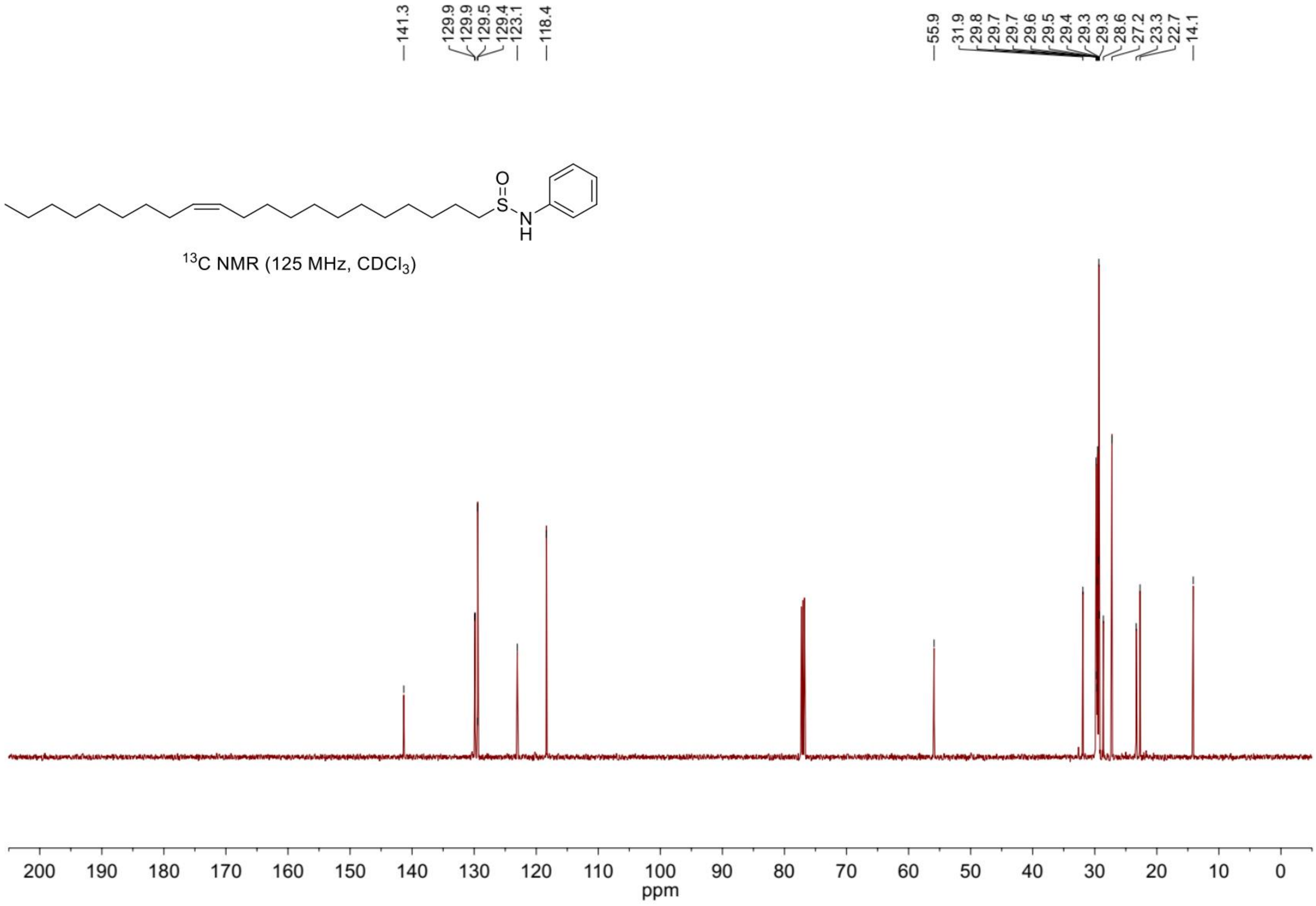

Go to table of contents

(8*Z*,11*Z*)-*N*-Phenylheptadeca-8,11-diene-1-sulfinamide (7h)

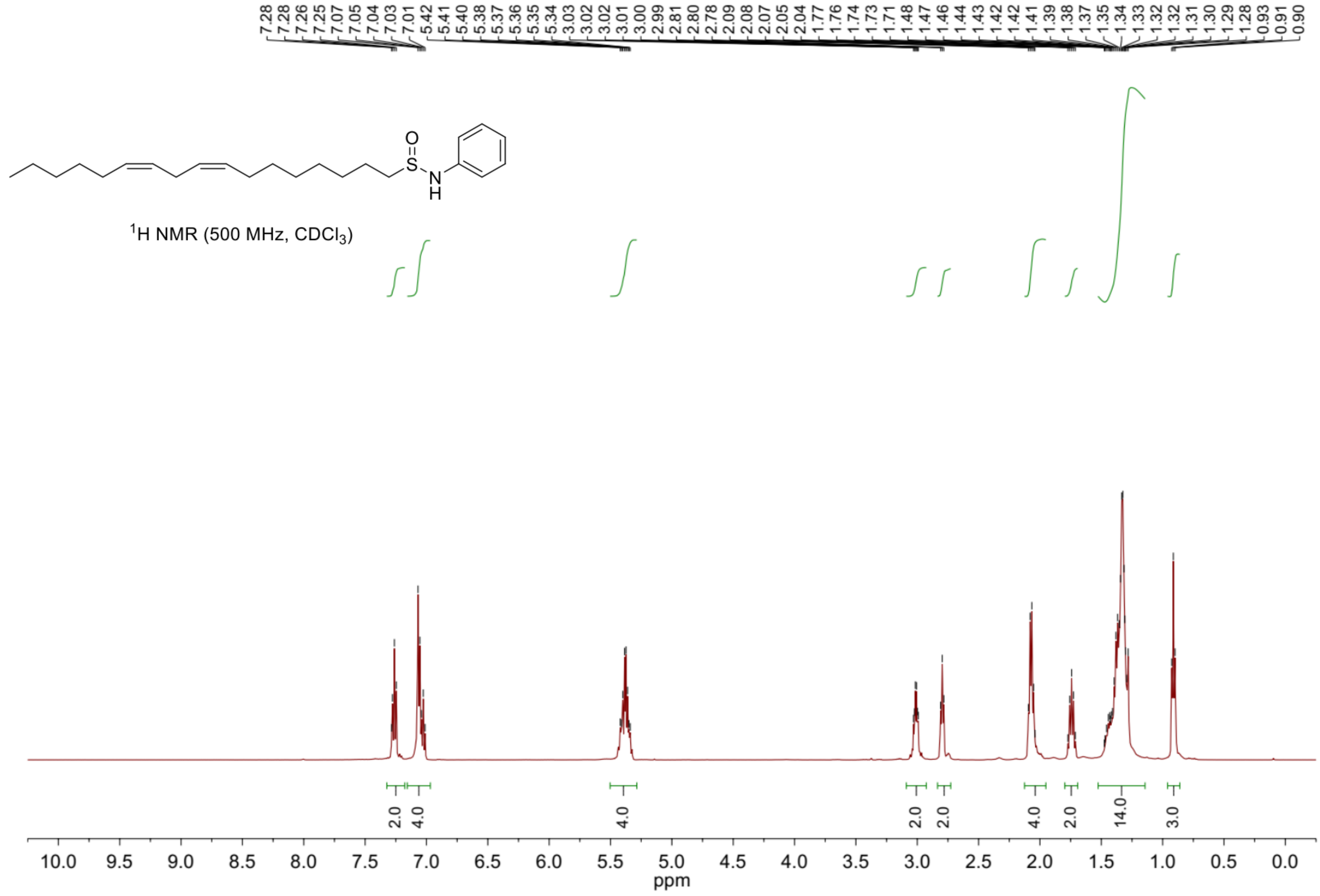

Go to table of contents

(8*Z*,11*Z*)-*N*-Phenylheptadeca-8,11-diene-1-sulfinamide (7h)

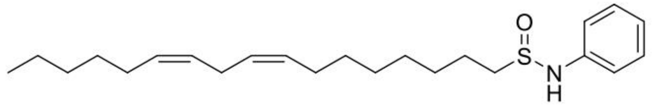

^{13}C NMR (125 MHz, $CDCl_3$)

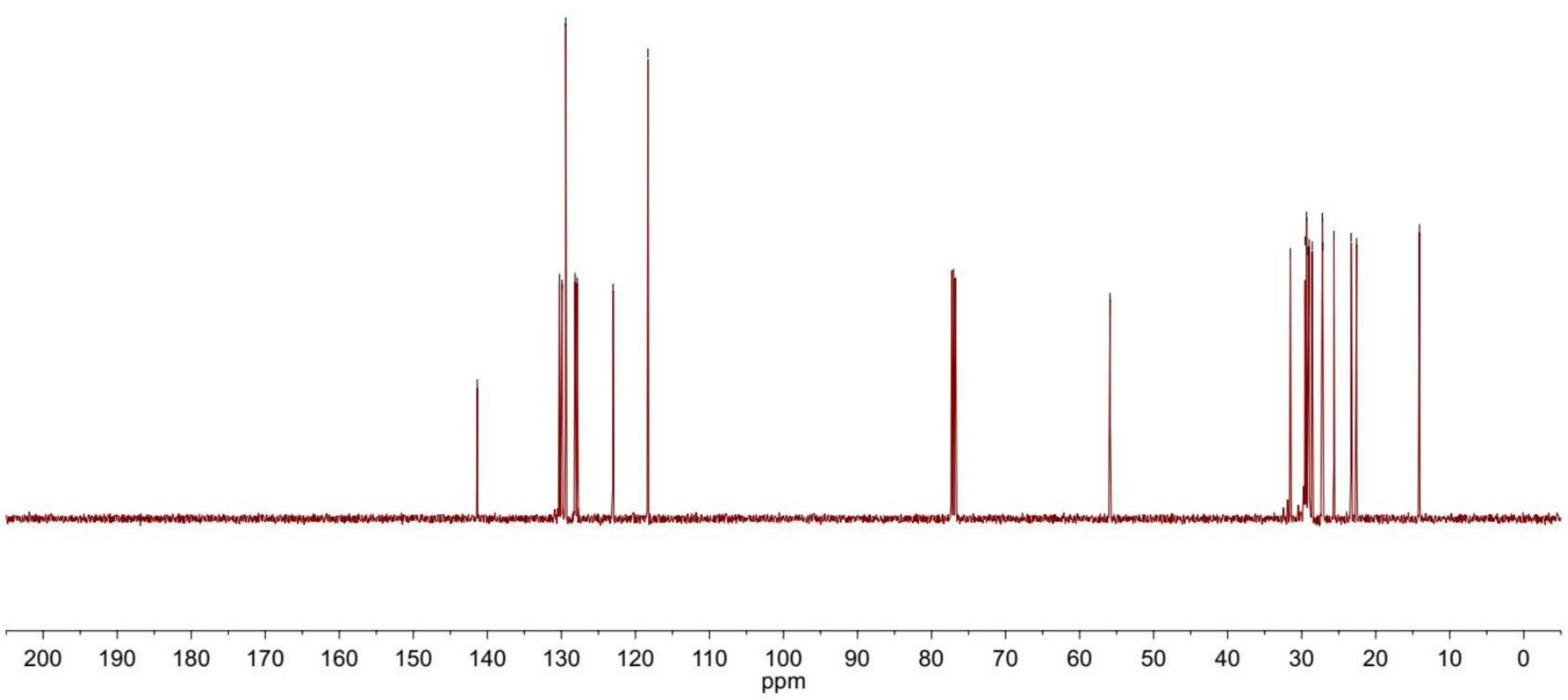

Go to table of contents

N-Phenyltetracosa-9,11-diyne-1-sulfinamide (7i)

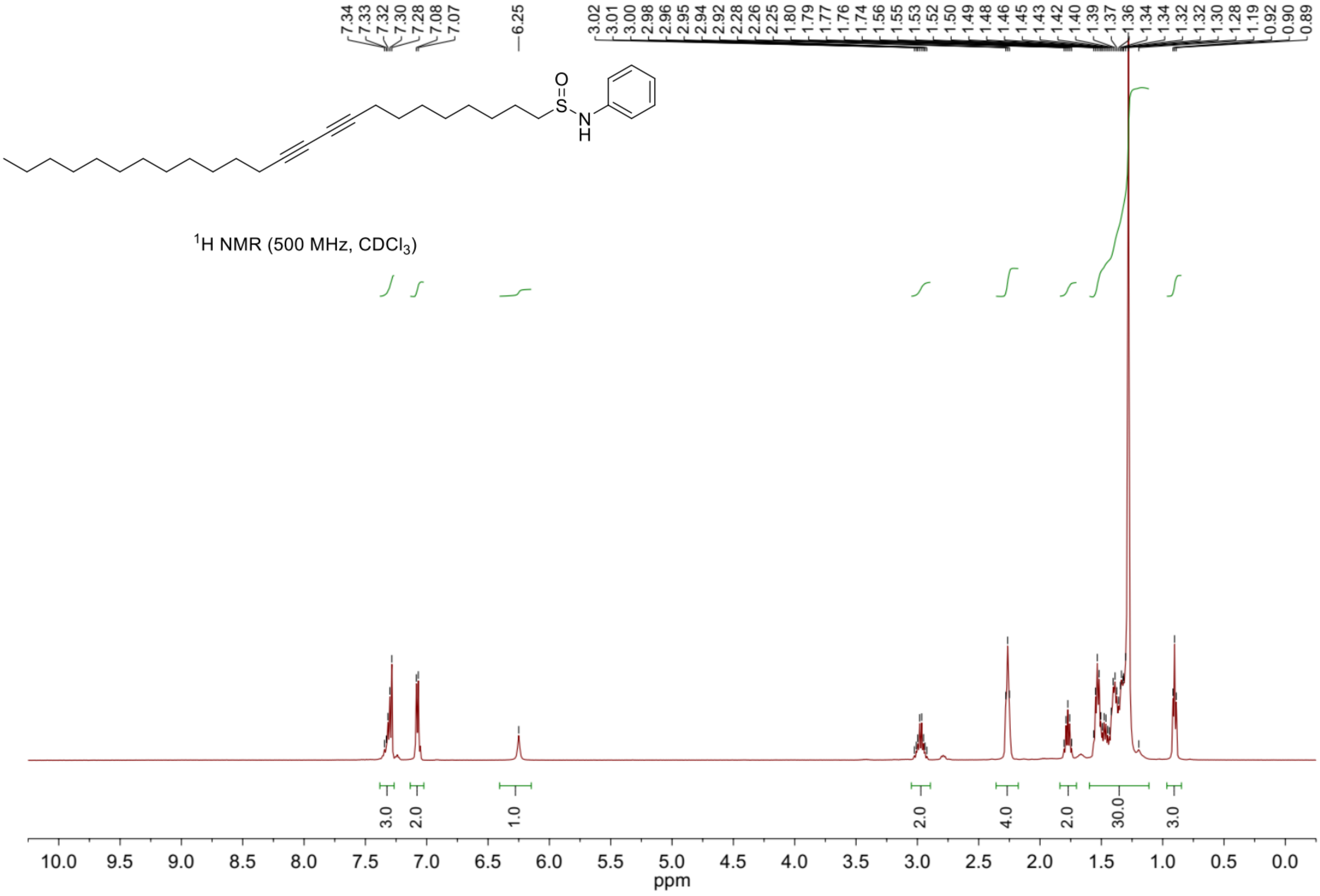

Go to table of contents

N-Phenyltetracosa-9,11-diyne-1-sulfinamide (7i)

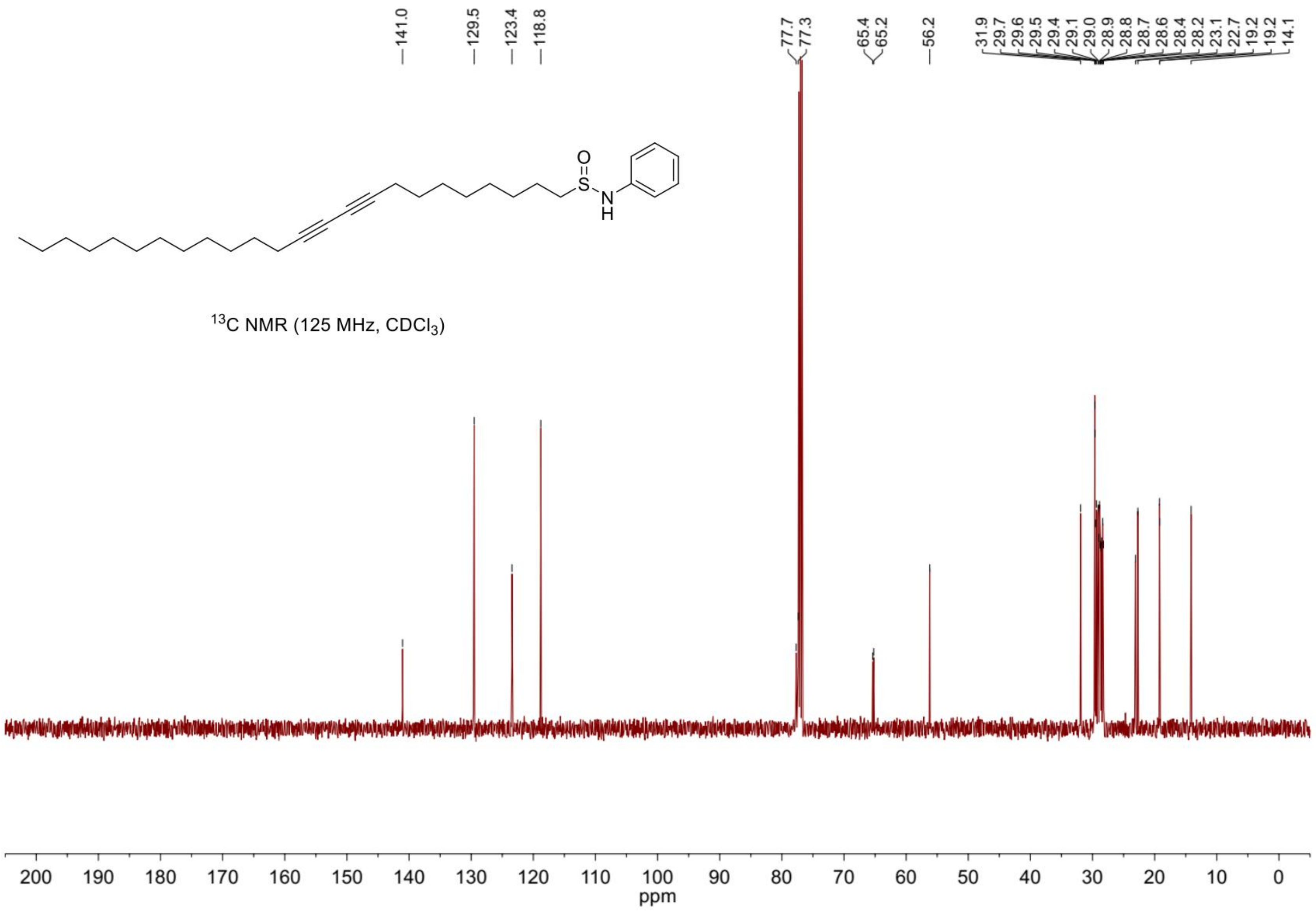

Go to table of contents

1-((1*S*,3*R*)-3-Acetyl-2,2-dimethylcyclobutyl)-*N*-phenylmethanesulfinamide (7j)

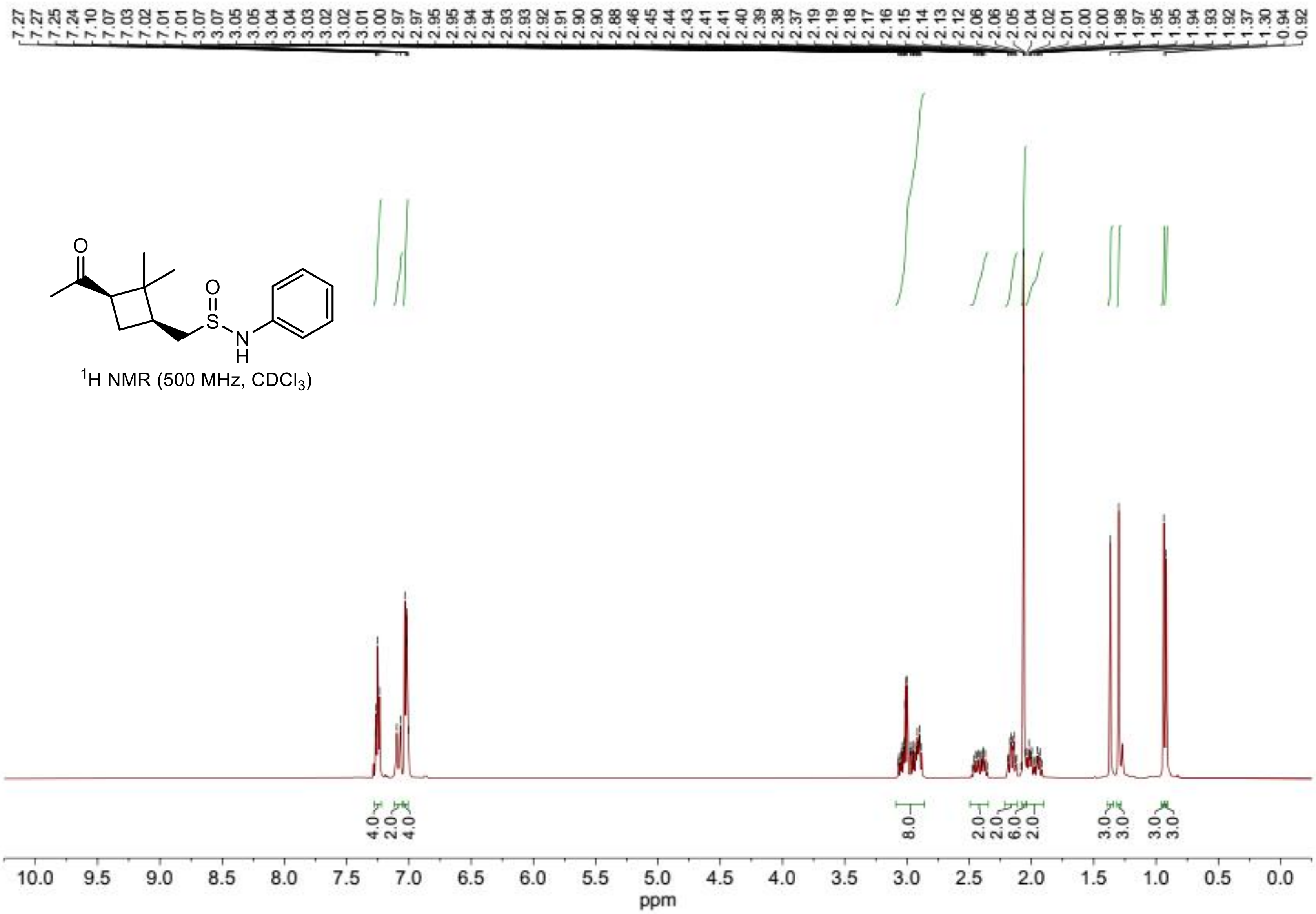

Go to table of contents

1-((1*S*,3*R*)-3-Acetyl-2,2-dimethylcyclobutyl)-*N*-phenylmethanesulfinamide (7j)

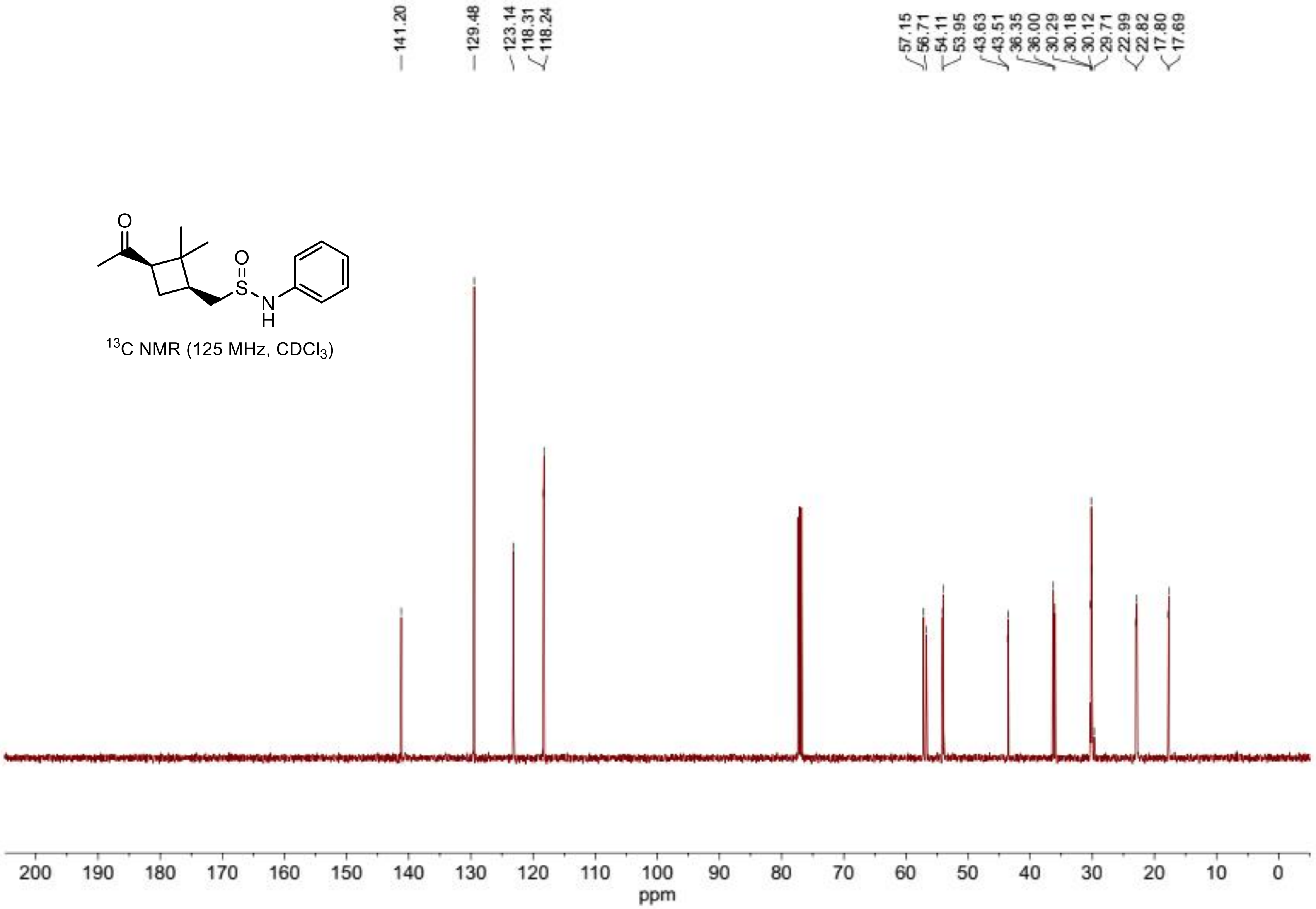

Go to table of contents

(6aS,6bR,8a*R*,10*S*,12a*R*,12b*R*,14b*S*)-10-hydroxy-2,2,6a,6b,9,9,12a-heptamethyl-*N*-phenyl-1,3,4,5,6,6a,6b,7,8,8a,9,10,11,12,12a,12b,13,14b-octadecahydropicene-4a(2*H*)-sulfinamide (7k)

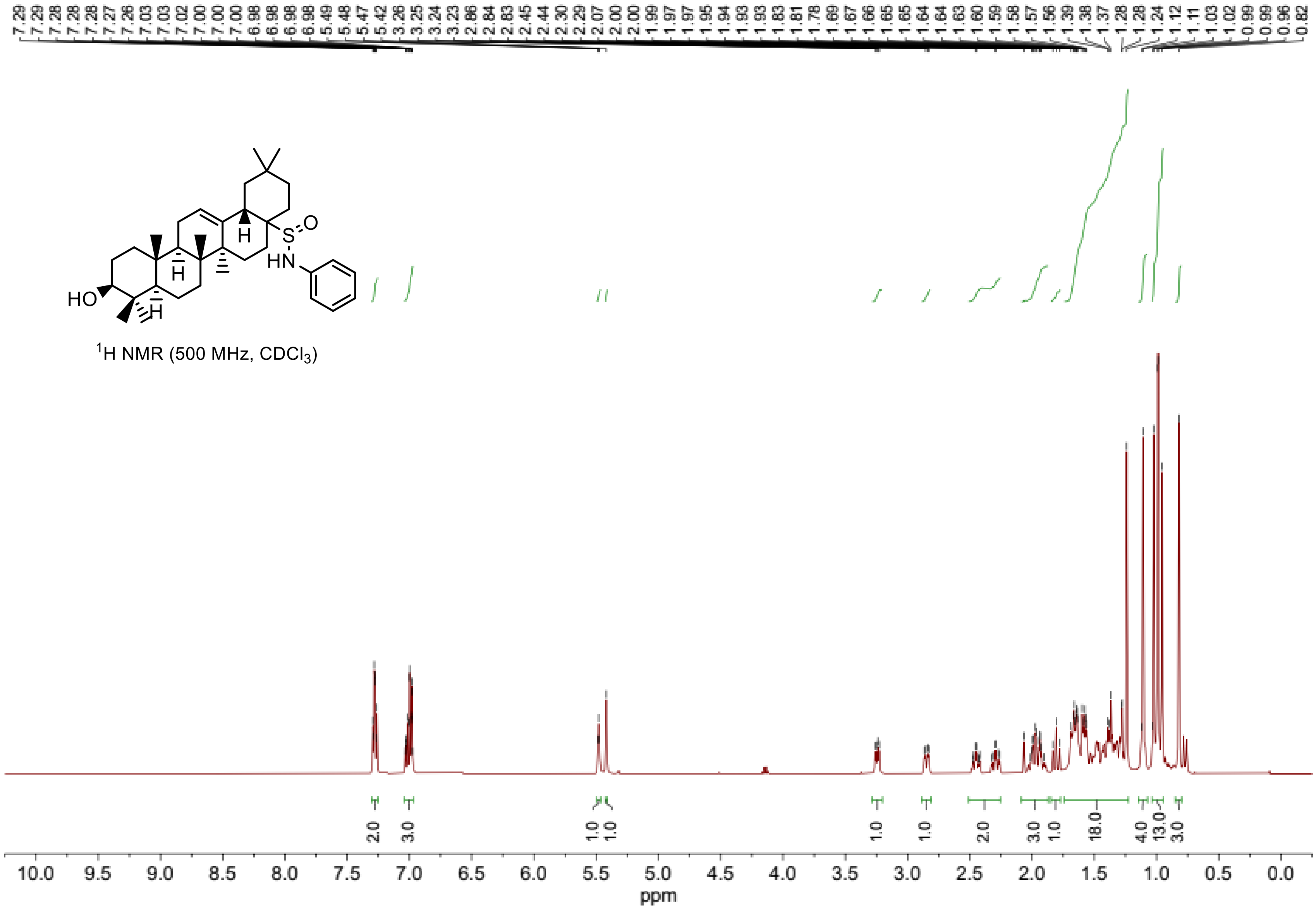

Go to table of contents

(6*aS*,6b*R*,8a*R*,10*S*,12a*R*,12b*R*,14b*S*)-10-hydroxy-2,2,6a,6b,9,9,12a-heptamethyl-*N*-phenyl-1,3,4,5,6,6a,6b,7,8,8a,9,10,11,12,12a,12b,13,14b-octadecahydropicene-4a(2*H*)-sulfinamide (7k)

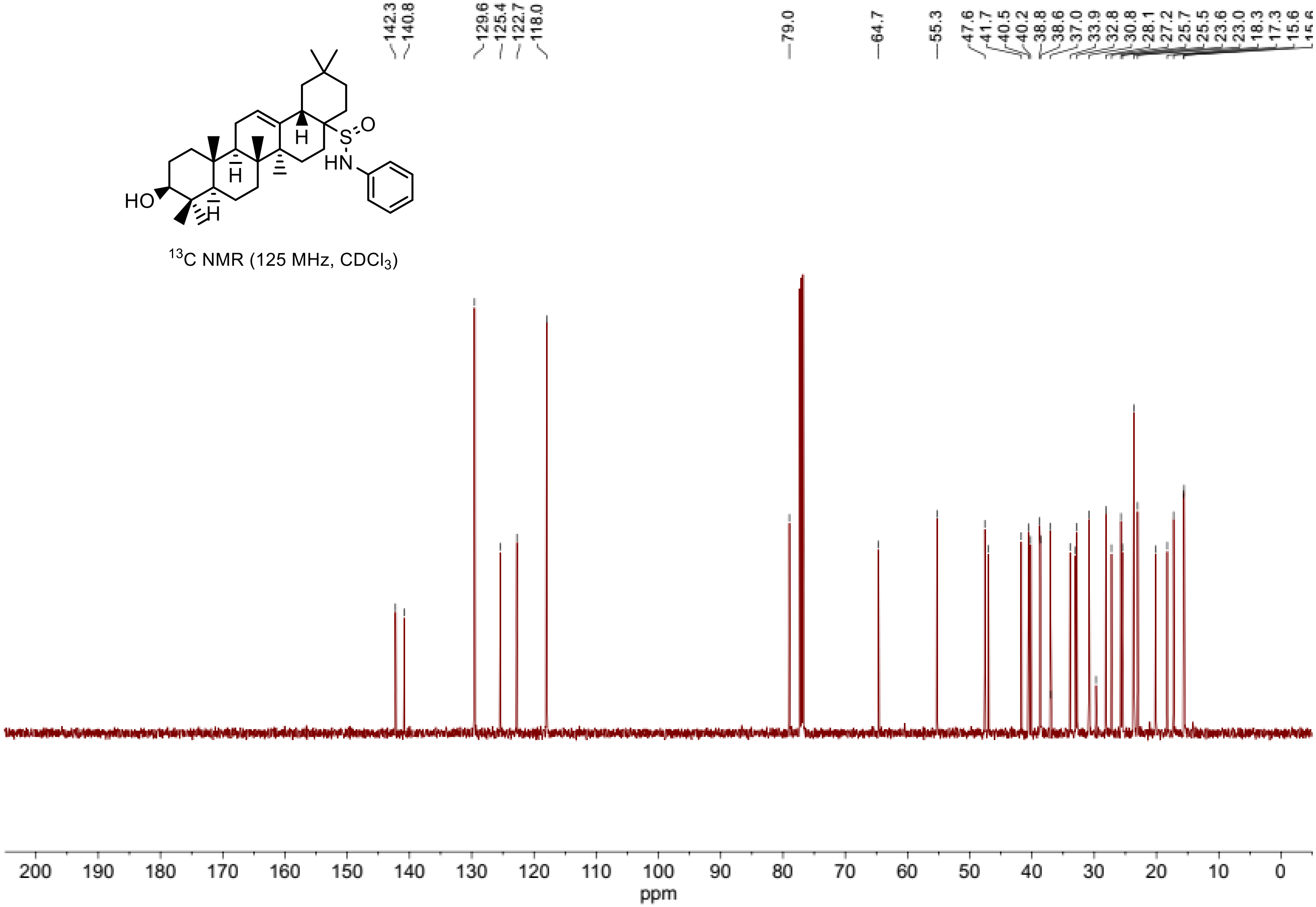

^{13}C NMR (125 MHz, $CDCl_3$)

Go to table of contents

(3*S*,6a*R*,6b*S*,8a*S*,11*S*,12a*S*,14a*R*,14b*S*)-4,4,6a,6b,8a,11,14b-Heptamethyl-14-oxo-11-((phenylamino)sulfinyl)-1,2,3,4,4a,5,6,6a,6b,7,8,8a,9,10,11,12,12a,14,14a,14b-icosahydropicen-3-yl acetate (7l)

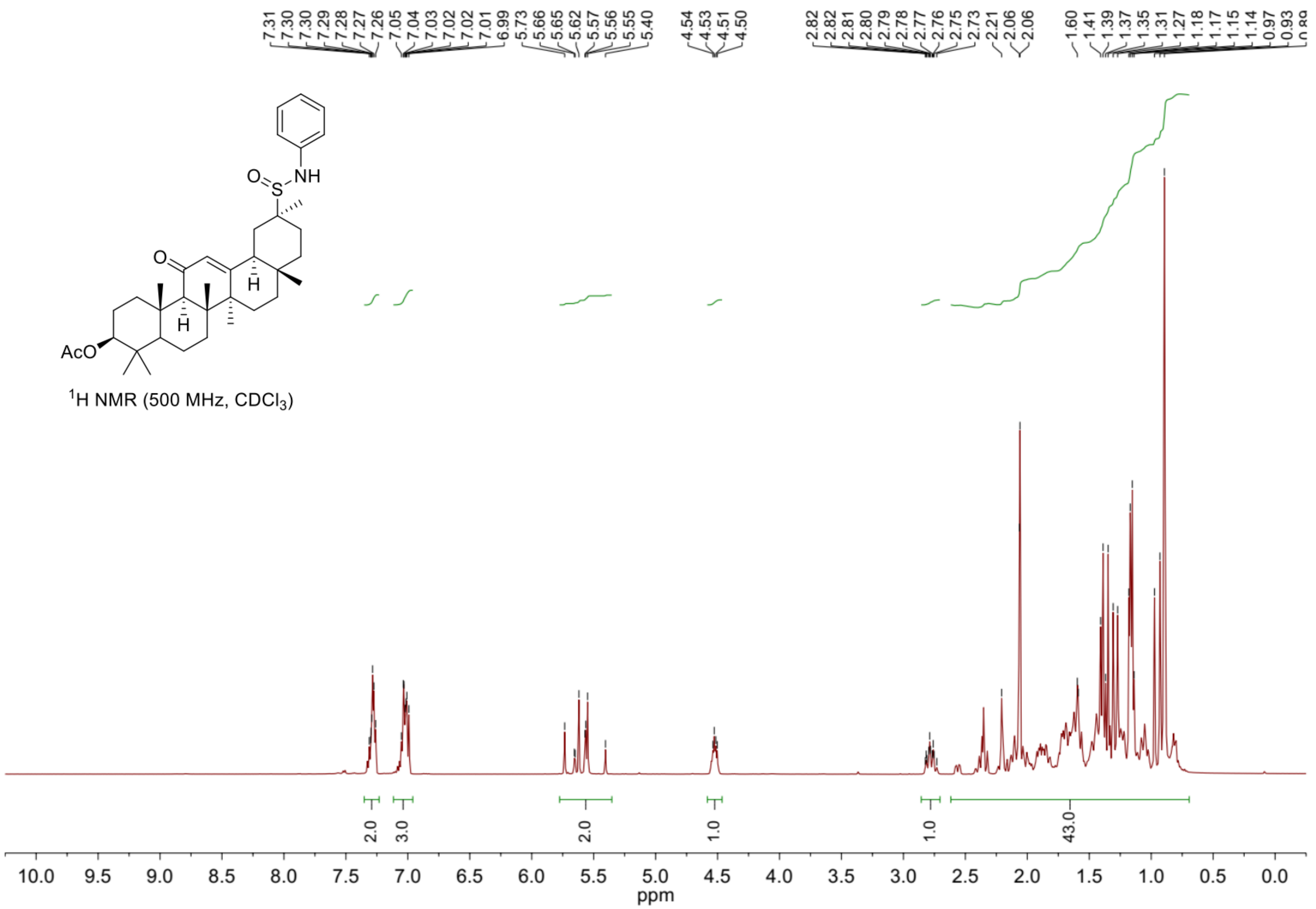

Go to table of contents

(3*S*,6a*R*,6b*S*,8a*S*,11*S*,12a*S*,14a*R*,14b*S*)-4,4,6a,6b,8a,11,14b-Heptamethyl-14-oxo-11-((phenylamino)sulfinyl)-1,2,3,4,4a,5,6,6a,6b,7,8,8a,9,10,11,12,12a,14,14a,14b-icosahydropicen-3-yl acetate (7l)

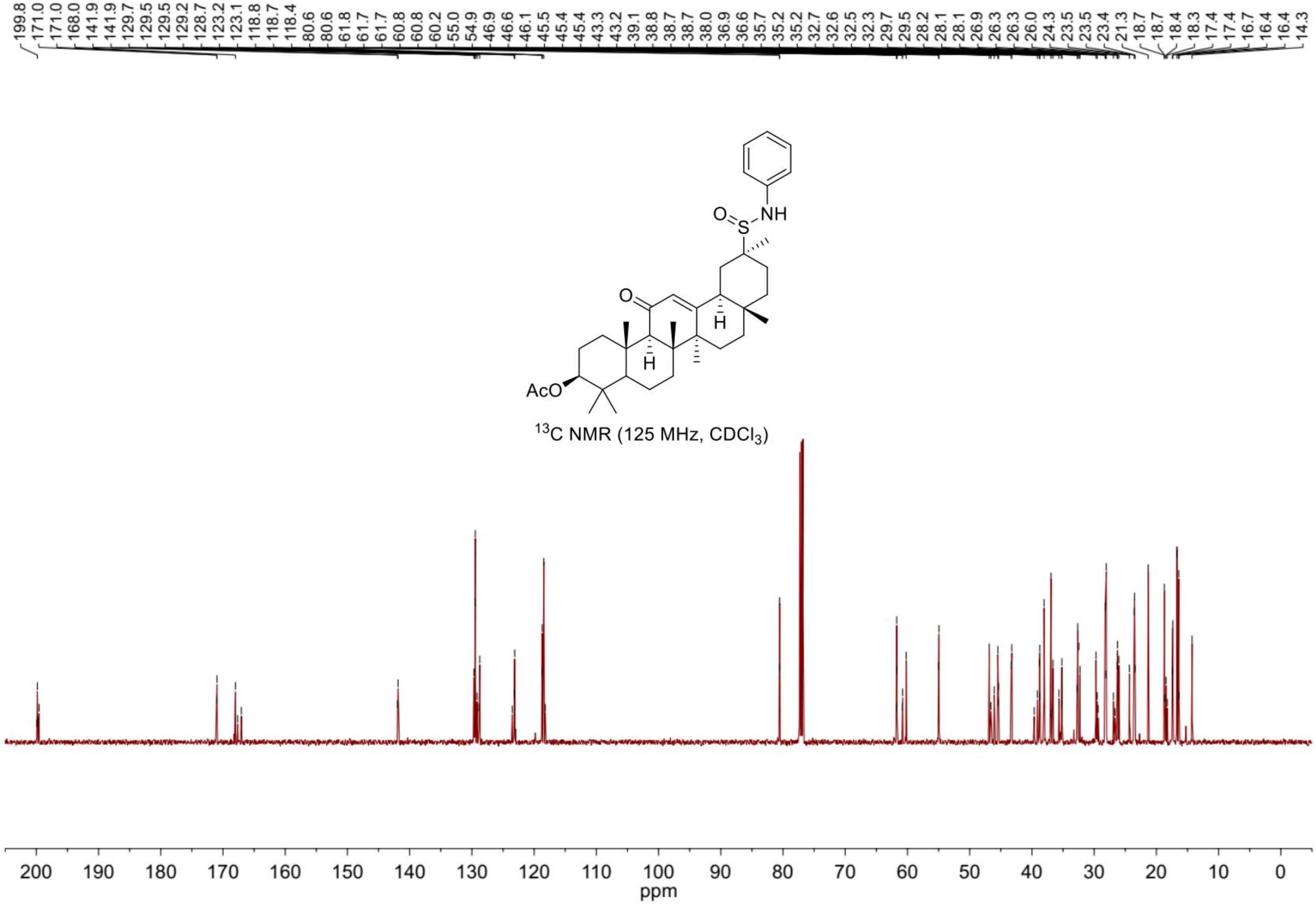

Go to table of contents

(3*R*)-3-((3*R*,7*R*,8*R*,9*S*,10*S*,13*R*,14*S*,17*R*)-3,7-Dihydroxy-10,13-dimethylhexadecahydro-1*H*-cyclopenta[a]phenanthren-17-yl)-*N*-phenylbutane-1-sulfinamide (7m)

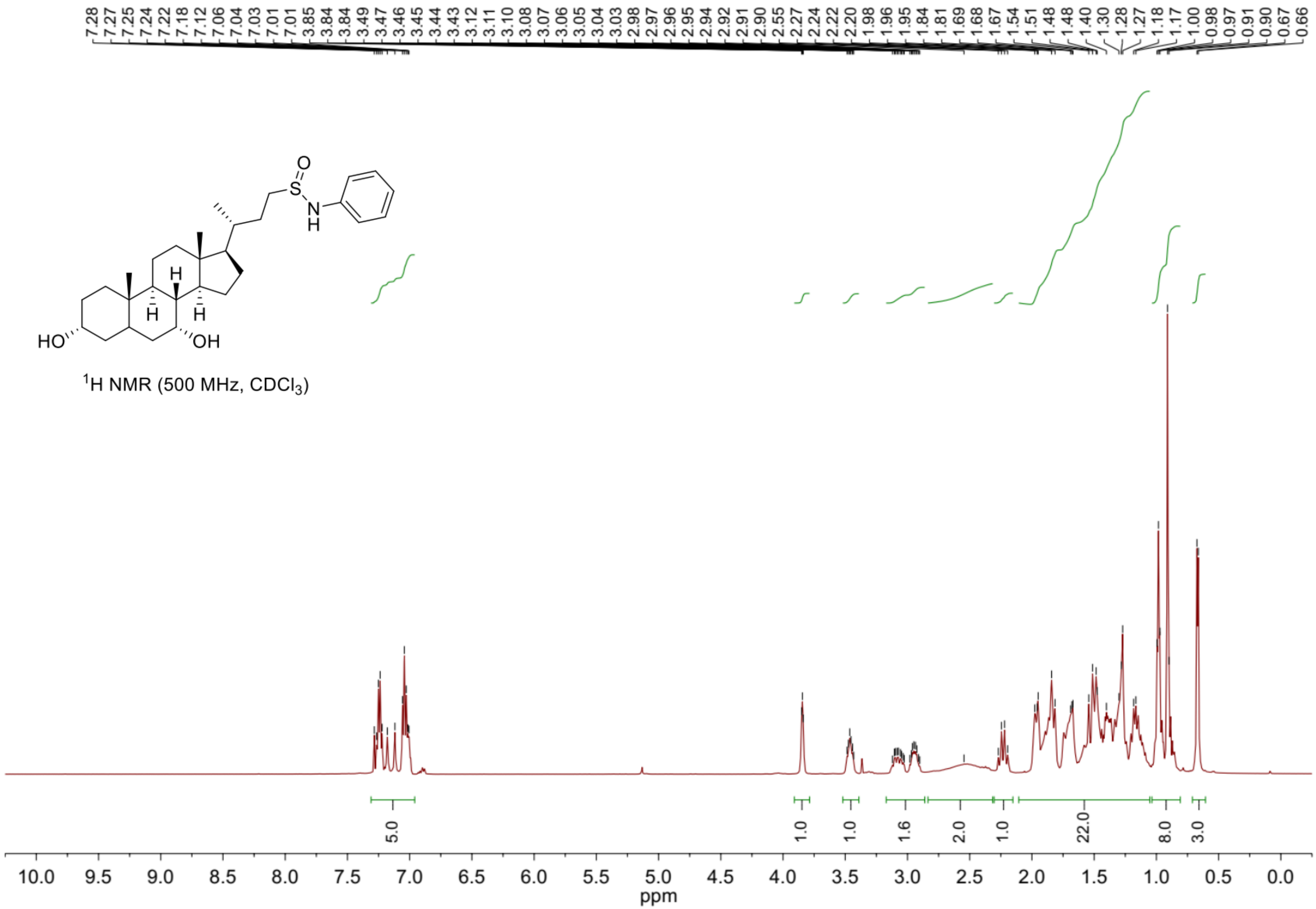

Go to table of contents

(3*R*)-3-((3*R*,7*R*,8*R*,9*S*,10*S*,13*R*,14*S*,17*R*)-3,7-Dihydroxy-10,13-dimethylhexadecahydro-1*H*-cyclopenta[a]phenanthren-17-yl)-*N*-phenylbutane-1-sulfinamide (7m)

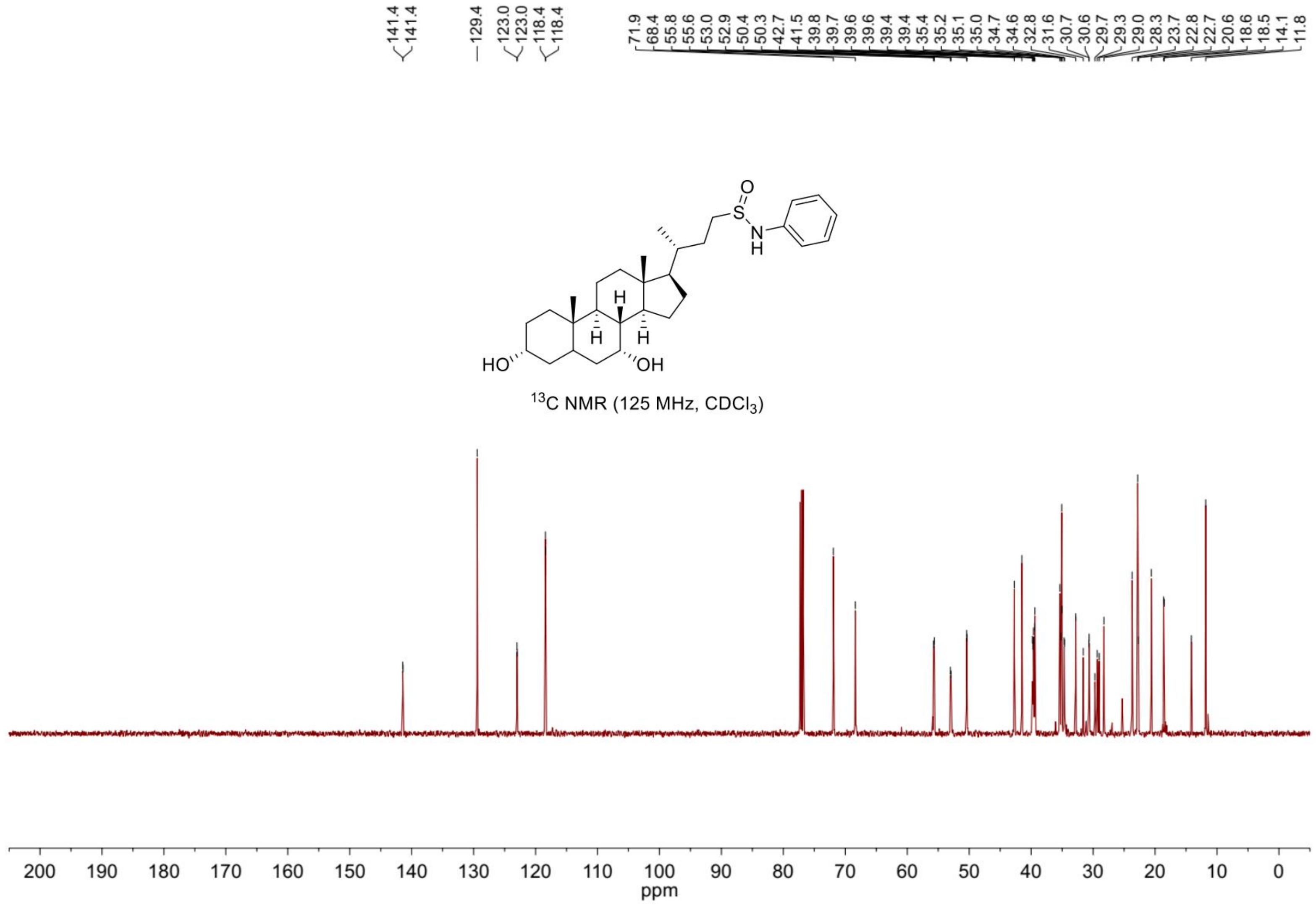

Go to table of contents

1-Cyclopropyl-*N*-phenylmethanesulfinamide (9) and *N*-phenylbut-3-ene-1-sulfinamide (9a)

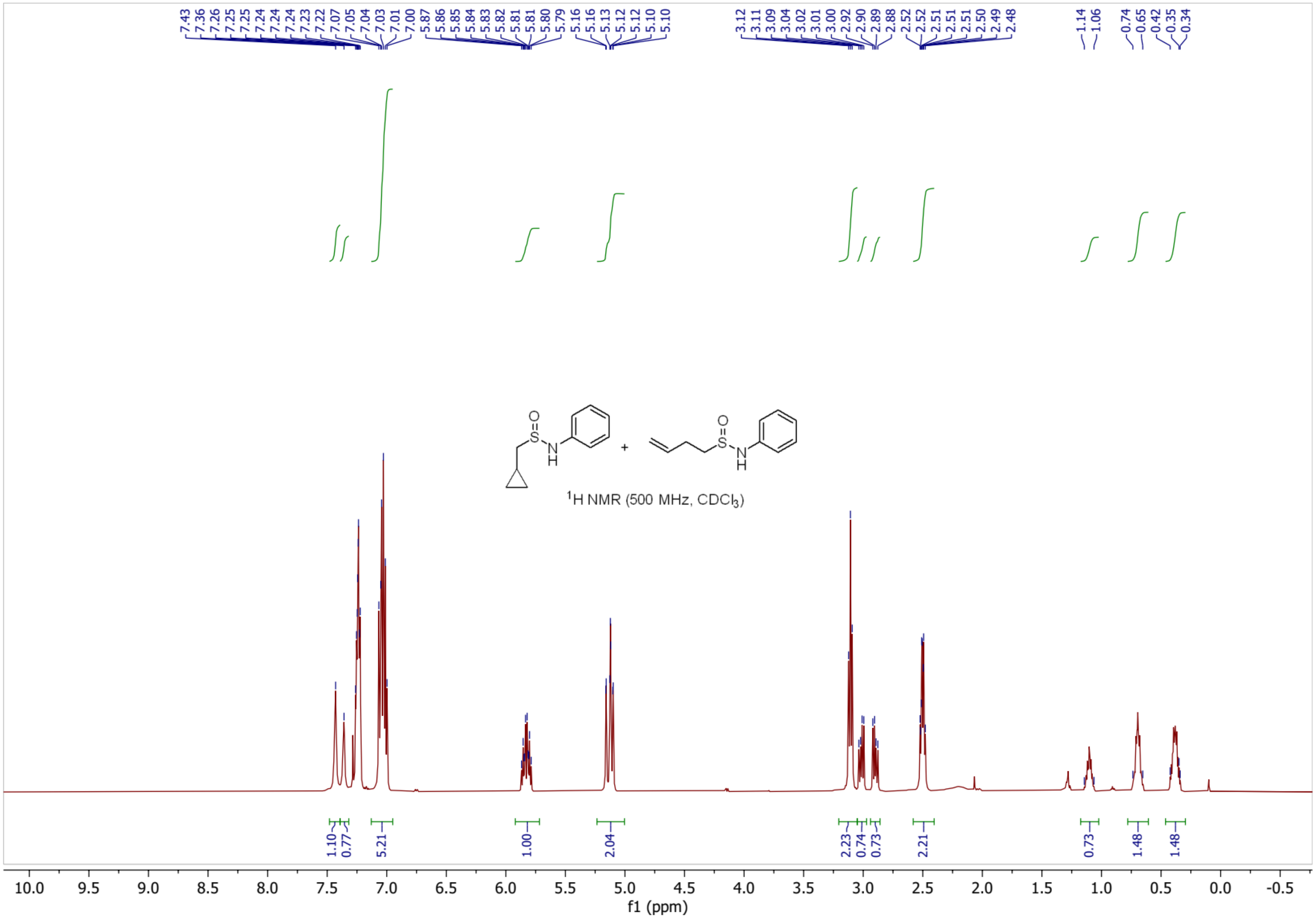

Go to table of contents

1-Cyclopropyl-*N*-phenylmethanesulfinamide (9) and *N*-phenylbut-3-ene-1-sulfinamide (9a)

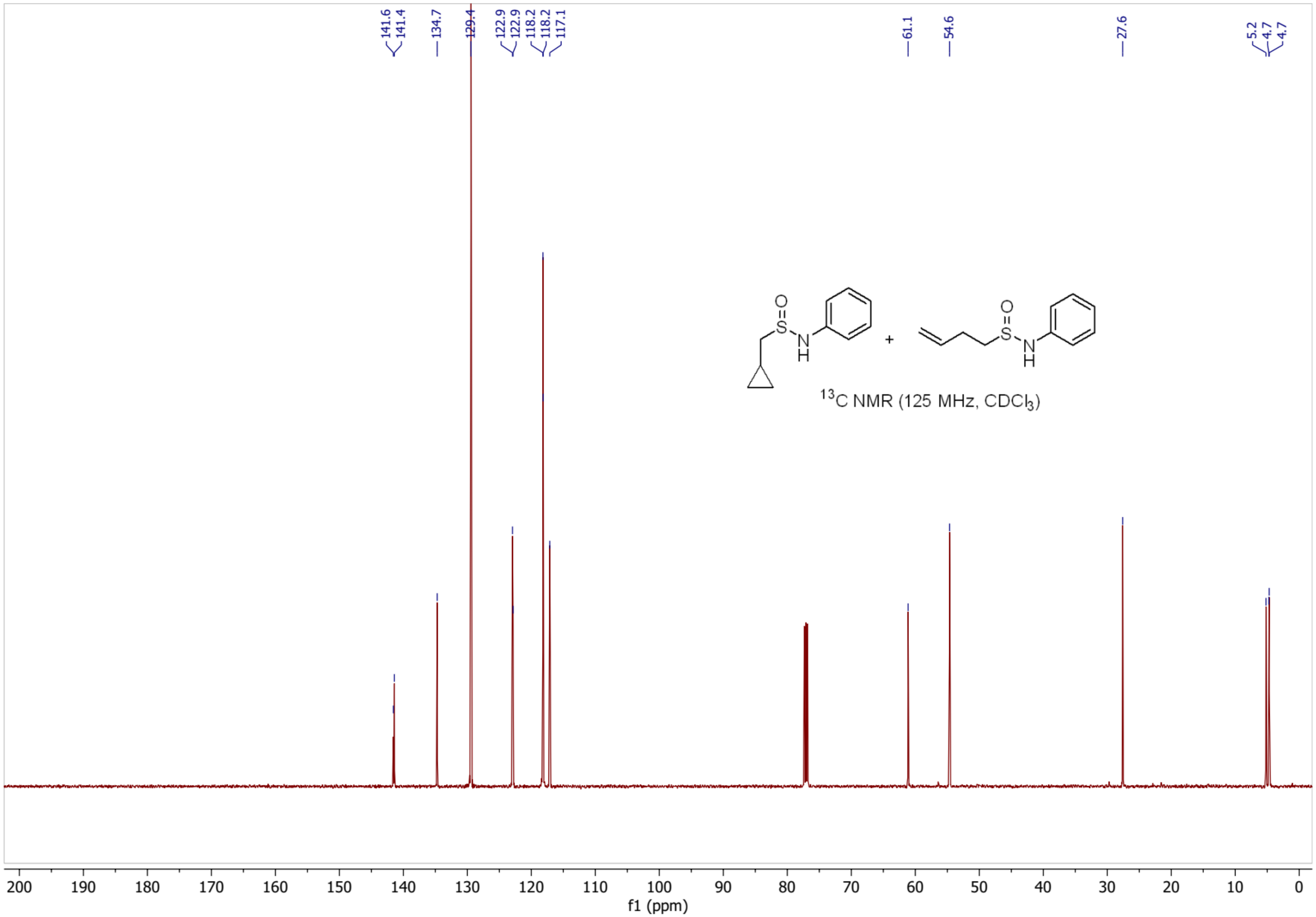

Go to table of contents

N -(4-chlorophenethyl)-2-methylpropane-1-sulfinamide (12a)

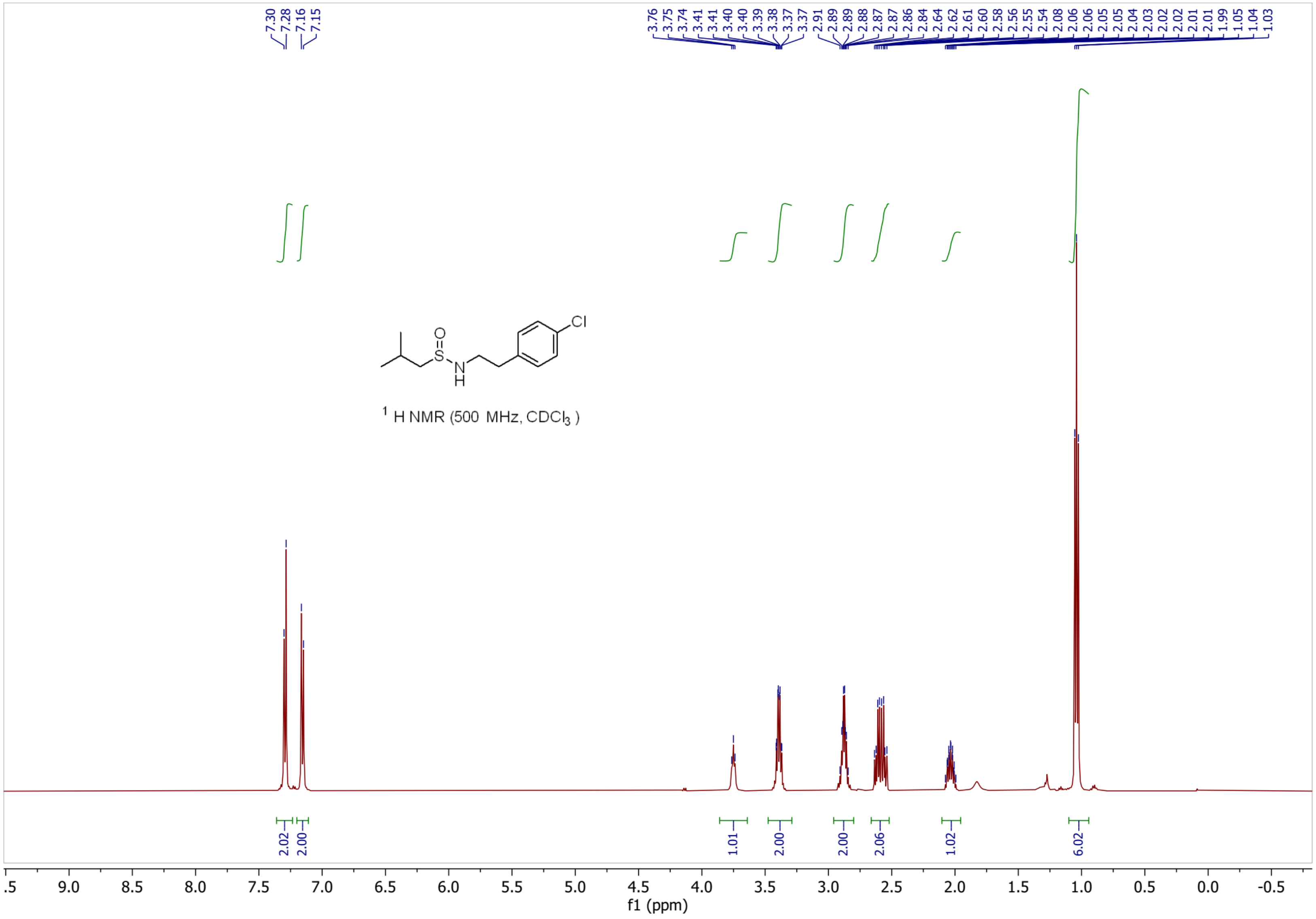

Go to table of contents

N-(4-chlorophenethyl)-2-methylpropane-1-sulfinamide (12a)

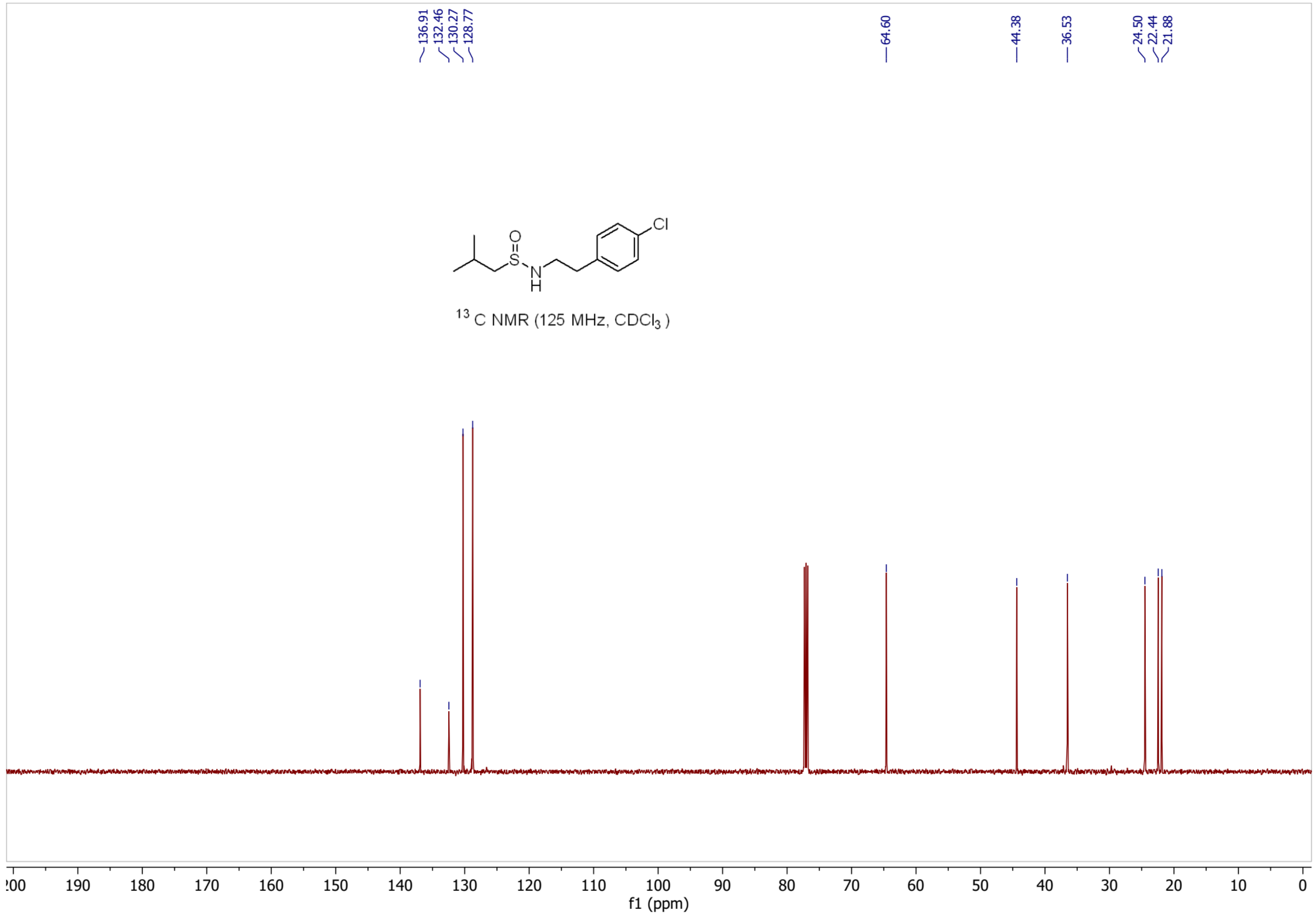

Go to table of contents

N-(3,5-difluorophenyl)-2-methylpropane-1-sulfinamide (12b)

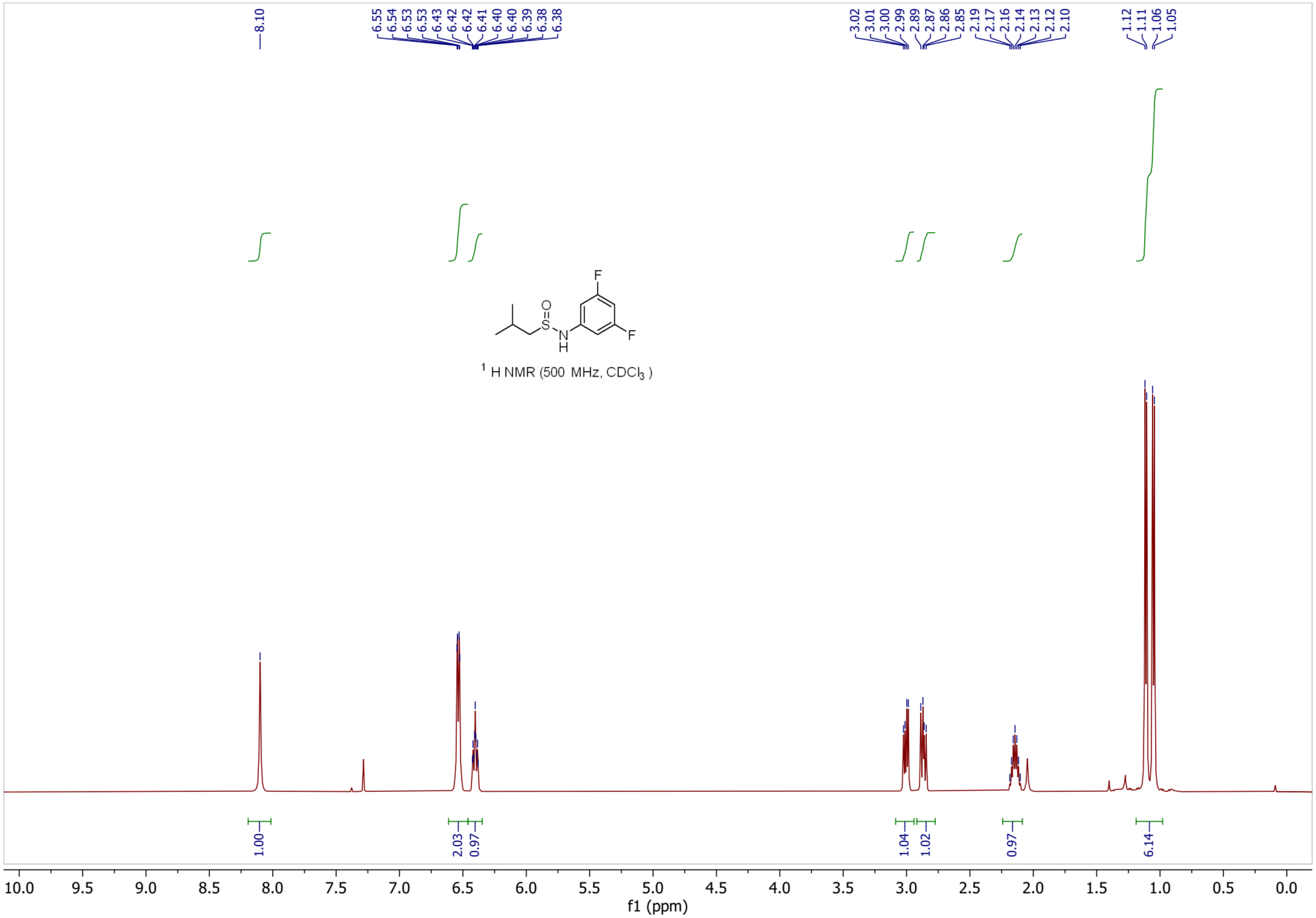

Go to table of contents

N-(3,5-difluorophenyl)-2-methylpropane-1-sulfinamide (12b)

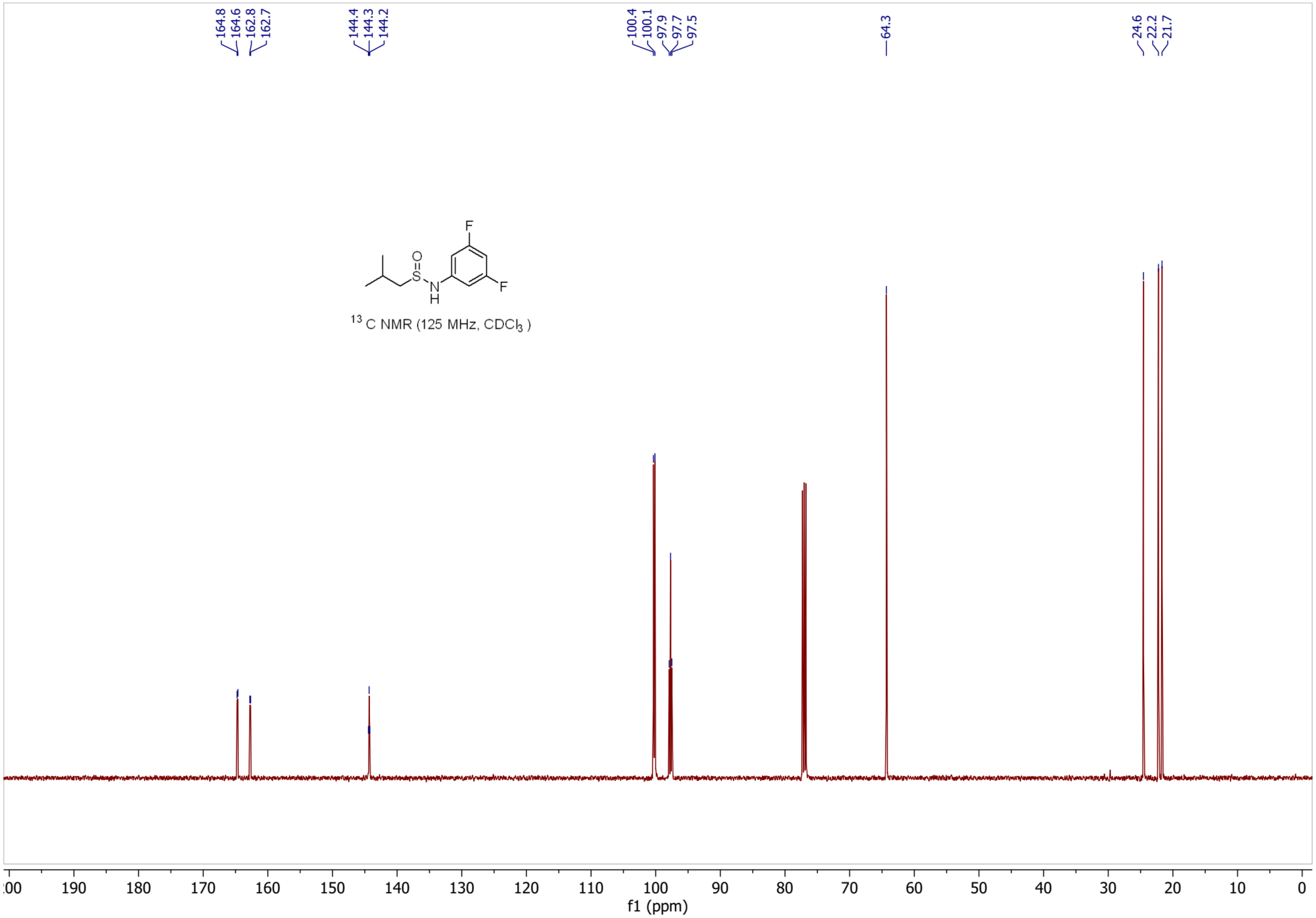

Go to table of contents

N-(4-fluorophenethyl)ethanesulfinamide (12c)

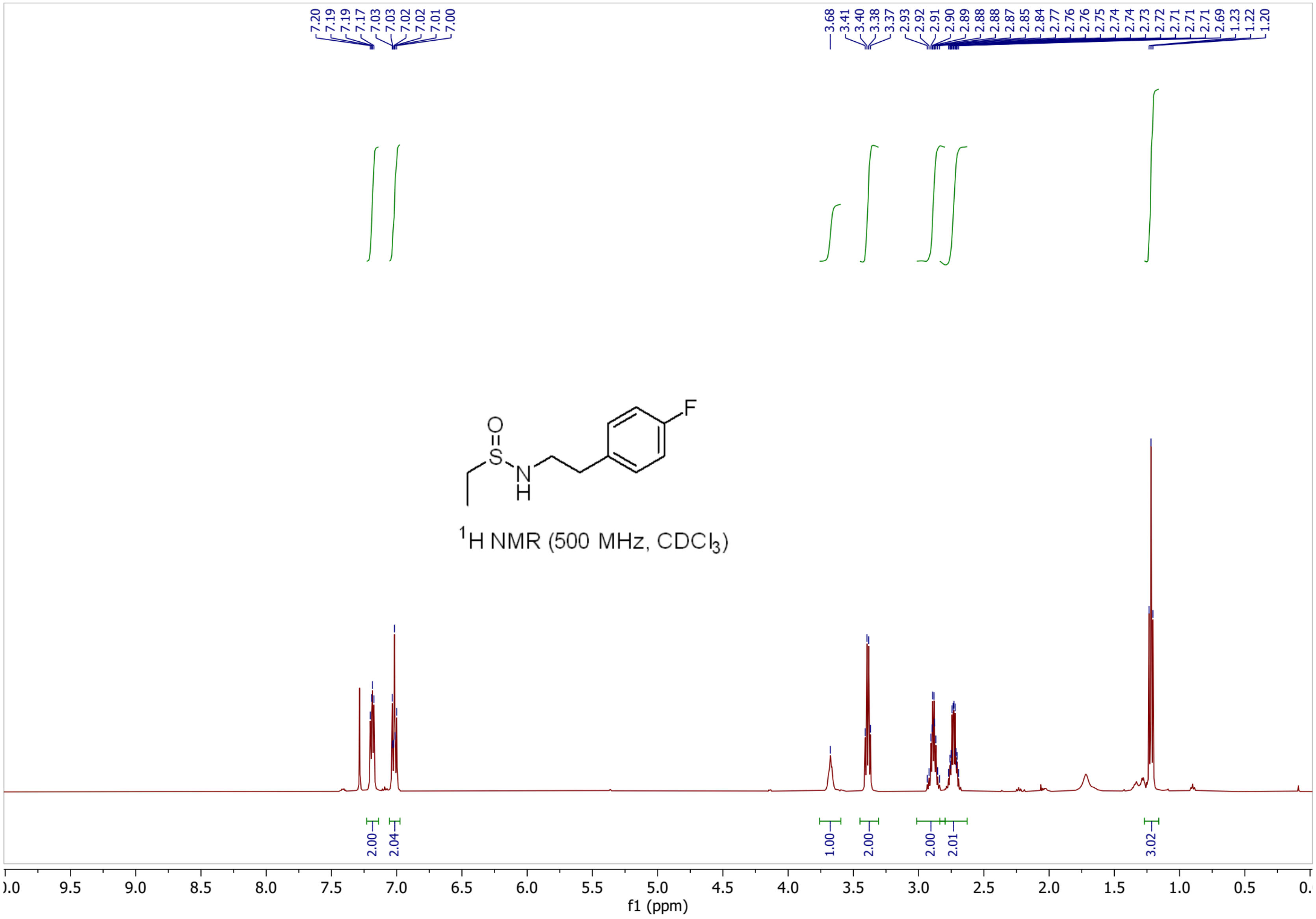

Go to table of contents

N-(4-fluorophenethyl)ethanesulfinamide (12c)

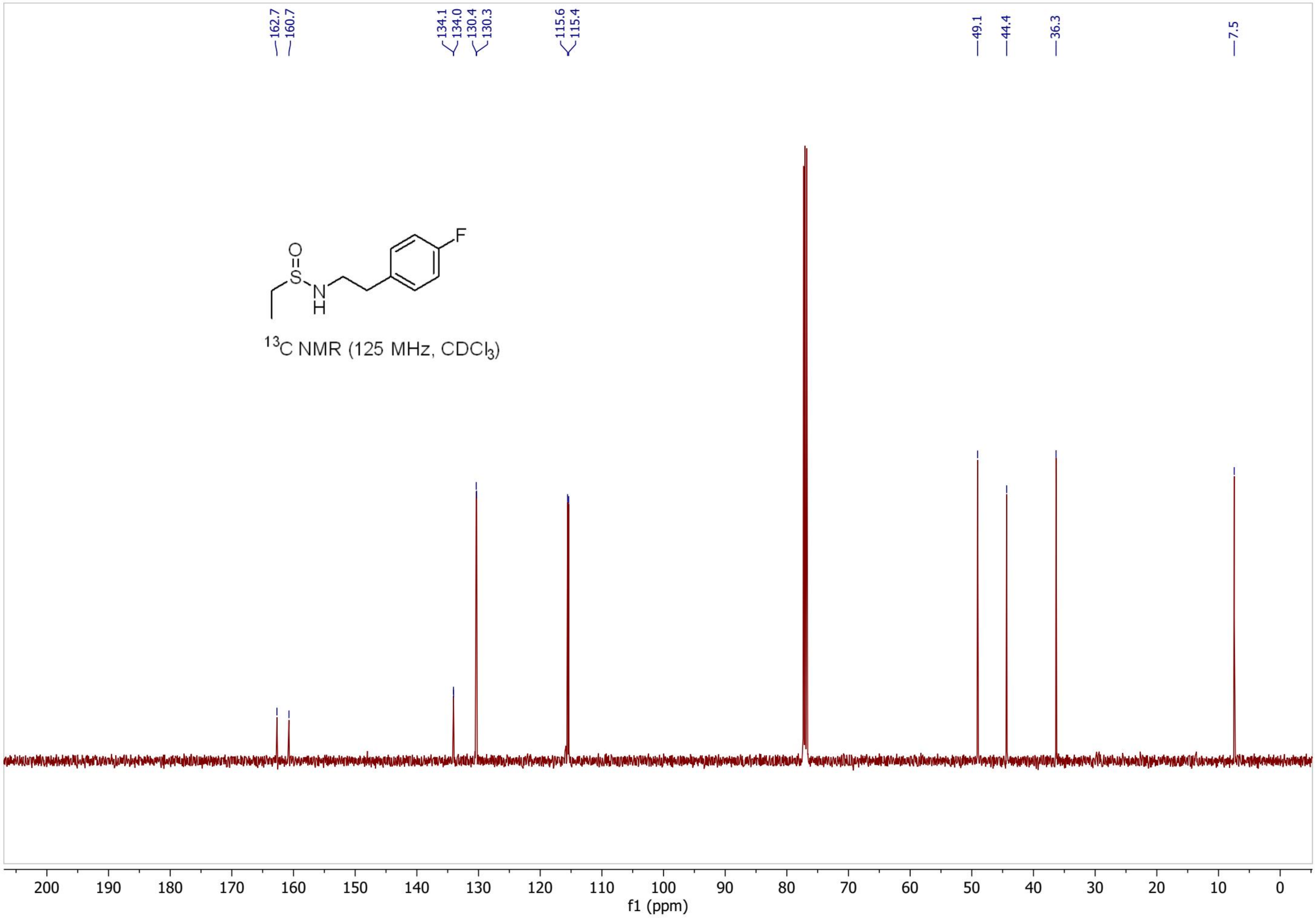

Go to table of contents

Methyl 3-((isobutylsulfinyl)amino)thiophene-2-carboxylate (12d)

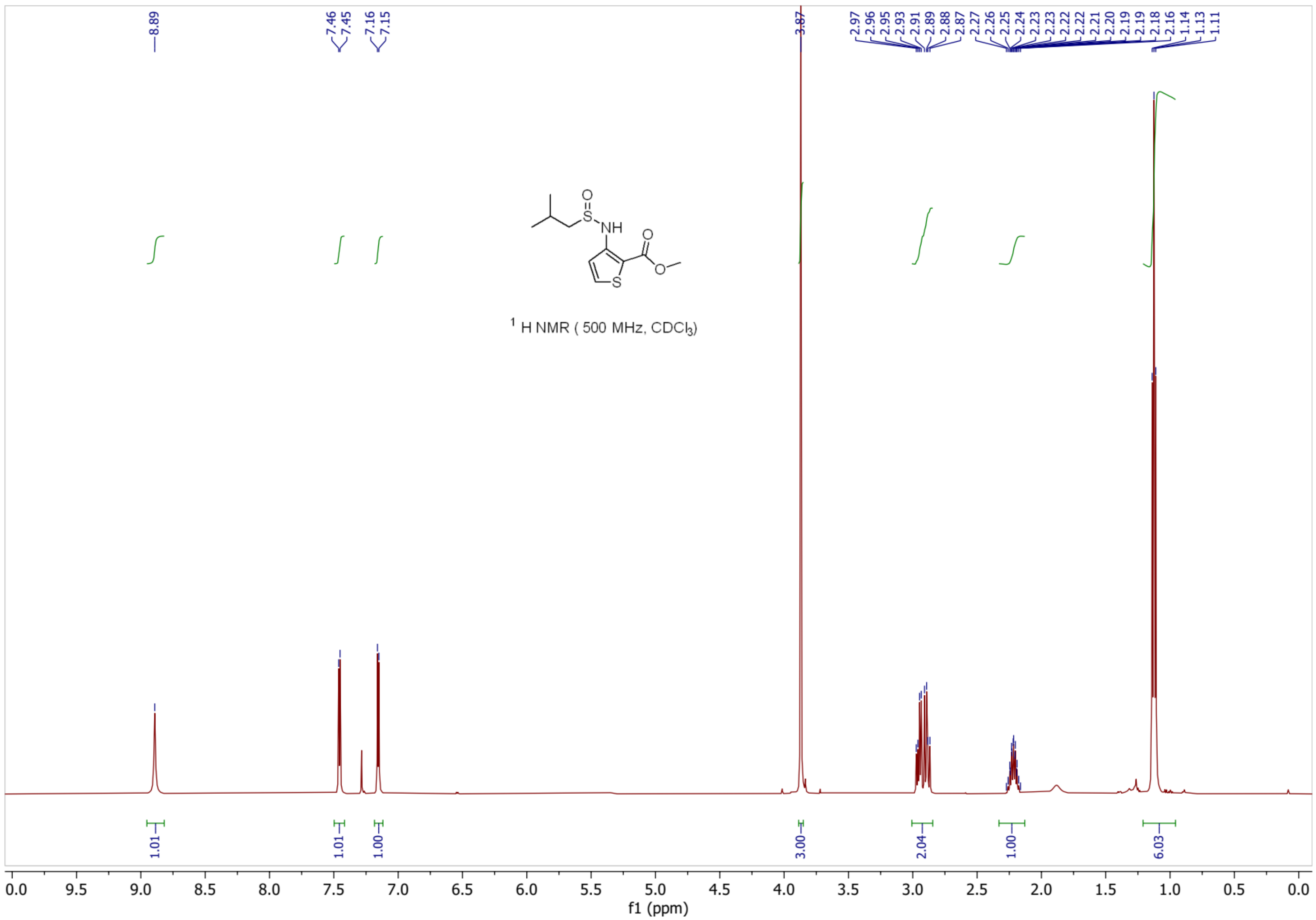

Go to table of contents

Methyl 3-((isobutylsulfinyl)amino)thiophene-2-carboxylate (12d)

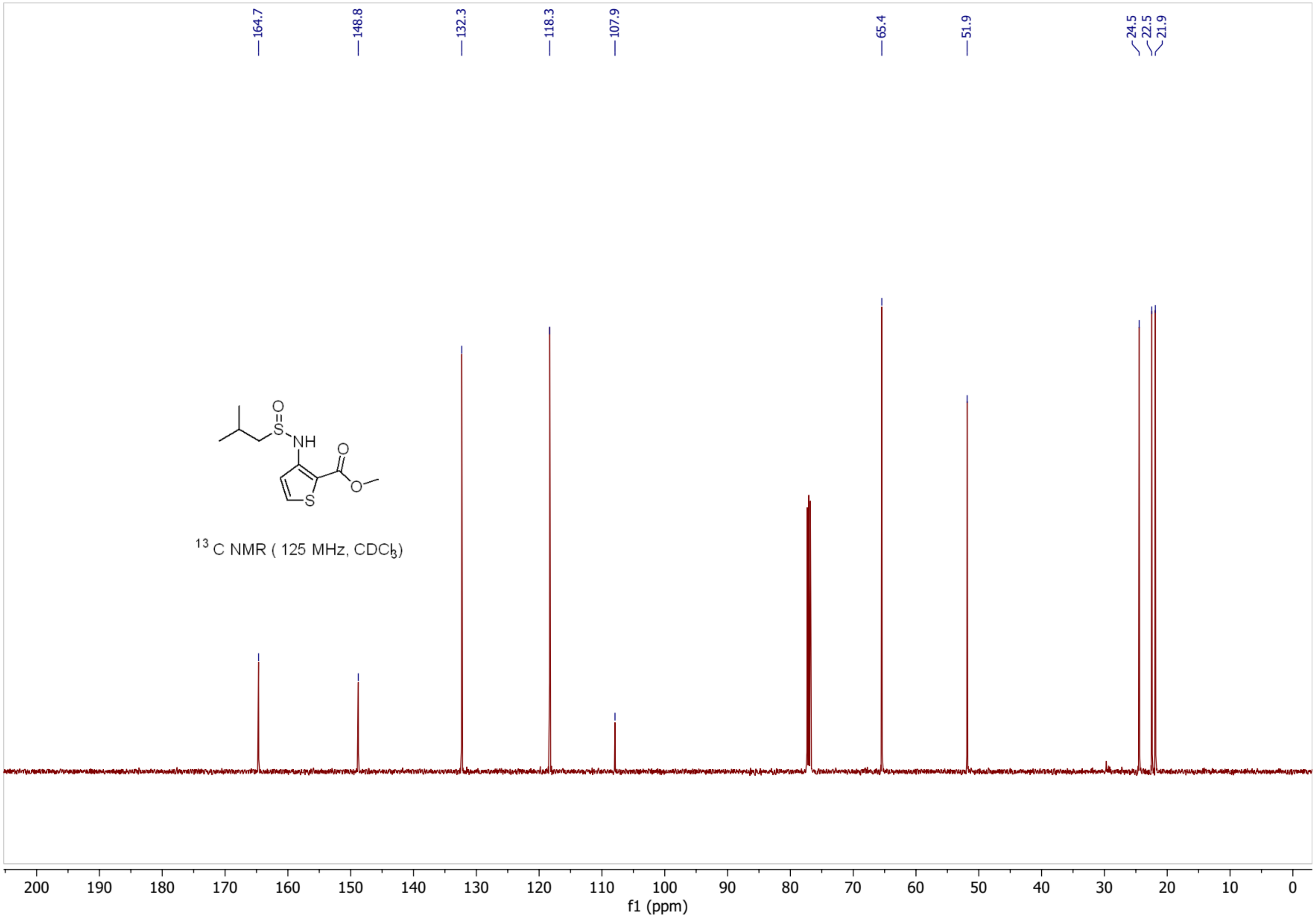

Go to table of contents

N-(3-bromo-4-methylphenyl)ethanesulfinamide (12e)

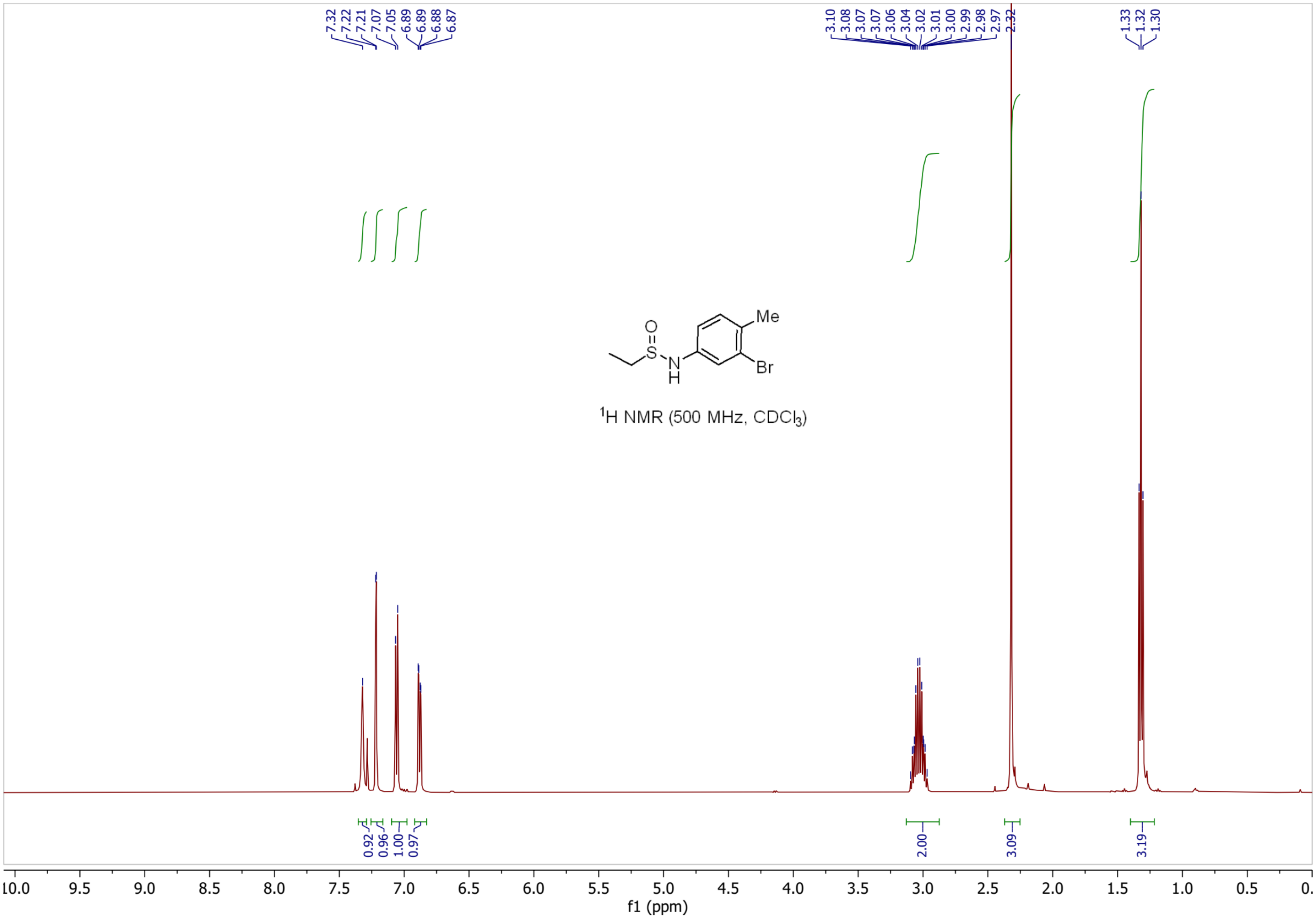

Go to table of contents

N-(3-bromo-4-methylphenyl)ethanesulfinamide (12e)

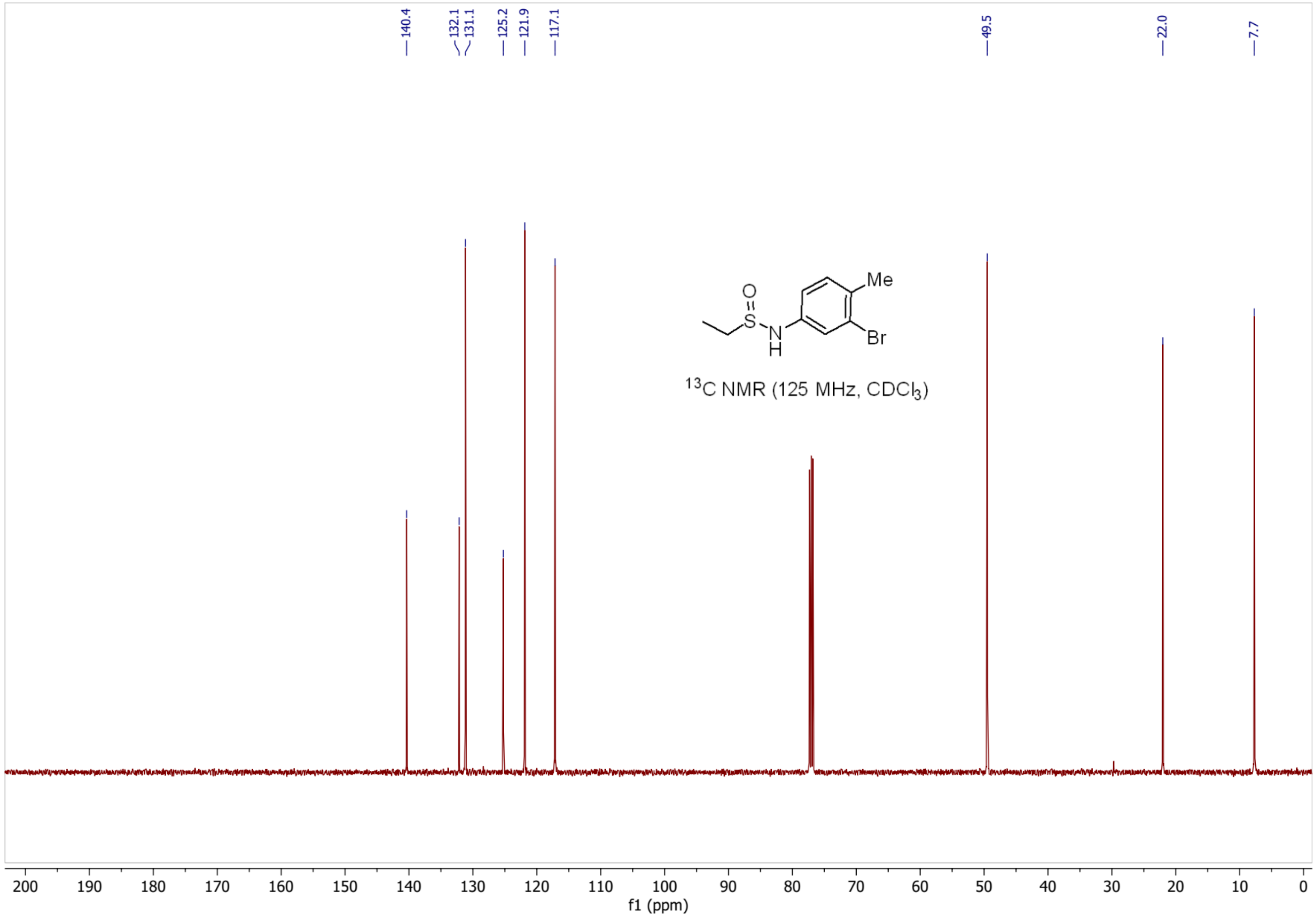

Go to table of contents

References

1. M. Newcomb, N. Tanaka, A. Bouvier, C. Tronche, J. H. Horner, O. M. Musa, F. N. Martinez, *J. Am. Chem. Soc.* **1996**, *118*, 8505–8506.
2. F. A. Saito, *Angew. Chem. Int. Ed.* **2022**, *61*, e202213872.
3. L. Li, S. hang, Y. Chen, X. Cui, G. Zhao, Z. Tang, G. Li, *ACS Catal.* **2022**, *12*, 15334–15340.
4. M. Ding, Z. X. Zhang, T. Q. Davies, M. C. Willis, *Org. Lett.* **2022**, *24*, 1711–1715.
5. Gaussian 16, Revision A.03, M. J. Frisch, G. W. Trucks, H. B. Schlegel, G. E. Scuseria, M. A. Robb, J. R. Cheeseman, G. Scalmani, V. Barone, G. A. Petersson, H. Nakatsuji, X. Li, M. Caricato, A. V. Marenich, J. Bloino, B. G. Janesko, R. Gomperts, B. Mennucci, H. P. Hratchian, J. V. Ortiz, A. F. Izmaylov, J. L. Sonnenberg, D.Williams-Young, F. Ding, F. Lipparini, F. Egidi, J. Goings, B. Peng, A. Petrone, T. Henderson, D. Ranasinghe, V. G. Zakrzewski, J. Gao, N. Rega, G. Zheng, W. Liang, M. Hada, M. Ehara, K. Toyota, R. Fukuda, J. Hasegawa, M. Ishida, T. Nakajima, Y. Honda, O. Kitao, H. Nakai, T. Vreven, K. Throssell, J. A. Montgomery, Jr., J. E. Peralta, F. Ogliaro, M. J. Bearpark, J. J. Heyd, E. N. Brothers, K. N. Kudin, V. N. Staroverov, T. A. Keith, R. Kobayashi, J. Normand, K. Raghavachari, A. P. Rendell, J. C. Burant, S. S. Iyengar, J. Tomasi, M. Cossi, J. M. Millam, M. Klene, C. Adamo, R. Cammi, J. W. Ochterski, R. L. Martin, K. Morokuma, O. Farkas, J. B. Foresman and D. J. Fox, Gaussian, Inc., Wallingford CT, **2016**.
6. S. Grimme, C. Bannwarth, P. Shushkov, *J Chem. Theory Comput.* **2017**, *13*, 1989–2009.
7. Y. Shao, Z. Gan, E. Epifanovsky, A. T. B. Gilbert, M. Wormit, J. Kussmann, A. W. Lange, A. Behn, J. Deng, X. Feng, D. Ghosh, M. Goldey, P. R. Horn, L. D. Jacobson, I. Kaliman, R. Z. Khaliullin, T. Kuś, A. Landau, J. Liu, E. I. Proynov, Y. M. Rhee, R. M. Richard, M. A. Rohrdanz, R. P. Steele, E. J. Sundstrom, H. L. Woodcock, P. M. Zimmerman, D. Zuev, B. Albrecht, E. Alguire, B. Austin, G. J. O. Beran, Y. a. Bernard, E. Berquist, K. Brandhorst, K. B. Bravaya, S. T. Brown, D. Casanova, C.-M. Chang, Y. Chen, S. H. Chien, K. D. Closser, D. L. Crittenden, M. Diedenhofen, R. A. DiStasio, H. Do, A. D. Dutoi, R. G. Edgar, S. Fatehi, L. Fusti-Molnar, A. Ghysels, A. Golubeva-Zadorozhnaya, J. Gomes, M. W. D. Hanson-Heine, P. H. P. Harbach, A. W. Hauser, E. G. Hohenstein, Z. C. Holden, T.-C. Jagau, H. Ji, B. Kaduk, K. Khistyaev, J. Kim, J. Kim, R. A. King, P. Klunzinger, D. Kosenkov, T. Kowalczyk, C. M. Krauter, K. U. Lao, A. D. Laurent, K. V. Lawler, S. V. Levchenko, C. Y. Lin, F. Liu, E. Livshits, R. C. Lochan, A. Luenser, P. Manohar, S. F. Manzer, S.-P. Mao, N. Mardirossian, A. V. Marenich, S. A. Maurer, N. J. Mayhall, E. Neuscamman, C. M. Oana, R. Olivares-Amaya, D. P. O'Neill, J. A. Parkhill, T. M. Perrine, R. Peverati, A. Prociuk, D. R. Rehn, E. Rosta, N. J. Russ, S. M. Sharada, S. Sharma, D. W. Small, A. Sodt, T. Stein, D. Stück, Y.-C. Su, A. J. W. Thom, T. Tsuchimochi, V. Vanovschi, L. Vogt, O. Vydrov, T. Wang, M. A. Watson, J. Wenzel, A. White, C. F. Williams, J. Yang, S. Yeganeh, S. R. Yost, Z.-Q. You, I. Y. Zhang, X. Zhang, Y. Zhao, B. R. Brooks, G. K. L. Chan, D. M. Chipman, C. J. Cramer, W. A. Goddard, M. S. Gordon, W. J. Hehre, A. Klamt, H. F. Schaefer, M. W. Schmidt, C. D. Sherrill, D. G. Truhlar, A. Warshel, X. Xu, A. Aspuru-Guzik, R. Baer, A. T. Bell, N. A. Besley, J.-D. Chai, A. Dreuw, B. D. Dunietz, T. R. Furlani, S. R. Gwaltney, C.-P. Hsu, Y. Jung, J. Kong, D. S. Lambrecht, W. Liang, C. Ochsenfeld, V. A. Rassolov, L. V. Slipchenko, J. E. Subotnik, T. Van Voorhis, J. M. Herbert, A. I. Krylov, P. M. W. Gill, M. Head-Gordon, *Mol. Phys.* **2015**, *113*, 184–215.
8. CYLview, 1.0b, C. Y. Legault, Université de Sherbrooke, **2009** (http://www.cylview.org).
9. W. Humphrey, A. Dalke, K. Schulten, *J. Molec. Graphics* **1996**, *14*, 33–38.
10. T. Lu, F. Chen, *J. Comput. Chem.* **2012**, *33*, 580–592.

11. Chemcraft - graphical software for visualization of quantum chemistry computations. https://www.chemcraftprog.com.

12. H. S. Yu, X. He, S. L. Li, D. G. Truhlar, *Chem. Sci.* **2016**, *7*, 5032–5051.

13. A. Schaefer, C. Huber, and R. Ahlrichs, *J. Chem. Phys.* **1994**, *100*, 5829–5835.

14. A. V. Marenich, C. J. Cramer, D. G. Truhlar, *J. Phys. Chem. B* **2009**, *113*, 6378–6396.

15. G. Luchini, J. V. Alegre-Requena, Y. Guan, I. Funes-Ardoiz and R. S. Paton, GoodVibes: GoodVibes, 2019, v3.0.1.

16. Y. Zhao and D. G. Truhlar, *J. Phys. Chem. A* **2005**, *109*, 5656.

17. S. Grimme, J. Antony, S. Ehrlich and H. Krieg, *J. Chem. Phys.* **2010**, *132*, 154104.

18. A. Hellweg, D. Rappoport, *Phys. Chem. Chem. Phys.* **2015**, *17*, 1010–1017.

19. B. P. Pritchard, D. Altarawy, B. Didier, T. D. Gibson, T. L. Windus, *J. Chem. Inf. Model.* **2019**, *59*, 4814–4820.

20. F. M. Bickelhaupt, K. N. Houk, *Angew. Chem., Int. Ed.*, **2017**, *56*, 10070-10086.

21. V. D. Nguyen, G. C. Haug, S. G. Greco, R. Trevino, G. B. Karki, H. D. Arman, O. V. Larionov, *Angew. Chem., Int. Ed.*, **2022**, *61*, 1–9.

22. R. Z. Khaliullin, E. A. Cobar, R. C. Lochan, A. T. Bell, M. Head-Gordon, *J. Phys. Chem. A* **2007**, *111*, 8753–8765.

23. S. Jin, G. C. Haug, R. Trevino, V. D. Nguyen, H. D. Arman, O. V. Larionov, *Chem. Sci.* **2021**, *12*, 13914–13921.

24. V. T. Nguyen, G. C. Haug, V. D. Nguyen, N. T. H. Vuong, G. B. Karki, H. D. Arman, O. V. Larionov, *Chem. Sci.* **2022**, *13*, 4170–4179.

25. https://github.com/patonlab/DBSTEP/tree/master

26. F. Pedregosa, G. Varoquaux, A. Gramfort, V. Michel, B. Thirion, O. Grisel, M. Blondel, P. Prettenhofer, R. Weiss, V. Dubourg, J. Vanderplas, A. Passos, D. Cournapeau, M. Brucher, M. Perrot, E. Duchesnay, *J. Mach. Learn. Res.* **2011**, *12*, 2825–2830.

27. T. Q. Chen, C. Guestrin, *Kdd'16: Proceedings of the 22nd Acm Sigkdd International Conference on Knowledge Discovery and Data Mining* **2016**, *8*, 785–794.

28. H. Drucker, C. C. Burges, L. Kaufman, A. J. Smola, V. N. Vapnik, "Support Vector Regression Machines", in *Adv. Neural. Inf. Process Syst. (NIPS 1996)* **1997**, *9*, 155–161.

29. U. Stańczyk, L. C. Jain, Eds. *Feature Selection for Data and Pattern Recognition*, *Studies in Computational Intelligence 584*, Springer-Verlag Berlin Heidelberg, **2015**.

30. M. Skurichina, R. P. W. Duin, Bagging for linear classifiers. *Pattern Recogn.* **1998**, *31*, 909–930.

31. L., Breiman, J. H. Friedman, R. A. Olshen, C. J. Stone, *Classification, and regression trees*. Routledge, **2017**.

32. P. Geurts, D. Ernst, L. Wehenkel, *Mach. Learn.* **2006**, *63*, 3-42.

33. J. H. Friedman, *Ann. Stat.* **2001**, 1189-1232.

34. G. C. Cawley, N. L. Talbot, *Neural Process* **2002**, *16*, 293-302.

35. E. Fix, J. L. Hodges, *Int. Stat. Rev.* **1989**, *57*, 238–247.

36. G. Biau, *J. Mach. Learn. Res.* **2012**, *13*, 1063–1095.

Go to table of contents

37. C. García, J. García, M. López Martín, R. Salmerón, *J. Appl. Stat.* **2015**, *42*, 648–661.
38. Y. Zhang, L. Chen, *Theor. Econ. Lett*. **2012**, *11*, 258-267
